# Enantioselective Synthesis of Highly Substituted Fluoroalkylated Benzopyranones and 3-Coumaranones via *N*-Heterocyclic Carbene-Catalyzed Intramolecular Annulations

**DOI:** 10.1021/acs.joc.3c01099

**Published:** 2023-10-04

**Authors:** Izabela Barańska, Katarzyna Rafińska, Zbigniew Rafiński

**Affiliations:** Faculty of Chemistry, Nicolaus Copernicus University in Torun, 7 Gagarin Street, Torun 87-100, Poland

## Abstract

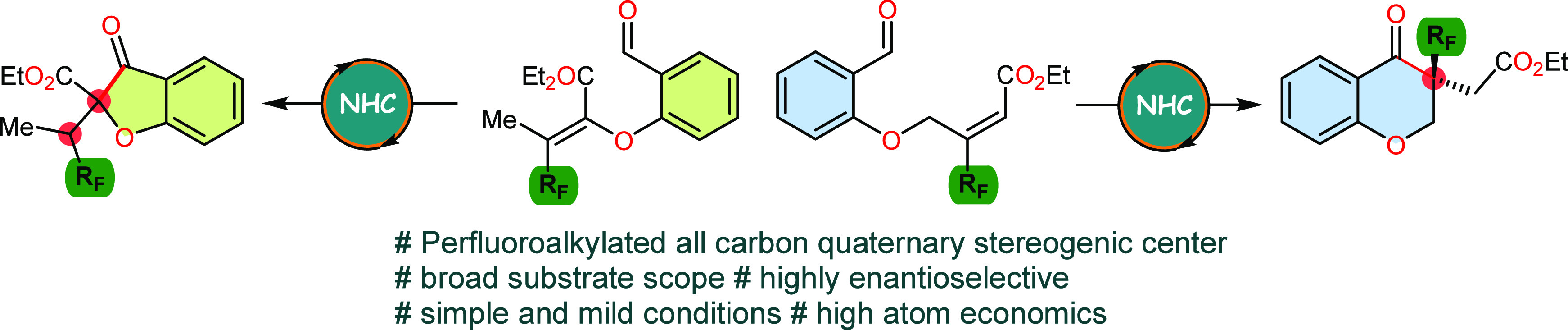

A highly enantioselective intramolecular NHC-catalyzed approach for the synthesis of fluoroalkylated benzopyranones and 3-coumaranones with all-carbon quaternary stereocenters is presented. This reaction is catalyzed by *N*-heterocyclic carbenes (NHCs) and involves annulation reactions between in situ generated acyl anion intermediates and highly substituted trifluoromethyl-β,β-disubstituted Michael acceptors. The method can also be extended to perfluoroalkyl homologues.

## Introduction

The introduction of fluorine atoms into the structure of organic compounds significantly impacts their chemical, physical, and biological properties. This influence is evident in the widespread applications of fluorinated organic compounds in medicinal chemistry (>20%, including top sellers), agrochemistry (>30%, plant protection products), materials chemistry, and other scientific fields.^1^ In contrast, only 30 naturally occurring organic compounds containing a carbon–fluorine bond have been discovered.^2^ Unfortunately, the synthesis of fluorinated organic compounds is highly demanding. Asymmetric reactions involving fluoroorganic reagents or substrates that enable the creation of a stereogenic carbon center with a trifluoromethyl group are highly challenging. There are two complementary strategies for their synthesis: the first one involves the direct introduction of the trifluoromethyl group through nucleophilic, electrophilic, or radical reagents. The second approach employs fluorinated substrates such as trifluorinated-containing building blocks to construct chiral fluorinated compounds.^3−6^ In this context, enantiopure trifluoromethylated molecules are at the forefront of innovation in modern organofluorine chemistry and garner high interest due to their increasing occurrence in a wide range of biologically active compounds.

We focused our attention on the strategy using CF_3_ substrates, which undergo intramolecular conversion to intriguing chiral products through an organocatalytic pathway using nucleophilic NHC catalysts.^7−17^

**Figure 1:**
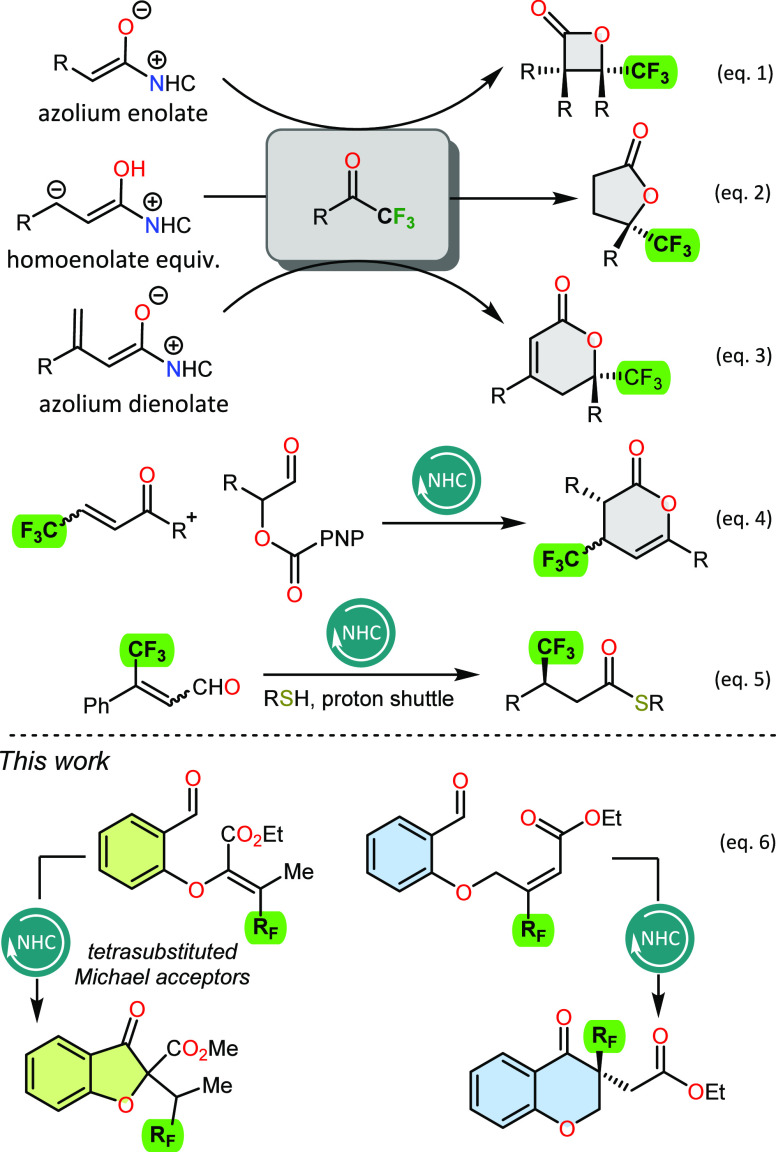
NHC-mediated synthesis of fluoroorganic compounds.

Currently, the most widely recognized transformations enable effective trifluoromethylation in the intermolecular variant, where appropriate ketones and reactive intermediate products, such as azolium enolate (Figure 1, eq 1),^18,19^ dienolate (Figure 1, eq 3),^20,21^ or homoenolate equivalents (Figure 1, eq 2)^22^ formed as a result of NHC activation. Recently, Smith et al. discovered that trifluoromethyl chalcones can lead to NHC-mediated annulation of dihydropyranones (Figure 1, eq 4).^23^ In 2017, Huang and co-workers demonstrated an elegant enantioselective β-protonation of enals via a shuttling strategy (Figure 1, eq 5).^24^ However, to the best of our knowledge, asymmetric intramolecular reactions involving NHC-mediated acyl anion chemistry and fluoroorganic substrates remain unreported in the literature (Figure 1, eq 6). The enantioselective formation of asymmetric C–CF_3_ bonds is a much more ambitious field of research that has experienced tremendous development since the beginning of this century. Asymmetric perfluoroalkylation, including trifluoromethylation, remains a difficult and challenging area due to possible interactions of fluorine atoms with the chiral catalyst, the influence of fluoroalkene geometry, and the sometimes laborious and tedious procedures of synthetic substrates. This makes the asymmetric synthesis of vitally important fluorinated building blocks an essential aspect of medicinal chemistry. To address this demand and as part of our continued interest in asymmetric carbene organocatalysis and the synthesis of biologically relevant molecules,^25−30^ we envisioned that more challenging β,β-disubstituted Michael acceptors with a trifluoromethyl source could undergo intramolecular enantioselective organocatalytic annulation reactions to a resulting all-carbon quaternary C–CF_3_ bond. This process could yield new types of chiral benzopyroanones and coumaranones in a highly stereocontrolled fashion.

## Results and Discussion

At the outset of this study, we investigated the annulation of salicylaldehyde-derived trifluoromethyl acrylate as a model substrate. We used morpholine-derived triazolium salts containing various chiral structural motifs in the presence of DIPEA as a base. Key results are briefly summarized in Table 1. A quick pre-catalyst screening revealed that NHCs generated from **A** and **C** are very reactive but selectivity remains low (entries 1,4). Camphor-derived triazolium salts **B1** and **B2** showed both low selectivity and catalytic activity (entries 2–3). Encouraging results were obtained using the verbenone-derived triazolium salt **D**, achieving a target reaction product with a 97% yield and 76% enantiomeric excess (entry 5). To our great delight, the aminoindanol motif of the NHC precatalyst could further increase the enantioselectivity and maintain the same level of reactivity (97%, 97% ee, entry 6). DIPEA was found to be the optimal base for this annulation reaction as the transformations performed using other organic (P_2_-Et, Piperidine, and DABCO) and inorganic (K_3_PO_4_, Cs_2_CO_3_) bases returned slightly inferior results (entries 7–12).

**Table 1:**
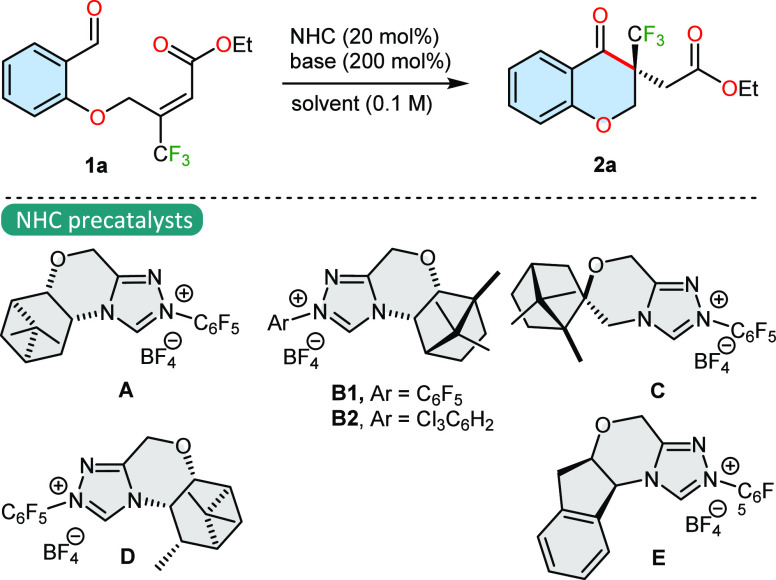
Optimization of the Reaction Parameters^a^

entry	preNHC	base	solvent	yield^b^(%)	*ee*^c^(%)
1	**A**	DIPEA	toluene	92	52
2	**B1**	DIPEA	toluene	52	12
3	**B2**	DIPEA	toluene	20	22
4	**C**	DIPEA	toluene	92	12
5	**D**	DIPEA	toluene	97	76
6	**E**	DIPEA	toluene	97	97
7	**E**	P_2_-Et	toluene	90	92
8	**E**	K_3_PO_4_	toluene	90	97
9	**E**	piperidine	toluene	95	97
10	**E**	DABCO	toluene	90	97
11	**E**	CH_3_CO_2_K	toluene	86	94
12	**E**	Cs_2_CO_3_	toluene	95	96
13	**E**	DIPEA	toluene	99	97
14	**E**	DIPEA	CH_2_Cl_2_	98	96
15	**E**	DIPEA	CPME	98	97
16	**E**	DIPEA	TAME	93	97
17	**E**	DIPEA	THF	95	97
18	**E**	DIPEA	*o*-xylene	>99	98
19^d^	**E**	DIPEA	*o*-xylene	90	96
20^e^	**E**	DIPEA	*o*-xylene	46	96

a Unless otherwise specified, the reaction was performed on a 0.1 mmol scale **1a** in solvent (1.0 mL) at room temperature.

b Yields of isolated products.

c ee values determined by HPLC on a chiral stationary column (see the Supporting Information).

d 2 mol % NHC **E**.

e 0.2 mol % NHC **E**. Abbreviations: phosphazene base P_2_-Et: 1-ethyl-2,2,4,4,4-pentakis(dimethylamino)-2λ5,4λ5-catenadi(phosphazene).

Solvents had little effect on the reaction outcome. The reaction proceeded smoothly in a variety of polar and nonpolar solvents (entries 14–17). The reduction in the quantity of the NHC catalyst allowed for the successful synthesis of the reaction products without a decrease in enantiomeric excess. However, it was observed that a decrease in the amount of catalyst was concomitant with a decrease in the overall yield of the reaction. Finally, *o*-xylene was the best one, yielding the expected trifluoromethylated chromanone quantitatively and with a high enantioselectivity of 98% (entry 18). With these optimized conditions in hand, we investigated the scope of the transformation and synthesized a panel of chiral trifluoromethylated benzopyranones bearing an all-carbon quaternary stereogenic center. As summarized in Table 2, various salicylaldehyde-derived β-trifluoromethyl acrylates with different substitution patterns in the aromatic ring reacted well to generate the desired chromanone derivatives containing a unique trifluoromethylated quaternary stereogenic center with excellent enantioselectivities.

**Table 2:**
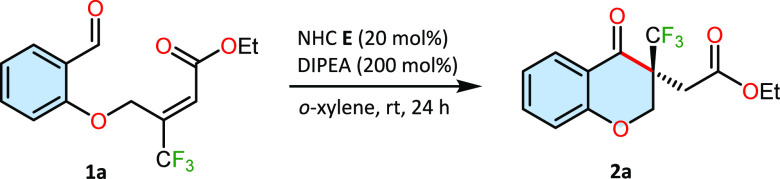

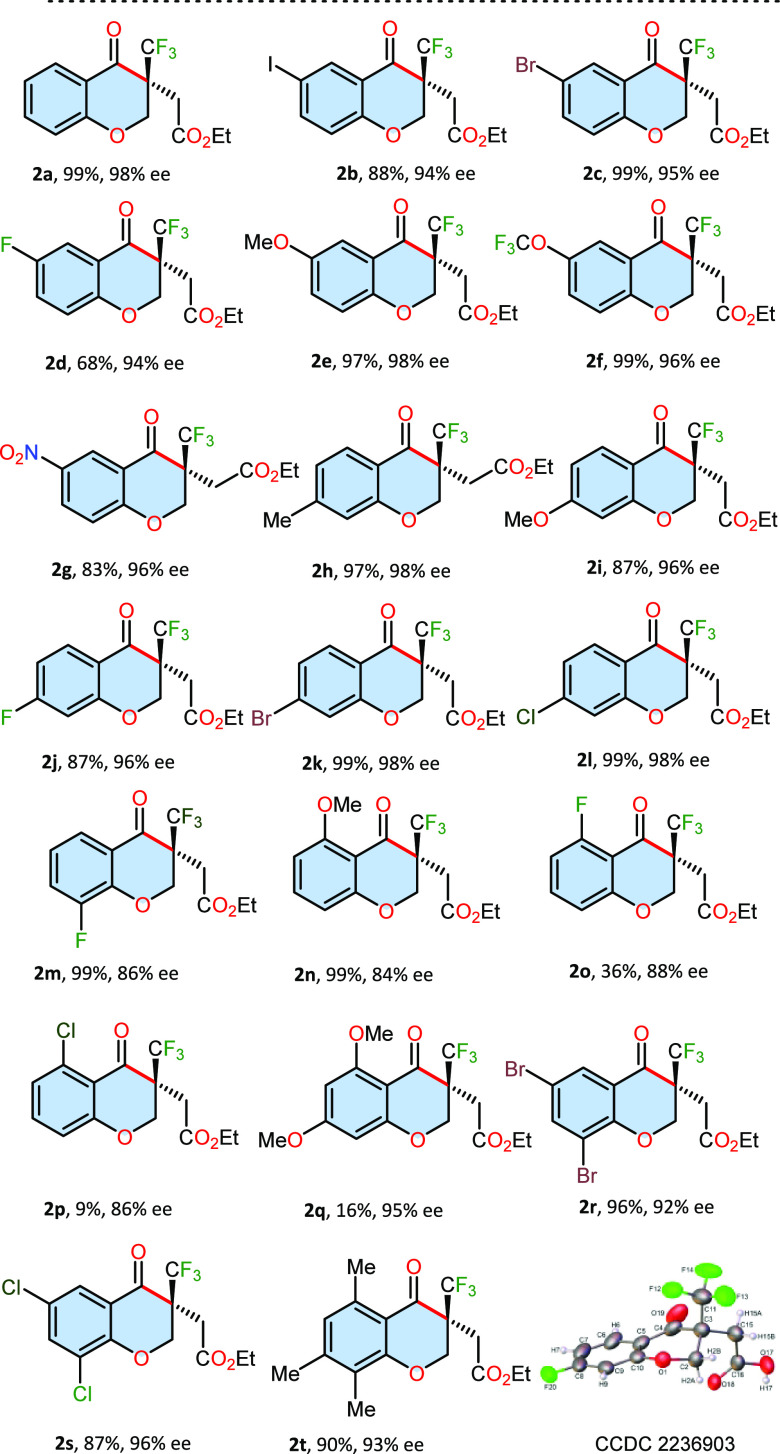
Substrate Scope of the NHC-Catalyzed Annulation for Constructing Benzopyranones Bearing a C–CF_3_ Quaternary Carbon Stereocenter^a^

a Unless otherwise specified, the reaction was performed on a 0.1 mmol scale in solvent (1.0 mL) at room temperature. Yields of isolated products. ee values determined by HPLC on a chiral stationary phase.

Generally, mono-, di-, and trisubstituted substrates were tolerated in this reaction model, affording their corresponding CF_3_-benzannulated products in low to excellent yields (9–99%) and high to excellent stereoselectivities (84–98% ee). The type of substituent used and its location in the aromatic ring were crucial from the point of view of reactivity. However, they had a weaker effect on the enantioselectivity of the annulation process. Substrates with electron-deficient groups, such as bromo-, iodo-, nitro-, and trifluorometoxy-, at the 6-position salicylaldehyde derivative (**2b**, **2c**, **2f**, and **2g**), reacted smoothly under optimized conditions to furnish the enantiopure β-CF_3_ γ-ketoesters derived from chromanone skeletons in high yields. A slight decrease in reactivity was observed for the 6-F derivative (**2d**). Incorporating a strong electron-donating OMe group at this position gave the corresponding product in a high yield and with enantiomeric excess (**2e**, 97%, 98% ee). Notably, regardless of the nature of the 7-position substituent, both electron-donating and withdrawing groups yielded the CF_3_-chromanone products in high yields and with excellent optical purities (**2h**–**2l**). Particularly interesting was the analysis of the influence of the substituent used in the 5-position of the aromatic ring on the reactivity and selectivity of the annulation due to the close proximity of activating and deactivating groups in an *ortho* orientation. Electron-withdrawing groups existing at the 5-position (**2o** and **2p**) resulted in decreased product yields. In addition, the influence of the size of the substituent is noticeable here; with the increase of its spatial volume, the reactivity decreases while maintaining unchanged stereoselectivity. Reactions using di- and trisubstituted substrates also yielded interesting results. For the 5,7-dimethoxy derivative **2q**, the target reaction product was obtained with a high 95% enantiomeric excess but with a significant decrease in the yield. It is worth noting that, for both the **2i** and **2n** derivatives, the Stetter reaction proceeds smoothly, giving ketoesters with very high yields. Moreover, salicylaldehyde-derived trifluoromethyl acrylates bearing 6,8-dibromo (**2r**) and 6,8-dichloro (**2s**) groups in the aryl ring were well tolerated, and the corresponding products were obtained in high yields with excellent enantioselectivities. Notably, a more sterically hindered 5,7,8-trimethyl analogue (**2t**) with an increased electron density in the phenyl ring led to the desired CF_3_-chromanone in a high yield and with high stereoselectivity. The absolute configuration of the acid form of **2j** was unambiguously confirmed to be (*S*) by single-crystal X-ray diffraction analysis, and the other products were assigned by analogy.

Next, we examined the impact of the size and length of perfluorinated alkyl chains on the reactivity of the chiral NHC catalyst (Table 3). We discovered that the increased steric effects associated with long-chain perfluoroalkyl groups (C_3_F_7_–C_8_F_17_, **2u**–**2x**) in these substrates hindered the reactivity of the chiral NHC catalyst. This lack of reactivity is likely due to steric hindrance between the substrate and the chiral NHC catalyst. Nevertheless, smaller achiral NHC variants proved to be effective in catalyzing annulations, resulting in racemic products. The synthesis of racemic heterocycles is often beneficial for testing or reaction screening purposes.

**Table 3:**
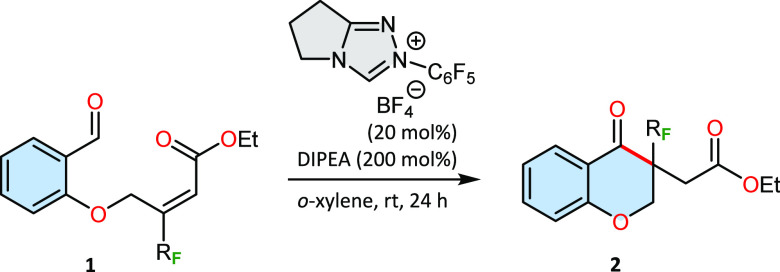

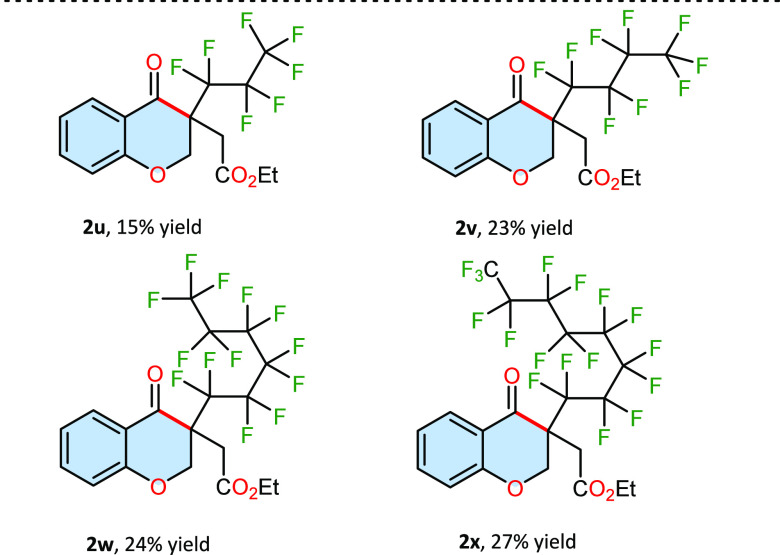
Scope of β-Perfluoroalkyl-β-Substituted Michael Acceptors^a^

a Unless otherwise specified, the reaction was performed on a 0.1 mmol scale in solvent (1.0 mL) at room temperature. Yields of isolated products. ee values determined by HPLC on a chiral stationary phase.

Surprisingly, during the preparation of β,β-perfluoroalkylated Michael acceptors, we observed the formation of adducts **3** (R_F_ = C_3_F_7_–C_8_F_17_) as side products. We then found that these highly substituted and sterically crowded Michael acceptors underwent an enantioselective intramolecular Stetter reaction to form a five-membered ring of the coumaranone system and two stereogenic centers with high diastereoselectivity and moderate enantioselectivity (Table 4, **4a**–**4d**). Moreover, the addition of the acyl anion equivalent occurred at the α-position of the Michael acceptor instead of at the β-carbon.

**Table 4:**
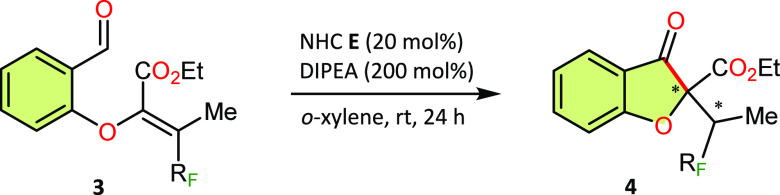

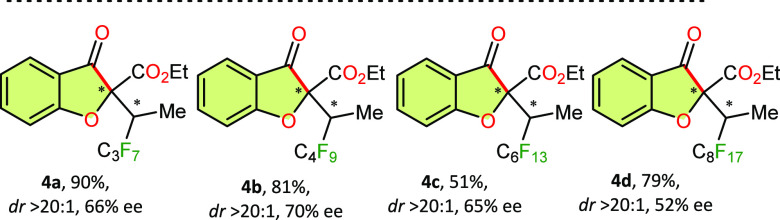
Enantio- and Diastereoselective Intramolecular Stetter Reaction of Tetrasubstituted Michael Acceptors^a^

a Unless otherwise specified, the reaction was performed on a 0.1 mmol scale in solvent (1.0 mL) at room temperature. Yields of isolated products. ee values determined by HPLC on a chiral stationary phase.

Presumably, two effects were involved. The first was due to the stabilization of the electron density and the fact that the large perfluoroalkyl group caused electrostatic repulsion. Second, the substrates can be regarded as the enol ethers of the corresponding ketones. Hence, the α-carbon atom is more electron-poor than the beta one. It is worth emphasizing that these are the first examples of tetrasubstituted Michael acceptors in NHC-mediated annulation reactions.

**Scheme 1:**
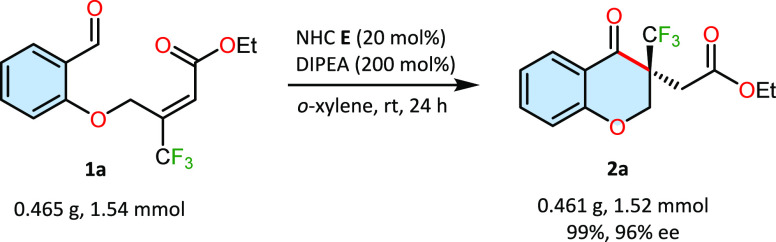
Scale-Up Reaction

To demonstrate the synthetic potential of the annulation process, a scale-up preparation of **2a** was performed (Scheme 1). The trifluoromethylated substrate **1a** (465 mg, 1.54 mmol) was reacted under standard conditions to give **2a** in a 99% yield (461 mg, 1.52 mmol). In comparison to the reaction on a 0.1 mmol scale, no decrease in the yield or stereoselectivity was observed.

Organic reactions conducted under solvent-free conditions are characterized by increased selectivity and efficiency along with the ease of manipulation all while circumventing the need for toxic and volatile solvents. Taking these advantages into account, especially in alignment with the principles of green chemistry, we opted to employ this approach for selected reactions (Table 5).^31^

**Table 5:**
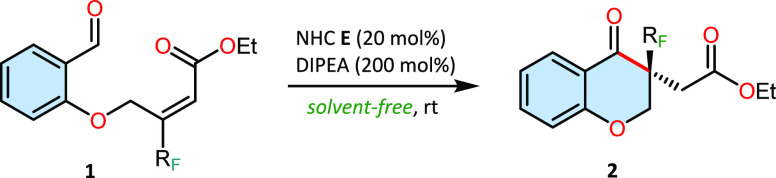

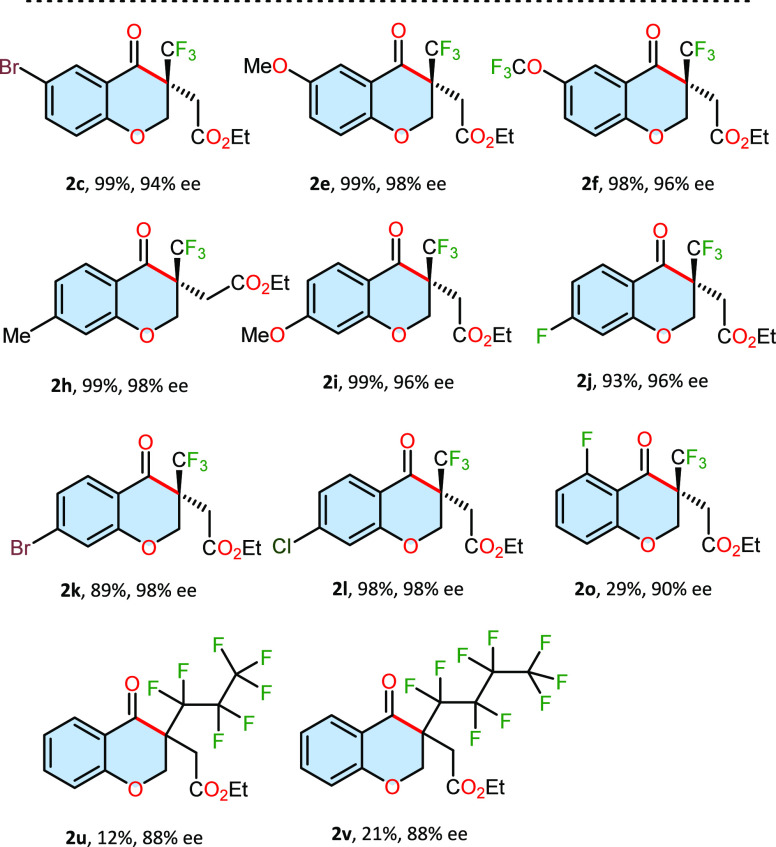
Enantioselective Synthesis of Fluorobenzopyranones under Solvent-Free Conditions^a^

a Unless otherwise specified, the reaction was performed on a 0.1 mmol scale without solvent at room temperature. Yields of isolated products. ee values determined by HPLC on a chiral stationary phase.

The reactions were found to proceed with remarkable smoothness over a time span of several hours, yielding the reaction products in near-quantitative amounts without a reduction in enantiomeric excess relative to the corresponding reactions conducted with solvent. Notably, the most significant differences in reactivity were observed for the higher homologues of the trifluoromethyl group. In the conventional approach employing both the solvent and a chiral catalyst, the reaction was unattainable. This pathway could be realized only by utilizing an achiral NHC catalyst. To our considerable satisfaction, under solvent-free conditions, the target reaction products were synthesized with elevated enantioselectivity.

## Conclusions

In conclusion, we have disclosed an NHC-catalyzed reaction of salicylaldehyde-derived trifluoromethyl acrylates for the enantioselective synthesis of benzopyranones bearing a quaternary C–CF_3_ stereogenic center. The developed procedure is the first original approach to the synthesis of chiral trifluorinated flavonoids containing C–CF_3_ bonds at the quaternary carbon atom. This core–structure-inspired strategy proceeded via the generation of chiral acyl anion equivalents and followed the intramolecular annulation reaction with high yields and enantioselectivities. Using tetrasubstituted Michael acceptors allowed us to obtain coumaranone derivatives in reasonable yields and selectivities. The enantioselective formation of asymmetric C–CF_3_ bonds through this methodology has opened up new avenues for the synthesis of biologically relevant and functionalized fluorinated molecules. Furthermore, the extension of this approach to other perfluoroalkyl homologues highlights the potential of this strategy in the broader context of organofluorine chemistry. The application of the procedure under solvent-free conditions proved to be an effective tool, yielding a series of target products with excellent efficiencies and enantioselectivities. The reactions took place at room temperature without the need for a ball mill, microwave irradiation, or ultrasonic irradiation.

Future research in this area could focus on the development of new chiral NHC catalysts with improved efficiency and selectivity as well as the exploration of alternative reaction pathways for the enantioselective synthesis of other fluorinated compounds. Additionally, the application of these newly synthesized chiral fluorinated compounds in various fields such as medicinal chemistry, agrochemistry, and materials chemistry will undoubtedly contribute to the further advancement of organofluorine chemistry and its numerous applications.

## Data Availability

The data underlying this study are available in the publishedarticle and its Supporting Information.

## Supporting Information Available

Experimental procedures and ^1^H and ^13^C NMR spectra of all the products (PDF)
