# Supplementary material for: Enantioselective Synthesis of Highly Substituted Fluoroalkylated Benzopyranones and 3-Coumaranones via N-Heterocyclic Carbene-Catalyzed Intramolecular Annulations
Source: J Org Chem. 2023 Oct 4;88(20):14339–44. doi: 10.1021/acs.joc.3c01099 (PMC10594660; doi:10.1021/acs.joc.3c01099)
Supplement: Supplementary file 1 — jo3c01099_si_001.pdf [file jo3c01099_si_001.pdf]

Supporting Information

Enantioselective, Fluoroalkylated Chromanone-Forming Annulations via NHC-Catalyzed Intramolecular Stetter Reactions

Izabela Barańska, Katarzyna Rafińska, Zbigniew Rafiński*

Faculty of Chemistry, Nicolaus Copernicus University in Torun, 7 Gagarin Street,
87-100 Torun, Poland.
*Corespondence: payudo@umk.pl

List of contents

1. General Methods S2
2. Synthetic Procedures S3
3. X-Ray Crystallography Data S41
4. NMR Spectra S44
5. HPLC Chromatograms S111
6. Literature S137

1. General Methods

Presented reactions were carried out in dry glassware under an inert atmosphere of argon. Selected reactions were monitored using thin-layer chromatography (TLC) and visualized under a UV lamp (254 nm). Anhydrous solvents were prepared using an INERT PureSolv Solvent Purification System. Purification of selected products was performed by column chromatography using a CombiFlash Rf + Lumen system with UV-vis and ELSD detectors. NMR spectra were recorded on a Bruker AMX 400 [400 MHz (^{1}H)] spectrometer and Bruker AMX 700 [700 MHz (^{1}H)] spectrometer, using $CDCl_3$ as a solvent and were reported in ppm relative to $CHCl_3$ residual peak (δ 7.24) for ^{1}H NMR and relative to the central $CDCl_3$ (δ 77.23) resonance for ^{13}C NMR. Coupling constants (*J*) were provided in Hz. The description does not include signals from perfluoroalkylated carbon atoms in the ^{13}C spectrum due to the multiplicity and very low intensity. Infrared spectra were measured on an Alpha FT-IR spectrometer from Bruker with an ATR module. Mass spectra were recorded on an Agilent 6530 Q-TOF LC/MS system coupled with a 1290 Infinity II liquid chromatograph. Melting points of obtained products were measured on Stuart SMP30 Melting Point Apparatus and Stuart SMP50 Melting Point Apparatus. The enantiomeric excess of chiral products was determined using HPLC Agilent Technologies 1200 Series and chiral stationary phases: Phenomenex Lux Cellulose-1 (3 μm) and Phenomenex Lux Amylose-1 (3 μm). The diffraction data of the studied compound were collected at T = 100 (2) K for the single crystal using Agilent Technologies SuperNova Dual Source with the CuKα radiation (λ = 1.54184 Å). The specific rotation of chiral products was determined using a polarimeter PolAAr 30-3000 from Optical Activity LTD.

2. Synthetic Procedures

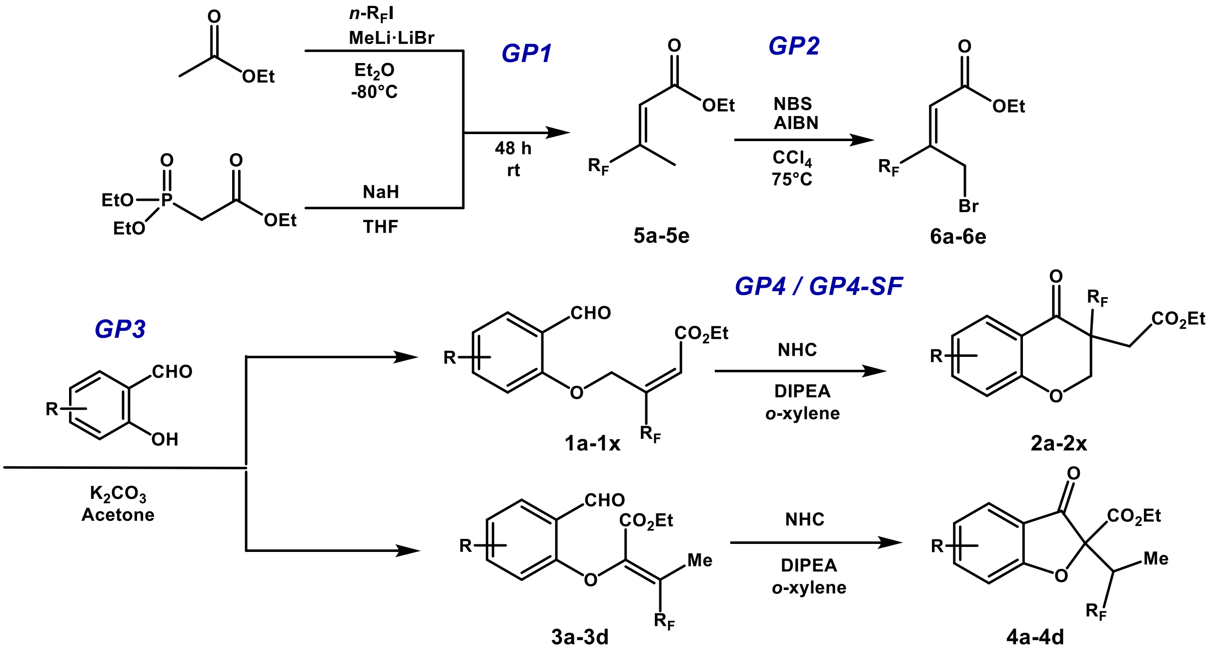

GP1. General Procedure 1: To the solution of perfluoroalkyl iodide (1 eq.), ethyl acetate (1.1 eq.) in Et_2O (0.4 M) was added at -95°C methyllithium (1.5 M in Et_2O with lithium bromide) (1 eq.), and stirring was continued for 1.5 h at the same temperature (the temperature was controlled with an immersion cooler). In a separate flask to the sodium hydride (1.2 eq.) in THF (1.4 M) was slowly introduced at 0°C (ice bath) triethyl phosphonoacetate (1.2 eq.), and the solution was stirred for 10 min at room temperature. This mixture was slowly transferred to the ketone solution at -80°C and then stirred for 48 h at room temperature. Then water, diethyl ether, and brine were added. The layers were separated, and the aqueous phase was extracted two times with diethyl ether. The combined organic extracts were dried over anhydrous magnesium sulfate and then concentrated under reduced pressure. The crude product was purified by flash chromatography.

GP2. General Procedure 2: To the solution of **5** (1 eq.) in CCl_4 (0.6 M) at room temperature was added NBS (1.16 eq.) and AIBN (0.037 eq.). The mixture was heated at 75°C (oil bath) for 24 h and then cooled to room temperature and diluted with CCl_4. The solid was filtered off and washed with CCl_4. The solvent was evaporated from the solution. The purification technique was adapted individually.

GP3. General Procedure 3: To the solution of salicylaldehyde (1 eq.) in acetone (1 M) was added potassium carbonate (1 eq.), and the mixture was stirred for 10 minutes at room temperature. Bromide **6** (1.06 eq.) was added, and stirring was continued for 24 h at the same temperature. The mixture was then diluted with acetone, and the precipitate was filtered off and

washed with acetone. After evaporation of the solvent, the crude product was purified by flash chromatography.

GP4. General Procedure 4: Enantioselective Intramolecular Stetter Reaction: A round bottom flask was charged with triazolium salt **E** (0.2 eq.) and *o*-xylene (0.1 M). Then *N*,*N*-diisopropylethylamine (2 eq.) was added, and the solution was allowed to stir at ambient temperature for 10 minutes. The substrate (**1** or **3**) (1 eq.) was added, and stirring was continued at the same temperature. The progress of the reaction was monitored by TLC. *o*-Xylene was evaporated, and the residue was dissolved in diethyl ether and petroleum ether. After filtration and evaporation of solvents, the product was obtained.

Note: All racemic products (**2a-2x**; **4a-4d**) were obtained according to the **General Procedure 4** using triazolium salt **F** as a catalyst.

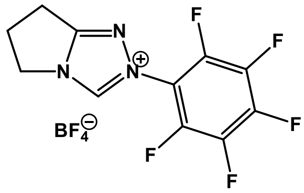

F

GP4-SF. General Procedure 4-SF: General Procedure for Solvent-Free Enantioselective Intramolecular Stetter Reaction : A round bottom flask was charged with triazolium salt (0.2 eq.) and substrate **1** or **3** (1 eq.). Then *N*,*N*-diisopropylethylamine (2 eq.) was added, and the solution was allowed to stir at ambient temperature. The progress of the reaction was monitored by TLC. After completion of the reaction, the mixture was evaporated to dryness. The residue was dissolved in diethyl ether and petroleum ether. After filtration and evaporation of solvents, the product was obtained.

GP5. General Procedure 5. Procedure for synthesis and sample preparation for XRD analysis :

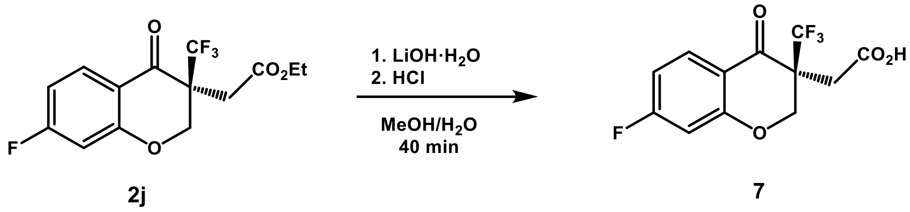

The compound **2j** (64 mg; 0.2 mmol) was dissolved in MeOH (3.68 mL) and water (1.05 mL) following the addition of LiOH·H_2O (41.96 mg; 1 mmol). The reaction was carried out at room temperature for 40 minutes. After this time the 1M HCl was added to pH=2. The organic phase was separated and the water phase was extracted with Et_2O(2x). The organic extract was dried with $MgSO_4$. After evaporation of solvents, the crude product was purified by flash chromatography to give 30 mg of pure compound **7** as a yellow oil(52%). The final product was further crystallized from EtOAc/pentane for XRD analysis.

Ethyl (E)-4,4,4-trifluoro-3-methylbut-2-enoate (**5a**)

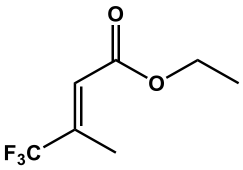

To the solution of ethyl (triphenylphosphoranylidene)acetate (197 mmol; 68.51 g) in 167 mL dichloromethane at 0° (ice bath) was slowly introduced trifluoroacetone (179 mmol; 16 mL) via syringe, and the solution was allowed to stir at ambient temperature for 94 h. Then pentane and diethyl ether was added to the flask. The precipitated solid was filtered off and washed with pentane. The solvent was evaporated, and the crude product was distilled at normal pressure. The expected product was obtained as a colorless liquid (22.51 g) with 69% yield.

bp: 128 - 131 °C.
^{1}H NMR (700 MHz, $CDCl_3$) δ 6.31 (quin, *J* = 1.5 Hz, 1H), 4.23 (q, *J* = 7.2 Hz, 2H), 2.24 (d, *J* = 1.7 Hz, 3H), 1.31 (t, *J* = 7.3 Hz, 3H).
^{13}C{^{1}H} NMR (101 MHz, $CDCl_3$) δ 164.6, 141.7 (q, *J* = 30.2 Hz), 123.1 (q, *J* = 273.9 Hz), 121.4 (q, *J* = 5.8 Hz), 60.6, 13.7, 11.9.
IR-ATR V_{max}: 1729, 1373, 1358, 1295, 1262, 1199, 1178, 1127, 1096, 1035, 1003, 894, 636 cm^{-1}.
HRMS (ESI-TOF) m/z: $(M + H)^+$ calcd for $C_7H_{10}F_3O_2$ 183.0633; found: 183.0636.
The above analysis results correspond to the literature data.[1]

Ethyl (E)-4,4,5,5,6,6,6-heptafluoro-3-methylhex-2-enoate (**5b**)

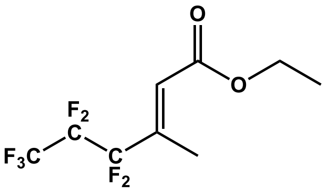

Perfluoropropyl iodide (34 mmol; 10 g), methyllithium (1.5 M in Et_2O with lithium bromide) (34 mmol; 22.70 mL), ethyl acetate (37 mmol; 3.63 mL), triethyl phosphonoacetate (41 mmol; 8 mL), sodium hydride (41 mmol; 1.66 g), diethyl ether (78 mL) and THF (24 mL) were used in the reaction carried out correspondingly to the **General Procedure 1**. The expected product was obtained as a colorless liquid (2.87 g) with 30% yield.

^{1}H NMR (700 MHz, $CDCl_3$) δ 6.29 (dd, *J* = 3.0, 1.3 Hz, 1H), 4.24 (q, *J* = 7.2 Hz, 2H), 2.28 - 2.26 (m, 3H), 1.32 (t, *J* = 7.1 Hz, 3H).
^{13}C{^{1}H} NMR (101 MHz, $CDCl_3$) δ 164.5, 142.1 (t, *J* = 21.5 Hz), 124.7 (t, *J* = 8.7 Hz), 60.9, 13.9, 13.1.
IR-ATR V_{max}: 1731, 1342, 1177, 1115, 1038, 862 cm^{-1}.
HRMS (ESI-TOF) m/z: $(M + H)^+$ calcd for $C_9H_{10}F_7O_2$ 283.0569; found: 283.0565.

Ethyl (E)-4,4,5,5,6,6,7,7,7-nonafluoro-3-methylhept-2-enoate (**5c**)

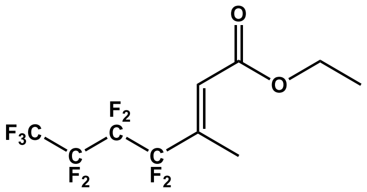

Perfluorobutyl iodide (22 mmol; 3.75 mL), methyllithium (1.5 M in Et_2O with lithium bromide) (22 mmol; 14.5 mL), ethyl acetate (24 mmol; 2.34 mL), triethyl phosphonoacetate (26 mmol; 5.20 mL), sodium hydride (26 mmol; 1.10 g), diethyl ether (50 mL) and THF (15 mL) were used in the reaction carried out correspondingly to the **General Procedure 1**. The expected product was obtained as a colorless liquid (5.94 g) with 81% yield.

^{1}H NMR (700 MHz, $CDCl_3$) δ 6.30 - 6.29 (m, 1H), 4.24 (q, *J* = 6.8 Hz, 2H), 2.27 (d, *J* = 1.7 Hz, 3H), 1.32 (t, *J* = 7.1 Hz, 3H).
^{13}C{^{1}H} NMR (176 MHz, $CDCl_3$) δ 164.1, 141.8 (t, *J* = 21.3 Hz), 124.4 (t, *J* = 9.0 Hz), 60.5, 13.5, 12.7.
IR-ATR V_{max}: 1731, 1231, 1187, 1132, 1106, 1039, 865, 823, 741 cm^{-1}.
HRMS (ESI-TOF) m/z: $(M + H)^+$ calcd for $C_{10}H_{10}F_9O_2$ 333.0537; found: 333.0535.

Ethyl (E)-4,4,5,5,6,6,7,7,8,8,9,9,9-tridecafluoro-3-methylnon-2-enoate (**5d**)

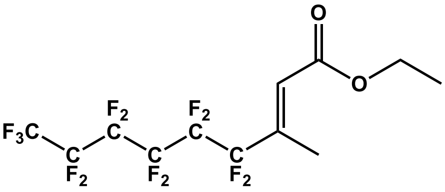

Perfluorohexyl iodide (22 mmol; 4.85 mL), methyllithium (1.5 M in Et_2O with lithium bromide) (22 mmol; 15.0 mL), ethyl acetate (25 mmol; 2.41 mL), triethyl phosphonoacetate (27 mmol; 5.34 mL), sodium hydride (27 mmol; 1.10 g), diethyl ether (52 mL) and THF (16 mL) were used in the reaction carried out correspondingly to the **General Procedure 1**. The expected product was obtained as a colorless liquid (4.00 g) with 42% yield.

^{1}H NMR (400 MHz, $CDCl_3$) δ 6.33 - 6.32 (m, 1H), 4.26 (q, *J* = 7.1 Hz, 2H), 2.29 (d, *J* = 1.2 Hz, 3H), 1.34 (t, *J* = 7.1 Hz, 3H).
^{13}C{^{1}H} NMR (101 MHz, $CDCl_3$) δ 164.5, 142.3 (t, *J* = 21.1 Hz), 124.8 (t, *J* = 9.1 Hz), 60.9, 13.9, 13.15 - 13.17 (m).
IR-ATR V_{max}: 1732, 1234, 1190, 1144, 1121, 1038, 721, 646 cm^{-1}.
HRMS (ESI-TOF) m/z: $(M + H)^+$ calcd for $C_{12}H_{10}F_{13}O_2$ 433.0473; found: 433.0477.
The above analysis results correspond to the literature data.[2]

Ethyl (E)-4,4,5,5,6,6,7,7,8,8,9,9,10,10,11,11,11-heptadecafluoro-3-methylundec-2-enoate (**5e**)

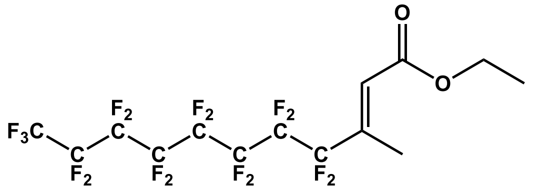

Heptadecafluoro-1-iodooctane (18 mmol; 4.84 mL), methyllithium (1.5 M in Et_2O with lithium bromide) (18 mmol; 12.3 mL), ethyl acetate (20 mmol; 1.97 mL), triethyl phosphonoacetate (22 mmol; 4.36 mL), sodium hydride (22 mmol; 0.90 g), diethyl ether (62.5 mL) and THF (13 mL) were used in the reaction carried out correspondingly to the **General Procedure 1**. Heptadecafluoro-1-iodooctane was introduced at -80°C. After introducing a methyllithium, stirring was continued for 1.5 h at -70°C. The expected product was obtained as a colorless liquid (4.61 g) with 48% yield.

^{1}H NMR (400 MHz, $CDCl_3$) δ 6.33 - 6.32 (m, 1H), 4.26 (q, *J* = 7.1 Hz, 2H), 2.30 - 2.29 (m, 3H), 1.34 (t, *J* = 7.1 Hz, 3H).
^{13}C{^{1}H} NMR (101 MHz, $CDCl_3$) δ 164.4, 142.3 (t, *J* = 21.1 Hz), 124.7 (t, *J* = 9.1 Hz), 60.7, 13.7, 12.86 - 12.88 (m).
IR-ATR V_{max}: 1731, 1193, 1145, 1039, 705, 657 cm^{-1}.
HRMS (ESI-TOF) m/z: $(M + H)^+$ calcd for $C_{14}H_{10}F_{17}O_2$ 533.0409; found: 533.0407.

Ethyl (Z)-3-(bromomethyl)-4,4,4-trifluorobut-2-enoate (**6a**)

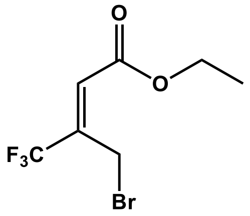

Ethyl (*E*)-4,4,4-trifluoro-3-methylbut-2-enoate (**5a**) (139 mmol; 25.35 g), NBS (161 mmol; 28.68 g), AIBN (5 mmol; 0.83 g) and CCl_4 (247 mL) were used in the reaction carried out correspondingly to the **General Procedure 2**. The crude product was distilled under reduced pressure. The expected product was obtained as a colorless liquid (27.73 g) with 76% yield.

bp: 70 - 75°/ 10 mmHg.
^{1}H NMR (400 MHz, $CDCl_3$) δ 6.46 - 6.45 (m, 1H), 4.53 (s, 2H), 4.30 (qd, *J* = 7.1, 0.6 Hz, 2H), 1.35 (td, *J* = 7.2, 0.8 Hz, 3H).
^{13}C{^{1}H} NMR (101 MHz, $CDCl_3$) δ 163.5, 141.2 (q, *J* = 30.5 Hz), 125.5 (q, *J* = 4.8 Hz), 122.5 (q, *J* = 275.5 Hz), 61.7, 18.8, 13.9.
IR-ATR V_{max}: 1726, 1376, 1359, 1301, 1264, 1207, 1187, 1132, 1020, 902, 709, 626, 587 cm^{-1}.
HRMS (ESI-TOF) m/z: $(M + H)^+$ calcd for $C_7H_9BrF_3O_2$ 260.9738; found: 260.9744.
The above analysis results correspond to the literature data.[3]

Ethyl (Z)-3-(bromomethyl)-4,4,5,5,6,6,6-heptafluorohex-2-enoate (**6b**)

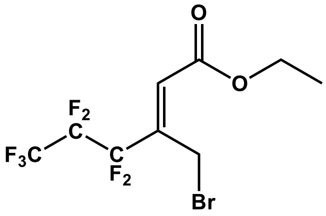

Ethyl (*E*)-4,4,5,5,6,6,6-heptafluoro-3-methylhex-2-enoate (**5b**) (7 mmol; 2.04 g), NBS (8 mmol; 1.49 g), AIBN (0.27 mmol; 44 mg) and CCl_4 (13 mL) were used in the reaction carried out correspondingly to the **General Procedure 2**. The crude product was purified by flash chromatography. The expected product was obtained as a yellow liquid (1.98 g) with 78% yield.

^{1}H NMR (700 MHz, $CDCl_3$) δ 6.40 (s, 1H), 4.51 (s, 2H), 4.31 (q, *J* = 7.0 Hz, 2H), 1.35 (t, *J* = 7.1 Hz, 3H).
^{13}C{^{1}H} NMR (101 MHz, $CDCl_3$) δ 163.1, 141.3 (t, *J* = 22.3 Hz), 128.9 (t, *J* = 7.9 Hz), 61.7, 19.5, 13.9.
IR-ATR V_{max}: 1729, 1375, 1343, 1181, 1115, 1025, 877, 749, 718 cm^{-1}.
HRMS (ESI-TOF) m/z: $(M + H)^+$ calcd for $C_9H_9BrF_7O_2$ 360.9674; found: 360.9678.

Ethyl (Z)-3-(bromomethyl)-4,4,5,5,6,6,7,7,7-nonafluorohept-2-enoate (**6c**)

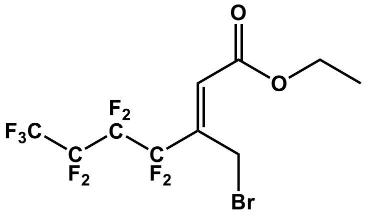

Ethyl (*E*)-4,4,5,5,6,6,7,7,7-nonafluoro-3-methylhept-2-enoate (**5c**) (11 mmol; 3.69 g), NBS (13 mmol; 2.29 g), AIBN (0.41 mmol; 67 mg) and CCl_4 (20 mL) were used in the reaction carried out correspondingly to the **General Procedure 2**. The crude product was purified by flash chromatography. The expected product was obtained as a yellow liquid (3.26 g) with 72% yield.

^{1}H NMR (400 MHz, $CDCl_3$) δ 6.43 (s, 1H), 4.54 (s, 2H), 4.33 (q, *J* = 7.1 Hz, 2H), 1.37 (t, *J* = 7.1 Hz, 3H).
^{13}C{^{1}H} NMR (101 MHz, $CDCl_3$) δ 163.1, 141.5 (t, *J* = 22.3 Hz), 129.1 (t, *J* = 7.9 Hz), 61.7, 19.5, 13.9.
IR- ATR V_{max}: 1729, 1375, 1348, 1228, 1189, 1132, 1080, 1035, 868, 831, 741 cm^{-1}.
HRMS (ESI-TOF) m/z: $(M + H)^+$ calcd for $C_{10}H_9BrF_9O_2$ 410.9642; found: 410.9647.

Ethyl (Z)-3-(bromomethyl)-4,4,5,5,6,6,7,7,8,8,9,9,9-tridecafluoronon-2-enoate (**6d**)

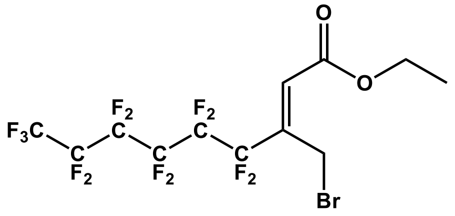

Ethyl (*E*)-4,4,5,5,6,6,7,7,8,8,9,9,9-tridecafluoro-3-methylnon-2-enoate (**5d**) (6 mmol; 2.63 g), NBS (7 mmol; 1.25 g), AIBN (0.22 mmol; 36 mg) and CCl_4 (11 mL) were used in the reaction carried out correspondingly to the **General Procedure 2**. The crude product was purified by flash chromatography. The expected product was obtained as a yellow liquid (2.08 g) with 68% yield.

^{1}H NMR (400 MHz, $CDCl_3$) δ 6.43 (s, 1H), 4.54 (s, 2H), 4.33 (q, *J* = 7.1 Hz, 2H), 1.38 (t, *J* = 7.2 Hz, 3H).
^{13}C{^{1}H} NMR (101 MHz, $CDCl_3$) δ 163.1, 141.6 (t, *J* = 22.3 Hz), 129.1 (t, *J* = 7.9 Hz), 61.7, 19.5, 13.8.
IR-ATR V_{max}: 1730, 1353, 1231, 1191, 1143, 1081, 1019, 808, 777, 738 cm^{-1}.
HRMS (ESI-TOF) m/z: $(M + H)^+$ calcd for $C_{12}H_9BrF_{13}O_2$ 510.9578; found: 510.9581.

Ethyl (Z)-3-(bromomethyl)-4,4,5,5,6,6,7,7,8,8,9,9,10,10,11,11,11-heptadecafluoroundec-2-enoate (**6e**)

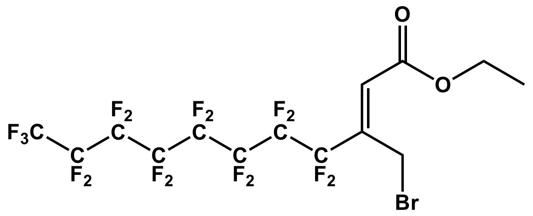

Ethyl (*E*)-4,4,5,5,6,6,7,7,8,8,9,9,10,10,11,11,11-heptadecafluoro-3-methylundec-2-enoate (**5e**) (7 mmol; 3.91 g), NBS (9 mmol; 1.52 g), AIBN (0.27 mmol; 45 mg) and CCl_4 (13 mL) were used in the reaction carried out correspondingly to the **General Procedure 2**. The crude product was purified by flash chromatography. The expected product was obtained as a colorless liquid (3.12 g) with 73% yield.

^{1}H NMR (700 MHz, $CDCl_3$) δ 6.41 (s, 1H), 4.52 (s, 2H), 4.31 (q, *J* = 7.2 Hz, 2H), 1.36 (t, *J* = 7.3 Hz, 3H).
^{13}C{^{1}H} NMR (101 MHz, $CDCl_3$) δ 163.0, 141.6 (t, *J* = 22.3 Hz), 129.0 (t, *J* = 7.9 Hz), 61.6, 19.3, 13.7.
IR-ATR V_{max}: 1731, 1373, 1194, 1145, 1024, 899, 720, 672, 656, 559, 528 cm^{-1}.
HRMS (ESI-TOF) m/z: $(M + H)^+$ calcd for $C_{14}H_9BrF_{17}O_2$ 610.9514; found: 610.9519.

Ethyl (E)-4,4,4-trifluoro-3-((2-formylphenoxy)methyl)but-2-enoate (**1a**)

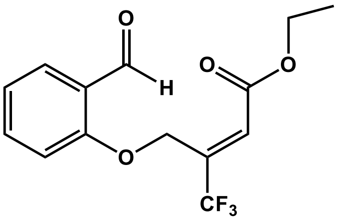

Ethyl (*Z*)-3-(bromomethyl)-4,4,4-trifluorobut-2-enoate (**6a**) ((18 mmol; 4.60 g), salicylaldehyde (18 mmol; 2.15 g), potassium carbonate (18 mmol; 2.43 g), and acetone (17 mL) were used in the reaction carried out correspondingly to the **General Procedure 3**. The expected product was obtained as a yellow liquid (3.67 g) with 67% yield.

^{1}**H NMR** (700 MHz, $CDCl_3$) δ 10.47 (d, *J* = 0.9 Hz, 1H), 7.89 (dd, *J* = 7.7, 1.7 Hz, 1H), 7.59 (ddd, *J* = 8.2, 7.3, 1.7 Hz, 1H), 7.13 - 7.10 (m, 2H), 6.66 (d, *J* = 1.3 Hz, 1H), 5.35 (d, *J* = 0.9 Hz, 2H), 4.30 (q, *J* = 6.9 Hz, 2H), 1.34 (t, *J* = 7.1 Hz, 3H).
^{13}**C{^{1}H} NMR** (101 MHz, $CDCl_3$) δ 189.5, 163.8, 160.3, 139.0 (q, *J* = 30.2 Hz), 135.9, 128.4, 126.8 (q, *J* = 5.6 Hz), 125.3, 122.4 (q, *J* = 275.5 Hz), 121.7, 112.6, 61.8, 61.1, 14.0.
IR-ATR V_{max}: 1726, 1688, 1599, 1484, 1458, 1390, 1305, 1284, 1178, 1132, 1020, 906, 846, 757, 646 cm^{-1}.
HRMS (ESI-TOF) m/z: $(M + H)^+$ calcd for $C_{14}H_{14}F_3O_4$ 303.0844; found: 303.0842.

Ethyl (E)-4,4,4-trifluoro-3-((2-formyl-4-iodophenoxy)methyl)but-2-enoate (**1b**)

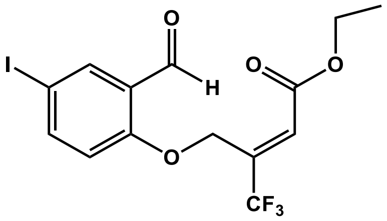

Ethyl (*Z*)-3-(bromomethyl)-4,4,4-trifluorobut-2-enoate (**6a**) (3.83 mmol; 1.0 g), 2-hydroxy-5-iodobenzaldehyde (3.6 mmol; 0.89 g), potassium carbonate (3.62 mmol; 0.5 g) and acetone (10 mL) were used in the reaction carried out correspondingly to the **General Procedure 3**. The expected product was obtained as a yellow liquid (0.23 g) with 14% yield.

^{1}**H NMR** (700 MHz, $CDCl_3$) δ 10.30 - 10.28 (m, 1H), 8.12 - 8.12 (m, 1H), 7.83 - 7.81 (m, 1H), 6.87 (d, *J* = 8.6 Hz, 1H), 6.62 (s, 1H), 5.30 (s, 2H), 4.26 (q, *J* = 7.1 Hz, 2H), 1.31 (t, *J* = 7.1 Hz, 3H).
^{13}**C{^{1}H} NMR** (176 MHz, $CDCl_3$) δ 187.4, 163.3, 159.4, 143.7, 138.2 (q, *J* = 30.5 Hz), 136.5, 126.7 (q, *J* = 5.4 Hz), 126.4, 121.9 (q, *J* = 275.2 Hz), 114.7, 83.8, 61.5, 60.8, 13.6.
IR-ATR $\mathbf{V_{max}}$: 1725, 1683, 1585, 1474, 1386, 1306, 1272, 1200, 1177, 1133, 1019, 902, 869, 809 cm^{-1}.
HRMS (ESI-TOF) m/z: $(M + H)^+$ calcd for $C_{14}H_{13}F_3IO_4$ 428.9811; found: 428.9809.

Ethyl (E)-3-((4-bromo-2-formylphenoxy)methyl)-4,4,4-trifluorobut-2-enoate (**1c**)

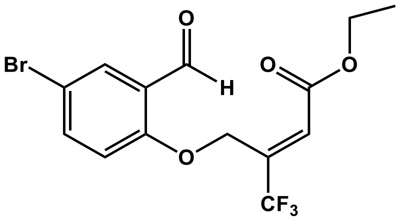

Ethyl (*Z*)-3-(bromomethyl)-4,4,4-trifluorobut-2-enoate (**6a**) (3.83 mmol; 1.0 g), 5-bromo-2-hydroxybenzaldehyde (3.63 mmol; 0.73 g), potassium carbonate (3.62 mmol; 0.50 g), and acetone (5 mL) were used in the reaction carried out correspondingly to the **General Procedure 3**. The expected product was obtained as a yellow oil (0.52 g) with 36% yield.

^{1}H NMR (400 MHz, $CDCl_3$) δ 10.35 (s, 1H), 7.95 (d, *J* = 2.6 Hz, 1H), 7.65 (dd, *J* = 8.8, 2.7 Hz, 1H), 7.01 (d, *J* = 8.9 Hz, 1H), 6.65 - 6.63 (m, 1H), 5.32 (d, *J* = 0.9 Hz, 2H), 4.28 (q, *J* = 7.1 Hz, 2H), 1.33 (t, *J* = 7.1 Hz, 3H).
^{13}C{^{1}H} NMR (101 MHz, $CDCl_3$) δ 188.0, 163.7, 159.1, 138.7 (q, *J* = 30.2 Hz), 138.2, 131.1, 127.1 (q, *J* = 5.6 Hz), 126.6, 122.3 (q, *J* = 275.5 Hz), 114.7, 114.6, 61.9, 61.4, 14.0.
IR-ATR V_{max}: 1725, 1684, 1590, 1476, 1388, 1306, 1270, 1241, 1200, 1177, 1134, 1087, 1019, 901, 882, 810 cm^{-1}.
HRMS (ESI-TOF) m/z: $(M + H)^+$ calcd for $C_{14}H_{13}BrF_3O_4$ 380.9949; found: 380.9953.

Ethyl (E)-4,4,4-trifluoro-3-((4-fluoro-2-formylphenoxy)methyl)but-2-enoate (**1d**)

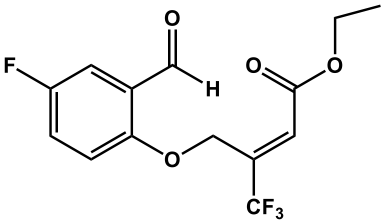

Ethyl (*Z*)-3-(bromomethyl)-4,4,4-trifluorobut-2-enoate (**6a**) (3.83 mmol; 1.0 g), 2-hydroxy-5-fluorobenzaldehyde (3.57 mmol; 0.50 g), potassium carbonate (3.62 mmol; 0.50 g), and acetone (5 mL) were used in the reaction carried out correspondingly to the **General Procedure 3**. The expected product was obtained as a yellow liquid (0.62 g) with 51% yield.

^{1}H NMR (700 MHz, $CDCl_3$) δ 10.36 (d, *J* = 3.4 Hz, 1H), 7.52 (dd, *J* = 8.2, 3.0 Hz, 1H), 7.26 (ddd, *J* = 9.0, 7.7, 3.0 Hz, 1H), 7.06 (dd, *J* = 9.0, 3.9 Hz, 1H), 6.62 (d, *J* = 0.8 Hz, 1H), 5.29 (d, *J* = 0.9 Hz, 2H), 4.26 (q, *J* = 7.2 Hz, 2H), 1.31 (t, *J* = 7.1 Hz, 3H).
^{13}C{^{1}H} NMR (101 MHz, $CDCl_3$) δ 188.3, 163.8, 157.6 (d, *J* = 222.5 Hz), 156.4 (d, *J* = 22.3 Hz), 138.9 (q, *J* = 30.2 Hz), 127.0 (q, *J* = 11.9, 5.8 Hz), 126.3 (d, *J* = 5.6 Hz), 122.4 (q, *J* = 275.2 Hz), 122.3 (d, *J* = 24.1 Hz), 114.6 (d, *J* = 7.2 Hz), 114.2 (d, *J* = 23.8 Hz), 61.9, 61.8, 14.0.
IR-ATR V_{max}: 1726, 1686, 1489, 1428, 1393, 1307, 1264, 1180, 1133, 1087, 1021, 967, 890, 813, 741, 718 cm^{-1}.
HRMS (ESI-TOF) m/z: $(M + H)^+$ calcd for $C_{14}H_{13}F_4O_4$ 321.0750; found: 321.0748.

Ethyl (E)-4,4,4-trifluoro-3-((2-formyl-4-methoxyphenoxy)methyl)but-2-enoate (**1e**)

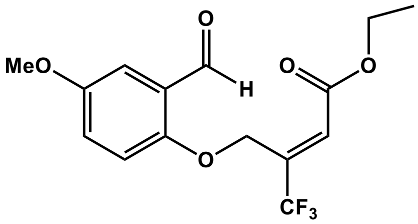

Ethyl (*Z*)-3-(bromomethyl)-4,4,4-trifluorobut-2-enoate (**6a**) (3.83 mmol; 1.0 g), 2-hydroxy-5-methoxybenzaldehyde (3.63 mmol; 0.45 mL), potassium carbonate (3.62 mmol; 0.50 g) and acetone (5 mL) were used in the reaction carried out correspondingly to the **General Procedure 3**. The expected product was obtained as a yellow liquid (0.51 g) with 40% yield.

^{1}H NMR (400 MHz, $CDCl_3$) δ 10.41 (s, 1H), 7.36 (d, *J* = 3.3 Hz, 1H), 7.15 (dd, *J* = 9.1, 3.3 Hz, 1H), 7.05 (d, *J* = 9.2 Hz, 1H), 6.63 (d, *J* = 1.3 Hz, 1H), 5.27 (d, *J* = 0.7 Hz, 2H), 4.28 (q, *J* = 7.1 Hz, 2H), 3.83 (s, 3H), 1.33 (t, *J* = 7.2 Hz, 3H).
^{13}C{^{1}H} NMR (101 MHz, $CDCl_3$) δ 189.3, 163.8, 155.0, 154.4, 139.1 (q, *J* = 30.2 Hz), 126.8 (q, *J* = 5.6 Hz), 125.8, 123.2, 122.4 (q, *J* = 275.5 Hz), 114.8, 110.4, 61.9, 61.8, 55.8, 14.0.
IR-ATR V_{max}: 1726, 1685, 1493, 1466, 1424, 1392, 1306, 1277, 1180, 1160, 1132, 1087, 1032, 813, 730 cm^{-1}.
HRMS (ESI-TOF) m/z: $(M + H)^+$ calcd for $C_{15}H_{16}F_3O_5$ 333.0950; found: 333.0947.

Ethyl (E)-4,4,4-trifluoro-3-((2-formyl-4-(trifluoromethoxy)phenoxy)methyl)but-2-enoate (**1f**)

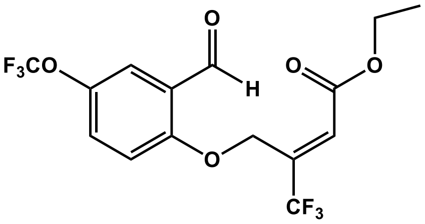

Ethyl (*Z*)-3-(bromomethyl)-4,4,4-trifluorobut-2-enoate (**6a**) (3.45 mmol; 0.9 g), 2-hydroxy-5-(trifluoromethoxy)benzaldehyde (3.52 mmol; 0.67 g), potassium carbonate (4.85 mmol; 0.67 g), and acetone (10 mL) were used in the reaction carried out correspondingly to the **General Procedure 3**. The expected product was obtained as a yellow liquid (0.64 g) with 47% yield.

^{1}H NMR (700 MHz, $CDCl_3$) δ 10.38 (s, 1H), 7.71 - 7.70 (m, 1H), 7.41 (ddd, *J* = 9.0, 3.0, 0.9 Hz, 1H), 7.12 (d, *J* = 9.0 Hz, 1H), 6.64 (d, *J* = 0.9 Hz, 1H), 5.34 (d, *J* = 0.8 Hz, 2H), 4.27 (q, *J* = 7.2 Hz, 2H), 1.32 (t, *J* = 7.3 Hz, 3H).
^{13}C{^{1}H} NMR (101 MHz, $CDCl_3$) δ 188.0, 163.7, 158.5, 143.48 - 143.52 (m), 138.7 (q, *J* = 30.2 Hz), 128.4, 127.1 (q, *J* = 5.6 Hz), 126.0, 122.3 (q, *J* = 275.2 Hz), 120.8, 120.4 (q, *J* = 257.5 Hz), 114.1, 61.9, 61.5, 14.0.
IR-ATR V_{max}: 1728, 1690, 1491, 1429, 1391, 1356, 1309, 1250, 1134, 1020, 907, 821, 772, 723, 642 cm^{-1}.
HRMS (ESI-TOF) m/z: $(M + H)^+$ calcd for $C_{15}H_{13}F_6O_5$ 387.0667; found: 387.0664.

Ethyl (E)-4,4,4-trifluoro-3-((2-formyl-4-nitrophenoxy)methyl)but-2-enoate (**1g**)

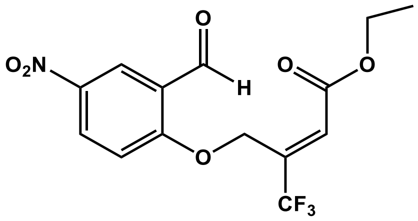

Ethyl (*Z*)-3-(bromomethyl)-4,4,4-trifluorobut-2-enoate (**6a**) (3.83 mmol; 1.0 g), 2-hydroxy-5-nitrobenzaldehyde (3.63 mmol; 0.61 g), potassium carbonate (4.01 mmol; 0.55 g), and acetone (10 mL) were used in the reaction carried out correspondingly to the **General Procedure 3**. The expected product was obtained as a yellow oil (0.10 g) with 8% yield.

^{1}H NMR (400 MHz, $CDCl_3$) δ 10.38 (s, 1H), 8.66 (d, *J* = 2.9 Hz, 1H), 7.27 (d, *J* = 9.2 Hz, 1H), 6.68 (d, *J* = 1.3 Hz, 1H), 5.49 (d, *J* = 0.9 Hz, 2H), 4.28 (q, *J* = 7.1 Hz, 2H), 1.32 (t, *J* = 7.1 Hz, 3H).
^{13}C{^{1}H} NMR (101 MHz, $CDCl_3$) δ 187.1, 163.8, 163.6, 142.2, 137.9 (q, *J* = 30.5 Hz), 130.6, 127.7 (q, *J* = 5.6 Hz), 125.0, 124.4, 122.2 (q, *J* = 275.5 Hz), 113.2, 62.1, 61.8, 13.9.
IR-ATR V_{max}: 1725, 1692, 1610, 1590, 1525, 1484, 1345, 1306, 1271, 1203, 1176, 1132, 1077, 1001, 942, 909, 818, 748 cm^{-1}.
HRMS (ESI-TOF) m/z: $(M + H)^+$ calcd for $C_{14}H_{13}F_3NO_6$ 348.0695; found: 348.0696.

Ethyl (E)-4,4,4-trifluoro-3-((2-formyl-5-methylphenoxy)methyl)but-2-enoate (**1h**)

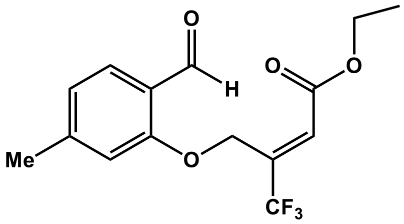

Ethyl (*Z*)-3-(bromomethyl)-4,4,4-trifluorobut-2-enoate (**6a**) (3.83 mmol; 1.0 g), 2-hydroxy-4-methylbenzaldehyde (3.63 mmol; 0.49 g), potassium carbonate (3.62 mmol; 0.50 g), and acetone (5 mL) were used in the reaction carried out correspondingly to the **General Procedure 3**. The expected product was obtained as a yellow liquid (0.44 g) with 36% yield.

^{1}H NMR (700 MHz, $CDCl_3$) δ 10.39 (d, *J* = 0.9 Hz, 1H), 7.78 (d, *J* = 7.7 Hz, 1H), 6.92 (dd, *J* = 7.5, 1.1 Hz, 1H), 6.89 (s, 1H), 6.66 (d, *J* = 0.9 Hz, 1H), 5.32 (d, *J* = 0.9 Hz, 2H), 4.30 (q, *J* = 6.9 Hz, 2H), 2.44 (s, 3H), 1.35 (t, *J* = 7.1 Hz, 3H).
^{13}C{^{1}H} NMR (101 MHz, $CDCl_3$) δ 190.1, 164.9, 161.4, 148.4, 140.1 (q, *J* = 30.5 Hz), 129.3, 127.7 (q, *J* = 6.0 Hz), 124.0, 123.7, 123.4 (q, *J* = 275.2 Hz), 114.1, 62.8, 62.0, 23.3, 15.0.
IR-ATR V_{max}: 1727, 1684, 1606, 1392, 1303, 1257,1202, 1178, 1159, 1133, 1112, 1088, 1024, 811, 739 cm^{-1}.
HRMS (ESI-TOF) m/z: $(M + H)^+$ calcd for $C_{15}H_{16}F_3O_4$ 317.1001; found: 317.1003.

Ethyl (E)-4,4,4-trifluoro-3-((2-formyl-5-methoxyphenoxy)methyl)but-2-enoate (**1i**)

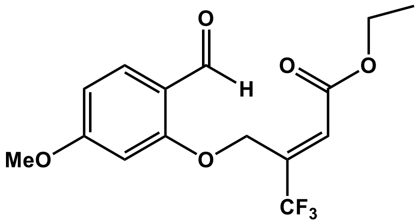

Ethyl (*Z*)-3-(bromomethyl)-4,4,4-trifluorobut-2-enoate (**6a**) (3.83 mmol; 1.0 g), 2-hydroxy-4-methoxybenzaldehyde (3.63 mmol; 0.55 g), potassium carbonate (3.62 mmol; 0.50 g), and acetone (5 mL) were used in the reaction carried out correspondingly to the **General Procedure 3**. The expected product was obtained as a yellow liquid (0.43 g) with 36% yield.

^{1}H NMR (700 MHz, $CDCl_3$) δ 10.29 (d, *J* = 0.9 Hz, 1H), 7.87 (d, *J* = 8.6 Hz, 1H), 6.65 (d, *J* = 0.9 Hz, 1H), 6.62 (dd, *J* = 8.6, 2.2 Hz, 1H), 6.58 (d, *J* = 2.2 Hz, 1H), 5.32 (s, 2H), 4.30 (q, *J* = 7.3 Hz, 2H), 3.90 (s, 3H), 1.34 (t, *J* = 7.1 Hz, 3H).
^{13}C{^{1}H} NMR (101 MHz, $CDCl_3$) δ 188.0, 166.1, 163.8, 162.0, 139.0 (q, *J* = 30.2 Hz), 130.3, 126.8 (q, *J* = 5.6 Hz), 122.4 (d, *J* = 275.2 Hz), 119.3, 107.0, 98.7, 61.8, 60.9, 55.7, 14.0.
IR-ATR V_{max}: 1726, 1678, 1599, 1579, 1503, 1465, 1444, 1396, 1305, 1257, 1193, 1166, 1134, 1112, 1087, 1021, 906, 826, 814 cm^{-1}.
HRMS (ESI-TOF) m/z: $(M + H)^+$ calcd for $C_{15}H_{16}F_3O_5$ 333.0950; found: 333.0953.

Ethyl (E)-4,4,4-trifluoro-3-((5-fluoro-2-formylphenoxy)methyl)but-2-enoate (**1j**)

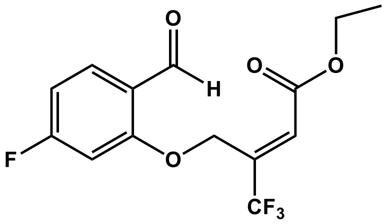

Ethyl (*Z*)-3-(bromomethyl)-4,4,4-trifluorobut-2-enoate (**6a**) (3.83 mmol; 1.0 g), 2-hydroxy-4-fluorobenzaldehyde (3.63 mmol; 0.51 g), potassium carbonate (3.62 mmol; 0.5 g), and acetone (5 mL) were used in the reaction carried out correspondingly to the **General Procedure 3**. The expected product was obtained as a yellow liquid (0.38 g) with 31% yield.

^{1}H NMR (400 MHz, $CDCl_3$) δ 10.33 (s, 1H), 7.93 - 7.87 (m, 1H), 6.83 - 6.76 (m, 2H), 6.66 (d, *J* = 1.1 Hz, 1H), 5.32 (d, *J* = 0.9 Hz, 2H), 4.29 (q, *J* = 7.2 Hz, 2H), 1.34 (t, *J* = 7.2 Hz, 3H).
^{13}C{^{1}H} NMR (101 MHz, $CDCl_3$) δ 187.8, 167.5 (d, *J* = 256.7 Hz), 163.7, 161.8 (d, *J* = 11.1 Hz), 138.5 (q, *J* = 30.5 Hz), 130.7 (d, *J* = 11.9 Hz), 127.2 (q, *J* = 5.6 Hz), 122.3 (q, *J* = 275.2 Hz), 122.0 (d, *J* = 3.2 Hz), 109.1 (d, *J* = 22.3 Hz), 100.7 (d, *J* = 26.2 Hz), 61.9, 61.4, 13.9.
IR-ATR V_{max}: 1726, 1688, 1603, 1594, 1497, 1433, 1392, 1307, 1276, 1253, 1209, 1182, 1134, 1088, 1020, 838, 813, 649, 477 cm^{-1}.
HRMS (ESI-TOF) m/z: $(M + H)^+$ calcd for $C_{14}H_{13}F_4O_4$ 321.0750; found: 321.0749.

Ethyl (E)-3-((5-bromo-2-formylphenoxy)methyl)-4,4,4-trifluorobut-2-enoate (**1k**)

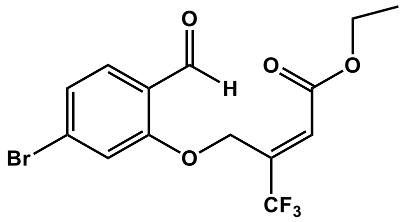

Ethyl (*Z*)-3-(bromomethyl)-4,4,4-trifluorobut-2-enoate (**6a**) (3.8 mmol; 1.0 g), 4-bromo-2-hydroxybenzaldehyde (3.6 mmol; 0.72 g), potassium carbonate (3.6 mmol; 0.5 g) and acetone (10 mL) were used in the reaction carried out correspondingly to the **General Procedure 3**. The expected product was obtained as a yellow solid (0.28 g) with 20% yield.

^{1}H NMR (700 MHz, $CDCl_3$) δ 10.34 (s, 1H), 7.71 (d, *J* = 8.6 Hz, 1H), 7.25 (d, *J* = 1.3 Hz, 1H), 7.23 - 7.22 (m, 1H), 6.64 (s, 1H), 5.31 (s, 2H), 4.28 (q, *J* = 7.2 Hz, 2H), 1.32 (t, *J* = 7.1 Hz, 3H).
^{13}C{^{1}H} NMR (176 MHz, $CDCl_3$) δ 188.0, 163.3, 159.9, 138.1 (q, *J* = 30.5 Hz), 130.0, 129.2, 126.9 (q, *J* = 5.4 Hz), 124.8, 123.7, 121.9 (q, *J* = 275.7 Hz), 115.9, 61.6, 60.8, 13.6.
IR-ATR V_{max}: 1719, 1682, 1586, 1413, 1386, 1301, 1211, 1190, 1175, 1144, 1022, 1005, 900, 806 cm^{-1}.
HRMS (ESI-TOF) m/z: $(M + H)^+$ calcd for $C_{14}H_{13}BrF_3O_4$ 380.9949; found: 380.9955.

Ethyl (E)-3-((5-chloro-2-formylphenoxy)methyl)-4,4,4-trifluorobut-2-enoate (**1l**)

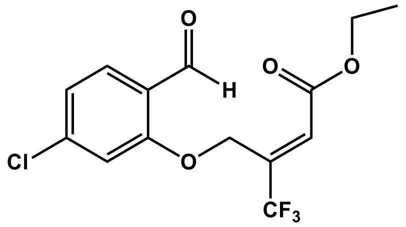

Ethyl (*Z*)-3-(bromomethyl)-4,4,4-trifluorobut-2-enoate (**6a**) (3.83 mmol; 1.0 g), 4-chloro-2-hydroxybenzaldehyde (3.63 mmol; 0.57 g), potassium carbonate (3.62 mmol; 0.5 g), and acetone (5 mL) were used in the reaction carried out correspondingly to the **General Procedure 3**. The expected product was obtained as a yellow solid (0.43 g) with 33% yield.

^{1}H NMR (400 MHz, $CDCl_3$) δ 10.36 (d, *J* = 0.7 Hz, 1H), 7.82 (d, *J* = 8.2 Hz, 1H), 7.12 - 7.06 (m, 2H), 6.66 (d, *J* = 1.2 Hz, 1H), 5.33 (d, *J* = 0.9 Hz, 2H), 4.30 (q, *J* = 7.1 Hz, 2H), 1.35 (t, *J* = 7.2 Hz, 3H).
^{13}C{^{1}H} NMR (101 MHz, $CDCl_3$) δ 188.2, 163.7, 160.5, 141.9, 138.5 (q, *J* = 30.5 Hz), 129.5, 127.2 (q, *J* = 5.6 Hz), 123.8, 122.3 (q, *J* = 275.2 Hz), 122.3, 113.3, 62.0, 61.3, 14.0.
IR-ATR V_{max}: 1718, 1683, 1589, 1418, 1386, 1356, 1297, 1245, 1211, 1192, 1175, 1108, 1085, 1022, 1006, 901, 634 cm^{-1}.
HRMS (ESI-TOF) m/z: $(M + H)^+$ calcd for $C_{14}H_{13}ClF_3O_4$ 337.0454; found: 337.0456.

Ethyl (E)-4,4,4-trifluoro-3-((2-fluoro-6-formylphenoxy)methyl)but-2-enoate (**1m**)

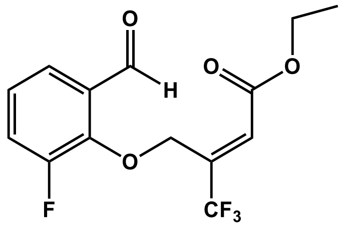

Ethyl (*Z*)-3-(bromomethyl)-4,4,4-trifluorobut-2-enoate (**6a**) (3.42 mmol; 0.9 g), 3-fluorosalicylaldehyde (3.24 mmol; 0.45 g), potassium carbonate (3.26 mmol; 0.45 g) and acetone (10 mL) were used in the reaction carried out correspondingly to the **General Procedure 3**. The expected product was obtained as a yellow liquid (0.68 g) with 62% yield.

^{1}H NMR (700 MHz, $CDCl_3$) δ 10.34 (d, *J* = 0.9 Hz, 1H), 7.64 - 7.62 (m, 1H), 7.36 (ddd, *J* = 11.6, 8.2, 1.7 Hz, 1H), 7.15 - 7.12 (m, 1H), 6.62 (d, *J* = 1.7 Hz, 1H), 5.37 (d, *J* = 1.3 Hz, 2H), 4.22 (q, *J* = 7.2 Hz, 2H), 1.29 (t, *J* = 7.3 Hz, 3H).
^{13}C{^{1}H} NMR (101 MHz, $CDCl_3$) δ 188.8 (d, *J* = 3.2 Hz), 163.5, 154.8 (d, *J* = 248.8 Hz), 148.5 (d, *J* = 11.1 Hz), 138.4 (q, *J* = 30.2 Hz), 130.1, 127.6 (q, *J* = 5.6 Hz), 124.1 (d, *J* = 7.2 Hz), 123.4 (d, *J* = 3.2 Hz), 122.8 (d, *J* = 20.1 Hz), 122.6 (q, *J* = 275.0 Hz), 65.8 (d, *J* = 8.7 Hz), 61.8, 13.9.
IR-ATR V_{max}: 1728, 1689, 1607, 1586, 1477, 1385, 1355, 1307, 1265, 1246, 1180, 1132, 1088, 1029, 988, 908, 789, 770 cm^{-1}.
HRMS (ESI-TOF) m/z: $(M + H)^+$ calcd for $C_{14}H_{13}F_4O_4$ 321.0750; found: 321.0752.

Ethyl (E)-4,4,4-trifluoro-3-((2-formyl-3-methoxyphenoxy)methyl)but-2-enoate (**1n**)

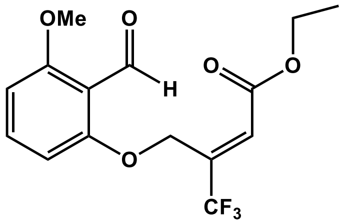

Ethyl (*Z*)-3-(bromomethyl)-4,4,4-trifluorobut-2-enoate (**6a**) (3.83 mmol; 1.0 g), 2-hydroxy-6-methoxybenzaldehyde (3.6 mmol; 0.55 g), potassium carbonate (3.98 mmol; 0.55 g) and acetone (10 mL) were used in the reaction carried out correspondingly to the **General Procedure 3**. The expected product was obtained as an orange oil (0.27 g) with 21% yield.

^{1}H NMR (700 MHz, $CDCl_3$) δ 10.44 (s, 1H), 7.44 (t, *J* = 8.6 Hz, 1H), 6.63 (t, *J* = 8.2 Hz, 2H), 6.59 (d, *J* = 0.9 Hz, 1H), 5.25 (s, 2H), 4.25 (q, *J* = 7.2 Hz, 2H), 3.89 (s, 3H), 1.30 (t, *J* = 7.1 Hz, 3H).
^{13}C{^{1}H} NMR (101 MHz, $CDCl_3$) δ 189.1, 163.8, 161.4, 161.3, 139.1 (q, *J* = 30.2 Hz), 135.7, 127.0 (q, *J* = 5.6 Hz), 122.4 (q, *J* = 275.2 Hz), 115.0, 105.1, 104.8, 61.8, 61.4, 56.1, 14.0.
IR-ATR V_{max}: 1728, 1689, 1597, 1581, 1474, 1399, 1355, 1305, 1255, 1177, 1135, 1106, 1088, 1025, 906, 822, 778 cm^{-1}.
HRMS (ESI-TOF) m/z: $(M + H)^+$ calcd for $C_{15}H_{16}F_3O_5$ 333.0950; found: 333.0948.

Ethyl (E)-4,4,4-trifluoro-3-((3-fluoro-2-formylphenoxy)methyl)but-2-enoate (**1o**)

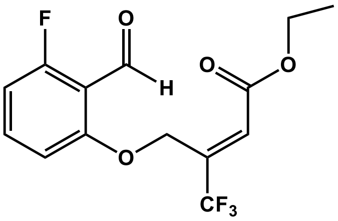

Ethyl (*Z*)-3-(bromomethyl)-4,4,4-trifluorobut-2-enoate (**6a**) (3.8 mmol; 1.0 g), 2-fluoro-6-hydroxybenzaldehyde (3.6 mmol; 0.50 g), potassium carbonate (3.6 mmol; 0.5 g) and acetone (10 mL) were used in the reaction carried out correspondingly to the **General Procedure 3**. The expected product was obtained as a white oil (0.16 g) with 14% yield.

^{1}H NMR (400 MHz, $CDCl_3$) δ 10.40 (dd, *J* = 1.4, 0.6 Hz, 1H), 7.51 (td, *J* = 8.5, 6.2 Hz, 1H), 6.88 (d, *J* = 8.4 Hz, 1H), 6.83 - 6.78 (m, 1H), 6.65 (d, *J* = 1.3 Hz, 1H), 5.32 (d, *J* = 0.7 Hz, 2H), 4.28 (q, *J* = 7.1 Hz, 2H), 1.34 (t, *J* = 7.1 Hz, 3H).
^{13}C{^{1}H} NMR (101 MHz, $CDCl_3$) δ 187.1, 163.9, 163.7, 161.3, 160.8 (d, *J* = 5.6 Hz), 138.7 (q, *J* = 30.5 Hz), 135.9 (d, *J* = 11.9 Hz), 127.1 (q, *J* = 5.6 Hz), 122.3 (q, *J* = 275.2 Hz), 109.9 (d, *J* = 21.4 Hz), 108.1 (d, *J* = 3.2 Hz), 61.9, 61.6, 14.0.
IR-ATR V_{max}: 1726, 1697, 1612, 1579, 1469, 1239, 1181, 1132, 1089, 1070, 1027, 784, 733 cm^{-1}.
HRMS (ESI-TOF) m/z: $(M + H)^+$ calcd for $C_{14}H_{13}F_4O_4$ 321.0750; found: 321.0754.

Ethyl (E)-3-((3-chloro-2-formylphenoxy)methyl)-4,4,4-trifluorobut-2-enoate (**1p**)

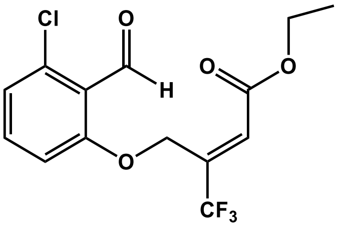

Ethyl (*Z*)-3-(bromomethyl)-4,4,4-trifluorobut-2-enoate (**6a**) (3.83 mmol; 1.0 g), 2-chloro-6-hydroxybenzaldehyde (3.6 mmol; 0.56 g), potassium carbonate (4.05 mmol; 0.56 g), and acetone (10 mL) were used in the reaction carried out correspondingly to the **General Procedure 3**. The expected product was obtained as a yellow liquid (0.18 g) with 14% yield.

^{1}H NMR (700 MHz, $CDCl_3$) δ 10.41 (s, 1H), 7.4 (t, *J* = 8.2 Hz, 1H), 7.06 (d, *J* = 8.2 Hz, 1H), 6.99 - 6.97 (m, 1H), 6.60 (d, *J* = 0.9 Hz, 1H), 5.26 (s, 2H), 4.23 (q, *J* = 7.0 Hz, 2H), 1.29 (t, *J* = 7.1 Hz, 3H).
^{13}C{^{1}H} NMR (101 MHz, $CDCl_3$) δ 188.2, 163.3, 160.4, 138.9, 138.3 (q, *J* = 30.5 Hz), 135.2, 134.0, 132.5, 126.7 (q, *J* = 5.4 Hz), 124.0, 121.9 (q, *J* = 275.7 Hz), 110.9, 61.5, 61.1, 13.6.
IR-ATR V_{max}: 1720, 1610, 1588, 1450, 1352, 1307, 1265, 1198, 1170, 1129, 1090, 1025, 964, 919, 782, 735, 648 cm^{-1}.
HRMS (ESI-TOF) m/z: $(M + H)^+$ calcd for $C_{14}H_{13}ClF_3O_4$ 337.0454; found: 337.0459.

Ethyl (E)-4,4,4-trifluoro-3-((2-formyl-3,5-dimethoxyphenoxy)methyl)but-2-enoate (**1q**)

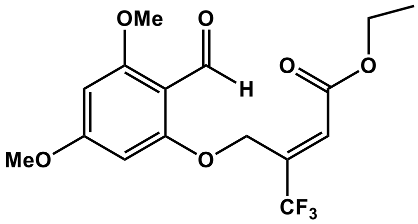

Ethyl (*Z*)-3-(bromomethyl)-4,4,4-trifluorobut-2-enoate (**6a**) (3.83 mmol; 1.0 g), 2-hydroxy-4,6-dimethoxybenzaldehyde (3.63 mmol; 0.66 g), potassium carbonate (3.62 mmol; 0.5 g) and acetone (10 mL) were used in the reaction carried out correspondingly to the **General Procedure 3**. The expected product was obtained as an orange oil (0.23 g) with 17% yield.

^{1}H NMR (700 MHz, $CDCl_3$) δ 10.30 (s, 1H), 6.60 (s, 1H), 6.18 (d, *J* = 1.8 Hz, 1H), 6.12 (d, *J* = 2.2 Hz, 1H), 5.25 (s, 2H), 4.25 (q, *J* = 7.2 Hz, 2H), 3.88 (s, 3H), 3.86 (s, 3H), 1.31 (t, *J* = 7.1 Hz, 3H).
^{13}C{^{1}H} NMR (176 MHz, $CDCl_3$) δ 186.9, 165.7, 163.4, 162.8, 162.7, 138.5 (q, *J* = 30.5 Hz), 126.4 (q, *J* = 5.4 Hz), 121.9 (q, *J* = 275.2 Hz), 108.7, 91.1, 90.7, 61.4, 60.8, 55.6, 55.2, 13.5.
IR-ATR V_{max}: 1728, 1677, 1600, 1573, 1459, 1415, 1334, 1305, 1250, 1207, 1180, 1156, 1117, 1086, 1025, 941, 908, 816, 730 cm^{-1}.
HRMS (ESI-TOF) m/z: $(M + H)^+$ calcd for $C_{16}H_{18}F_3O_6$ 363.1055; found: 363.1050.

Ethyl (E)-3-((2,4-dibromo-6-formylphenoxy)methyl)-4,4,4-trifluorobut-2-enoate (**1r**)

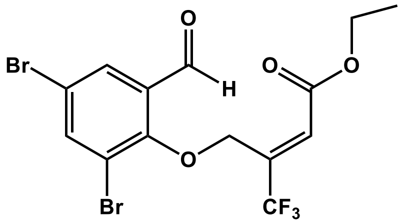

Ethyl (*Z*)-3-(bromomethyl)-4,4,4-trifluorobut-2-enoate (**6a**) (3.83 mmol; 1.0 g), 3,5-dibromosalicylaldehyde (4.97 mmol; 1.4 g), potassium carbonate (5.0 mmol; 0.69 g) and acetone (5 mL) were used in the reaction carried out correspondingly to the **General Procedure 3**. The expected product was obtained as a yellow oil (0.76 g) with 33% yield.

^{1}H NMR (700 MHz, $CDCl_3$) δ 10.23 - 10.22 (m, 1H), 7.95 - 7.94 (m, 1H), 7.91 - 7.90 (m, 1H), 6.62 (s, 1H), 5.26 (s, 2H), 4.19 (qd, *J* = 7.2, 1.4 Hz, 2H), 1.27 (td, *J* = 7.1, 1.4 Hz, 3H).
^{13}C{^{1}H} NMR (101 MHz, $CDCl_3$) δ 187.7, 163.5, 157.4, 141.4, 137.8 (q, *J* = 30.1 Hz), 132.0, 130.6, 127.8 (q, *J* = 5.6 Hz), 122.5 (q, *J* = 275.5 Hz), 118.8, 118.7, 66.7, 61.9, 13.9.
IR-ATR V_{max}: 1727, 1692, 1573, 1443, 1379, 1305, 1201, 1181, 1133, 1087, 1028, 976, 905, 875, 743, 728, 689, 633 cm^{-1}.
HRMS (ESI-TOF) m/z: $(M + H)^+$ calcd for $C_{14}H_{12}Br_2F_3O_4$ 458.9054; found: 458.9055.

Ethyl (E)-3-((2,4-dichloro-6-formylphenoxy)methyl)-4,4,4-trifluorobut-2-enoate (**1s**)

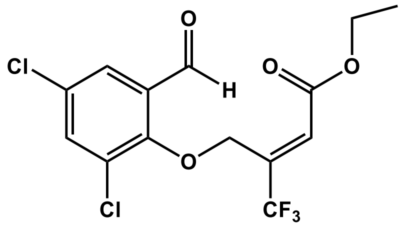

Ethyl (*Z*)-3-(bromomethyl)-4,4,4-trifluorobut-2-enoate (**6a**) (2.23 mmol; 0.58 g), 3,5-dichlorosalicylaldehyde (2.91 mmol; 0.55 g), potassium carbonate (2.91 mmol; 0.40 g) and acetone (3 mL) were used in the reaction carried out correspondingly to the **General Procedure 3**. The expected product was obtained as a yellow oil (0.16 g) with 19% yield.

^{1}H NMR (700 MHz, $CDCl_3$) δ 10.25 (s, 1H), 7.71 (d, *J* = 2.6 Hz, 1H), 7.62 (d, *J* = 2.6 Hz, 1H), 6.62 (d, *J* = 1.3 Hz, 1H), 5.26 (s, 2H), 4.19 (q, *J* = 7.0 Hz, 2H), 1.27 (t, *J* = 7.1 Hz, 3H).
^{13}C{^{1}H} NMR (101 MHz, $CDCl_3$) δ 187.8, 163.5, 155.8, 137.8 (q, *J* = 30.9 Hz), 135.7, 131.5, 131.0, 129.5, 127.9 (q, *J* = 5.8 Hz), 126.7, 122.5 (q, *J* = 275.2 Hz), 66.3, 61.9, 13.9.
IR-ATR V_{max}: 1727, 1695, 1440, 1381, 1351, 1307, 1265, 1205, 1178, 1166, 1131, 1089, 1027, 978, 877 cm^{-1}.
HRMS (ESI-TOF) m/z: $(M + H)^+$ calcd for $C_{14}H_{12}Cl_2F_3O_4$ 371.0065; found: 371.0062.

Ethyl (E)-4,4,4-trifluoro-3-((2-formyl-3,5,6-trimethylphenoxy)methyl)but-2-enoate (**1t**)

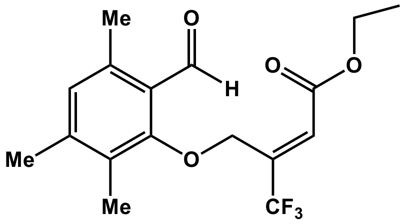

Ethyl (*Z*)-3-(bromomethyl)-4,4,4-trifluorobut-2-enoate (**6a**) (3.8 mmol; 1.0 g), 2-hydroxy-3,4,6-trimethylbenzaldehyde (3.6 mmol; 0.59 g), potassium carbonate (4.27 mmol; 0.59 g) and acetone (10 mL) were used in the reaction carried out correspondingly to the **General Procedure 3**. The expected product was obtained as a yellow oil (0.37 g) with 28% yield.

^{1}H NMR (700 MHz, $CDCl_3$) δ 10.46 (s, 1H), 6.85 (s, 1H), 6.59 (d, *J* = 1.3 Hz, 1H), 4.98 (s, 2H), 4.21 (q, *J* = 7.1 Hz, 2H), 2.52 (s, 3H), 2.28 (s, 3H), 2.19 (s, 3H), 1.28 (t, *J* = 7.3 Hz, 3H).
^{13}C{^{1}H} NMR (101 MHz, $CDCl_3$) δ 192.4, 163.7, 160.8, 144.9, 138.7, 138.4 (q, *J* = 30.2 Hz), 129.7, 127.9, 126.7 (q, *J* = 5.8 Hz), 125.6, 122.6 (q, *J* = 275.5 Hz), 67.1, 20.9, 20.6, 14.0, 11.7.
IR-ATR V_{max}: 1730, 1685, 1606, 1555, 1477, 1454, 1375, 1303, 1285, 1240, 1204, 1178, 1134, 1084, 1028, 775, 638 cm^{-1}.
HRMS (ESI-TOF) m/z: $(M + H)^+$ calcd for $C_{17}H_{20}F_3O_4$ 345.1314; found: 345.1317.

Ethyl (E)-4,4,5,5,6,6,6-heptafluoro-3-((2-formylphenoxy)methyl)hex-2-enoate (**1u**)

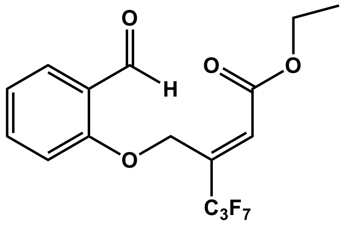

Ethyl (*Z*)-3-(bromomethyl)-4,4,5,5,6,6,6-heptafluorohex-2-enoate (**6b**) (2.77 mmol; 1.0 g), salicylaldehyde (2.64 mmol; 0.32 g), potassium carbonate (2.64 mmol; 0.36 g) and acetone (3.6 mL) were used in the reaction carried out correspondingly to the **General Procedure 3**. The expected product was obtained as a yellow oil (0.13 g) with 12% yield.

^{1}H NMR (700 MHz, $CDCl_3$) δ 10.38 (d, *J* = 0.9 Hz, 1H), 7.86 (dd, *J* = 7.7, 1.7 Hz, 1H), 7.58 - 7.56 (m, 1H), 7.10 - 7.07 (m, 1H), 7.05 (d, *J* = 8.6 Hz, 1H), 6.65 (s, 1H), 5.15 (s, 2H), 4.23 (q, *J* = 7.2 Hz, 2H), 1.27 (t, *J* = 7.1 Hz, 3H).
^{13}C{^{1}H} NMR (101 MHz, $CDCl_3$) δ 189.3, 163.2, 160.5, 138.1 (t, *J* = 21.9 Hz), 135.9, 131.4 (t, *J* = 8.7 Hz), 128.3, 125.2, 121.7, 112.7, 61.8, 60.7, 13.8.
IR-ATR V_{max}: 1731, 1691, 1600, 1485, 1458, 1392, 1341, 1284, 1225, 1180, 1115, 1021, 961, 866, 845, 755 cm^{-1}.
HRMS (ESI-TOF) m/z: $(M + H)^+$ calcd for $C_{16}H_{14}F_7O_4$ 403.0780; found: 403.0778.

Ethyl (E)-4,4,5,5,6,6,7,7,7-nonafluoro-3-((2-formylphenoxy)methyl)hept-2-enoate (**1v**)

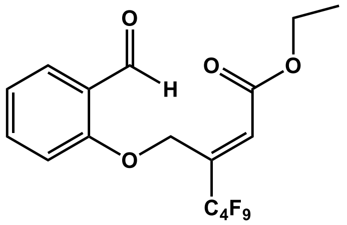

Ethyl (*Z*)-3-(bromomethyl)-4,4,5,5,6,6,7,7,7-nonafluorohept-2-enoate (**6c**) (2.43 mmol; 1.0 g), salicylaldehyde (2.58 mmol; 0.31 g), potassium carbonate (2.43 mmol; 0.34 g) and acetone (3.4 mL) were used in the reaction carried out correspondingly to the **General Procedure 3**. The expected product was obtained as a yellow oil (0.16 g) with 15% yield.

^{1}H NMR (400 MHz, $CDCl_3$) δ 10.39 (d, *J* = 0.9 Hz, 1H), 7.87 (dd, *J* = 7.7, 1.7 Hz, 1H), 7.58 (ddd, *J* = 8.4, 7.3, 1.8 Hz, 1H), 7.12 - 7.06 (m, 2H), 6.67 (s, 1H), 5.17 (s, 2H), 4.24 (q, *J* = 7.2 Hz, 2H), 1.27 (t, *J* = 7.2 Hz, 3H).
^{13}C{^{1}H} NMR (101 MHz, $CDCl_3$) δ 189.3, 163.3, 160.5, 138.2 (t, *J* = 22.3 Hz), 135.9, 131.1 (t, *J* = 8.7 Hz), 128.3, 125.2, 121.7, 112.7, 61.9, 60.7, 13.9.
IR-ATR V_{max}: 1731, 1692, 1600, 1485, 1458, 1190, 1133, 1097, 1021, 858, 832, 755, 742 cm^{-1}.
HRMS (ESI-TOF) m/z: $(M + H)^+$ calcd for $C_{17}H_{14}F_9O_4$ 453.0748; found: 453.0750.

Ethyl (E)-4,4,5,5,6,6,7,7,8,8,9,9,9-tridecafluoro-3-((2-formylphenoxy)methyl)non-2-enoate (**1w**)

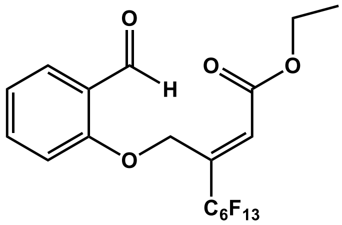

Ethyl (*Z*)-3-(bromomethyl)-4,4,5,5,6,6,7,7,8,8,9,9,9-tridecafluoronon-2-enoate (**6d**) (1.96 mmol; 1.0 g), salicylaldehyde (1.85 g; 0.23 g), potassium carbonate (1.85 mmol; 0.26 g) and acetone (3 mL) were used in the reaction carried out correspondingly to the **General Procedure 3**. The expected product was obtained as a yellow oil (0.16 g) with 15% yield.

^{1}H NMR (700 MHz, $CDCl_3$ δ 10.37 (d, *J* = 0.9 Hz, 1H), 7.86 (dd, *J* = 7.5, 1.9 Hz, 1H), 7.58 - 7.56 (m, 1H), 7.10 - 7.08 (m, 1H), 7.05 (d, *J* = 8.2 Hz, 1H), 6.66 (s, 1H), 5.15 (s, 2H), 4.22 (q, *J* = 7.2 Hz, 2H), 1.27 (t, *J* = 7.1 Hz, 3H).
^{13}C{^{1}H} NMR (101 MHz, $CDCl_3$) δ 189.2, 163.2, 160.5, 138.3 (t, *J* = 21.9 Hz), 135.8, 131.5 (t, *J* = 8.7 Hz), 128.2, 125.2, 121.7, 112.7, 61.8, 60.7, 13.7.
IR-ATR V_{max}: 1732, 1692, 1600, 1485, 1458, 1232, 1190, 1143, 1121, 1098, 1022, 758, 704 cm^{-1}.
HRMS (ESI-TOF) m/z: $(M + H)^+$ calcd for $C_{19}H_{14}F_{13}O_4$ 553.0685; found: 553.0689.

Ethyl (E)-4,4,5,5,6,6,7,7,8,8,9,9,10,10,11,11,11-heptadecafluoro-3-((2-formylphenoxy)methyl)undec-2-enoate (**1x**)

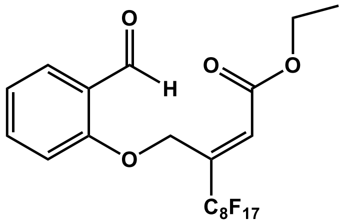

Ethyl (*Z*)-3-(bromomethyl)-4,4,5,5,6,6,7,7,8,8,9,9,10,10,11,11,11-heptadecafluoroundec-2-enoate (**6e**) (1.64 mmol; 1.0 g), salicylaldehyde (1.54 mmol; 0.19 g), potassium carbonate (1.54 mmol; 0.21 g) and acetone (2.5 mL) were used in the reaction carried out correspondingly to the **General Procedure 3**. Expected product was obtained as yellow oil (0.19 g) with 18% yield.

^{1}H NMR (700 MHz, $CDCl_3$) δ 10.38 (d, *J* = 0.9 Hz, 1H), 7.86 (dd, *J* = 7.7, 1.7 Hz, 1H), 7.57 (ddd, *J* = 9.0, 6.5, 2.0 Hz, 1H), 7.09 (t, *J* = 7.5 Hz, 1H), 7.05 (d, *J* = 8.2 Hz, 1H), 6.66 (s, 1H), 5.15 (s, 2H), 4.22 (q, *J* = 7.3 Hz, 2H), 1.26 (t, *J* = 7.1 Hz, 3H).
^{13}C{^{1}H} NMR (101 MHz, $CDCl_3$) δ 189.1, 163.2, 160.5, 138.3 (t, *J* = 21.9 Hz), 135.8, 131.5 (t, *J* = 8.7 Hz), 128.2, 125.2, 121.6, 112.7, 61.8, 60.7, 13.7.
IR-ATR V_{max}: 1732, 1692, 1600, 1485, 1458, 1194, 1145, 1022, 848, 813, 757, 655 cm^{-1}.
HRMS (ESI-TOF) m/z: $(M + H)^+$ calcd for $C_{21}H_{14}F_{17}O_4$ 653.0621; found: 653.0623.

Ethyl (Z)-4,4,5,5,6,6,6-heptafluoro-2-(2-formylphenoxy)-3-methylhex-2-enoate (**3a**)

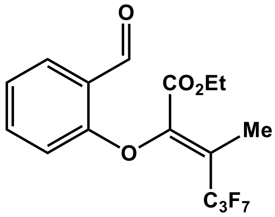

Ethyl (*Z*)-3-(bromomethyl)-4,4,5,5,6,6,6-heptafluorohex-2-enoate (**6b**) (2.77 mmol; 1.0 g), salicylaldehyde (2.64 mmol; 0.32 g), potassium carbonate (2.64 mmol; 0.36 g) and acetone (3.6 mL) were used in the reaction carried out correspondingly to the **General Procedure 3**. The expected product was obtained as a yellow oil (0.039 g) with 4% yield.

^{1}H NMR (700 MHz, $CDCl_3$) δ 10.48 (d, *J* = 0.9 Hz, 1H), 7.91 (dd, *J* = 7.7 Hz, 1.7 Hz, 1H), 7.52 - 7.50 (m, 1H), 7.20 - 7.18 (m, 1H), 6.83 (dd, *J* = 8.2, 0.9 Hz, 1H), 4.09 (q, *J* = 7.2 Hz, 2H), 2.24 (s, 3H), 0.95 (t, *J* = 7.1 Hz, 3H).
^{13}C{^{1}H} NMR (101 MHz, $CDCl_3$) δ 188.7, 161.4, 158.5, 144.3 (t, *J* = 4.8 Hz), 135.5, 128.4, 125.9, 123.9, 123.6 (t, *J* = 22.3 Hz), 115.7, 62.2, 13.5, 13.2 (t, *J* = 5.2 Hz).
IR-ATR V_{max}: 1734, 1696, 1602, 1584, 1481, 1458, 1219, 1201, 1182, 1118, 1049, 957, 928, 864, 760, 752, 707 cm^{-1}.
HRMS (ESI-TOF) m/z: $(M + H)^+$ calcd for $C_{16}H_{14}F_7O_4$ 403.0780; found: 403.0781.

Ethyl (Z)-4,4,5,5,6,6,7,7,7-nonafluoro-2-(2-formylphenoxy)-3-methylhept-2-enoate (**3b**)

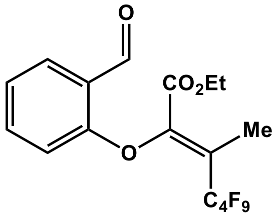

Ethyl (*Z*)-3-(bromomethyl)-4,4,5,5,6,6,7,7,7-nonafluorohept-2-enoate (**6c**) (2.43 mmol; 1.0 g), salicylaldehyde (2.58 mmol; 0.31 g), potassium carbonate (2.43 mmol; 0.34 g) and acetone (3.4 mL) were used in the reaction carried out correspondingly to the **General Procedure 3**. The expected product was obtained as a yellow oil (0.078 g) with 7% yield.

^{1}H NMR (700 MHz, $CDCl_3$) δ 10.47 (d, *J* = 0.9 Hz, 1H), 7.90 (dd, *J* = 7.3, 1.9 Hz, 1H), 7.51 - 7.49 (m, 1H), 7.19 - 7.17 (m, 1H), 6.83 - 6.82 (m, 1H), 4.08 (q, *J* = 7.2 Hz, 2H), 2.23 (s, 3H), 0.94 (t, *J* = 7.1 Hz, 3H).
^{13}C{^{1}H} NMR (101 MHz, $CDCl_3$) δ 188.7, 161.4, 158.5, 144.4 (t, *J* = 4.4 Hz), 135.5, 128.3, 125.9, 123.9, 123.6 (t, *J* = 23.8 Hz), 115.7, 62.2, 13.5, 13.2 (t, *J* = 5.2 Hz).
IR-ATR V_{max}: 1736, 1698, 1603, 1481, 1458, 1230, 1218, 1134, 1097, 1050, 1017, 862, 840, 761, 744, 713, 645 cm^{-1}.
HRMS (ESI-TOF) m/z: $(M + H)^+$ calcd for $C_{17}H_{14}F_9O_4$ 453.0748; found: 453.0746.

Ethyl (Z)-4,4,5,5,6,6,7,7,8,8,9,9,9-tridecafluoro-2-(2-formylphenoxy)-3-methylnon-2-enoate (**3c**)

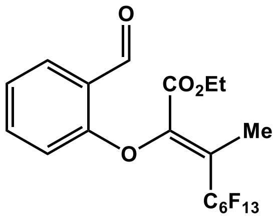

Ethyl (*Z*)-3-(bromomethyl)-4,4,5,5,6,6,7,7,8,8,9,9,9-tridecafluoronon-2-enoate (**6d**) (1.96 mmol; 1.0 g), salicylaldehyde (1.85 g; 0.23 g), potassium carbonate (1.85 mmol; 0.26 g) and acetone (3 mL) were used in the reaction carried out correspondingly to the **General Procedure 3**. The expected product was obtained as a yellow oil (0.096 g) with 9% yield.

^{1}H NMR (700 MHz, $CDCl_3$) δ 10.48 (d, *J* = 0.9 Hz, 1H), 7.91 (dd, *J* = 7.7, 1.7 Hz, 1H), 7.51 (ddd, *J* = 8.2, 7.3, 1.7 Hz, 1H), 7.21 - 7.18 (m, 1H), 6.84 - 6.83 (m, 1H), 4.09 (q, *J* = 7.3 Hz, 2H), 2.25 (s, 3H), 0.95 (t, *J* = 7.1 Hz, 3H).
^{13}C{^{1}H} NMR (101 MHz, $CDCl_3$) δ 188.6, 161.4, 158.5, 144.4 (t, *J* = 4.4 Hz), 135.5, 128.3, 125.9, 123.9, 123.7 (t, *J* = 23.1 Hz), 115.7, 62.2, 13.4, 13.1 (t, *J* = 5.2 Hz).
IR-ATR V_{max}: 1735, 1698, 1603, 1481, 1458, 1233, 1190, 1143, 1049, 762, 706, 657 cm^{-1}.
HRMS (ESI-TOF) m/z: $(M + H)^+$ calcd for $C_{19}H_{14}F_{13}O_4$ 553.0685; found: 553.0688.

Ethyl (Z)-4,4,5,5,6,6,7,7,8,8,9,9,10,10,11,11,11-heptadecafluoro-2-(2-formylphenoxy)-3-methylundec-2-enoate (**3d**)

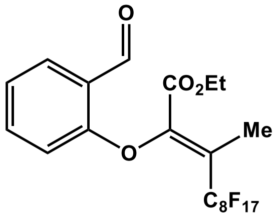

Ethyl (*Z*)-3-(bromomethyl)-4,4,5,5,6,6,7,7,8,8,9,9,10,10,11,11,11-heptadecafluoroundec-2-enoate (**6e**) (1.64 mmol; 1.0 g), salicylaldehyde (1.54 mmol; 0.19 g), potassium carbonate (1.54 mmol; 0.21 g) and acetone (2.5 mL)) were used in the reaction carried out correspondingly to the **General Procedure 3**. The expected product was obtained as a yellow oil (0.064 g) with 6% yield.

^{1}H NMR (700 MHz, $CDCl_3$) δ 10.48 (d, *J* = 0.9 Hz, 1H), 7.91 (dd, *J* = 7.7, 1.7, 1H), 7.52 - 7.50 (m, 1H), 7.21 - 7.18 (m, 1H), 6.83 (d, *J* = 7.7 Hz, 1H), 4.09 (q, *J* = 7.2 Hz, 2H), 2.25 (s, 3H), 0.95 (t, *J* = 7.1 Hz, 3H).
^{13}C{^{1}H} NMR (101 MHz, $CDCl_3$) δ 188.7, 161.4, 158.5, 144.4 (t, *J* = 4.4 Hz), 135.5, 128.3, 125.9, 123.9, 123.7 (t, *J* = 23.1 Hz), 115.7, 62.2, 13.5, 13.2 (t, *J* = 5.2 Hz).
IR-ATR V_{max}: 1736, 1698, 1603, 1481, 1458, 1236, 1200, 1144, 1049, 761, 715, 704, 660 cm^{-1}.
HRMS (ESI-TOF) m/z: $(M + H)^+$ calcd for $C_{21}H_{14}F_{17}O_4$ 653.0621; found: 653.0620.

Ethyl (S)-2-(4-oxo-3-(trifluoromethyl)chroman-3-yl)acetate (**2a**)

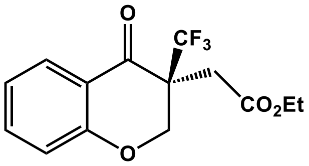

Precatalyst **E** (10.44 mg; 0.022 mmol), *N,N*-diisopropylethylamine (34.5 µl; 0.198 mmol), ethyl (*E*)-4,4,4-trifluoro-3-((2-formylphenoxy)methyl)but-2-enoate (**1a**) (30 mg; 0.099 mmol) and *o*-xylene (1.0 mL) were used in the reaction carried out correspondingly to the **General Procedure 4**. The reaction was carried out for 19 h. The expected product was obtained as a colorless liquid (29.62 mg) with 99% yield. The enantiomeric excess was determined by HPLC with an Phenomenex Amylose Lux-1 3µm column (n–hexane/i–PrOH = 95:5, flow rate 0.7 mL/min, λ = 254 nm).

^{1}H NMR (400 MHz, $CDCl_3$) δ 7.98 (ddd, *J* = 8.0, 1.7, 0.4 Hz, 1H), 7.54 (ddd, *J* = 8.9, 6.6, 1.8 Hz, 1H), 7.09 (ddd, *J* = 8.0, 7.1, 1.0 Hz, 1H), 7.02 (ddd, *J* = 8.3, 1.1, 0.5 Hz, 1H), 4.97 (dq, *J* = 12.2, 1.9 Hz, 1H), 4.73 (d, *J* = 12.2 Hz, 1H), 4.14 (q, *J* = 7.1 Hz, 2H), 3.40 (d, *J* = 17.0 Hz, 1H), 2.61 (d, *J* = 16.9 Hz, 1H), 1.24 (t, *J* = 7.2 Hz, 3H).
^{13}C{^{1}H} NMR (101 MHz, $CDCl_3$) δ 186.0, 168.8, 161.3, 136.7, 127.9, 124.8 (q, *J* = 285.3 Hz), 122.1, 120.4, 118.0, 68.4 (d, *J* = 1.6 Hz), 61.4, 51.5 (q, *J* = 23.6 Hz), 32.9 (q, *J* = 2.4 Hz), 13.9.
IR-ATR V_{max}: 1736, 1695, 1607, 1481, 1467, 1457, 1378, 1315, 1297, 1234, 1175, 1150, 1044, 1019, 779, 758 cm^{-1}.
HRMS (ESI-TOF) m/z: $(M + H)^+$ calcd for $C_{14}H_{14}F_3O_4$ 303.0844; found: 303.0841.
$[\alpha]^D_{25}$ = -12.57° (c 0.079 g/ml, $CHCl_3$)
ee: 98%

Ethyl (S)-2-(6-iodo-4-oxo-3-(trifluoromethyl)chroman-3-yl)acetate (**2b**)

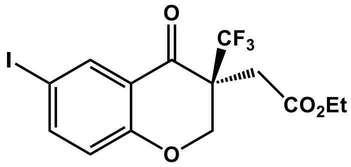

Precatalyst **E** (15.5 mg; 0.033 mmol), *N,N*-diisopropylethylamine (59.2 µl; 0.34 mmol), ethyl (*E*)-4,4,4-trifluoro-3-((2-formyl-4-iodophenoxy)methyl)but-2-enoate (**1b**) (71 mg; 0.17 mmol) and *o*-xylene (1.7 mL) were used in the reaction carried out correspondingly to the **General Procedure 4**. The reaction was carried out for 1 h 20 min. The expected product was obtained as a beige solid (62.62 mg) with 88% yield. The enantiomeric excess was determined by HPLC with an Phenomenex Amylose Lux-1 3µm column (n–hexane/i–PrOH = 90:10, flow rate 1.0 mL/min, λ = 254 nm).

mp: 69.1-72.1 °C.
^{1}H NMR (700 MHz, $CDCl_3$) δ 8.24 (d, *J* = 2.2 Hz, 1H), 7.77 (dd, *J* = 8.5, 2.4 Hz, 1H), 6.79 (d, *J* = 8.6 Hz, 1H), 4.95 - 4.93 (m, 1H), 4.70 (d, *J* = 12.5 Hz, 1H), 4.14 - 4.09 (m, 2H), 3.40 (d, *J* = 17.2 Hz, 1H), 2.55 (d, *J* = 17.2 Hz, 1H), 1.23 (t, *J* = 7.1 Hz, 3H).
^{13}C{^{1}H} NMR (176 MHz, $CDCl_3$) δ 184.5, 168.5, 160.5, 144.6, 136.0, 124.1 (q, *J* = 285.6 Hz), 121.8, 120.0, 84.1, 68.0, 61.3, 50.9 (q, *J* = 23.4 Hz), 32.5, 13.6.

IR-ATR V_{max}: 1725, 1692, 1592, 1473, 1414, 1322, 1279, 1202, 1172, 1106, 1066, 1017, 826 cm^{-1}.
HRMS (ESI-TOF) m/z: $(M + H)^+$ calcd for $C_{14}H_{13}F_3IO_4$ 428.9811; found: 428.9810.
$[\alpha]^D_{25}$ = +9.94° (c 0.026 g/ml, $CHCl_3$).
ee: 94%

Ethyl (S)-2-(6-bromo-4-oxo-3-(trifluoromethyl)chroman-3-yl)acetate (**2c**)

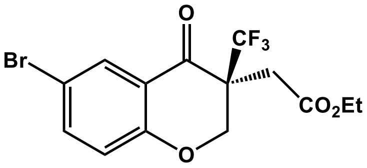

Precatalyst **E** (7.35 mg; 0.016 mmol), *N*,*N*-diisopropylethylamine (27 µl; 0.16 mmol), ethyl (*E*)-3-((4-bromo-2-formylphenoxy)methyl)-4,4,4-trifluorobut-2-enoate (**1c**) (30 mg; 0.079 mmol) and *o*-xylene (0.79 mL) were used in the reaction carried out correspondingly to the **General Procedure 4**. The reaction was carried out for 21 h. Expected product was obtained as yellow oil (29.96 mg) with 99% yield. The enantiomeric excess was determined by HPLC with an Phenomenex Cellulose Lux-1 3µm column (n–hexane/i–PrOH = 90:10, flow rate 1.0 mL/min, λ = 254 nm).

^{1}H NMR (400 MHz, $CDCl_3$) δ 8.08 (d, *J* = 2.5 Hz, 1H), 7.61 (dd, *J* = 8.9, 2.5 Hz, 1H), 6.93 (d, *J* = 8.8 Hz, 1H), 4.96 (dq, *J* = 12.3, 1.9 Hz, 1H), 4.72 (d, *J* = 12.2 Hz, 1H), 4.14 (qd, *J* = 7.1, 1.3 Hz, 2H), 3.42 (d, *J* = 17.1 Hz, 1H), 2.58 (d, *J* = 17.2 Hz, 1H), 1.25 (t, *J* = 7.2 Hz, 3H).
$^{13}C\{^1H\}$ NMR (101 MHz, CDCl3) δ 185.0, 168.8, 160.2, 139.4, 130.3, 124.6 (q, *J* = 285.6 Hz), 121.7, 120.1, 114.9, 68.5, 61.6, 51.3 (q, *J* = 23.6 Hz), 32.9 (q, *J* = 2.4 Hz), 14.0.
IR-ATR V_{max}: 1734, 1698, 1601, 1476, 1458, 1417, 1377, 1276, 1174, 1141, 1103, 1069, 1020, 826, 607, 527 cm^{-1}.
HRMS (ESI-TOF) m/z: $(M + H)^+$ calcd for $C_{14}H_{13}BrF_3O_4$ 380.9949; found: 380.9954.
$[\alpha]^D_{25}$ = +5.18° (c 0.0064 g/ml, $CHCl_3$).
ee: 95%

Ethyl (S)-2-(6-fluoro-4-oxo-3-(trifluoromethyl)chroman-3-yl)acetate (**2d**)

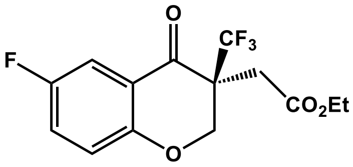

Precatalyst **E** (10.00 mg; 0.02 mmol), *N*,*N*-diisopropylethylamine (34.8 µl; 0.2 mmol), ethyl (*E*)-4,4,4-trifluoro-3-((4-fluoro-2-formylphenoxy)methyl)but-2-enoate (**1d**) (32.0 mg; 0.10 mmol) and *o*-xylene (0.5 mL) were used in the reaction carried out correspondingly to the **General Procedure 4**. The reaction was carried out for 5 h. The product was further purified by flash chromatography. The expected product was obtained as a brown oil (21.68 mg) with 68% yield. The enantiomeric excess was determined by HPLC with an Phenomenex Amylose Lux-1 3µm column (n–hexane/i–PrOH = 90:10, flow rate 1.0 mL/min, λ = 254 nm).

^{1}H NMR (700 MHz, $CDCl_3$) δ 7.63 (dd, *J* = 8.2, 3.0 Hz, 1H), 7.30 - 7.26 (m, 1H), 7.02 (dd, *J* = 9.0, 4.3 Hz, 1H), 4.96 (dq, *J* = 12.1, 2.1 Hz, 1H), 4.72 (d, *J* = 12.5 Hz, 1H), 4.17 - 4.12 (m, 2H), 3.43 (d, *J* = 17.2 Hz, 1H), 2.61 (d, *J* = 17.2 Hz, 1H), 1.26 (t, *J* = 7.1 Hz, 3H).
^{13}C{^{1}H} NMR (101 MHz, $CDCl_3$) δ 185.5, 168.8, 157.6 (d, *J* = 242.4 Hz), 157.5 (d, *J* = 1.6 Hz), 124.6 (q, *J* = 285.6 Hz), 124.3 (d, *J* = 24.6 Hz), 120.9 (d, *J* = 7.2 Hz), 119.8 (d, *J* = 7.2 Hz), 112.8 (d, *J* = 23.0 Hz), 68.6, 61.6, 51.4 (q, *J* = 23.8 Hz), 33.0 (q, *J* = 2.4 Hz), 13.9.
IR-ATR V_{max}: 1734, 1698, 1489, 1437, 1275, 1230, 1181, 1158, 1129, 1022, 884, 770 cm^{-1}.
HRMS (ESI-TOF) m/z: $(M + H)^+$ calcd for $C_{14}H_{13}F_4O_4$ 321.0750; found: 321.0747.
$[\alpha]^D{}_{25}$ = -4.26° (c 0.0073 g/ml, $CHCl_3$).
ee: 94%

Ethyl (S)-2-(6-methoxy-4-oxo-3-(trifluoromethyl)chroman-3-yl)acetate (**2e**)

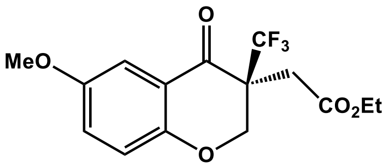

Precatalyst **E** (8.43 mg; 0.018 mmol), *N,N*-diisopropylethylamine (31 μl; 0.18 mmol), ethyl (*E*)-4,4,4-trifluoro-3-((2-formyl-4-methoxyphenoxy)methyl)but-2-enoate (**1e**) (30 mg; 0.090 mmol) and *o*-xylene (0.90 mL) were used in the reaction carried out correspondingly to the **General Procedure 4**. The reaction was carried out for 24 h. The expected product was obtained as an orange oil (29.00 mg) with 97% yield. The enantiomeric excess was determined by HPLC with an Phenomenex Cellulose Lux-1 3μm column (n–hexane/i–PrOH = 90:10, flow rate 1.0 mL/min, λ = 254 nm).

^{1}H NMR (700 MHz, $CDCl_3$) δ 7.36 (d, *J* = 3.0 Hz, 1H), 7.13 (dd, *J* = 9.0, 3.4 Hz, 1H), 6.93 (d, *J* = 9.0 Hz, 1H), 4.89 (dd, *J* = 12.0, 1.7 Hz, 1H), 4.66 (d, *J* = 12.0 Hz, 1H), 4.11 (q, *J* = 7.0 Hz, 2H), 3.81 (s, 3H), 3.38 (d, *J* = 16.8 Hz, 1H), 2.58 (d, *J* = 16.8 Hz, 1H), 1.22 (t, *J* = 7.1 Hz, 3H).
^{13}C{^{1}H} NMR (101 MHz, $CDCl_3$) δ 186.1, 168.9, 156.0, 154.6, 126.2, 123.3, 120.3, 119.4, 107.9, 68.6, 61.5, 55.8, 51.4 (q, *J* = 23.6 Hz), 33.0 (q, *J* = 2.4 Hz), 13.9.
IR-ATR V_{max}: 1734, 1691, 1492, 1429, 1286, 1181, 1163, 1097, 1068, 1021, 870, 830 cm^{-1}.
HRMS (ESI-TOF) m/z: $(M + H)^+$ calcd for $C_{15}H_{16}F_3O_5$ 333.0950; found: 333.0952.
$[\alpha]^D{}_{25}$ = -13.13° (c 0.0075 g/ml, $CHCl_3$)
ee: 98%

Ethyl (S)-2-(4-oxo-6-(trifluoromethoxy)-3-(trifluoromethyl)chroman-3-yl)acetate (**2f**)

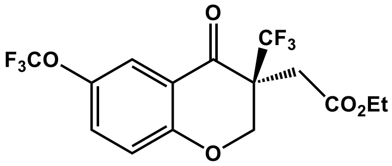

Precatalyst **E** (15.50 mg; 0.033 mmol), *N,N*-diisopropylethylamine (59.2 μl; 0.34 mmol), ethyl (*E*)-4,4,4-trifluoro-3-((2-formyl-4-methylphenoxy)methyl)but-2-enoate (**1f**) (64.0 mg; 0.17 mmol) and *o*-xylene (1.7 mL) were used in the reaction carried out correspondingly to the **General Procedure 4**. The reaction was carried out for 1 h 20 min. The expected product was obtained as a yellow oil (65.00 mg) with 99% yield. The enantiomeric excess was determined

by HPLC with an Phenomenex Cellulose Lux-1 3μm column (n–hexane/i–PrOH = 98:2, flow rate 1.0 mL/min, λ = 254 nm).

^{1}H NMR (700 MHz, $CDCl_3$) δ 7.82 (d, *J* = 2.6 Hz, 1H), 7.41 (dd, *J* = 9.0, 3.0 Hz, 1H), 7.07 (d, *J* = 9.0 Hz, 1H), 5.00 (dd, *J* = 12.5, 1.7 Hz, 1H), 4.76 (d, *J* = 12.5 Hz, 1H), 4.17 - 4.13 (m, 2H), 3.46 (d, *J* = 17.2 Hz, 1H), 2.61 (d, *J* = 17.2 Hz, 1H), 1.26 (t, *J* = 7.1 Hz, 3H).
^{13}C{^{1}H} NMR (176 MHz, $CDCl_3$) δ 184.8, 168.4, 159.2, 143.2, 129.5, 124.1 (q, *J* = 285.6 Hz), 120.3, 120.0 (q, *J* = 257.8 Hz), 119.5, 119.4, 68.2, 61.3, 50.9 (q, *J* = 24.0 Hz), 32.5, 13.5.
IR-ATR V_{max}: 1736, 1702, 1620, 1490, 1438, 1157, 1022, 835, 781 cm^{-1}.
HRMS (ESI-TOF) m/z: $(M + H)^+$ calcd for $C_{15}H_{13}F_6O_5$ 387.0667; found: 387.0666.
$[\alpha]^{D}_{25}$ = +24.19° (c 0.026 g/ml, $CHCl_3$).
ee: 96%

Ethyl (S)-2-(6-nitro-4-oxo-3-(trifluoromethyl)chroman-3-yl)acetate (**2g**)

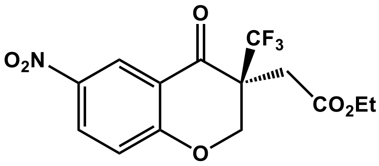

Precatalyst **E** (8.07 mg; 0.017 mmol), *N,N*-diisopropylethylamine (30.1 μl; 0.17 mmol), ethyl (*E*)-4,4,4-trifluoro-3-((2-formyl-4-nitrophenoxy)methyl)but-2-enoate (**1g**) (30 mg; 0.086 mmol) and *o*-xylene (0.87 mL) were used in the reaction carried out correspondingly to the **General Procedure 4**. The reaction was carried out for 24 h. The expected product was obtained as a yellow oil (24.88 mg) with 83% yield. The enantiomeric excess was determined by HPLC with an Phenomenex Cellulose Lux-1 3μm column (n–hexane/i–PrOH = 90:10, flow rate 1.0 mL/min, λ = 254 nm).

^{1}H NMR (400 MHz, $CDCl_3$) δ 8.87 (dd, *J* = 2.8, 0.2 Hz, 1H), 8.40 (dd, *J* = 9.2, 2.8 Hz, 1H), 7.17 (dd, *J* = 9.2, 0.4 Hz, 1H), 5.10 (dq, *J* = 12.5, 1.9 Hz, 1H), 4.85 (d, *J* = 12.5 Hz, 1H), 4.19 - 4.10 (m, 2H), 3.51 (d, *J* = 17.5 Hz, 1H), 2.61 (d, *J* = 17.5 Hz, 1H), 1.27 (t, *J* = 7.2 Hz, 3H).
^{13}C{^{1}H} NMR (101 MHz, $CDCl_3$) δ 184.5, 168.8, 165.0, 142.6, 130.9, 124.4, 124.4 (q, *J* = 284.5 Hz), 119.9, 119.5, 68.9, 61.9, 51.3 (q, *J* = 24.1 Hz), 32.8 (q, *J* = 2.4 Hz), 14.0.
IR-ATR V_{max}: 1732, 1706, 1619, 1587, 1528, 1483, 1437, 1339, 1279, 1174, 1112, 1065, 1017, 986, 914, 842, 747, 619 cm^{-1}.
HRMS (ESI-TOF) m/z: $(M + H)^+$ calcd for $C_{14}H_{13}F_3NO_6$ 348.0695; found: 348.0694.
$[\alpha]^{D}_{25}$ = +22.03° (c 0.0093 g/ml, $CHCl_3$).
ee: 96%

Ethyl (S)-2-(7-methyl-4-oxo-3-(trifluoromethyl)chroman-3-yl)acetate (**2h**)

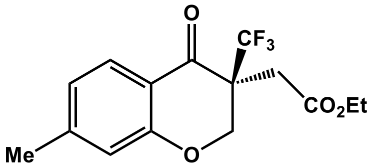

Precatalyst **E** (8.86 mg; 0.019 mmol), *N,N*-diisopropylethylamine (33 μl; 0.19 mmol), ethyl (E)-4,4,4-trifluoro-3-((2-formyl-5-methylphenoxy)methyl)but-2-enoate (**1h**) (30 mg; 0.095

mmol) and *o*-xylene (0.95 mL) were used in the reaction carried out correspondingly to the **General Procedure 4**. The reaction was carried out for 22 h. The expected product was obtained as a yellow oil (29.00 mg) with 97% yield. The enantiomeric excess was determined by HPLC with an Phenomenex Cellulose Lux-1 3μm column (n–hexane/i–PrOH = 90:10, flow rate 1.0 mL/min, λ = 254 nm).

^{1}H NMR (700 MHz, $CDCl_3$) δ 7.87 (d, *J* = 8.2 Hz, 1H), 6.92 - 6.90 (m, 1H), 6.83 (s, 1H), 4.97 - 4.93 (m, 1H), 4.71 (d, *J* = 12.5 Hz, 1H), 4.14 (q, *J* = 7.3 Hz, 2H), 3.38 (d, *J* = 16.8 Hz, 1H), 2.61 (d, *J* = 16.8 Hz, 1H), 2.40 (s, 3H), 1.25 (t, *J* = 7.1 Hz, 3H).
^{13}C{^{1}H} NMR (101 MHz, $CDCl_3$) δ 185.6, 168.9, 161.3, 148.6, 127.8, 124.8 (q, *J* = 285.3 Hz), 123.5, 118.2, 118.0, 68.4 (d, *J* = 1.6 Hz), 61.4, 51.4 (q, *J* = 23.6 Hz), 32.9 (q, *J* = 2.4 Hz), 22.0, 14.0.
IR-ATR V_{max}: 1736, 1690, 1615, 1377, 1288, 1230, 1174, 1103, 1023, 943, 923, 821 cm^{-1}.
HRMS (ESI-TOF) m/z: $(M + H)^+$ calcd for $C_{15}H_{16}F_3O_4$ 317.1001; found: 317.1005.
$[\alpha]^D_{25}$ = +10.44° (c 0.013 g/ml, $CHCl_3$)
ee: 98%

Ethyl (S)-2-(7-methoxy-4-oxo-3-(trifluoromethyl)chroman-3-yl)acetate (**2i**)

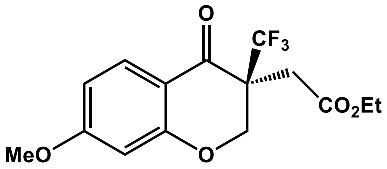

Precatalyst **E** (8.43 mg; 0.018 mmol), *N*,*N*-diisopropylethylamine (31 μl; 0.18 mmol), ethyl (*E*)-4,4,4-trifluoro-3-((2-formyl-5-methoxyphenoxy)methyl)but-2-enoate (**1i**) (30 mg; 0.090 mmol) and *o*-xylene (0.90 mL) were used in the reaction carried out correspondingly to the **General Procedure 4**. The reaction was carried out for 19 h. The expected product was obtained as a yellow oil (26.00 mg) with 87% yield. The enantiomeric excess was determined by HPLC with an Phenomenex Cellulose Lux-1 3μm column (n–hexane/i–PrOH = 90:10, flow rate 1.0 mL/min, λ = 254 nm).

^{1}H NMR (700 MHz, $CDCl_3$) δ 7.91 (d, *J* = 8.6 Hz, 1H), 6.65 (dd, *J* = 9.0, 2.4 Hz, 1H), 6.45 (d, *J* = 2.6 Hz, 1H), 4.97 (dd, *J* = 12.3, 1.9 Hz, 1H), 4.72 (d, *J* = 12.1 Hz, 1H), 4.15 (q, *J* = 7.3 Hz, 2H), 3.87 (s, 3H), 3.36 (d, *J* = 16.8 Hz, 1H), 2.60 (d, *J* = 16.8 Hz, 1H), 1.25 (t, *J* = 7.3 Hz, 3H).
^{13}C{^{1}H} NMR (176 MHz, $CDCl_3$) δ 185.3, 169.9, 167.7, 164.4, 130.7, 125.9 (q, *J* = 285.6 Hz), 115.3, 111.8, 101.8, 69.7, 62.4, 56.7, 52.2 (q, *J* = 24.0 Hz), 33.8, 14.9.
IR-ATR V_{max}: 1728, 1679, 1610, 1577, 1189, 1172, 1098, 1023, 942, 927, 840, 825, 765 cm^{-1}.
HRMS (ESI-TOF) m/z: $(M + H)^+$ calcd for $C_{15}H_{16}F_3O_5$ 333.0950; found: 333.0949.
$[\alpha]^D_{25}$ = +22.73° (c 0.0096 g/ml, $CHCl_3$)
ee: 96%

Ethyl (S)-2-(7-fluoro-4-oxo-3-(trifluoromethyl)chroman-3-yl)acetate (**2j**)

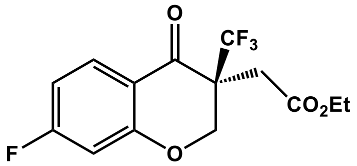

Precatalyst **E** (8.75 mg; 0.019 mmol), *N,N*-diisopropylethylamine (33 µl; 0.19 mmol), ethyl (*E*)-4,4,4-trifluoro-3-((5-fluoro-2-formylphenoxy)methyl)but-2-enoate (**1j**) (30 mg; 0.094 mmol) and *o*-xylene (0.94 mL) were used in the reaction carried out correspondingly to the **General Procedure 4**. The reaction was carried out for 21 h. The expected product was obtained as a brown oil (26.00 mg) with 87% yield. The enantiomeric excess was determined by HPLC with an Phenomenex Cellulose Lux-1 3µm column (n–hexane/i–PrOH = 90:10, flow rate 1.0 mL/min, λ = 254 nm).

^{1}H NMR (700 MHz, $CDCl_3$) δ 8.00 (dd, *J* = 8.9, 6.5 Hz, 1H), 6.81 (ddd, *J* = 8.8, 8.1, 2.3 Hz, 1H), 6.71 (dd, *J* = 9.6, 2.4 Hz, 1H), 5.00 (dq, *J* = 12.3, 1.9 Hz, 1H), 4.74 (d, *J* = 12.2 Hz, 1H), 4.14 (q, *J* = 7.2 Hz, 2H), 3.41 (d, *J* = 17.1, 1H), 2.58 (d, *J* = 17.1 Hz, 1H), 1.25 (t, *J* = 7.2 Hz, 3H).
^{13}C{^{1}H} NMR (101 MHz, $CDCl_3$) δ 184.7, 168.9, 167.9 (d, *J* = 258.3 Hz), 163.0 (d, *J* = 13.5 Hz), 130.6 (d, *J* = 11.1 Hz), 124.6 (q, *J* = 285.3 Hz), 117.4, 110.7 (d, *J* = 23.0 Hz), 104.9 (d, *J* = 25.4 Hz), 68.9 (d, *J* = 1,6 Hz), 61.6, 51.3 (q, *J* = 23.8 Hz), 32.8 (q, *J* = 2.4 Hz), 14.0.
IR-ATR V_{max}: 1734, 1696, 1613, 1589, 1439, 1395, 1378, 1245, 1176, 1146, 1095, 1022, 953, 855, 822, 767 cm^{-1}.
HRMS (ESI-TOF) m/z: $(M + H)^+$ calcd for $C_{14}H_{13}F_4O_4$ 321.0750; found: 321.0746.
$[\alpha]^D_{25}$ = +18.05° (c 0.011 g/ml, $CHCl_3$).
ee: 96%

Ethyl (S)-2-(7-bromo-4-oxo-3-(trifluoromethyl)chroman-3-yl)acetate (**2k**)

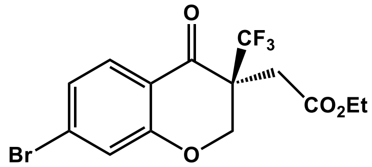

Precatalyst **E** (14.71 mg; 0.031 mmol), *N,N*-diisopropylethylamine (55.7 µl; 0.32 mmol), ethyl (*E*)-3-((5-bromo-2-formylphenoxy)methyl)-4,4,4-trifluorobut-2-enoate (**1k**) (60 mg; 0.16 mmol) and *o*-xylene (1.6 mL) were used in the reaction carried out correspondingly to the **General Procedure 4**. The reaction was carried out for 3 h. The expected product was obtained as a white solid (60.56 mg) with 99% yield. The enantiomeric excess was determined by HPLC with an Phenomenex Amylose Lux-1 3µm column (n–hexane/i–PrOH = 90:10, flow rate 1.0 mL/min, λ = 254 nm).

mp: 69.2-72.6°C.
^{1}H NMR (700 MHz, $CDCl_3$) δ 7.81 (d, *J* = 8.6 Hz, 1H), 7.22 - 7.20 (m, 2H), 4.97 - 4.94 (m, 1H), 4.70 (d, *J* = 12.1 Hz, 1H), 4.11 (qd, *J* = 7.2, 1.3 Hz, 2H), 3.40 (d, *J* = 17.2 Hz, 1H), 2.56 (d, *J* = 17.2 Hz, 1H), 1.23 (t, *J* = 7.1 Hz, 3H).

^{13}C{^{1}H} NMR (176 MHz, $CDCl_3$) δ 185.0, 168.4, 161.0, 131.1, 128.7, 125.5, 124.2 (q, *J* = 283.9 Hz), 120.9, 119.0, 68.3, 61.2, 51.0 (q, *J* = 24.0 Hz), 32.4, 13.6.
IR-ATR V_{max}: 1727, 1697, 1599, 1563, 1411, 1384, 1192, 1177, 1040, 1020, 940, 871, 819 cm^{-1}.
HRMS (ESI-TOF) m/z: $(M + H)^+$ calcd for $C_{14}H_{13}BrF_3O_4$ 380.9949; found: 380.9952.
$[\alpha]^D{}_{25}$ = +28.90° (c 0.029 g/ml, $CHCl_3$).
ee: 98%

Ethyl (S)-2-(7-chloro-4-oxo-3-(trifluoromethyl)chroman-3-yl)acetate (**2l**)

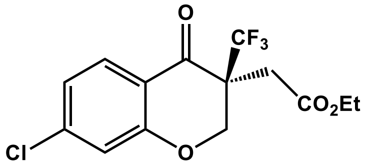

Precatalyst **E** (8.32 mg; 0.018 mmol), *N,N*-diisopropylethylamine (31.0 μl; 0.18 mmol), ethyl (*E*)-3-((5-chloro-2-formylphenoxy)methyl)-4,4,4-trifluorobut-2-enoate (**1l**) (30 mg; 0.089 mmol) and *o*-xylene (0.9 mL) were used in the reaction carried out correspondingly to the **General Procedure 4**. The reaction was carried out for 19 h. The expected product was obtained as an orange oil (29.91 mg) with 99% yield. The enantiomeric excess was determined by HPLC with an Phenomenex Cellulose Lux-1 3μm column (n–hexane/i–PrOH = 90:10, flow rate 1.0 mL/min, λ = 254 nm).

^{1}H NMR (700 MHz, $CDCl_3$) δ 7.89 (d, *J* = 8.6 Hz, 1H), 7.05 (dd, *J* = 8.6, 1.9 Hz, 1H), 7.03 (d, *J* = 1.7 Hz, 1H), 4.96 (dd, *J* = 12.5, 1.9 Hz, 1H), 4.71 (d, *J* = 12.1 Hz, 1H), 4.11 (qd, *J* = 7.3, 1.7 Hz, 2H), 3.40 (d, *J* = 17.2 Hz, 1H), 2.56 (d, *J* = 17.2 Hz, 1H), 1.22 (t, *J* = 7.3 Hz, 3H).
^{13}C{^{1}H} NMR (101 MHz, CDCl3) δ 185.1, 168.8, 161.6, 142.8, 129.1, 124.6 (q, *J* = 285.6 Hz), 123.0, 119.0, 118.2, 66.7, 61.6, 51.4 (q, *J* = 23.8 Hz), 32.8, 14.0.
IR-ATR V_{max}: 1735, 1697, 1600, 1569, 1425, 1390, 1378, 1246, 1231, 1176, 1076, 1067, 1039, 1021, 941, 864, 821, 765 cm^{-1}.
HRMS (ESI-TOF) m/z: $(M + H)^+$ calcd for $C_{14}H_{13}ClF_3O_4$ 337.0454; found: 337.0449.
$[\alpha]^D{}_{25}$ = +21.63° (c 0.0078 g/ml, $CHCl_3$).
ee: 98%

Ethyl (S)-2-(8-fluoro-4-oxo-3-(trifluoromethyl)chroman-3-yl)acetate (**2m**)

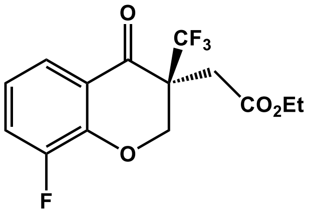

Precatalyst **E** (18.68 mg; 0.04 mmol), *N,N*-diisopropylethylamine (69.7 μl; 0.40 mmol), ethyl (*E*)-4,4,4-trifluoro-3-((2-fluoro-6-formylphenoxy)methyl)but-2-enoate (**1m**) (64.05 mg; 0.20 mmol) and *o*-xylene (2.0 mL) were used in the reaction carried out correspondingly to the **General Procedure 4**. The reaction was carried out for 23 h. The expected product was obtained as a yellow oil (63.54 mg) with 99% yield. The enantiomeric excess was determined

by HPLC with an Phenomenex Amylose Lux-1 3μm column (n–hexane/i–PrOH = 90:10, flow rate 1.0 mL/min, λ = 254 nm).

^{1}H NMR (700 MHz, $CDCl_3$) δ 7.78 - 7.76 (m, 1H), 7.37 (ddd, *J* = 10.1, 8.2, 1.5 Hz, 1H), 7.04 (td, *J* = 8.0, 4.5 Hz, 1H), 5.05 (dd, *J* = 12.5, 1.7 Hz, 1H), 4.84 (d, *J* = 12.5 Hz, 1H), 4.17 - 4.13 (m, 1H), 3.47 (d, *J* = 17.2 Hz, 1H), 2.61 (d, *J* = 17.2 Hz, 1H), 1.26 (t, *J* = 7.3 Hz, 3H).
^{13}C{^{1}H} NMR (101 MHz, $CDCl_3$) δ 185.2 (d, *J* = 3.2 Hz), 168.8, 151.5 (d, *J* = 249.5 Hz), 149.6 (d, *J* = 11.9 Hz), 124.6 (q, *J* = 285.6 Hz), 122.9 (d, *J* = 4.0 Hz), 122.6 (d, *J* = 17.5 Hz), 122.4, 121.5 (d, *J* = 6.4 Hz), 69.0 (d, *J* = 1.6 Hz), 61.6, 51.6 (q, *J* = 23.8 Hz), 32.9 (q, *J* = 2.4 Hz), 13.9.
IR-ATR V_{max}: 1735, 1701, 1619, 1492, 1455, 1378, 1293, 1260, 1237, 1183, 1162, 1069, 1023, 1002, 816, 797 cm^{-1}.
HRMS (ESI-TOF) m/z: $(M + H)^+$ calcd for $C_{14}H_{13}F_4O_4$ 321.0750; found: 321.0753.
[α]^D$_{25}$ = +31.85° (c 0.0058 g/ml, $CHCl_3$).
ee: 86%

Ethyl (S)-2-(5-methoxy-4-oxo-3-(trifluoromethyl)chroman-3-yl)acetate (**2n**)

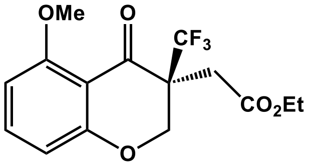

Precatalyst **E** (19.7 mg; 0.042 mmol), *N,N*-diisopropylethylamine (73.2 μl; 0.42 mmol), ethyl (*E*)-4,4,4-trifluoro-3-((2-formyl-3-methoxyphenoxy)methyl)but-2-enoate (**1n**) (70 mg; 0.21 mmol) and *o*-xylene (2.1 mL) were used in the reaction carried out correspondingly to the **General Procedure 4**. The reaction was carried out for 26.5 h. The expected product was obtained as yellow oil (69.36 mg) with 99% yield. The enantiomeric excess was determined by HPLC with an Phenomenex Amylose Lux-1 3μm column (n–hexane/i–PrOH = 98:2, flow rate 1.0 mL/min, λ = 254 nm).

^{1}H NMR (700 MHz, $CDCl_3$) δ 7.41 (t, *J* = 8.4 Hz, 1H), 6.58 (dd, *J* = 8.2, 0.9 Hz, 1H), 6.56 (d, *J* = 8.6 Hz, 1H), 4.89 (dd, *J* = 12.1, 1.7 Hz, 1H), 4.65 (d, *J* = 12.1 Hz, 1H), 4.11 (qd, *J* = 7.1, 2.8 Hz, 2H), 3.92 (s, 3H), 3.36 (d, *J* = 16.8 Hz, 1H), 2.57 (d, *J* = 16.8 Hz, 1H), 1.22 (t, *J* = 7.3 Hz, 3H).
^{13}C{^{1}H} NMR (101 MHz, $CDCl_3$) δ 183.9, 169.1, 163.0, 161.6, 136.8, 124.9 (q, *J* = 285.6 Hz), 111.0, 109.9, 104.7, 67.9 (d, *J* = 1.6 Hz), 61.4, 56.2, 52.3 (q, *J* = 23.3 Hz), 33.2 (q, *J* = 2.4 Hz), 13.9.
IR-ATR V_{max}: 1728, 1689, 1601, 1476, 1175, 1097, 1023, 804, 763, 741 cm^{-1}.
HRMS (ESI-TOF) m/z: $(M + H)^+$ calcd for $C_{15}H_{16}F_3O_5$ 333.0950; found: 333.0956.
[α]^D$_{25}$ = +32.42° (c 0.03 g/ml, $CHCl_3$).
ee: 84%

Ethyl (S)-2-(5-fluoro-4-oxo-3-(trifluoromethyl)chroman-3-yl)acetate (**2o**)

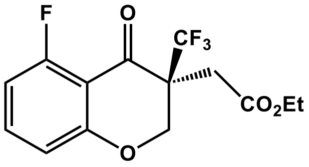

Precatalyst **E** (8.75 mg; 0.019 mmol), *N,N*-diisopropylethylamine (32.7 µl; 0.19 mmol), ethyl (*E*)-4,4,4-trifluoro-3-((3-fluoro-2-formylphenoxy)methyl)but-2-enoate (**1o**) (30.0 mg; 0.094 mmol) and *o*-xylene (0.47 mL) were used in the reaction carried out correspondingly to the **General Procedure 4**. The reaction was carried out for 46 h. The product was further purified by flash chromatography. The expected product was obtained as a colorless oil (10.87 mg) with 36% yield. The enantiomeric excess was determined by HPLC with an Phenomenex Cellulose Lux-1 3µm column (n–hexane/i–PrOH = 90:10, flow rate 1.0 mL/min, λ = 254 nm).

^{1}H NMR (400 MHz, $CDCl_3$) δ 7.48 (td, *J* = 8.4, 5.9 Hz, 1H), 6.83 (dt, *J* = 8.5, 1.1 Hz, 1H), 6.78 (ddd, *J* = 10.6, 8.3, 1.0 Hz, 1H), 4.98 (dq, *J* = 12.3, 2.1 Hz, 1H), 4.72 (d, *J* = 12.2 Hz, 1H), 4.15 (q, *J* = 7.2 Hz, 2H), 3.42 (d, *J* = 17.0 Hz, 1H), 2.59 (d, *J* = 17.1 Hz, 1H), 1.25 (t, *J* = 7.2 Hz, 3H).
^{13}C{^{1}H} NMR (101 MHz, $CDCl_3$) δ 183.4, 168.9, 162.2 (d, *J* = 268.6 Hz), 162.1, 136.8 (d, *J* = 11.9 Hz), 124.6 (q, *J* = 285.3 Hz), 113.8 (d, *J* = 4.0 Hz), 109.9, 109.6, 68.3, 61.6, 52.0 (q, *J* = 23.6 Hz), 33.0 (q, *J* = 2.4 Hz), 13.9.
IR-ATR V_{max}: 1729, 1703, 1621, 1579, 1477, 1468, 1378, 1307, 1286, 1238, 1176, 1137, 1091, 1072, 1025, 1006, 791 cm^{-1}.
HRMS (ESI-TOF) m/z: $(M + H)^+$ calcd for $C_{14}H_{13}F_4O_4$ 321.0750; found: 321.0755.
$[\alpha]^D{}_{25}$ = +12.41° (c 0.0040 g/ml, $CHCl_3$).
ee: 88%

Ethyl (S)-2-(5-chloro-4-oxo-3-(trifluoromethyl)chroman-3-yl)acetate (**2p**)

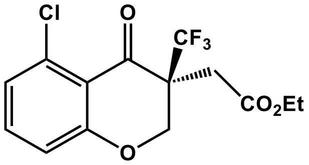

Precatalyst **E** (11 mg; 0.024 mmol), *N,N*-diisopropylethylamine (41.8 µl; 0.24 mmol), ethyl (*E*)-3-((3-chloro-2-formylphenoxy)methyl)-4,4,4-trifluorobut-2-enoate (**1p**) (40 mg; 0.12 mmol) and *o*-xylene (1.2 mL) were used in the reaction carried out correspondingly to the **General Procedure 4**. The reaction was carried out for 48 h. The product was further purified by flash chromatography. The expected product was obtained as yellow oil (3.50 mg) with 9% yield. The enantiomeric excess was determined by HPLC with an Phenomenex Amylose Lux-1 3µm column (n–hexane/i–PrOH = 90:10, flow rate 1.0 mL/min, λ = 254 nm).

^{1}H NMR (700 MHz, $CDCl_3$) δ 7.37 (t, *J* = 8.2 Hz, 1H), 7.11 (dd, *J* = 7.7, 0.9 Hz, 1H), 6.94 (dd, *J* = 8.4, 1.1 Hz, 1H), 4.90 (dd, *J* = 12.3, 1.9 Hz, 1H), 4.70 - 4.68 (m, 1H), 4.12 (q, *J* = 6.9 Hz, 2H), 3.39 (d, *J* = 17.2 Hz, 1H), 2.60 (d, *J* = 17.2 Hz, 1H), 1.22 (t, *J* = 7.1 Hz, 3H).
^{13}C{^{1}H} NMR (101 MHz, $CDCl_3$) δ 184.0, 168.8, 162.8, 135.6, 135.4, 125.7, 125.6, 124.7 (q, *J* = 285.6 Hz), 117.1, 68.1, 61.6, 52.2 (q, *J* = 23.8 Hz), 33.4 (q, *J* = 2.4 Hz), 13.9.

IR-ATR V_{max}: 1737, 1701, 1597, 1566, 1472, 1451, 1320, 1265, 1238, 1200, 1174, 1047, 1025, 875, 800 cm^{-1}.
HRMS (ESI-TOF) m/z: $(M + H)^+$ calcd for $C_{14}H_{13}ClF_3O_4$ 337.0454; found: 337.0452.
$[\alpha]^D_{25}$ = +6.79° (c 0.0032 g/ml, $CHCl_3$).
ee: 86%

Ethyl (S)-2-(5,7-dimethoxy-4-oxo-3-(trifluoromethyl)chroman-3-yl)acetate (**2q**)

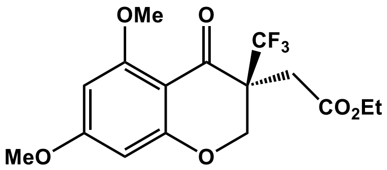

Precatalyst **E** (15.5 mg; 0.033 mmol), *N,N*-diisopropylethylamine (59.2 µl; 0.34 mmol), ethyl (*E*)-4,4,4-trifluoro-3-((2-formyl-3,5-dimethoxyphenoxy)methyl)but-2-enoate (**1q**) (60.0 mg; 0.17 mmol) and *o*-xylene (0.85 mL) were used in the reaction carried out correspondingly to the **General Procedure 4**. The reaction was carried out for 24 h. The product was further purified by flash chromatography. The expected product was obtained as a yellow oil (9.77 mg) with 16% yield. The enantiomeric excess was determined by HPLC with an Phenomenex Amylose Lux-1 3µm column (n–hexane/i–PrOH = 90:10, flow rate 1.0 mL/min, λ = 254 nm).

^{1}H NMR (700 MHz, $CDCl_3$) δ 6.08 (dd, *J* = 11.1, 2.4 Hz, 2H), 4.89 - 4.86 (m, 1H), 4.64 (d, *J* = 12.1 Hz, 1H), 4.14 - 4.09 (m, 2H), 3.88 (s, 3H), 3.83 (s, 3H), 3.32 (d, *J* = 16.8 Hz, 1H), 2.56 (d, *J* = 16.8 Hz, 1H), 1.22 (t, *J* = 7.1 Hz, 3H).
^{13}C{^{1}H} NMR (101 MHz, $CDCl_3$) δ 182.2, 169.1, 166.6, 164.8, 163.1, 125.0 (q, *J* = 285.6 Hz), 105.6, 93.5, 68.0, 61.3, 56.2, 55.7, 51.8 (q, *J* = 23.3 Hz), 33.0 (q, *J* = 2.4 Hz), 14.0.
IR-ATR V_{max}: 1735, 1678, 1605, 1571, 1459, 1427, 1394, 1377, 1216, 1157, 1137, 1104, 1072, 1022, 963, 949, 823, 783 cm^{-1}.
HRMS (ESI-TOF) m/z: $(M + H)^+$ calcd for $C_{16}H_{18}F_3O_6$ 363.1055; found: 363.1058.
$[\alpha]^D_{25}$ = +30.63° (c 0.0016 g/ml, $CHCl_3$).
ee: 95%

Ethyl (S)-2-(6,8-dibromo-4-oxo-3-(trifluoromethyl)chroman-3-yl)acetate (**2r**)

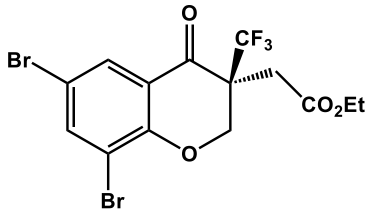

Precatalyst **E** (14.34 mg; 0.031 mmol), *N,N*-diisopropylethylamine (52.3 µl; 0.3 mmol), ethyl (*E*)-3-((2,4-dibromo-6-formylphenoxy)methyl)-4,4,4-trifluorobut-2-enoate (**1r**) (70.6 mg; 0.15 mmol) and *o*-xylene (1.6 mL) were used in the reaction carried out correspondingly to the **General Procedure 4**. The reaction was carried out for 1 h 20 min. The expected product was obtained as a brown oil (66.00 mg) with 96% yield. The enantiomeric excess was determined by HPLC with an Phenomenex Amylose Lux-1 3µm column (n–hexane/i–PrOH = 90:10, flow rate 1.0 mL/min, λ = 254 nm).

^{1}H NMR (700 MHz, CDCl3) δ 8.06 (d, *J* = 2.6 Hz, 1H), 7.92 (d, *J* = 2.6 Hz, 1H), 5.05 - 5.02 (m, 1H), 4.87 (d, *J* = 12.5 Hz, 1H), 4.18 - 4.12 (m, 2H), 3.48 (d, *J* = 17.6 Hz, 1H), 2.61 (d, *J* = 17.2 Hz, 1H), 1.27 (t, *J* = 7.3 Hz, 3H).
^{13}C{^{1}H} NMR (176 MHz, $CDCl_3$) δ 184.2, 168.4, 156.4, 141.4, 129.3, 124.0 (q, *J* = 285.6 Hz), 121.9, 114.4, 112.5, 68.6, 61.4, 50.7 (q, *J* = 24.0 Hz), 32.6, 13.6.
IR-ATR V_{max}: 1728, 1702, 1585, 1469, 1444, 1270, 1235, 1192, 1165, 1127, 1109, 1018, 881, 785, 628 cm^{-1}.
HRMS (ESI-TOF) m/z: $(M + H)^+$ calcd for $C_{14}H_{12}Br_2F_3O_4$ 458.9054; found: 458.9051.
$[\alpha]^D_{25}$ = +39.34° (c 0.028 g/ml, $CHCl_3$).
ee: 92%

Ethyl (S)-2-(6,8-dichloro-4-oxo-3-(trifluoromethyl)chroman-3-yl)acetate (**2s**)

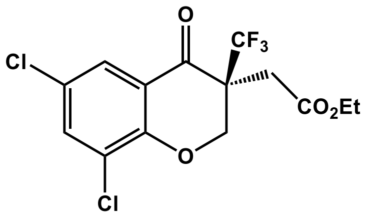

Precatalyst **E** (15.00 mg; 0.16 mmol), *N,N*-diisopropylethylamine (55.7 µl; 0.32 mmol), ethyl (*E*)-3-((2,4-dichloro-6-formylphenoxy)methyl)-4,4,4-trifluorobut-2-enoate (**1s**) (60 mg; 0.16 mmol) and *o*-xylene (1.7 mL) were used in the reaction carried out correspondingly to the **General Procedure 4**. The reaction was carried out for 3 h. The expected product was obtained as a yellow oil (52.00 mg) with 87% yield. The enantiomeric excess was determined by HPLC with an Phenomenex Amylose Lux-1 3µm column (n–hexane/i–PrOH = 90:10, flow rate 1.0 mL/min, λ = 254 nm).

^{1}H NMR (700 MHz, $CDCl_3$) δ 7.83 (dd, *J* = 12.9, 2.6 Hz, 1H), 7.60 (dd, *J* = 12.0, 2.6 Hz, 1H), 5.02 - 4.99 (m, 1H), 4.84 (d, *J* = 12.5 Hz, 1H), 4.15 - 4.09 (m, 2H), 3.45 (d, *J* = 17.2 Hz, 1H), 2.58 (d, *J* = 17.6 Hz, 1H), 1.23 (t, *J* = 7.1 Hz, 3H).
^{13}C{^{1}H} NMR (101 MHz, $CDCl_3$) δ 184.7, 168.8, 155.5, 136.3, 127.5, 125.9, 124.4 (q, *J* = 285.6 Hz), 124.1, 122.0, 69.1, 61.8, 51.2 (q, *J* = 24.1 Hz), 33.0 (q, *J* = 2.4 Hz), 14.0.
IR-ATR V_{max}: 1734, 1704, 1594, 1473, 1441, 1199, 1176, 1132, 1091, 1021, 879, 844, 808, 647 cm^{-1}.
HRMS (ESI-TOF) m/z: $(M + H)^+$ calcd for $C_{14}H_{12}Cl_2F_3O_4$ 371.0065; found: 371.0066.
$[\alpha]^D_{25}$ = +33.48° (c 0.024 g/ml, $CHCl_3$).
ee: 96%

Ethyl (S)-2-(5,7,8-trimethyl-4-oxo-3-(trifluoromethyl)chroman-3-yl)acetate (**2t**)

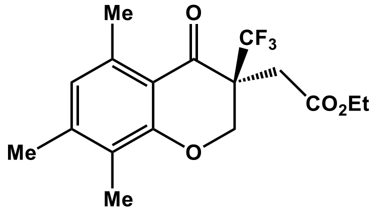

Precatalyst **E** (16.30 mg; 0.035 mmol), *N,N*-diisopropylethylamine (59.23 µl; 0.34 mmol), ethyl (*E*)-4,4,4-trifluoro-3-((2-fluoro-6-formylphenoxy)methyl)but-2-enoate (**1t**) (60 mg; 0.17 mmol) and *o*-xylene (1.7 mL) were used in the reaction carried out correspondingly to the **General Procedure 4**. The reaction was carried out for 20 h. The expected product was obtained as a yellow oil (53.75 mg) with 90% yield. The enantiomeric excess was determined by HPLC with an Phenomenex Amylose Lux-1 3µm column (n–hexane/i–PrOH = 90:10, flow rate 1.0 mL/min, λ = 254 nm).

^{1}H NMR (700 MHz, $CDCl_3$) δ 6.68 (s, 1H), 4.80 (dd, *J* = 12.1, 1.7 Hz, 1H), 4.68 (d, *J* = 12.1 Hz, 1H), 4.10 (q, *J* = 7.3 Hz, 2H), 3.29 (d, *J* = 16.3 Hz, 1H), 2.60 (d, *J* = 16.8 Hz, 1H), 2.57 (s, 3H), 2.26 (s, 3H), 2.11 (s, 3H), 1.20 (t, *J* = 7.1 Hz, 3H).
^{13}C{^{1}H} NMR (101 MHz, $CDCl_3$) δ 187.3, 169.0, 160.1, 145.2, 139.5, 127.1, 125.0 (q, *J* = 285.6 Hz), 123.0, 117.3, 67.9 (d, *J* = 1.6 Hz), 61.3, 52.1 (q, *J* = 23.0 Hz), 33.5 (q, *J* = 2.4 Hz), 22.7, 20.5, 13.9, 11.2.
IR-ATR V_{max}: 1736, 1682, 1606, 1562, 1482, 1447, 1410, 1176, 1107, 1021, 952, 859 cm^{-1}.
HRMS (ESI-TOF) m/z: $(M + H)^+$ calcd for $C_{17}H_{20}F_3O_4$ 345.1314; found: 345.1311.
$[\alpha]^D_{25}$ = +14.65° (c 0.024 g/ml, $CHCl_3$).
ee: 93%

Ethyl (S)-2-(4-oxo-3-(perfluoropropyl)chroman-3-yl)acetate (**2u**)

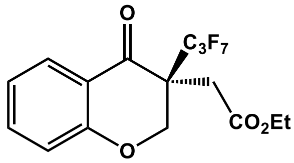

Precatalyst **E** (9.34 mg; 0.02 mmol), *N,N*-diisopropylethylamine (34.84 µl; 0.2 mmol), ethyl (*E*)-4,4,5,5,6,6,6-heptafluoro-3-((2-formylphenoxy)methyl)hex-2-enoate (**1u**) (31.63 mg; 0.1 mmol) were used in the reaction carried out correspondingly to the **General Procedure 4-SF**. The reaction was carried out for 24 h. The product was further purified by flash chromatography. The expected product was obtained as a colorless oil (5 mg) with 12% yield. The enantiomeric excess was determined by HPLC with an Phenomenex Amylose Lux-1 3µm column (n–hexane/i–PrOH = 95:5, flow rate 0.8 mL/min, λ = 254 nm).

^{1}H NMR (700 MHz, $CDCl_3$) δ 7.95 (dd, *J* = 7.7, 1.7 Hz, 1H), 7.54 (ddd, *J* = 8.4, 7.2, 1.7 Hz, 1H), 7.09 (ddd, *J* = 8.0, 7.2, 1.0 Hz, 1H), 7.00 - 6.99 (m, 1H), 5.02 - 5.00 (m, 1H), 4.79 (d, *J* = 12.5 Hz, H), 4.1 (qd, *J* = 7.2, 2.1 Hz, 2H), 3.49 (d, *J* = 17.2 Hz, 1H), 2.65 (dd, *J* = 17.2, 1.3 Hz, 1H), 1.24 (t, *J* = 7.1 Hz, 3H).
^{13}C{^{1}H} NMR (101 MHz, $CDCl_3$) δ 186.2, 169.1, 161.2, 136.5, 127.8, 122.2, 121.3, 117.9, 70.6, 68.2, 61.5, 33.4, 13.9.

IR-ATR V_{max}: 1730, 1698, 1602, 1518, 1483, 1456, 1373, 1341, 1284, 1181, 1115, 1022, 996, 962, 921, 896, 864, 805, 751 cm^{-1}.
HRMS (ESI-TOF) m/z: $(M + H)^+$ calcd for $C_{16}H_{14}F_7O_4$ 403.0780; found: 403.0784.
ee: 88%

Ethyl (S)-2-(4-oxo-3-(perfluorobutyl)chroman-3-yl)acetate (**2v**)

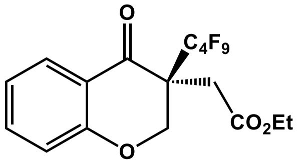

Precatalyst **E** (9.34 mg; 0.02 mmol), *N*,*N*-diisopropylethylamine (34.84 µl; 0.2 mmol), ethyl (*E*)-4,4,5,5,6,6,7,7,7-nonafluoro-3-((2-formylphenoxy)methyl)hept-2-enoate (**1v**) (45.23 mg; 0.1 mmol) were used in the reaction carried out correspondingly to the **General Procedure 4-SF**. The reaction was carried out for 24 h. The product was further purified by flash chromatography. The expected product was obtained as a colorless oil (9.5 mg) with 21% yield. The enantiomeric excess was determined by HPLC with an Phenomenex Amylose Lux-1 3µm column (n–hexane/i–PrOH = 93:7, flow rate 1.0 mL/min, λ = 254 nm).

^{1}H NMR (700 MHz, $CDCl_3$) δ 7.95 (dd, *J* = 8.0, 1.5, 1H), 7.52 (ddd, J = 8.2, 7.3, 1.7 Hz, 1H), 7.08 (ddd, *J* = 8.0, 7.1, 1.3 Hz, 1H), 7.00 - 6.99 (m, 1H), 5.02 - 5.00 (m, 1H), 4.79 (d, *J* = 12.9 Hz, 1H), 4.10 (qd, J = 7.0, 2.2 Hz, 2H), 3.49 (d, *J* = 17.2 Hz, 1H), 2.65 (dd, *J* = 17.2, 1.3 Hz, 1H), 1.22 (t, *J* = 7.1 Hz, 1H).
^{13}C{^{1}H} NMR (101 MHz, $CDCl_3$) δ 186.2, 169.1, 161.2, 136.5, 127.8, 122.2, 121.3, 118.0, 68.2, 61.5, 52.4, 33.4, 13.9.
IR-ATR V_{max}: 1733, 1697, 1606, 1483, 1458, 1375, 1350, 1295, 1233, 1203, 1135, 1095, 1021, 940, 798, 757 cm^{-1}.
HRMS (ESI-TOF) m/z: $(M + H)^+$ calcd for $C_{17}H_{14}F_9O_4$ 453.0748; found: 453.0743.
ee: 88%

Ethyl (S)-2-(4-oxo-3-(perfluorohexyl)chroman-3-yl)acetate (**2w**)

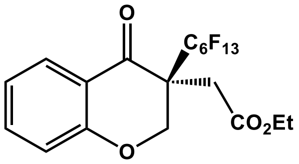

Precatalyst **E** (6.80 mg; 0.015 mmol), *N*,*N*-diisopropylethylamine (25.5 µl; 0.15 mmol), ethyl (*E*)-4,4,5,5,6,6,7,7,8,8,9,9,9-tridecafluoro-3-((2-formylphenoxy)methyl)non-2-enoate (**1w**) (40.5 mg; 0.073 mmol) were used in the reaction carried out correspondingly to the **General Procedure 4-SF**. The reaction was carried out for 24 h. The product was further purified by flash chromatography. The expected product was obtained as a colorless oil (5 mg) with 12% yield.

^{1}H NMR (700 MHz, $CDCl_3$) δ 7.98 (dd, *J* = 7.9, 1.8, 1H), 7.54 (ddd, *J* = 8.3, 6.7, 1.7 Hz, 1H), 7.10 (ddd, *J* = 8.0, 7.2, 1.0 Hz, 1H), 7.02 (dd, *J* = 8.3, 1.0 Hz, 1H), 5.05 - 5.02 (m, 1H), 4.81 (d,

J = 12.7 Hz, 1H), 4.12 (q, *J* = 7.1 Hz, 2H), 3.51 (d, *J* = 17.1 Hz, 1H), 2.67 (dd, *J* = 17.1, 1.3 Hz, 1H), 1.24 (t, *J* = 7.2 Hz, 3H).
^{13}C{^{1}H} NMR (101 MHz, $CDCl_3$) δ 186.2, 169.1, 161.2, 136.5, 127.8, 122.2, 121.3, 117.9, 68.2, 61.5, 52.5 (t, *J* = 19.9 Hz), 33.4 (q, *J* = 4.5 Hz), 13.9.
IR-ATR V_{max}: 1739, 1697, 1609, 1483, 1460, 1236, 1201, 1145, 1046, 1022, 761 cm^{-1}.
HRMS (ESI-TOF) m/z: $(M + H)^+$ calcd for $C_{19}H_{14}F_{13}O_4$ 553.0685; found: 553.0686.

Ethyl (S)-2-(4-oxo-3-(perfluorooctyl)chroman-3-yl)acetate (**2x**)

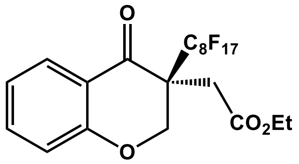

Precatalyst **E** (9.34 mg; 0.02 mmol), *N*,*N*-diisopropylethylamine (34.84 µl; 0.2 mmol), ethyl (E)-4,4,5,5,6,6,7,7,8,8,9,9,10,10,11,11,11-heptadecafluoro-3-((2-formylphenoxy)methyl)undec-2-enoate (**1x**) (65.23 mg; 0.1 mmol) were used in the reaction carried out correspondingly to the **General Procedure 4-SF**. The reaction was carried out for 24 h. The product was further purified by flash chromatography. The expected product was obtained as a white solid (9.92 mg) with 15% yield.

mp: 69.3-74.1°C.
^{1}H NMR (700 MHz, $CDCl_3$) δ 7.95 (dd, *J* = 8.0, 1.5 Hz, 1H), 7.52 (ddd, *J* = 8.5, 7.0, 1.7 Hz, 1H), 7.08 (td, *J* = 7.5, 0.9 Hz, 1H), 7.00 - 6.99 (m, 1H), 5.01 (d, *J* = 12.5 Hz, 1H), 4.79 (d, *J* = 12.5 Hz, 1H), 4.10 (qd, *J* = 7.0, 1.8 Hz, 2H), 3.49 (d, *J* = 17.2 Hz, 1H), 2.65 (d, *J* = 16.8 Hz, 1H), 1.21 (t, *J* = 7.1 Hz, 3H).
^{13}C{^{1}H} NMR (176 MHz, $CDCl_3$) δ 185.8, 168.7, 160.8, 136.1, 127.4, 121.8, 120.9, 117.5, 67.8, 61.1, 52.1 (t, *J* = 19.6 Hz), 33.0, 13.5.
IR-ATR V_{max}: 1737, 1694, 1610, 1483, 1460, 1329, 1293, 1195, 1144, 1048, 1023, 964, 939, 766, 658, 517 cm^{-1}.
HRMS (ESI-TOF) m/z: $(M + H)^+$ calcd for $C_{21}H_{14}F_{17}O_4$ 653.0621; found: 653.0619.

Ethyl 2-(3,3,4,4,5,5,5-heptafluoropentan-2-yl)-3-oxo-2,3-dihydrobenzofuran-2-carboxylate (**4a**)

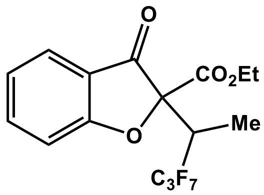

Precatalyst **E** (6.9 mg; 0.015 mmol), *N*,*N*-diisopropylethylamine (26 µl; 0.149 mmol), ethyl (*Z*)-4,4,5,5,6,6,6-heptafluoro-2-(2-formylphenoxy)-3-methylhex-2-enoate (**3a**) (30 mg; 0.075 mmol) and *o*-xylene (0.75 mL) were used in the reaction carried out correspondingly to the **General Procedure 4**. The reaction was carried out for 26 h. The product was further purified by flash chromatography. The expected product was obtained as a yellow oil (27.00 mg) with 90% yield. The enantiomeric excess was determined by HPLC with an Phenomenex Cellulose Lux-1 3µm column (n–hexane/i–PrOH = 99:1, flow rate 1.0 mL/min, λ = 254 nm).

^{1}H NMR (400 MHz, $CDCl_3$) δ 7.71 - 7.67 (m, 2H), 7.31 - 7.28 (m, 1H), 7.19 - 7.15 (m, 1H), 4.29 (q, J = 7.1 Hz, 2H), 3.88 - 3.76 (m, 1H), 1.46 (dd, J = 7.1, 0.9 Hz, 3H), 1.30 (t, J = 7.2 Hz, 3H).
^{13}C{^{1}H} NMR (101 MHz, $CDCl_3$) δ 193.0 (d, J = 2.4 Hz), 171.5, 164.4, 138.5, 125.1, 123.0, 119.3 (d, J = 3.2 Hz), 113.5, 91.6 (d, J = 4.0 Hz), 63.5, 39.7 (dd, J = 20.7, 17.1 Hz), 13.9, 10.2.
IR-ATR V_{max}: 1755, 1735, 1613, 1477, 1464, 1347, 1299, 1277, 1219, 1194, 1181, 1112, 1032, 916, 756 cm^{-1}.
HRMS (ESI-TOF) m/z: $(M + H)^+$ calcd for $C_{16}H_{14}F_7O_4$ 403.0780; found: 403.0779.
$[\alpha]^D_{25}$ = +1.94° (c 0.00720 g/ml, $CHCl_3$).
ee: 66%; ***dr*** >20:1.

Ethyl 2-(3,3,4,4,5,5,6,6,6-nonafluorohexan-2-yl)-3-oxo-2,3-dihydrobenzofuran-2-carboxylate (**4b**)

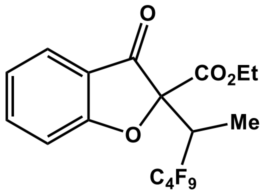

Precatalyst **E** (6.2 mg; 0.013 mmol), *N,N*-diisopropylethylamine (23 μl; 0.133 mmol), ethyl (*Z*)-4,4,5,5,6,6,7,7,7-nonafluoro-2-(2-formylphenoxy)-3-methylhept-2-enoate (**3b**) (30 mg; 0.066 mmol) and *o*-xylene (0.66 mL) were used in the reaction carried out correspondingly to the **General Procedure 4**. The reaction was carried out for 19 h. The product was further purified by flash chromatography. The expected product was obtained as an orange oil (24.00 mg) with 81% yield. The enantiomeric excess was determined by HPLC with an Phenomenex Cellulose Lux-1 3μm column (n–hexane/i–PrOH = 90:10, flow rate 1.0 mL/min, λ = 254 nm).

^{1}H NMR (700 MHz, $CDCl_3$) δ 7.68 (td, J = 7.7, 1.3 Hz, 2H), 7.29 - 7.27 (m, 1H), 7.17 - 7.15 (m, 1H), 4.27 (q, J = 7.3 Hz, 2H), 3.85 - 3.78 (m, 1H), 1.44 (d, J = 6.9 Hz, 3H), 1.28 (t, J = 7.1 Hz, 3H).
^{13}C{^{1}H} NMR (101 MHz, $CDCl_3$) δ 192.9 (d, J = 3.2 Hz), 171.5, 164.4, 138.5, 125.1, 123.0, 119.3 (d, J = 2.4 Hz), 113.5, 91.6 (d, J = 4.0 Hz), 63.5, 39.8 (dd, J = 20.7, 17.1 Hz), 13.9, 10.2.
IR-ATR V_{max}: 1755, 1736, 1613, 1477, 1464, 1299, 1213, 1133, 1019, 977, 857, 838, 756 cm^{-1}.
HRMS (ESI-TOF) m/z: $(M + H)^+$ calcd for $C_{17}H_{14}F_9O_4$ 453.0748; found: 453.0753.
$[\alpha]^D_{25}$ = -8.00° (c 0.00812 g/ml, $CHCl_3$).
ee: 70%; ***dr*** >20:1.

Ethyl 3-oxo-2-(3,3,4,4,5,5,6,6,7,7,8,8,8-tridecafluorooctan-2-yl)-2,3-dihydrobenzofuran-2-carboxylate (**4c**)

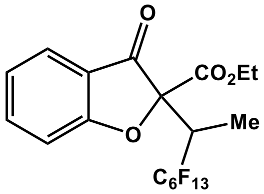

Precatalyst **E** (5.07 mg; 0.011 mmol), *N,N*-diisopropylethylamine (19 µl; 0.11 mmol), ethyl (*Z*)-4,4,5,5,6,6,7,7,8,8,9,9,9-tridecafluoro-2-(2-formylphenoxy)-3-methylnon-2-enoate (**3c**) (30 mg; 0.054 mmol) and *o*-xylene (0.55 mL) were used in the reaction carried out correspondingly to the **General Procedure 4**. The reaction was carried out for 3 h. The product was further purified by flash chromatography. The expected product was obtained as a brown oil (15.37 mg) with 51% yield. The enantiomeric excess was determined by HPLC with an Phenomenex Amylose Lux-1 3µm column (n–hexane/i–PrOH = 90:10, flow rate 1.0 mL/min, λ = 254 nm).

^{1}H NMR (700 MHz, $CDCl_3$) δ 7.68 - 7.67 (m, 2H), 7.28 (d, *J* = 7.7 Hz, 1H), 7.17 - 7.14 (m, 1H), 4.27 (q, *J* = 7.2 Hz, 2H), 3.85 - 3.78 (m, 1H), 1.44 (d, *J* = 7.3 Hz, 3H), 1.28 (t, *J* = 7.1 Hz, 3H).
^{13}C{^{1}H} NMR (101 MHz, $CDCl_3$) δ 192.9 (d, *J* = 2.4 Hz), 171.4, 164.4, 138.4, 125.1, 123.0, 119.3 (d, *J* = 3.2 Hz), 113.5, 91.6 (d, *J* = 4.0 Hz), 63.5, 39.9 (dd, *J* = 20.7, 16.7 Hz), 13.9, 10.2.
IR-ATR V_{max}: 1754, 1731, 1613, 1465, 1229, 1194, 1142, 1027, 977, 756, 708 cm^{-1}.
HRMS (ESI-TOF) m/z: $(M + H)^+$ calcd for $C_{19}H_{14}F_{13}O_4$ 553.0685; found: 553.0683.
$[\alpha]^{D}_{25}$ = -1.70° (c 0.0124 g/ml, $CHCl_3$).
ee: 65%; ***dr*** >20:1.

Ethyl 2-(3,3,4,4,5,5,6,6,7,7,8,8,9,9,10,10,10-heptadecafluorodecan-2-yl)-3-oxo-2,3-dihydrobenzofuran-2-carboxylate (**4d**)

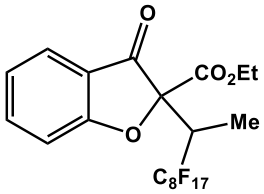

Precatalyst **E** (2.86 mg; 0.0061 mmol), *N,N*-diisopropylethylamine (11 µl; 0.061 mmol), ethyl (*Z*)-4,4,5,5,6,6,7,7,8,8,9,9,10,10,11,11,11-heptadecafluoro-2-(2-formylphenoxy)-3-methylundec-2-enoate (**3d**) (20 mg; 0.031 mmol) and *o*-xylene (0.31 mL) were used in the reaction carried out correspondingly to the **General Procedure 4**. The reaction was carried out for 3 h. The product was further purified by flash chromatography. The expected product was obtained as a yellow oil (15.98 mg) with 79% yield. The enantiomeric excess was determined by HPLC with an Phenomenex Cellulose Lux-1 3µm column (n–hexane/i–PrOH = 90:10, flow rate 1.0 mL/min, λ = 254 nm).

^{1}H NMR (400 MHz, $CDCl_3$) δ 7.72 - 7.68 (m, 2H), 7.30 (dd, *J* = 9.1, 0.9 Hz, 1H), 7.19 - 7.16 (m, 1H), 4.29 (q, *J* = 7.1 Hz, 2H), 3.89 - 3.78 (m, 1H), 1.47 - 1.45 (m, 3H), 1.30 (t, *J* = 7.2 Hz, 3H).
^{13}C{^{1}H} NMR (101 MHz, $CDCl_3$) δ 192.9 (d, *J* = 3.2 Hz), 171.4, 164.4, 138.4, 125.1, 123.0, 119.3 (d, *J* = 3.2 Hz), 113.5, 91.6 (d, *J* = 4.8 Hz), 63.5, 39.9 (dd, *J* = 20.7, 17.5 Hz), 13.9, 10.2.
IR-ATR V_{max}: 1750, 1729, 1614, 1466, 1237, 1200, 1165, 1145, 1036, 956, 760, 706, 663, 580, 553, 514 cm^{-1}.
HRMS (ESI-TOF) m/z: $(M + H)^+$ calcd for $C_{21}H_{14}F_{17}O_4$ 653.0621; found: 653.0618.
$[\alpha]^{D}_{25}$ = +3.88° (c 0.00644 g/ml, $CHCl_3$).
ee: 52%; ***dr*** >20:1.

(S)-2-(7-fluoro-4-oxo-3-(trifluoromethyl)chroman-3-yl)acetic acid (**7**)

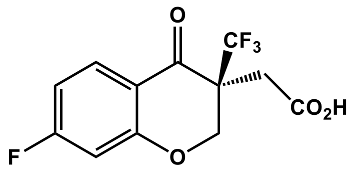

^{1}H NMR (400 MHz, $CDCl_3$) δ 7.97 (dd, *J* = 8.8, 6.5 Hz, 1H), 6.80 (ddd, *J* = 8.8, 8.2, 2.4 Hz, 1H), 6.69 (dd, *J* = 9.7, 2.4 Hz, 1H), 4.97 - 4.92 (m, 1H), 4.73 (d, *J* = 12.3 Hz, 1H), 3.45 (d, *J* = 17.6 Hz, 1H), 2.60 (d, *J* = 17.6 Hz, 1H).
^{13}C{^{1}H} NMR (101 MHz, $CDCl_3$) δ 184.4, 173.7, 168.0 (d, *J* = 258.9 Hz), 163.1 (d, *J* = 13.9 Hz), 130.7 (d, *J* = 11.6 Hz), 124.5 (q, *J* = 258.7 Hz), 117.2, 110.8 (d, *J* = 23.1 Hz), 105.0 (d, *J* = 24.7 Hz), 68.6, 51.1 (q, *J* = 23.9 Hz), 32.2 (m).
IR-ATR V_{max}: 2987, 1735, 1696, 1614, 1589, 1440, 1410, 1376, 1281, 1243, 1228, 1176, 1147, 1096, 1069, 1039, 954, 854, 822, 767, 734, 629, 575, 527, 482 cm^{-1}.
HRMS (ESI-TOF) m/z: $(M + H)^+$ calcd for $C_{21}H_9F_4O_4$ 293.0437 found: 293.0433.

3. X-Ray Crystallography Data

Good-quality single-crystal of investigated compound (**7**) was selected for the X-ray diffraction experiment at T = 100(2) K. The crystal was mounted with paratone-N oil to the MiTeGen micromount (Fig. S1). Diffraction data were collected on the Agilent Technologies SuperNova Dual Source with the Cu$K\alpha$ radiation (λ = 1.54184 Å). The lattice parameters were obtained by least-squares fit to the optimized setting angles of the reflections collected by using the CrysAlis CCD software[1]. Data were reduced using the CrysAlis RED program[1]. The multi-scan numerical absorption correction implemented in SCALE3 ABSPACK scaling algorithm were applied[1]. The structural determination procedure was carried out using the SHELX package[2].

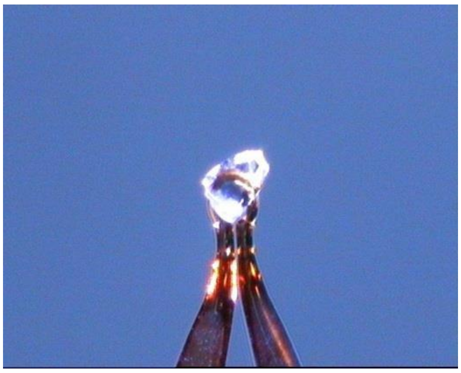

Figure S1. Single-crystal of investigated compound selected for the analysis.

The structure was solved with direct methods, and then successive least-squares refinements were carried out based on full-matrix least-squares on F^2 using the SHELXL program[2]. All H-atoms bound to C-atoms were positioned geometrically with the C−H bond length equal to 0.93 and 0.97 Å for the aromatic and methylene hydrogen atoms, respectively, and constrained to ride on their parent atoms with U_{iso}(H) = 1.2U_{eq}(C). The hydroxyl H-atom was located on a Fourier difference map and refined as riding with U_{iso}(H) = 1.5U_{eq}(O). In case of the O–H bond the DFIX 0.82 restraint was applied. Few distinct peaks on the difference Fourier map were indicating the presence of a disordered solvent molecules in the crystal lattice. All attempts to model a disordered solvents used for crystallization failed. Therefore, the solvent contribution has been removed applying the appropriate MASK procedure in Olex2[3] program. Calculated void volume was approximately 347.1 Å^3 (23.4%) occupied by 129.4 electrons per unit cell. The figures for this report were prepared using Olex2[3] and Mercury[4] programs.

Investigated compound crystallizes in the monoclinic *I*2 space group with one molecule of the compound in the asymmetric unit of the crystal lattice (Fig. S2). The configuration of the asymmetric C3 atom is *S*. The crystallographic data are summarized in Table S1. The arrangement of molecules of investigated compound in the crystal is presented in Fig. S3.

Table S1. Crystal data and structure refinement for investigated compound.

**Identification code**	
Empirical formula	$C_{12}H_8F_4O_4$
Formula weight	292.18
Temperature/K	100(2)
Crystal system	monoclinic
Space group	*I*2
a/Å	7.36090(17)
b/Å	6.79957(17)
c/Å	29.7048(7)
α/°	90
β/°	95.063(2)
γ/°	90
Volume/Å^3	1480.95(6)
Z	4
ρcalc g/cm3	1.310
μ/mm^{-1}	1.159
F(000)	592.0
Crystal size/mm^3	0.19 × 0.15 × 0.08
Radiation	CuK*α* (*λ* = 1.54184)
2*Θ* range for data collection/°	5.974 to 134.154
Index ranges	$-8 \leq h \leq 8$, $-8 \leq k \leq 8$, $-35 \leq l \leq 35$
Reflections collected	10197
Independent reflections	2651 [R_{int} = 0.0175, R_{sigma} = 0.0136]
Data/restraints/parameters	2651/2/184
Goodness-of-fit on F^2	1.098
Final *R* indexes [*I*>=2*σ* (*I*)]	R_1 = 0.0394, wR_2 = 0.1178
Final *R* indexes [all data]	R_1 = 0.0408, wR_2 = 0.1206
Largest diff. peak/hole / e Å^{-3}	0.35/-0.21
Flack parameter	0.05(6)

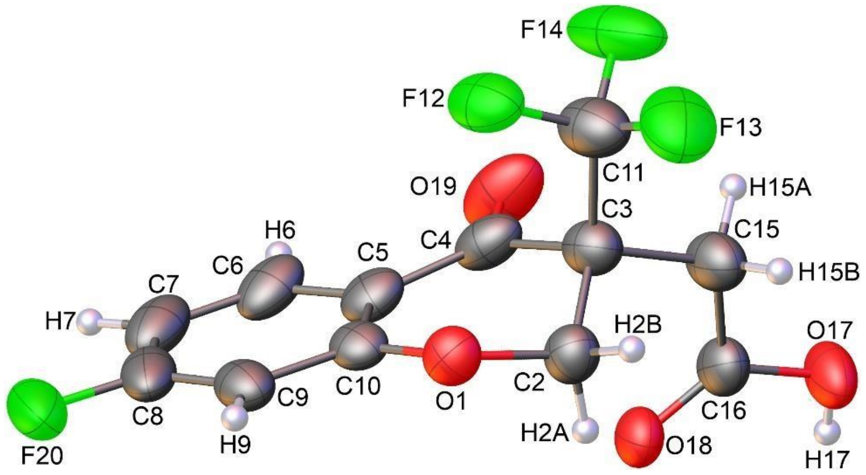

Figure S2. Asymmetric unit of the crystal lattice investigated compound showing the atom labelling scheme. Displacement ellipsoids are drawn at the 50% probability level.

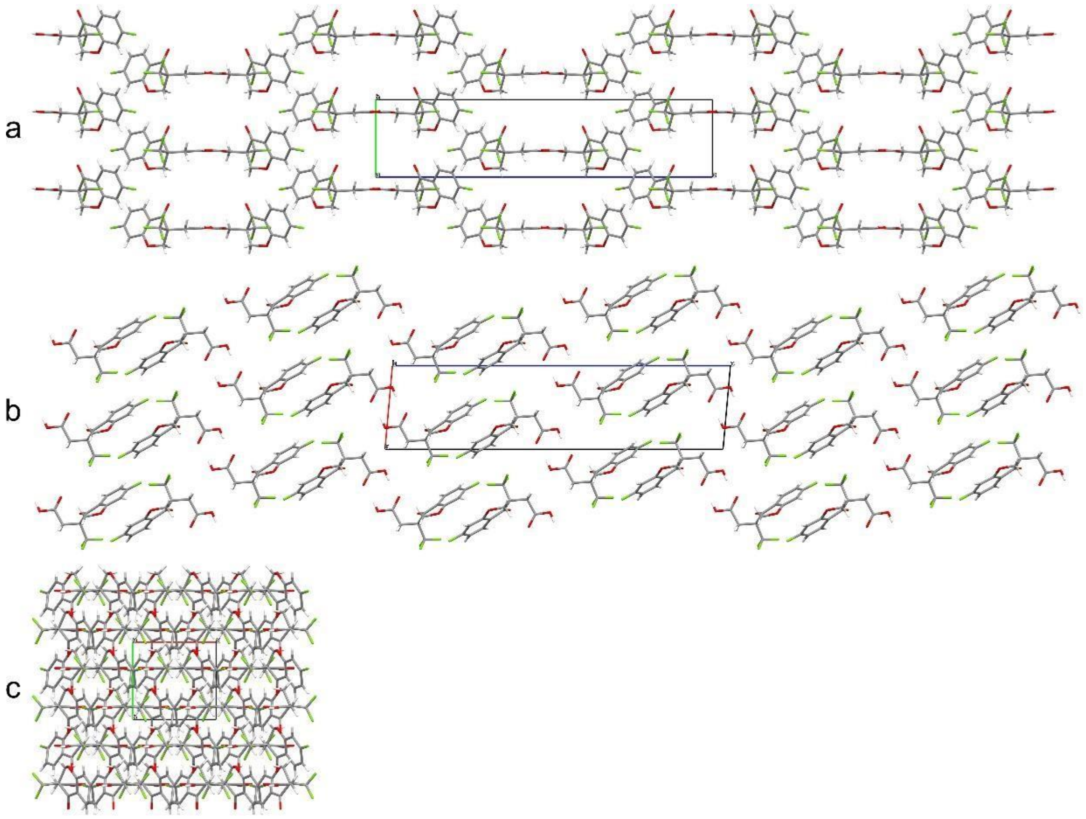

Figure S3. Supramolecular architecture of investigated compound in the crystal, viewed along *a*, *b* and *c*-direction.

References

1. *CrysAlis CCD and CrysAlis RE*D; Oxford Diffraction Ltd: Yarnton, 2008.
2. G.M. Sheldrick, *Acta Crystallogr. Sect. A*, 2008, **64**, 112−122.
3. O.V. Dolomanov, L.J. Bourhis, R.J. Gildea, J.A.K. Howard, H. Puschmann, *J. Appl. Cryst.*, 2009, **42**, 339–341.
4. C.F. Macrae, P.R. Edgington, P. McCabe, E. Pidcock, G.P. Shields, R. Taylor, M. Towler, J. van de Streek, *J. Appl. Cryst.*, 2006, **39**, 453–457.

4. NMR Spectra

^{1}H NMR 700 MHz $CDCl_3$

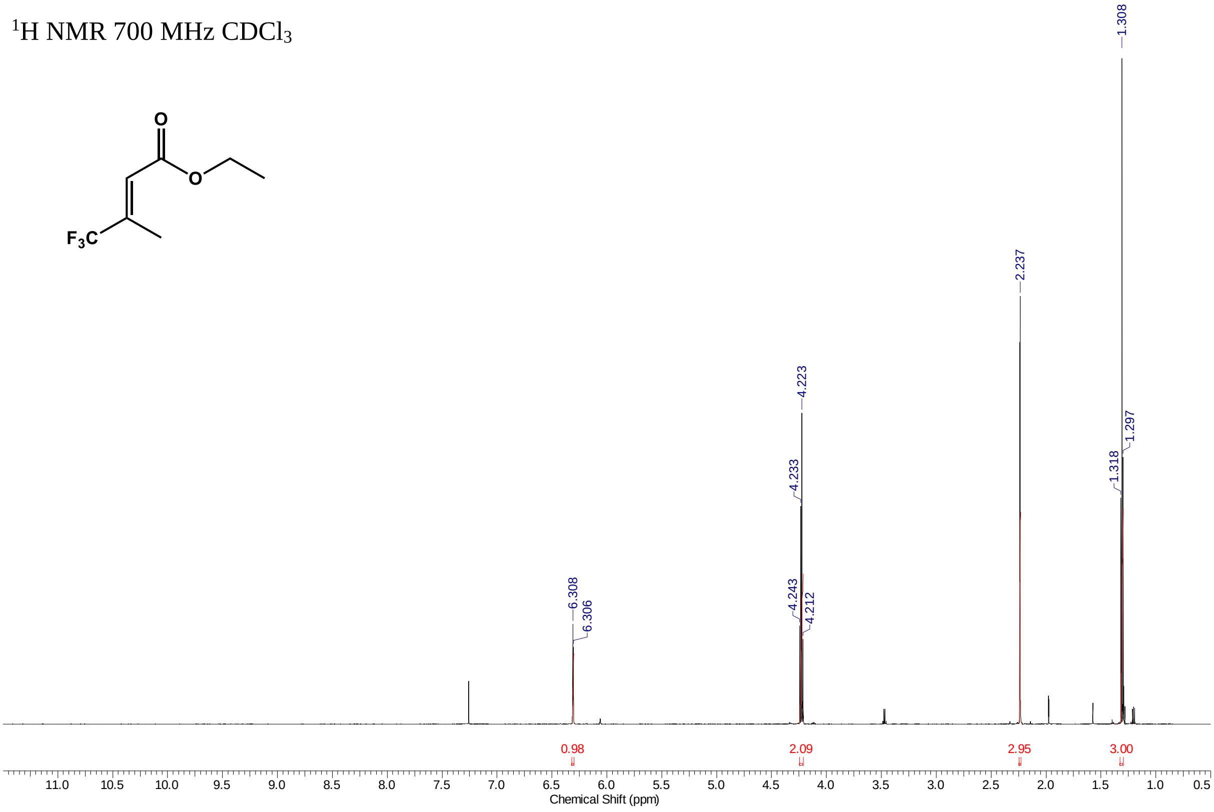

$^{13}C\{^1H\}$ NMR 101 MHz $CDCl_3$

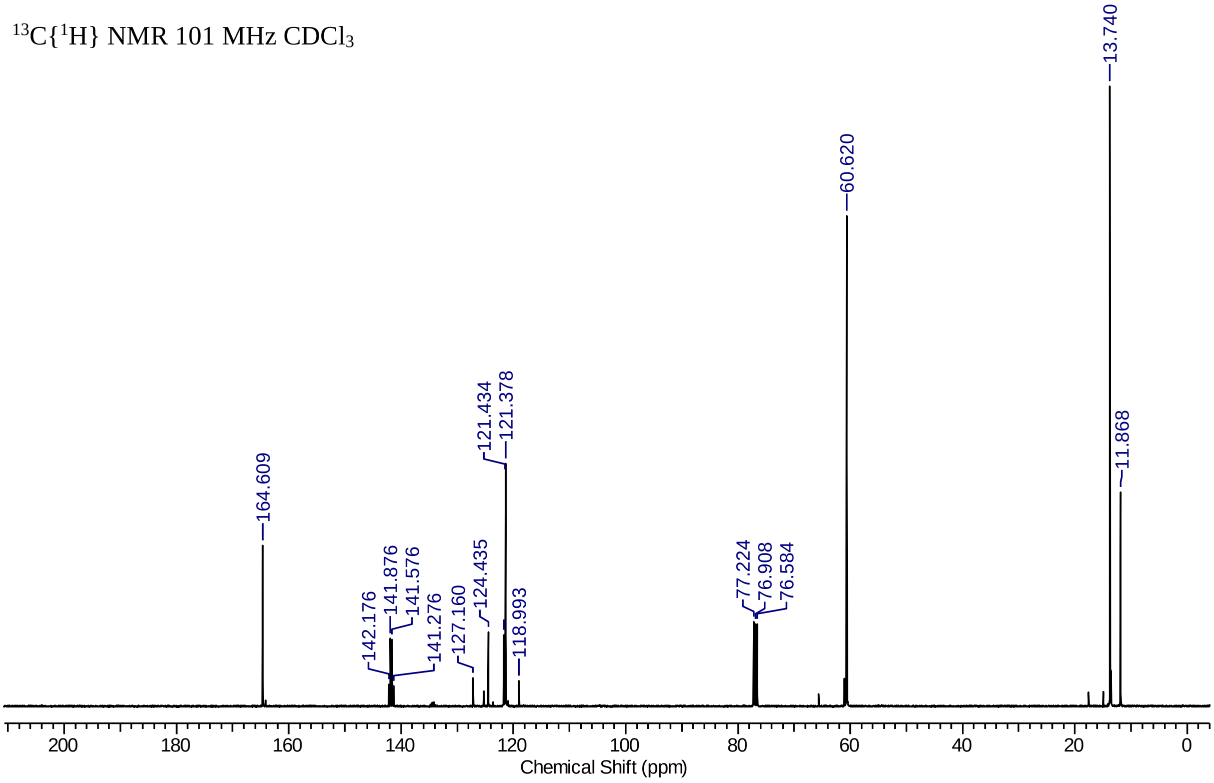

Figure S4. ^{1}H and ^{13}C NMR spectra of compound **5a**.

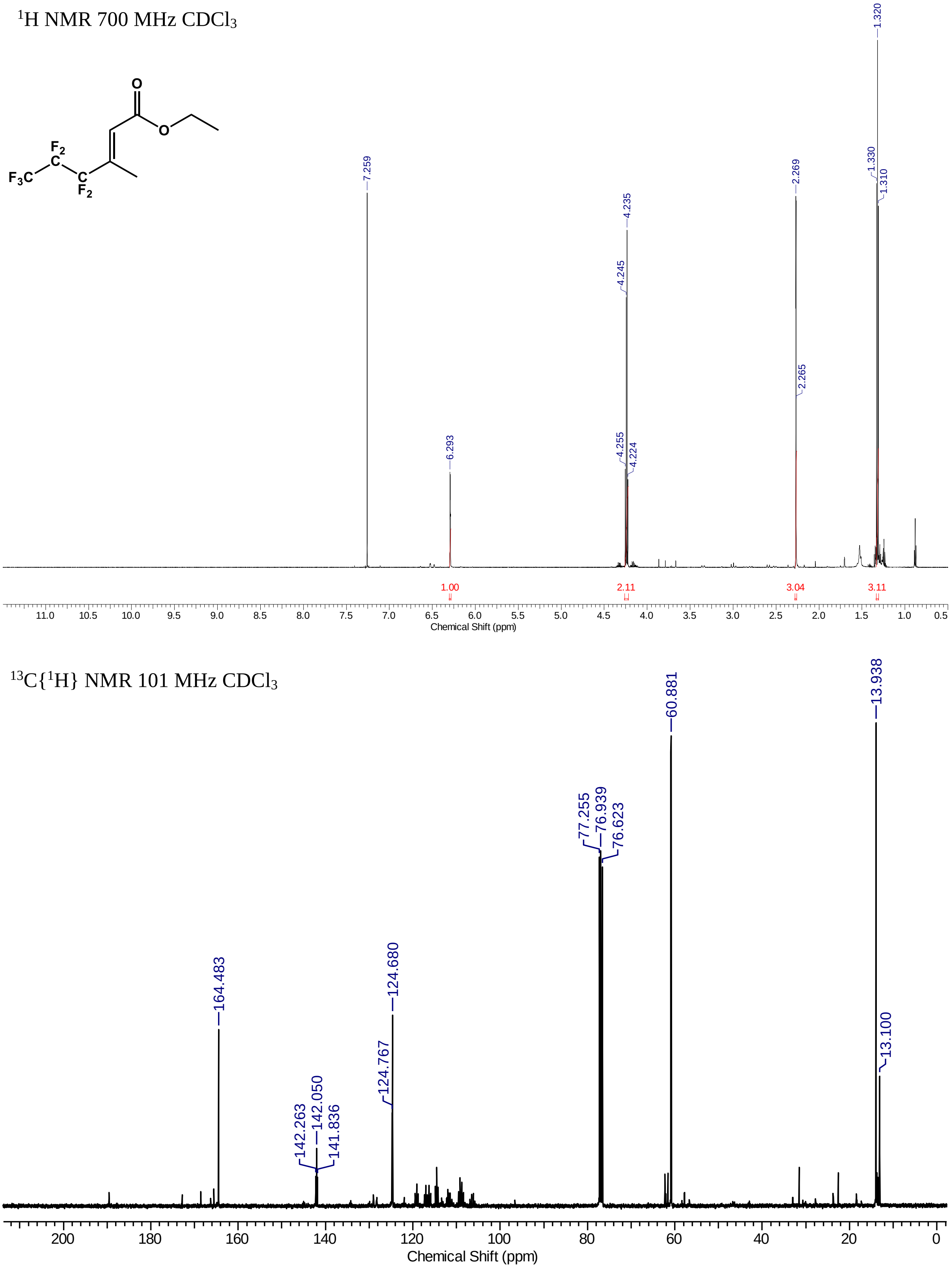

Figure S5. ^{1}H and ^{13}C NMR spectra of compound **5b**.

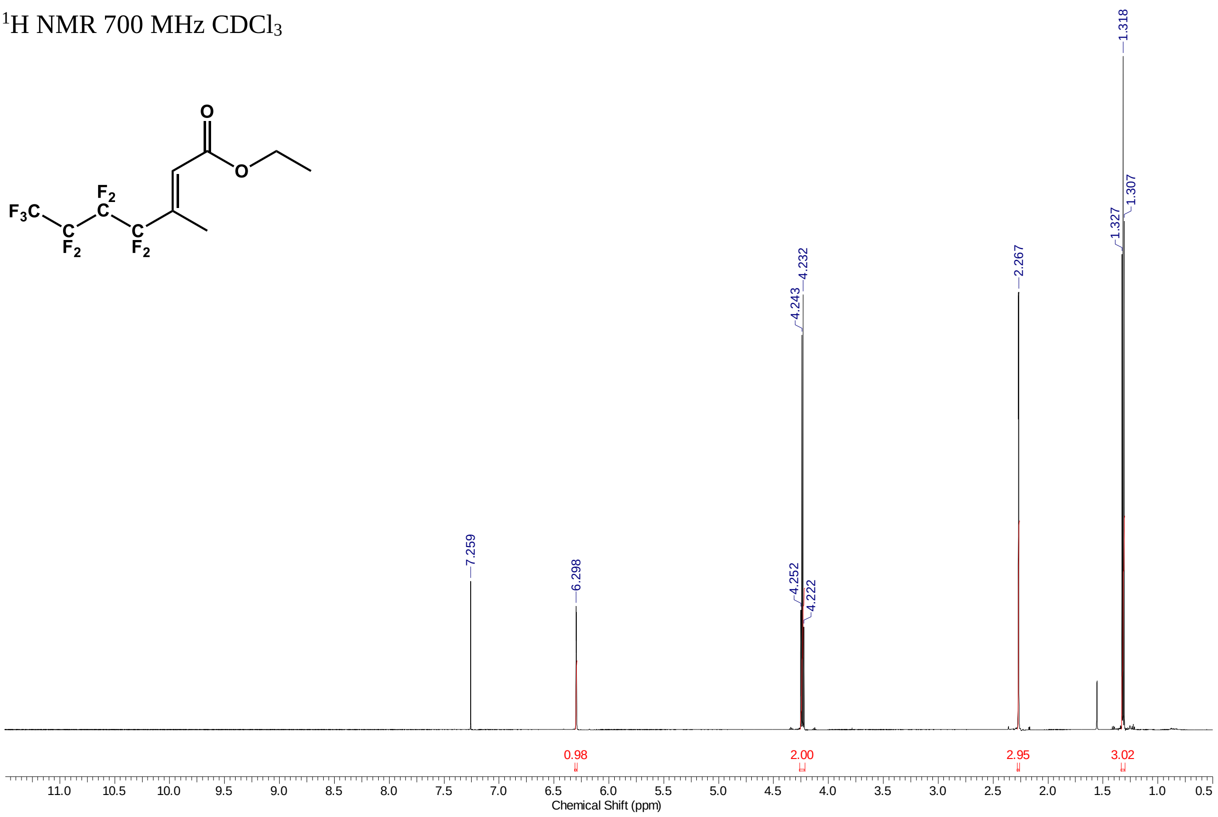

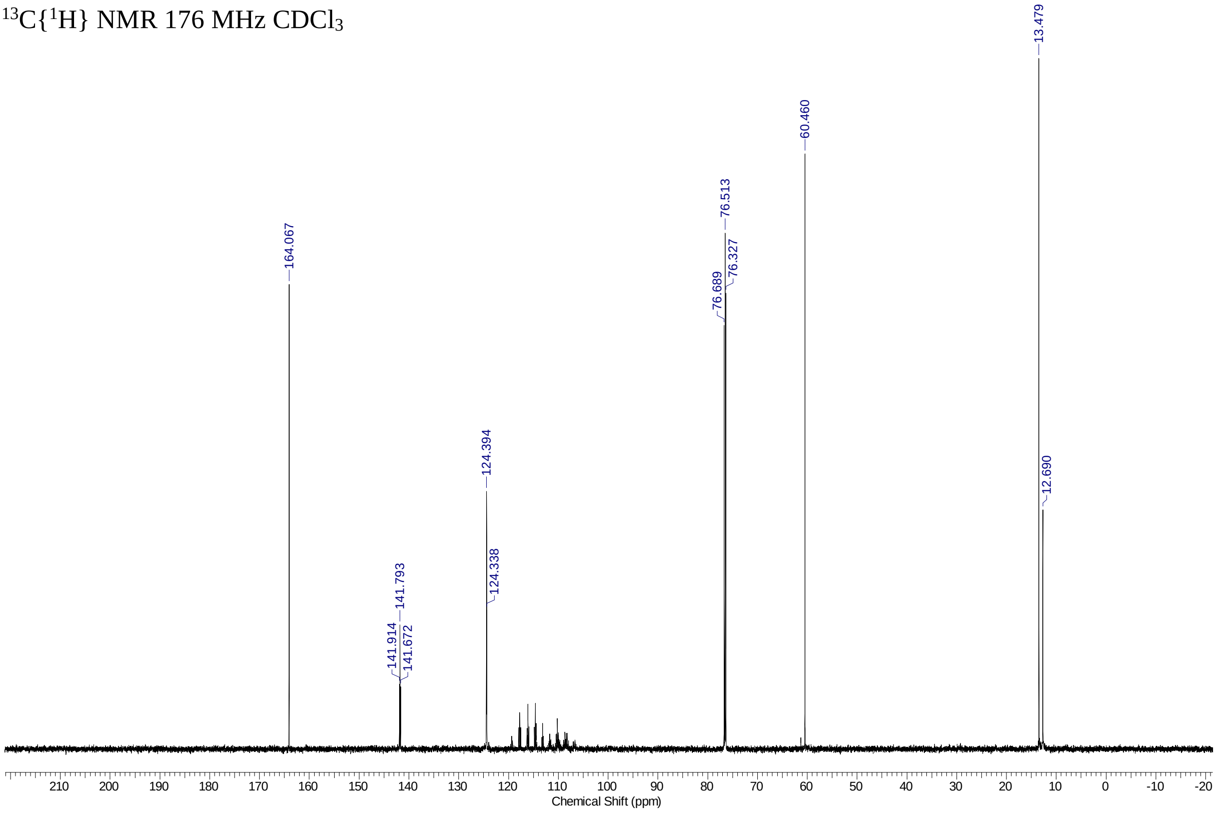

Figure S6. ^{1}H and ^{13}C NMR spectra of compound **5c**.

^{1}H NMR 400 MHz $CDCl_3$

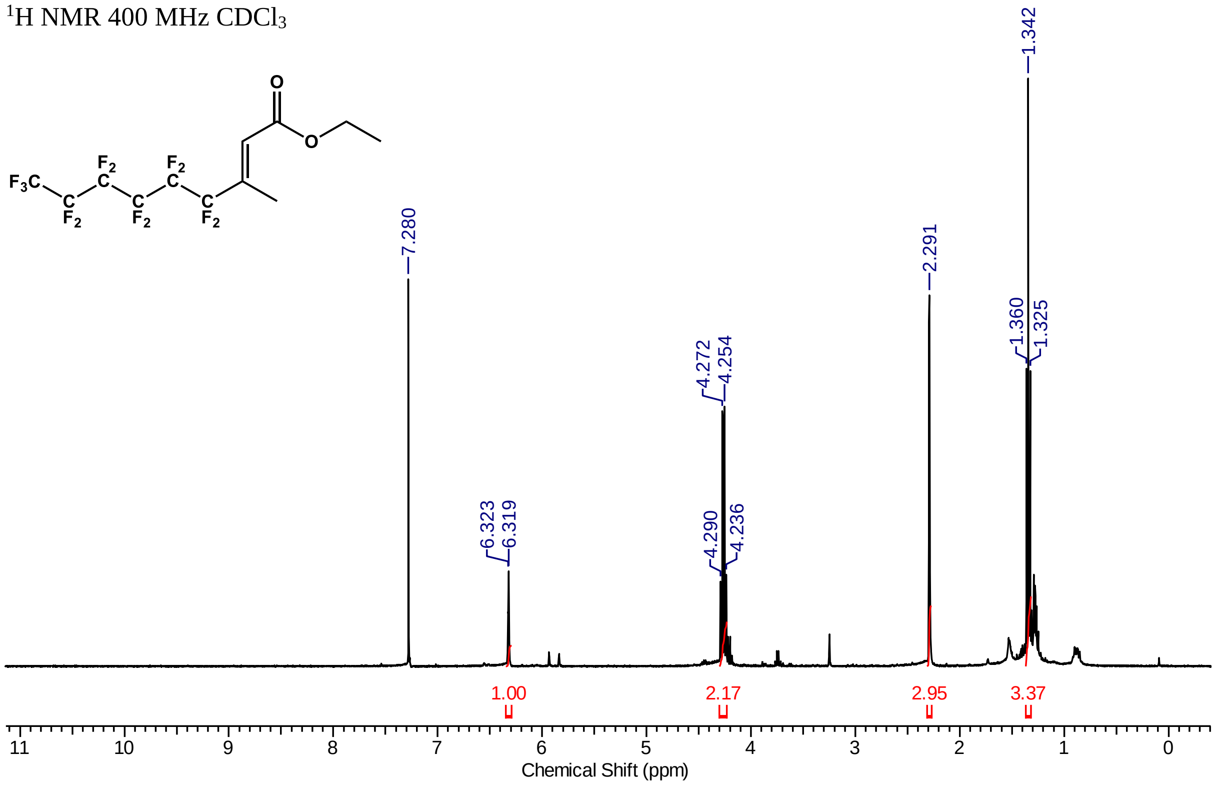

^{13}C{^{1}H} NMR 176 MHz $CDCl_3$

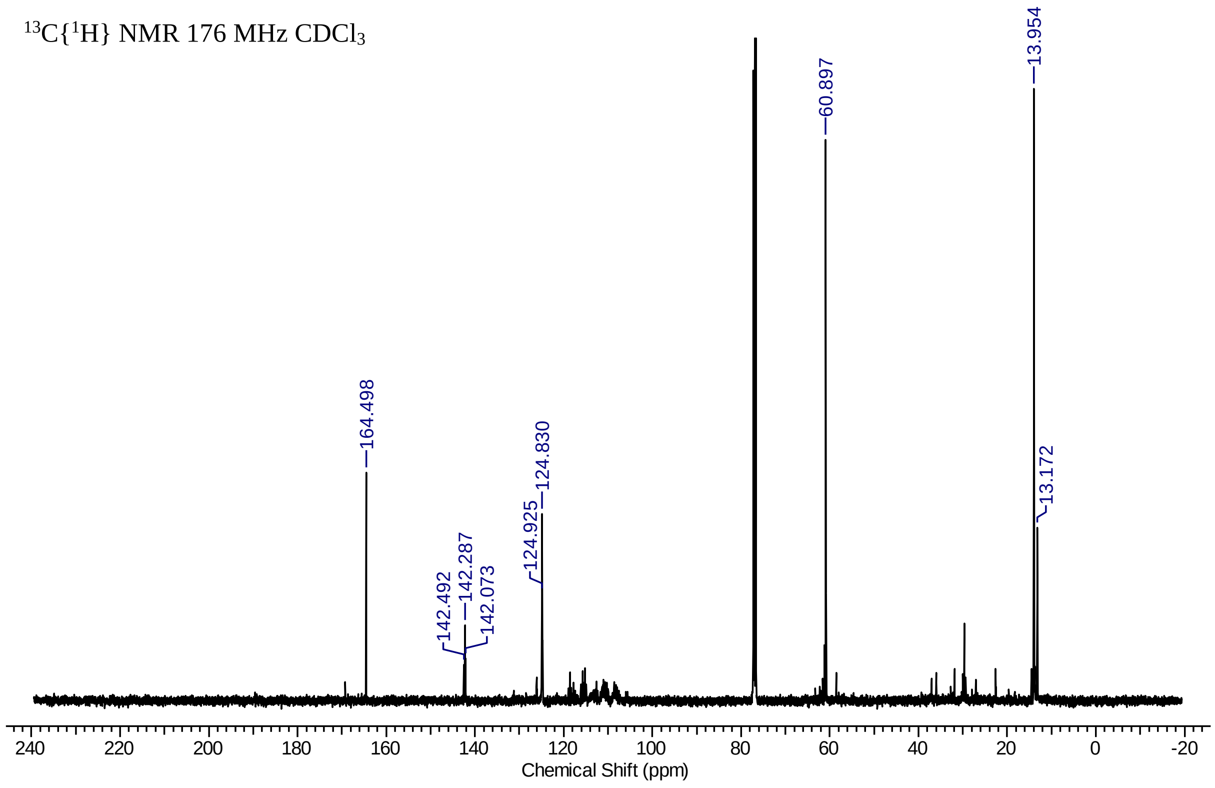

Figure S7. ^{1}H and ^{13}C NMR spectra of compound **5d**.

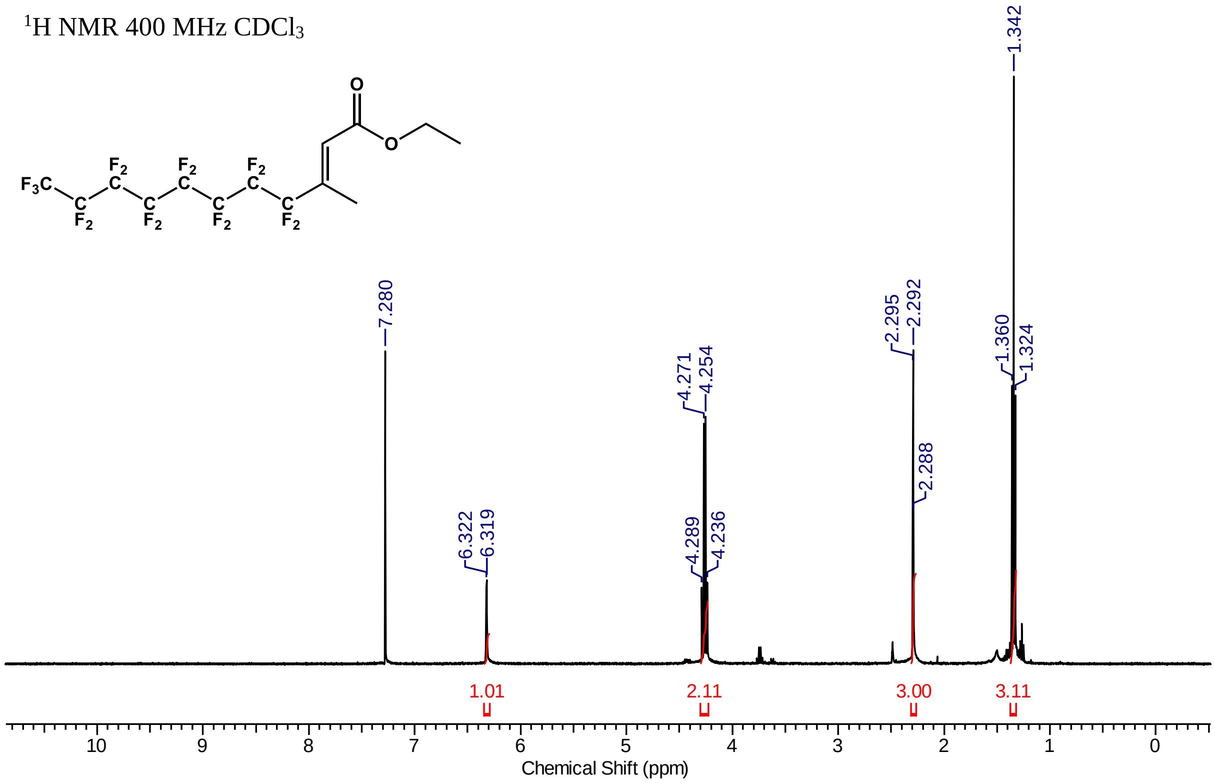

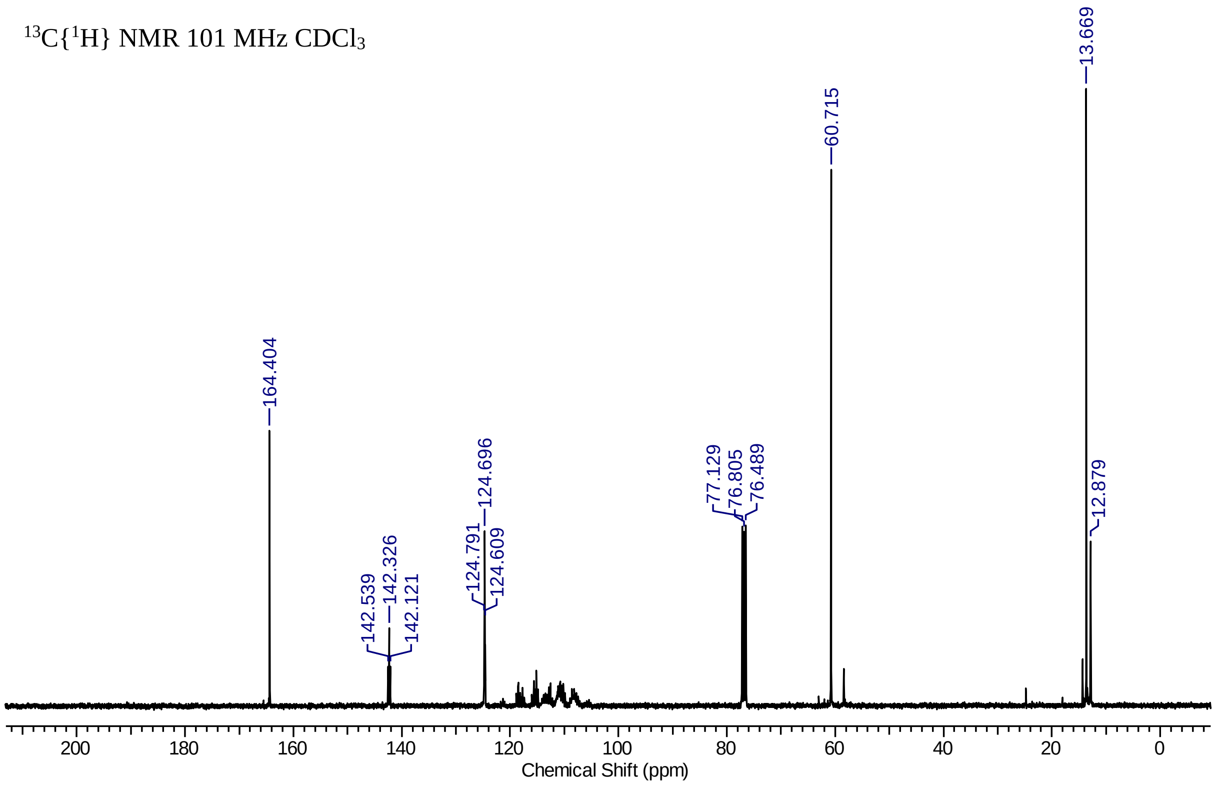

Figure S8. ^{1}H and ^{13}C NMR spectra of compound **5e**.

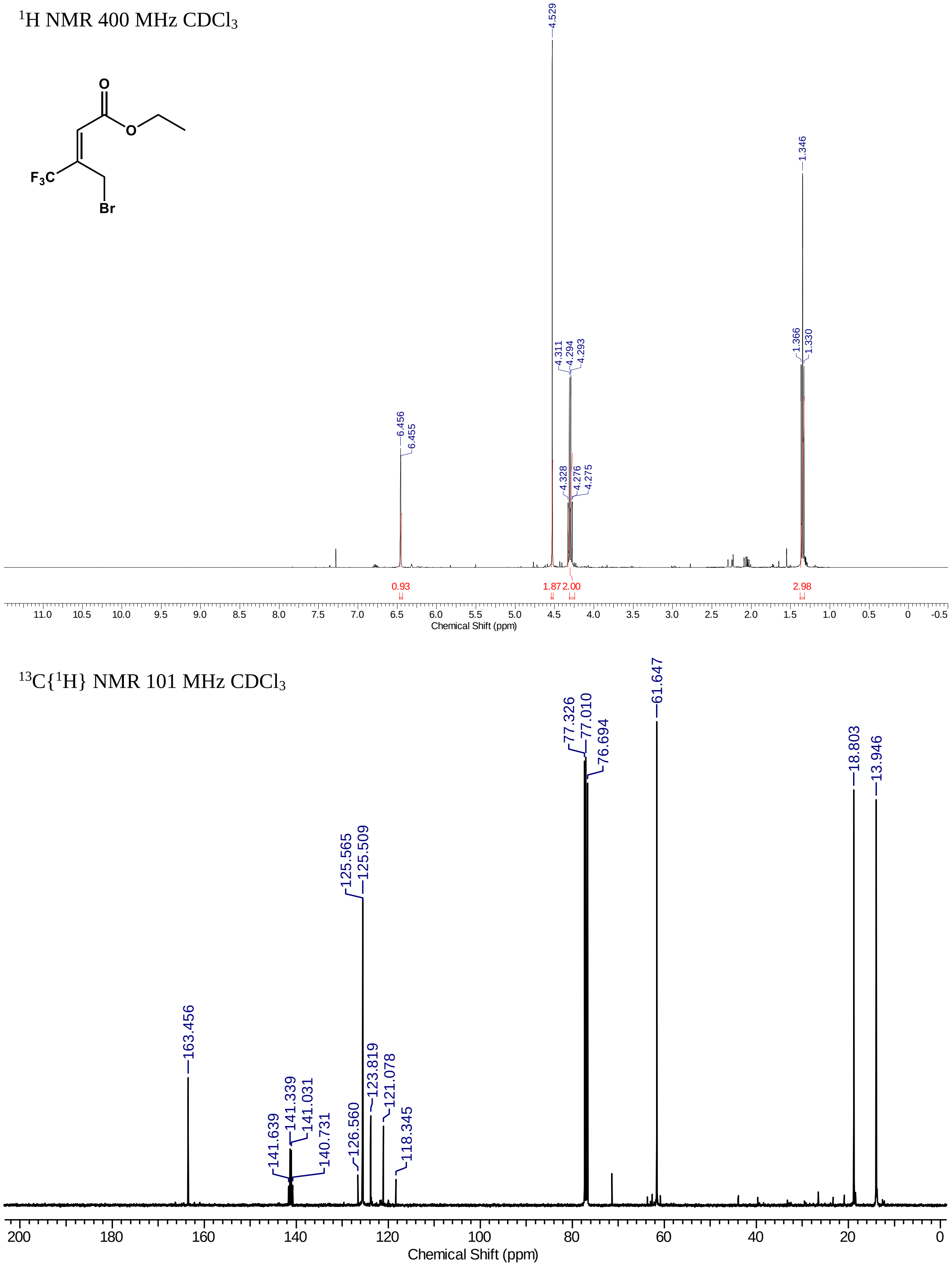

Figure S9. ^{1}H and ^{13}C NMR spectra of compound **6a**.

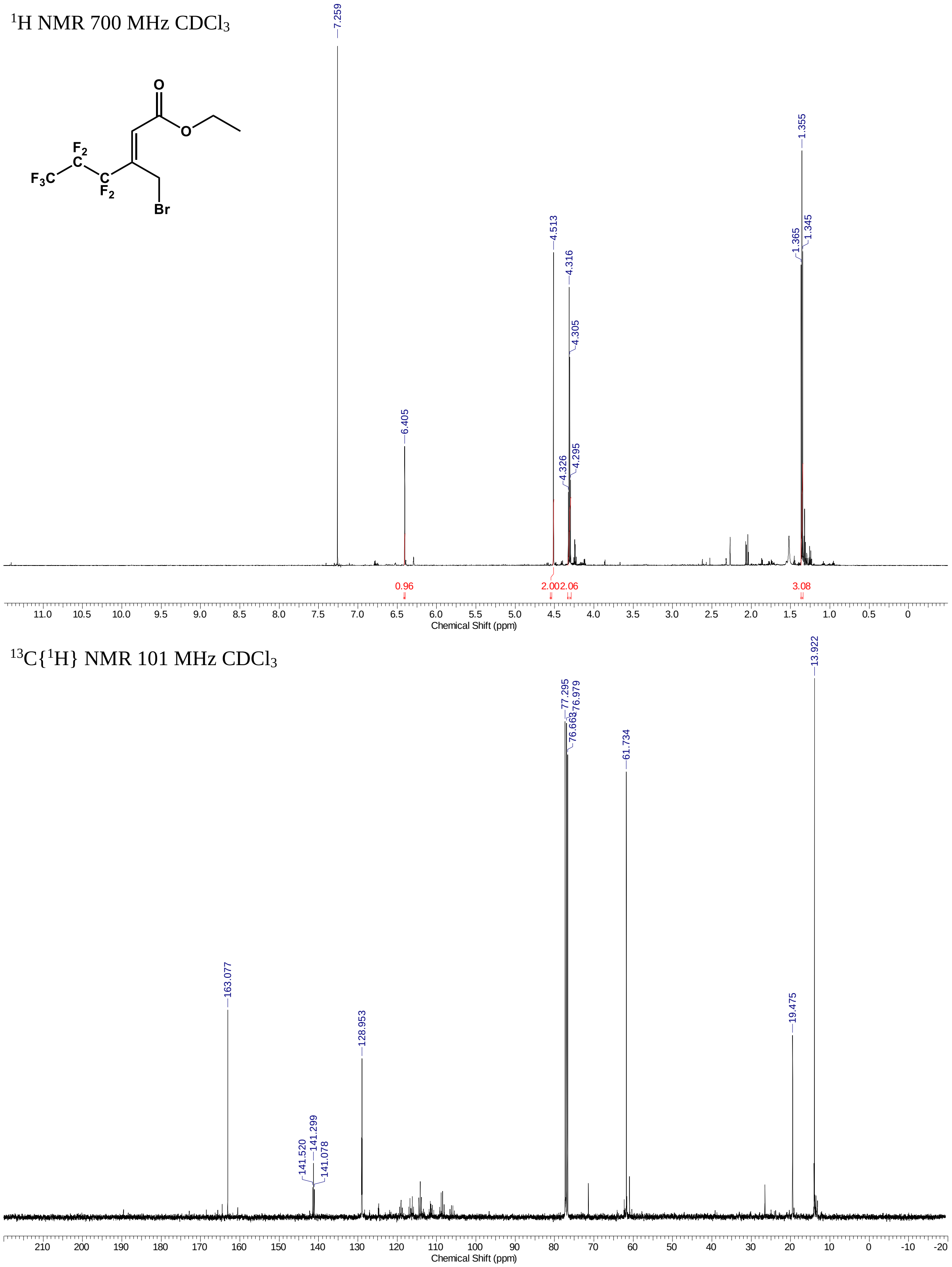

Figure S10. ^{1}H and ^{13}C NMR spectra of compound **6b**.

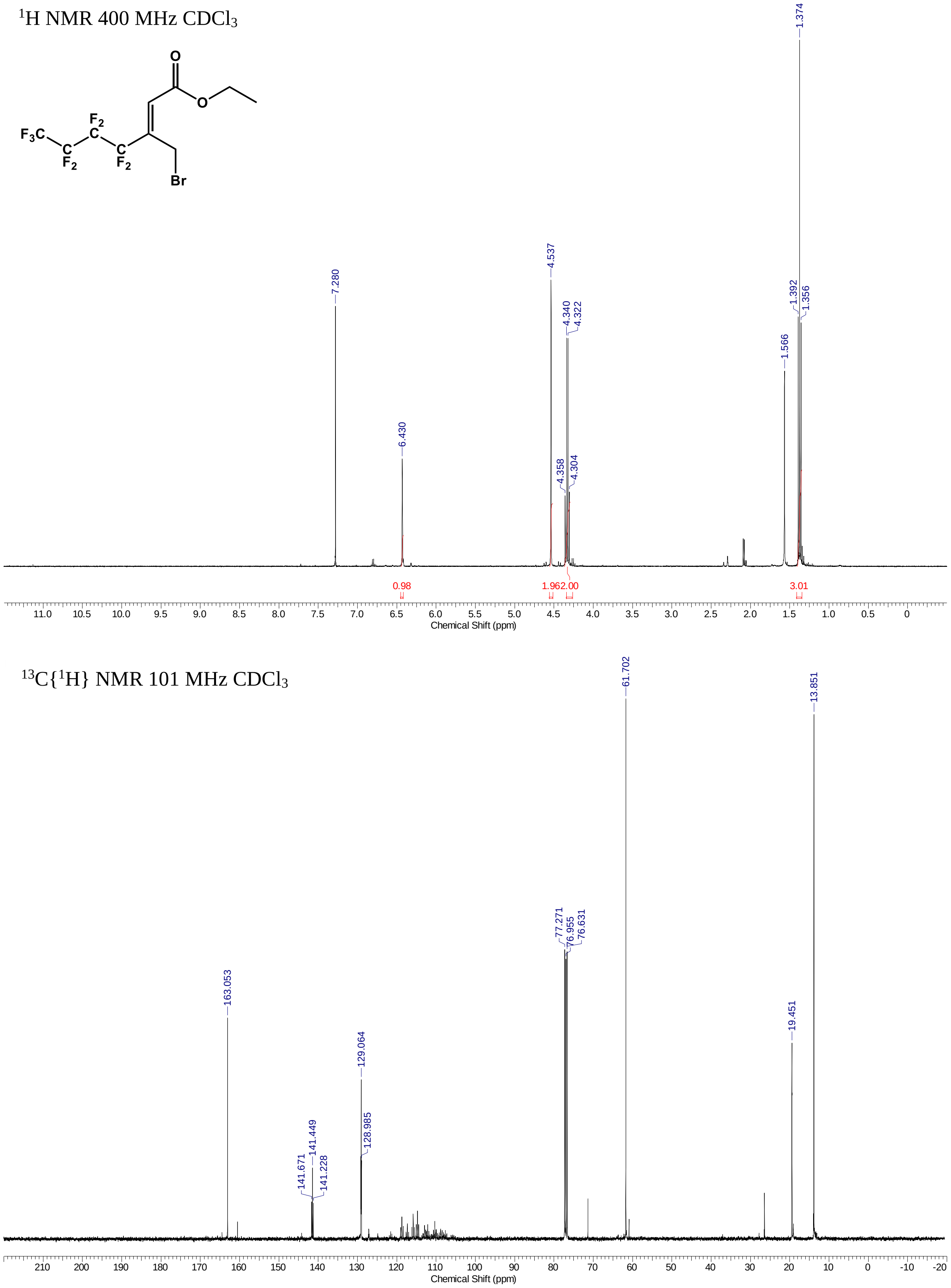

Figure S11. ^{1}H and ^{13}C NMR spectra of compound **6c**.

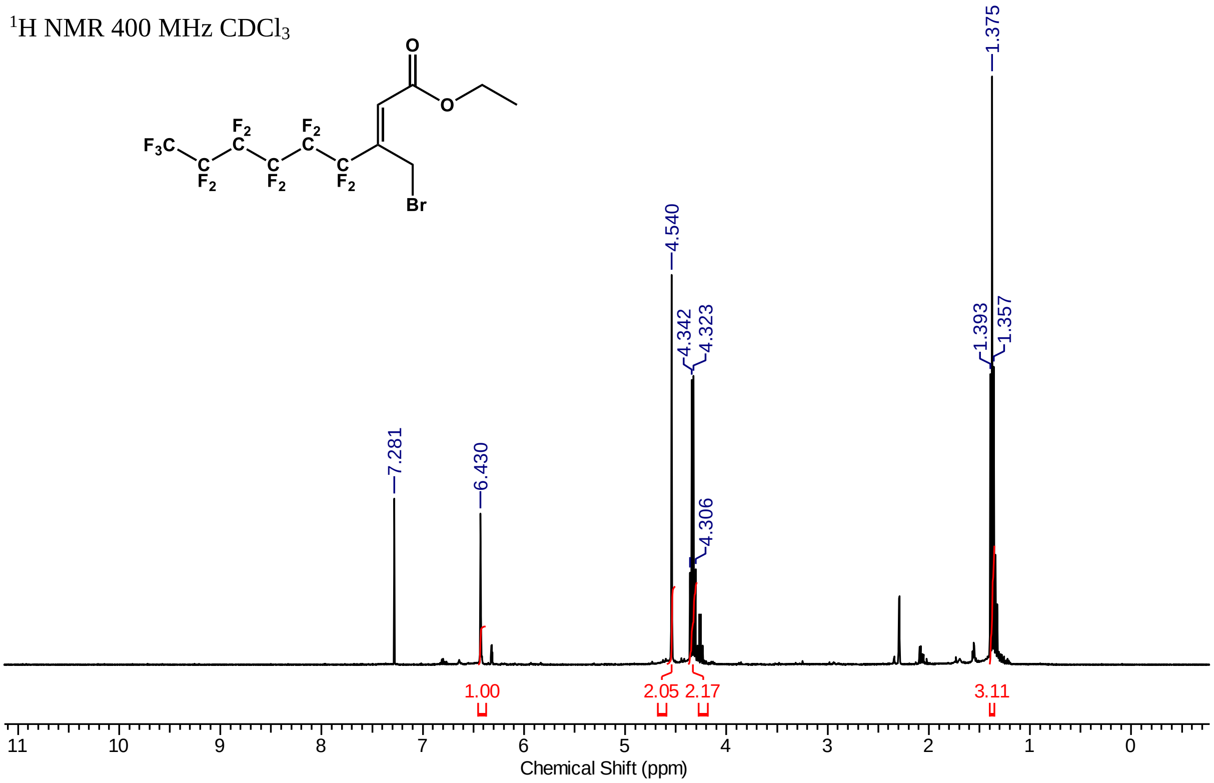

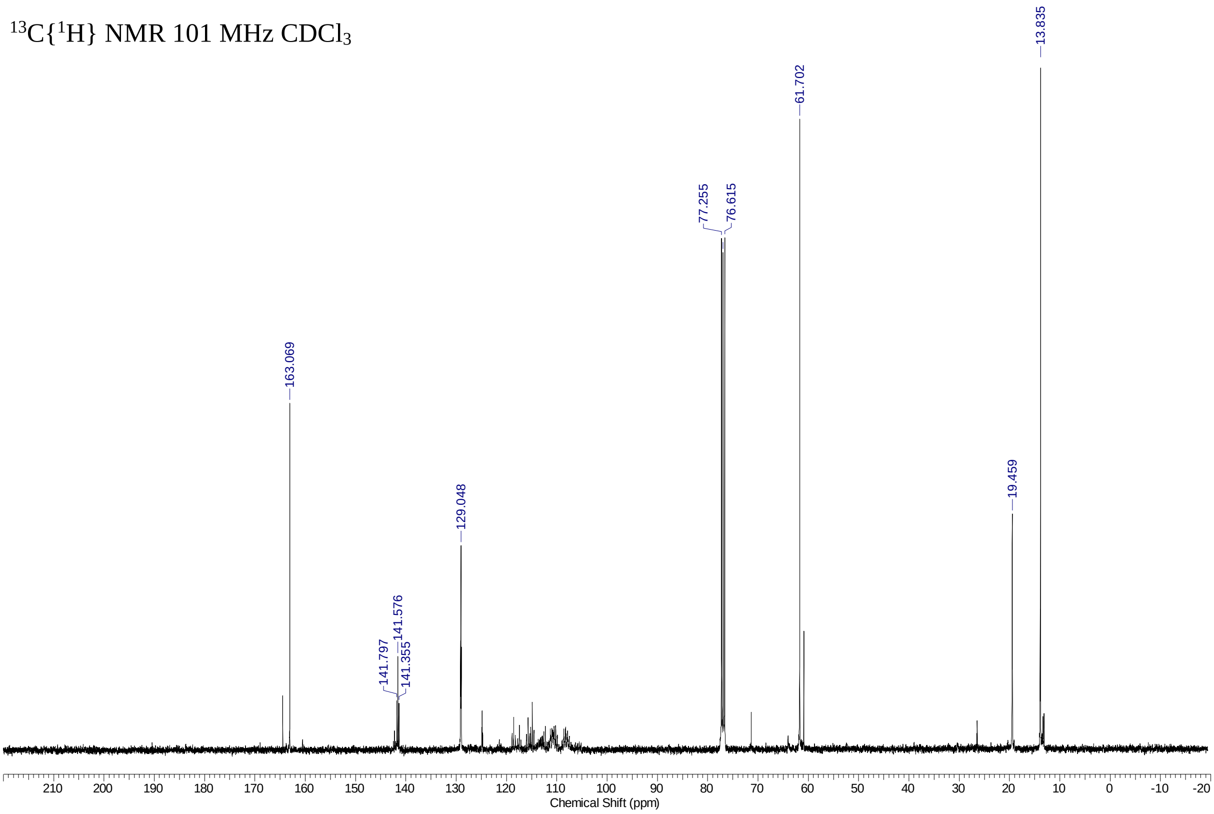

Figure S12. ^{1}H and ^{13}C NMR spectra of compound **6d**.

^1H NMR 700 MHz $CDCl_3$

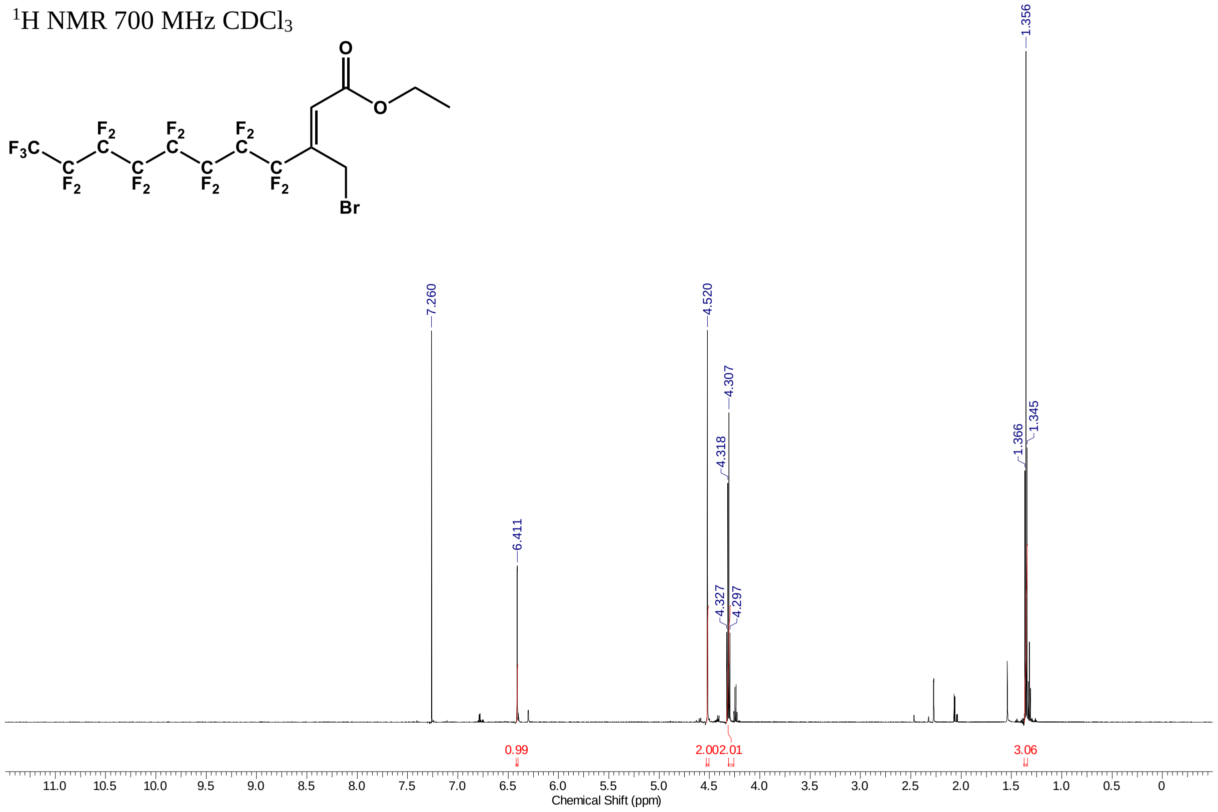

$^{13}C\{^1H\}$ NMR 101 MHz $CDCl_3$

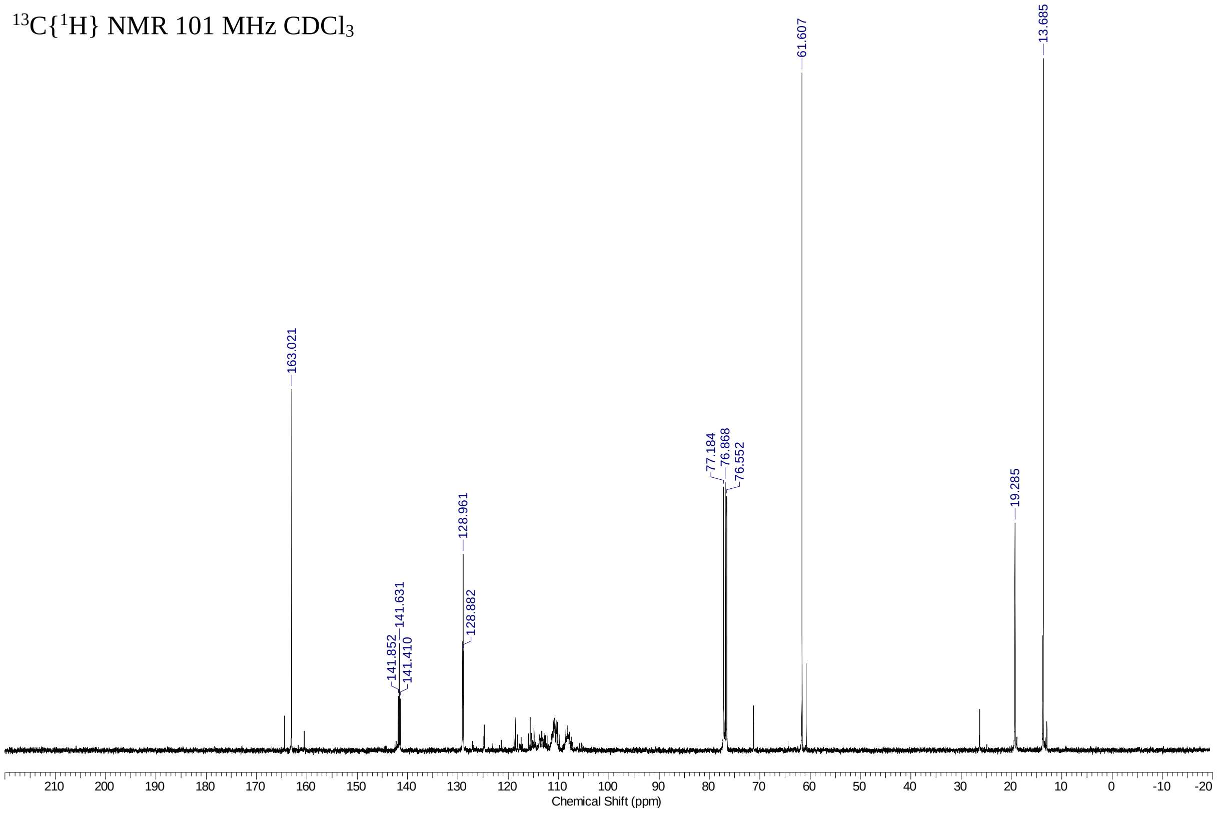

Figure S13. ^1H and ^{13}C NMR spectra of compound **6e**.

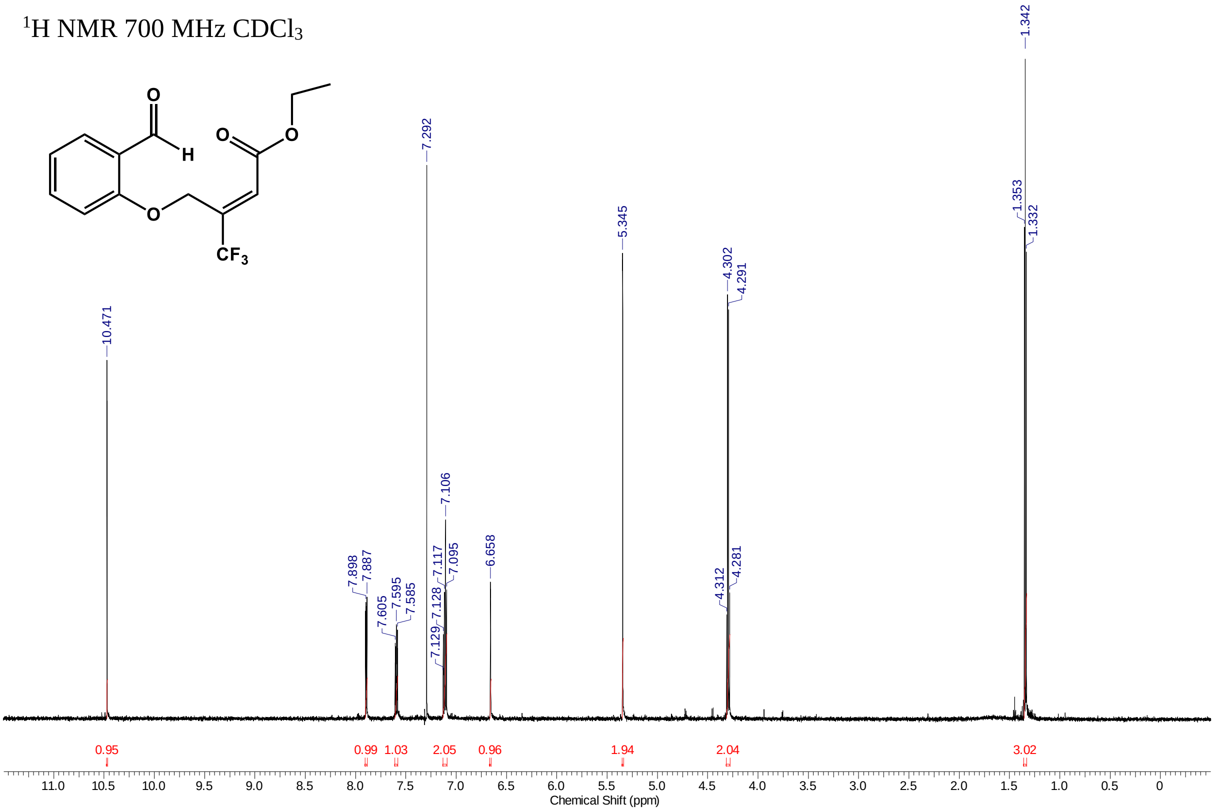

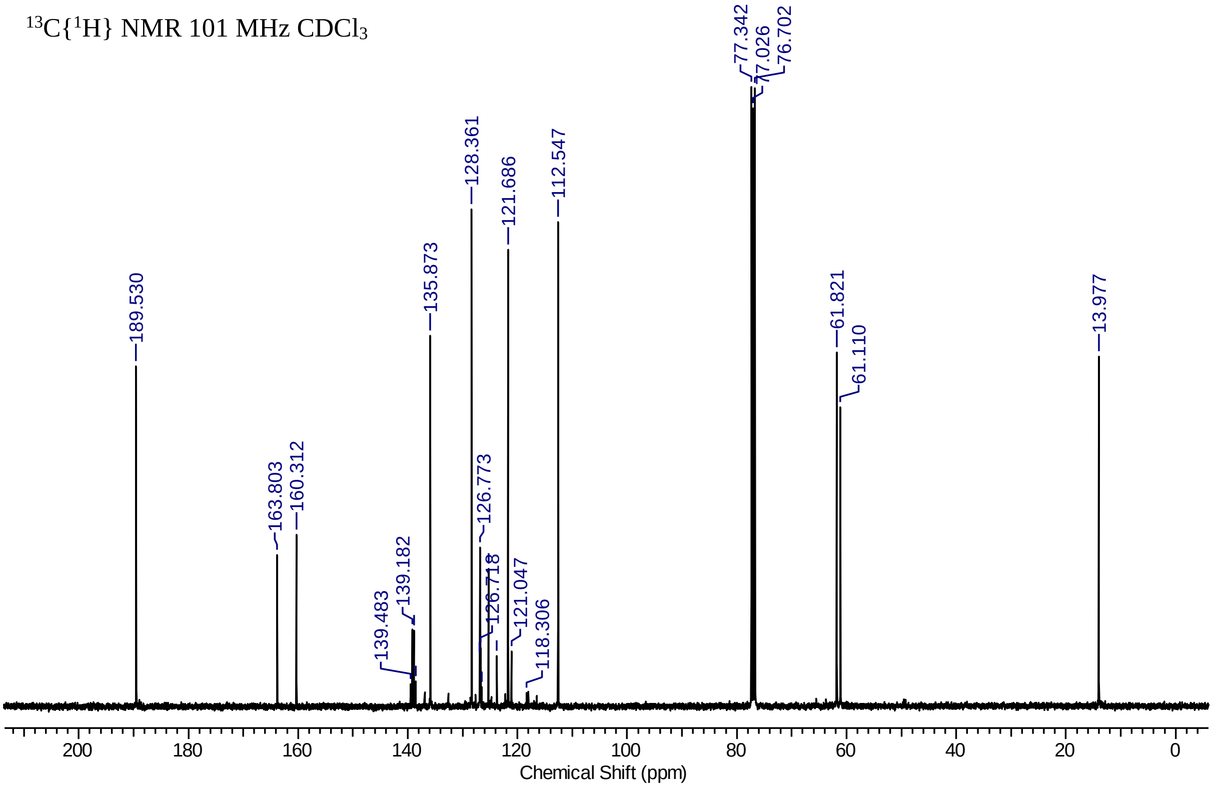

Figure S14. ^{1}H and ^{13}C NMR spectra of compound **1a**.

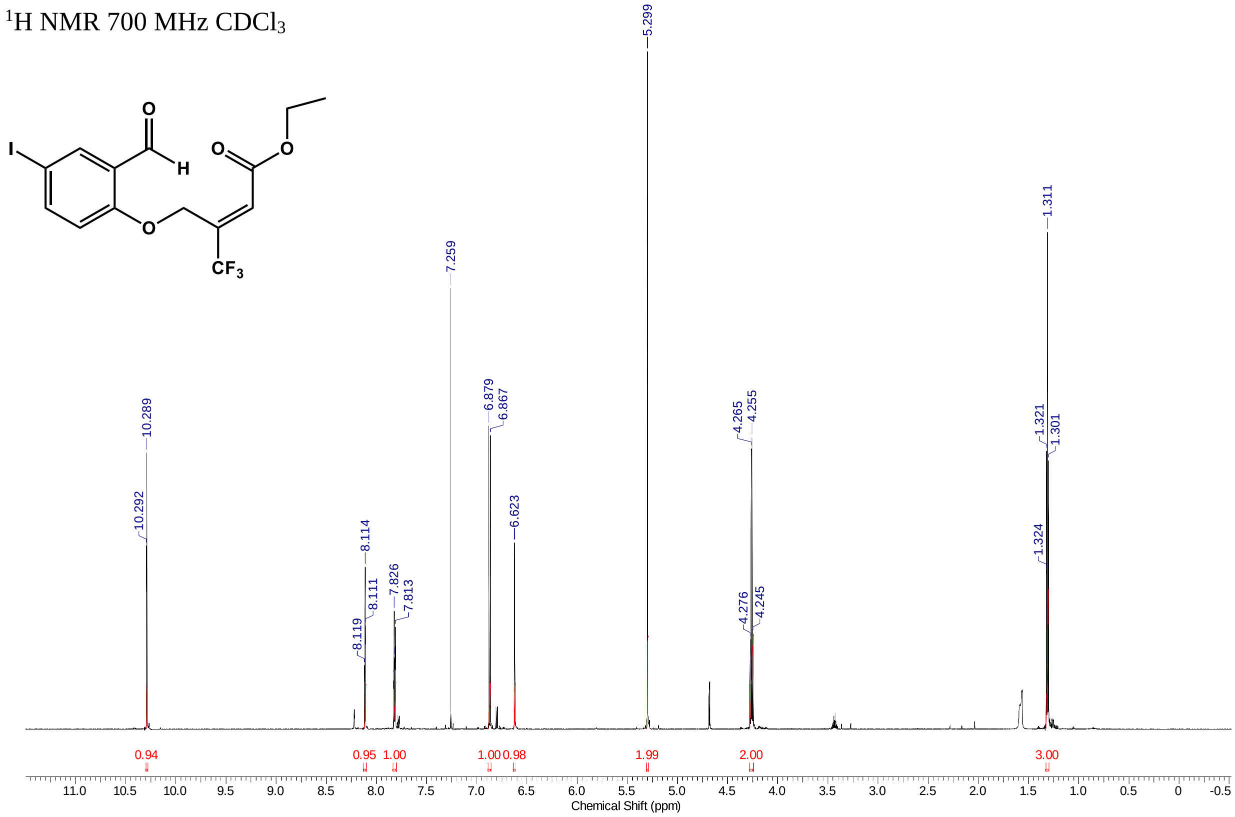

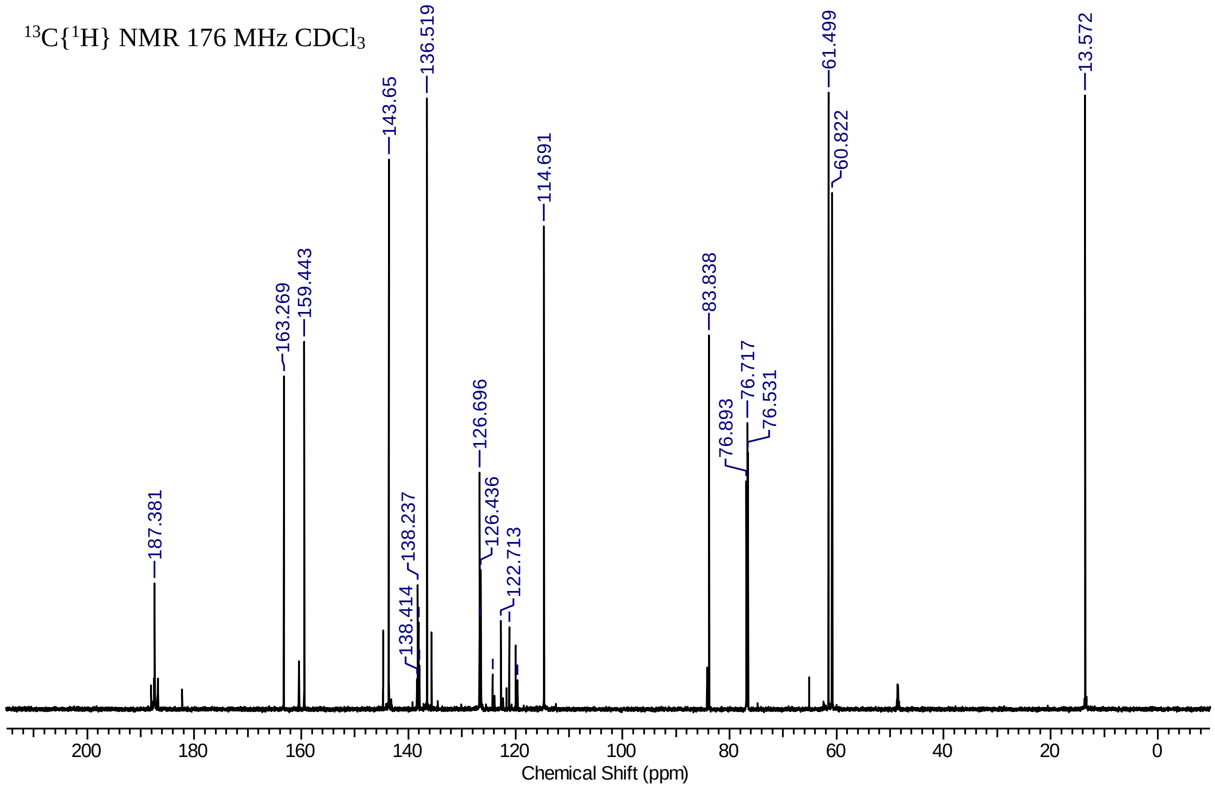

Figure S15. ^{1}H and ^{13}C NMR spectra of compound **1b**.

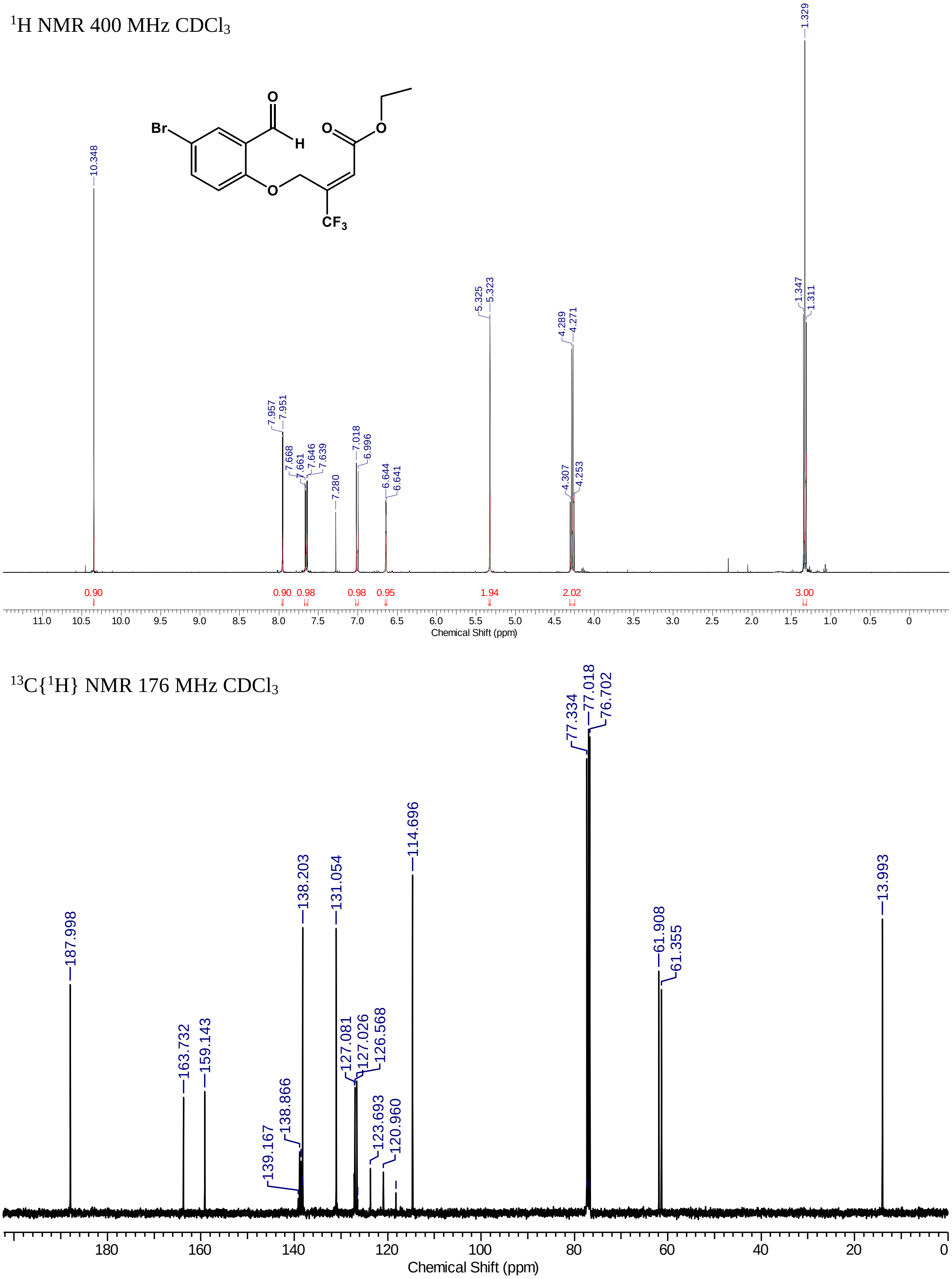

Figure S16. ^{1}H and ^{13}C NMR spectra of compound **1c**.

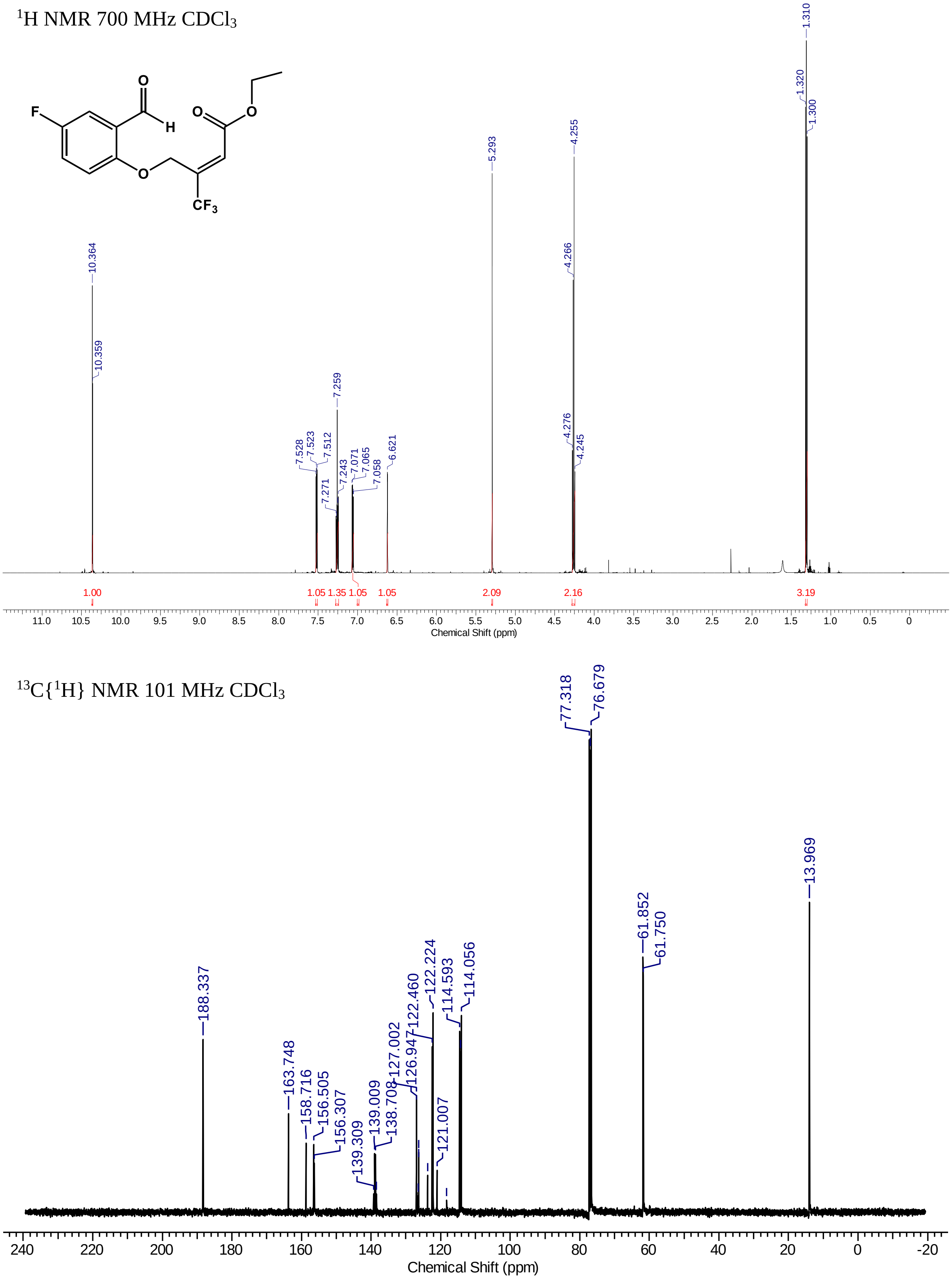

Figure S17. ^{1}H and ^{13}C NMR spectra of compound **1d**.

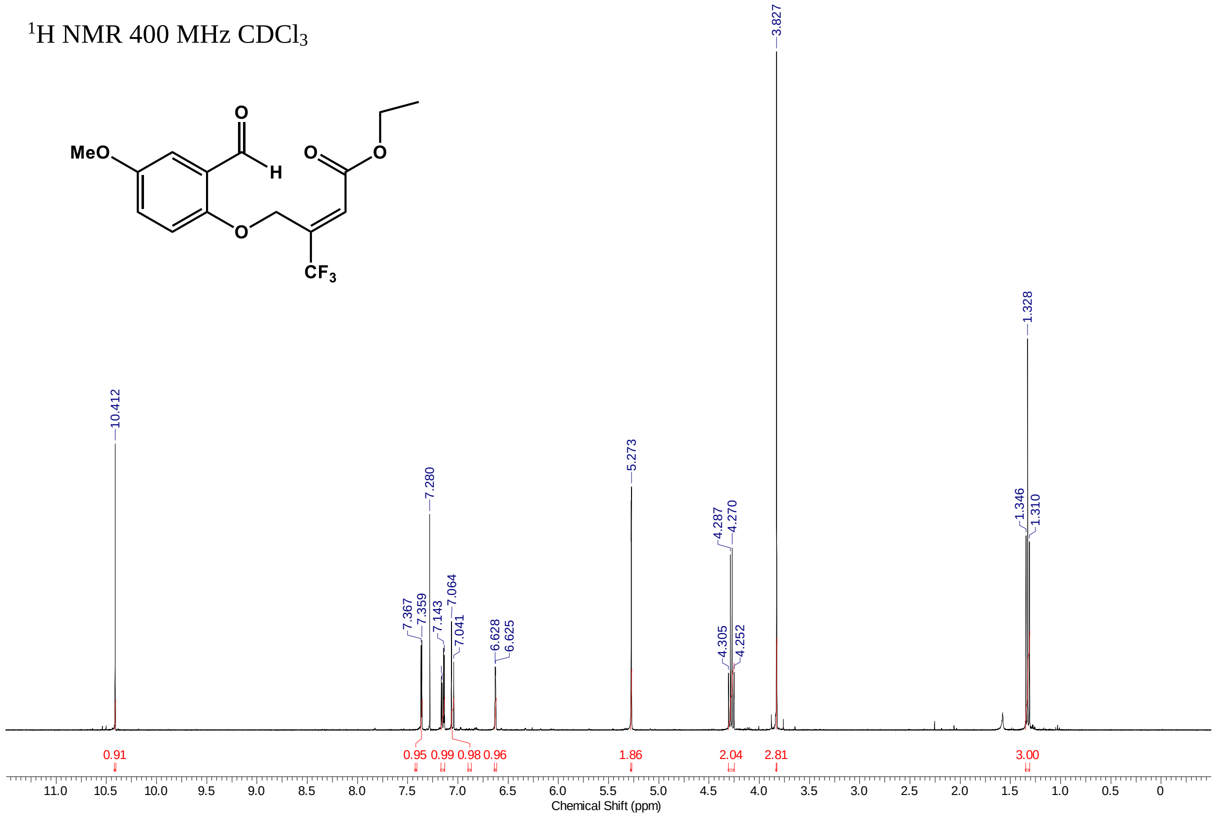

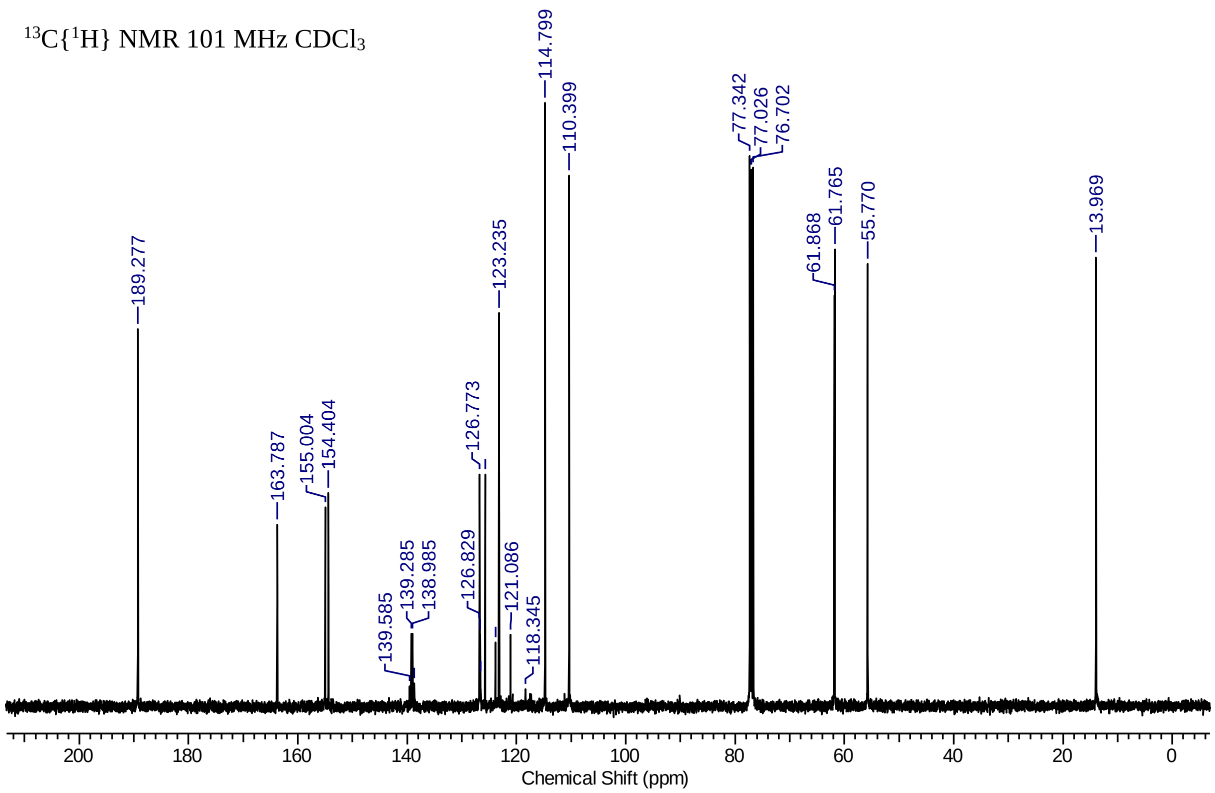

Figure S18. ^{1}H and ^{13}C NMR spectra of compound **1e**.

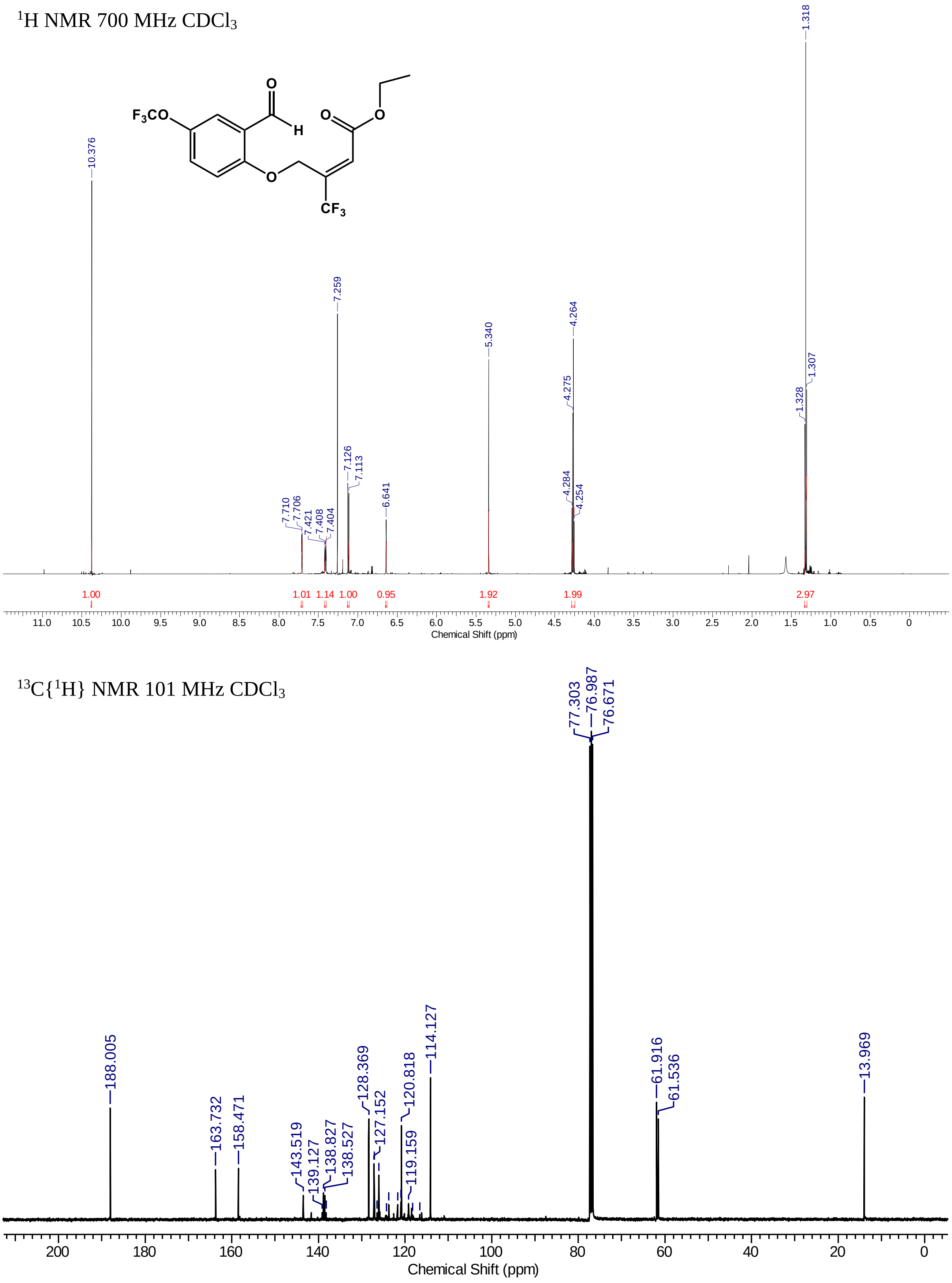

Figure S19. ^{1}H and ^{13}C NMR spectra of compound **1f**.

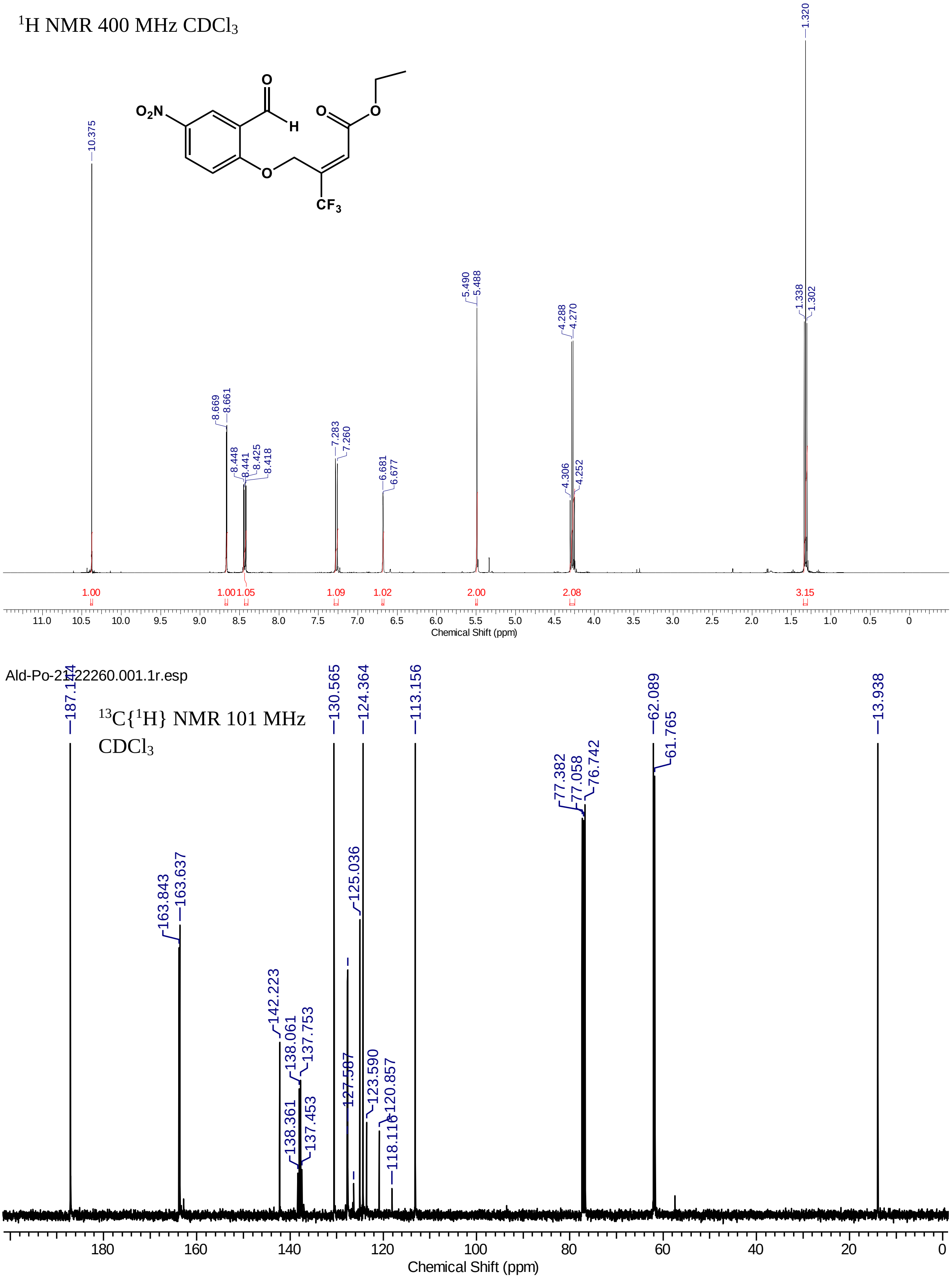

Figure S20. ^{1}H and ^{13}C NMR spectra of compound **1g**.

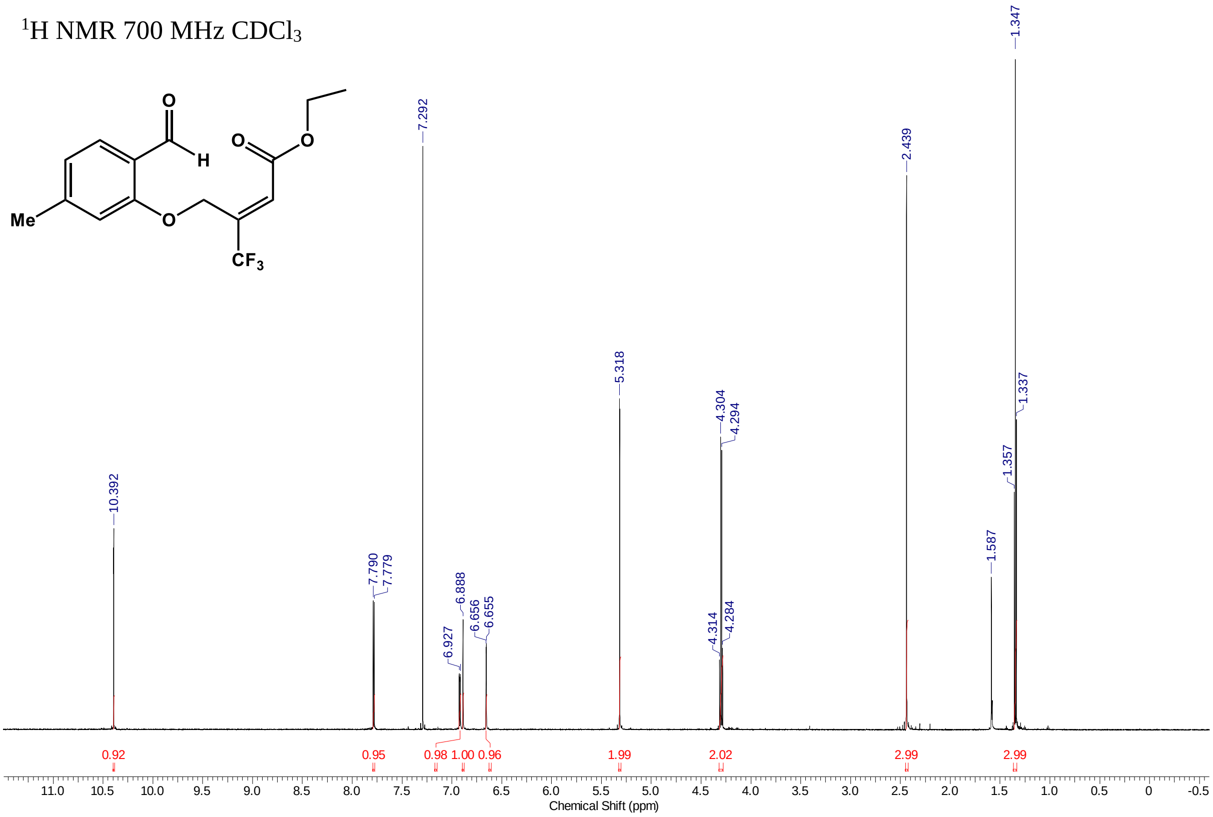

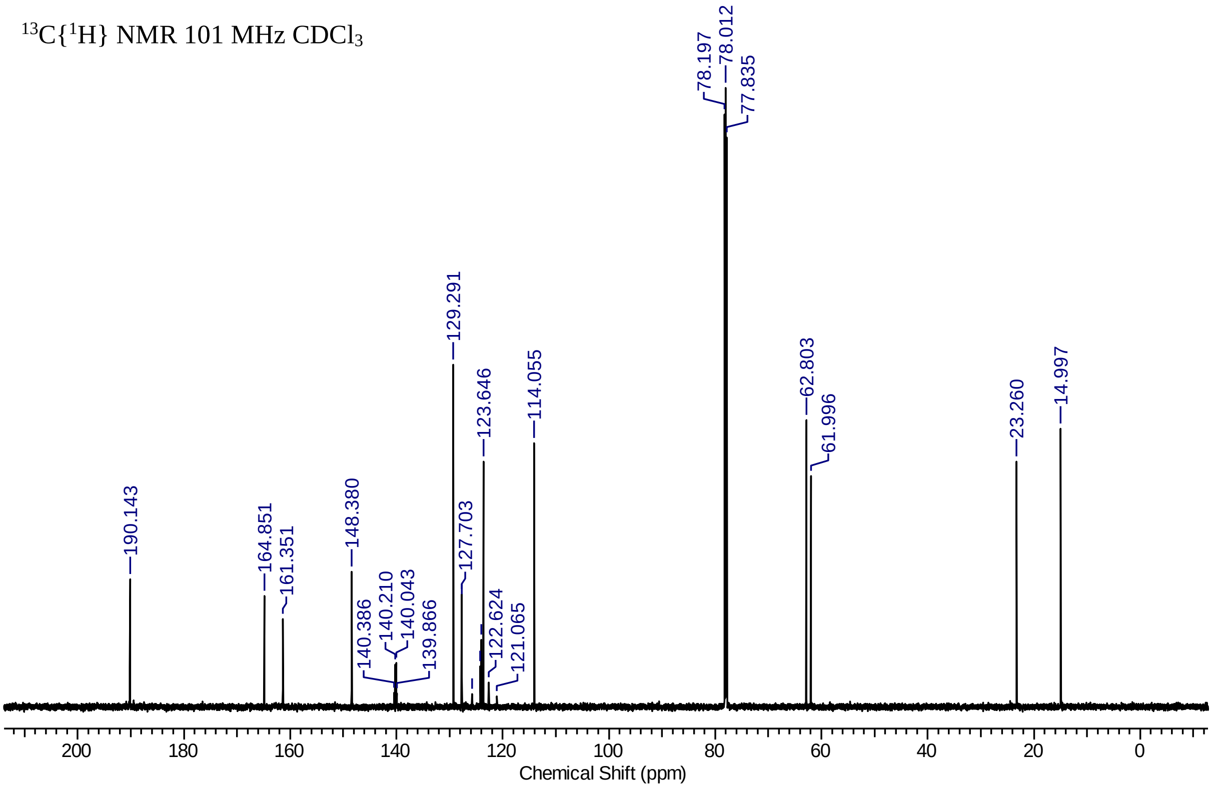

Figure S21. ^{1}H and ^{13}C NMR spectra of compound **1h**.

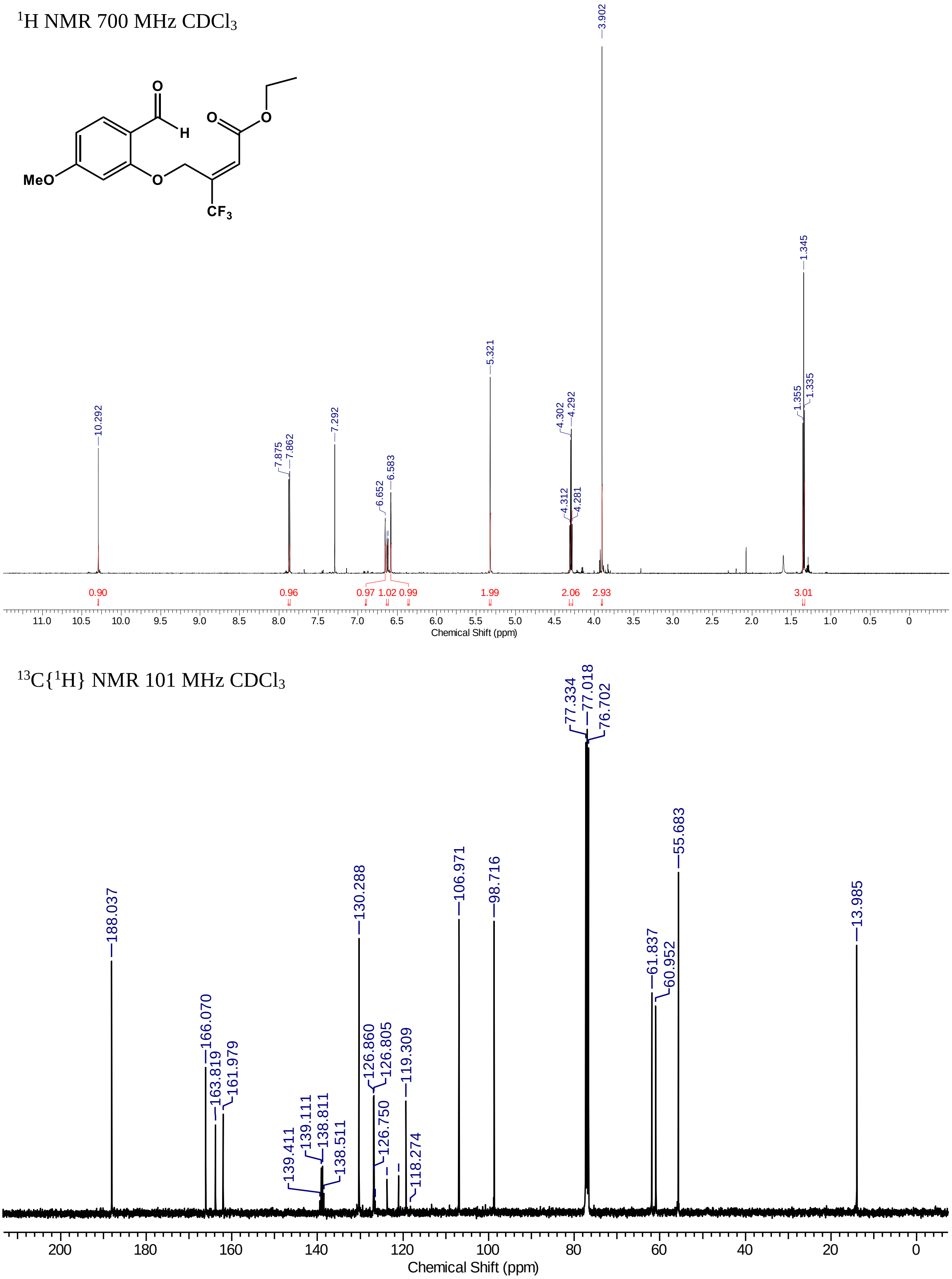

Figure S22. ^{1}H and ^{13}C NMR spectra of compound **1i**.

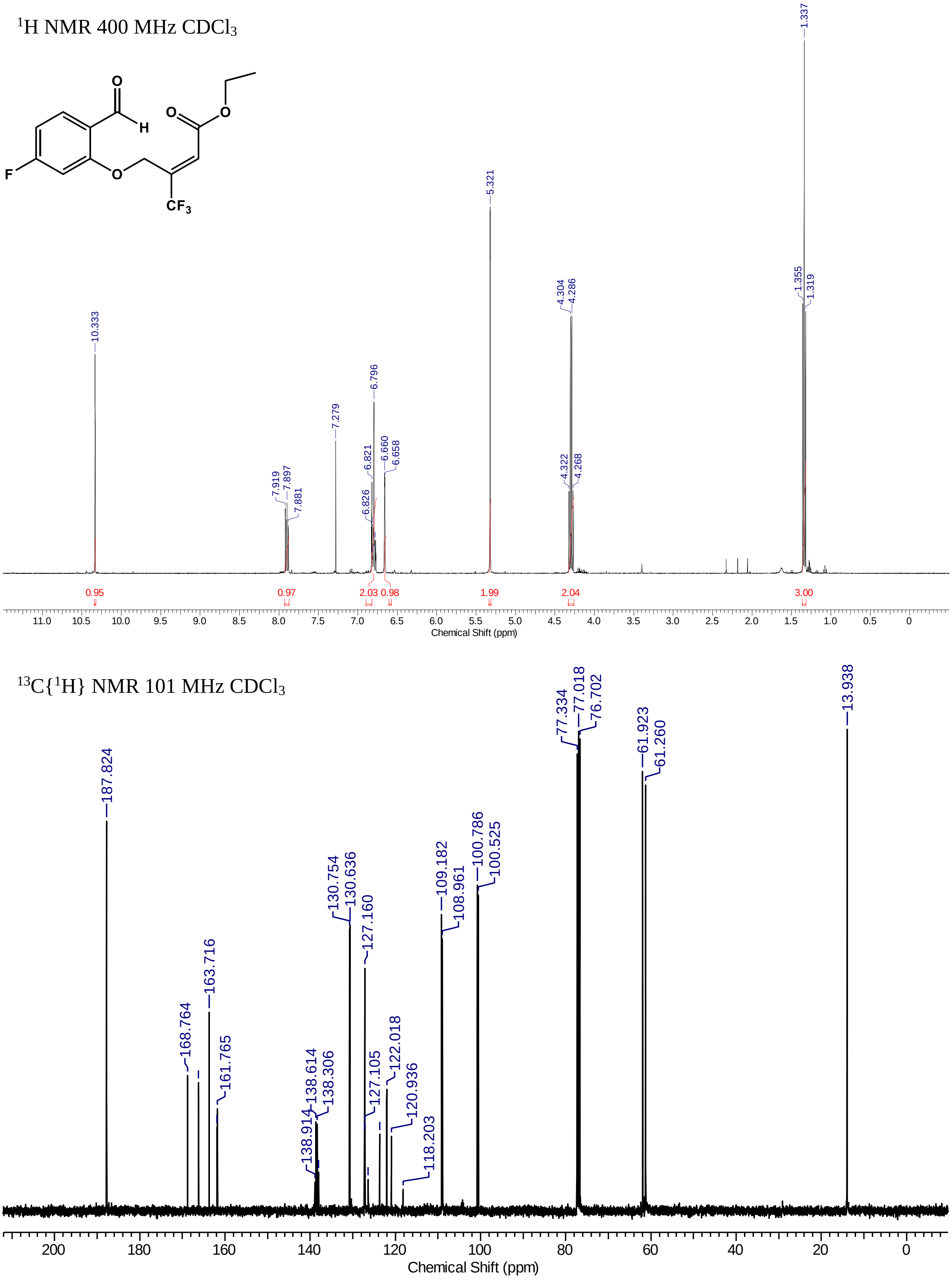

Figure S23. ^{1}H and ^{13}C NMR spectra of compound **1j**.

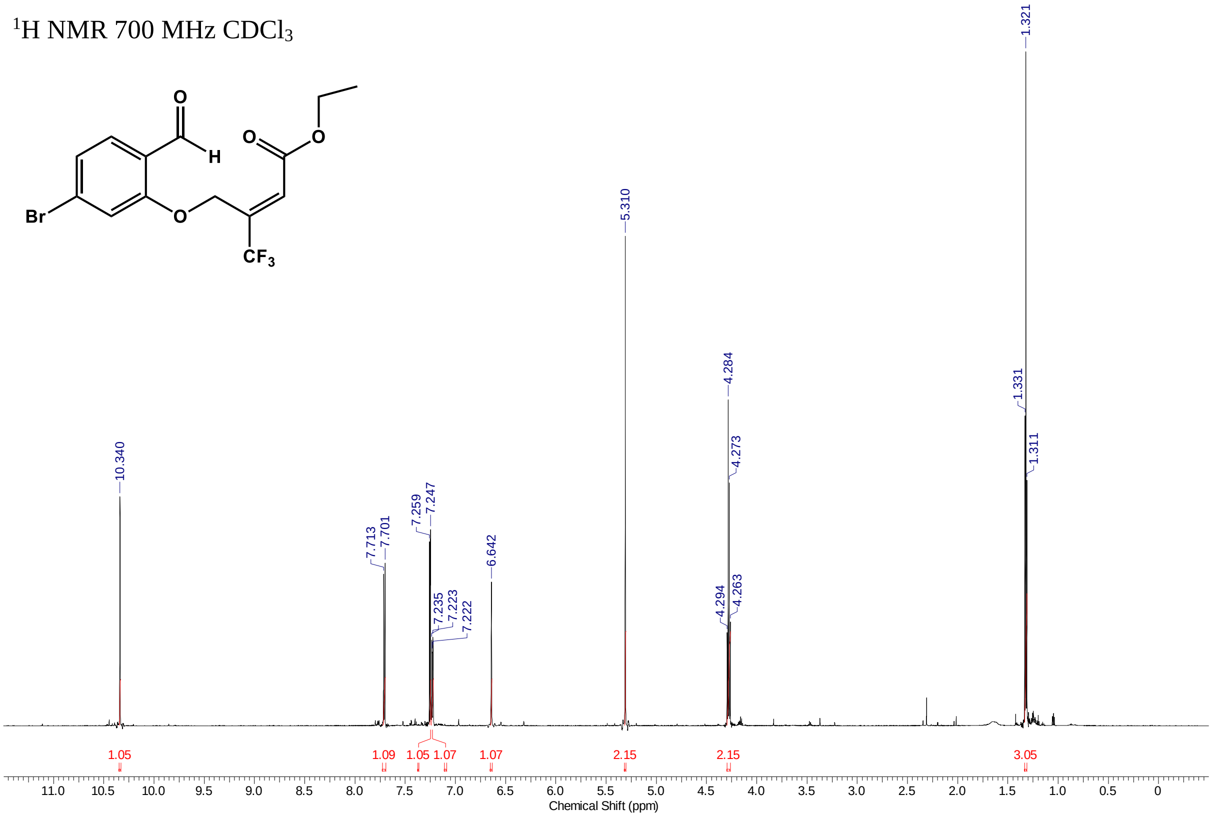

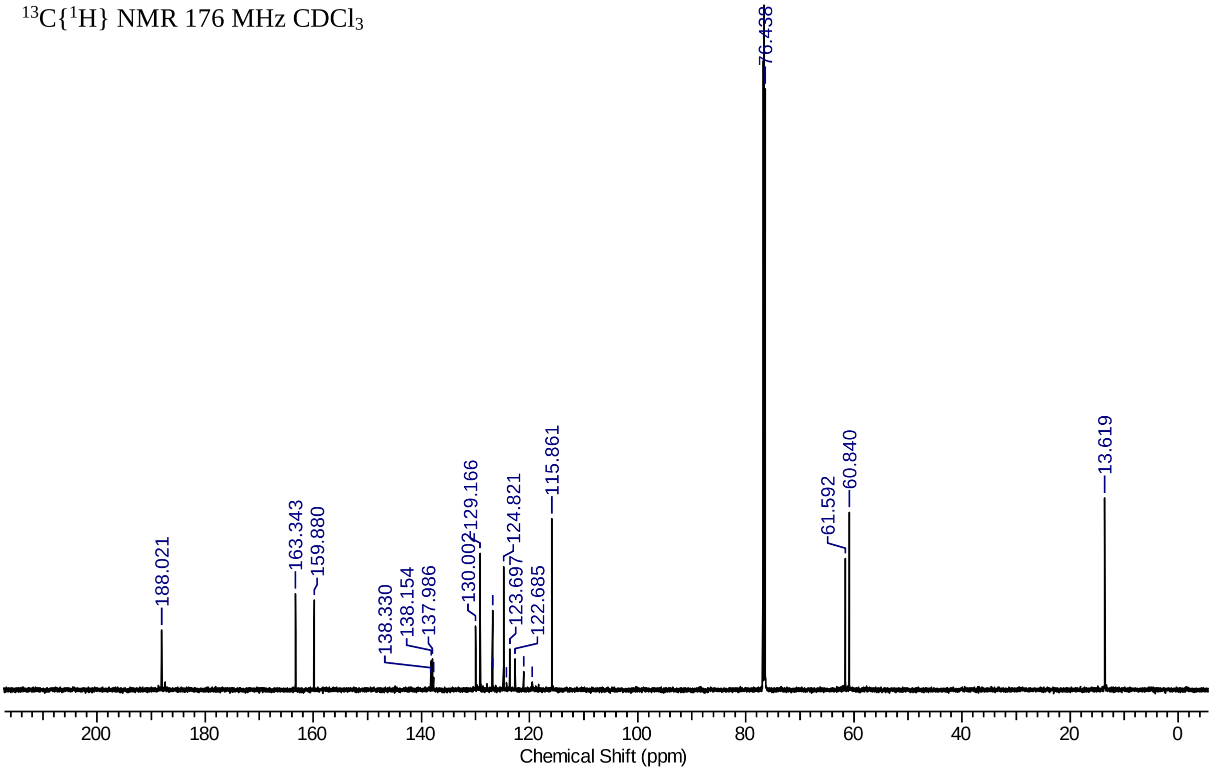

Figure S24. ¹H and ¹³C NMR spectra of compound **1k**.

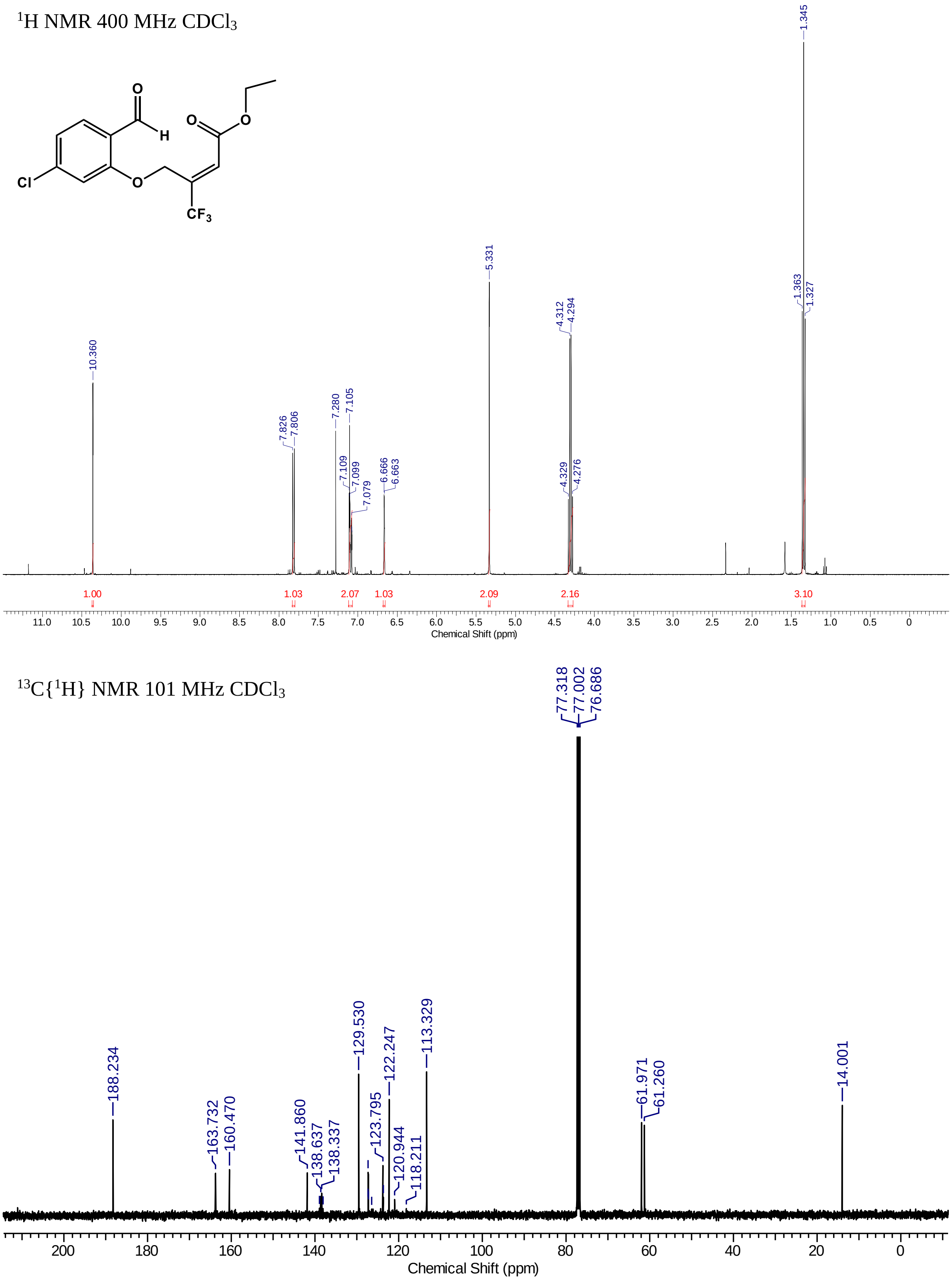

Figure S25. ^{1}H and ^{13}C NMR spectra of compound **1l**.

^{1}H NMR 700 MHz $CDCl_3$

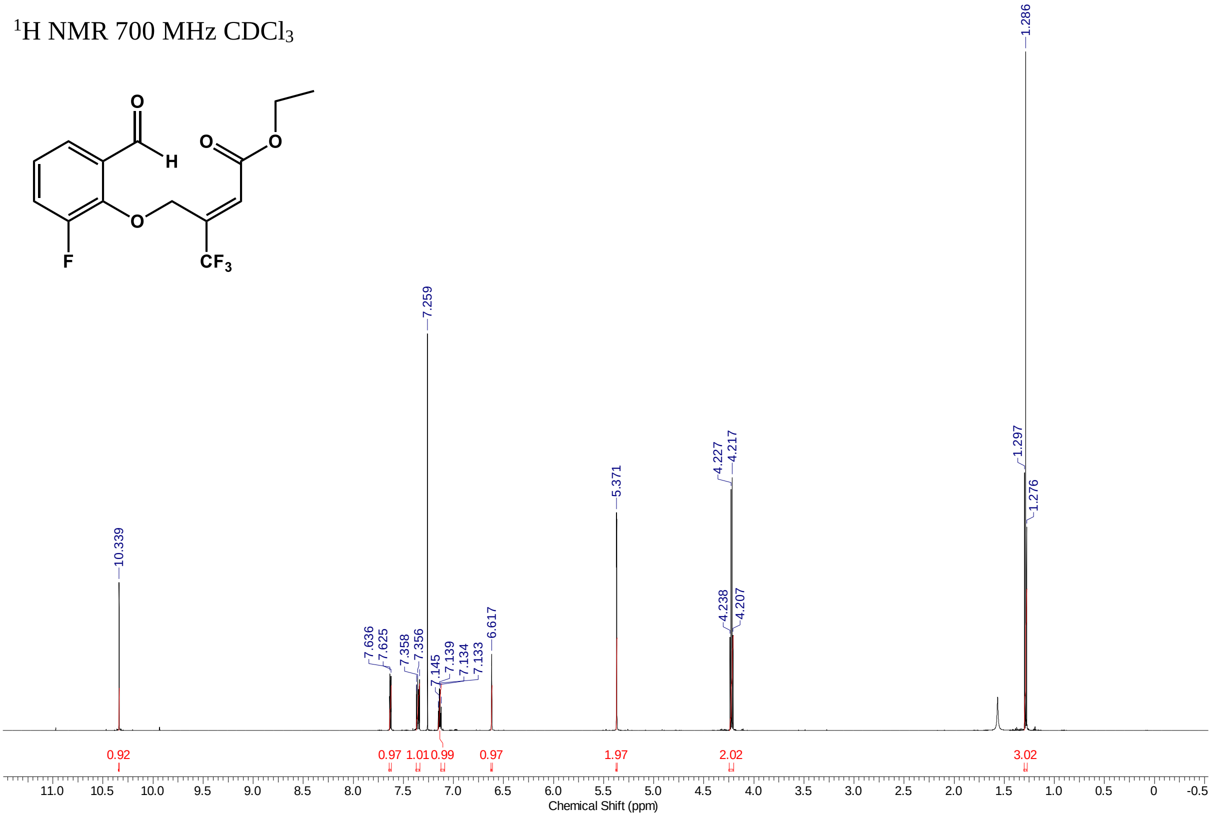

$^{13}C\{^{1}H\}$ NMR 101 MHz $CDCl_3$

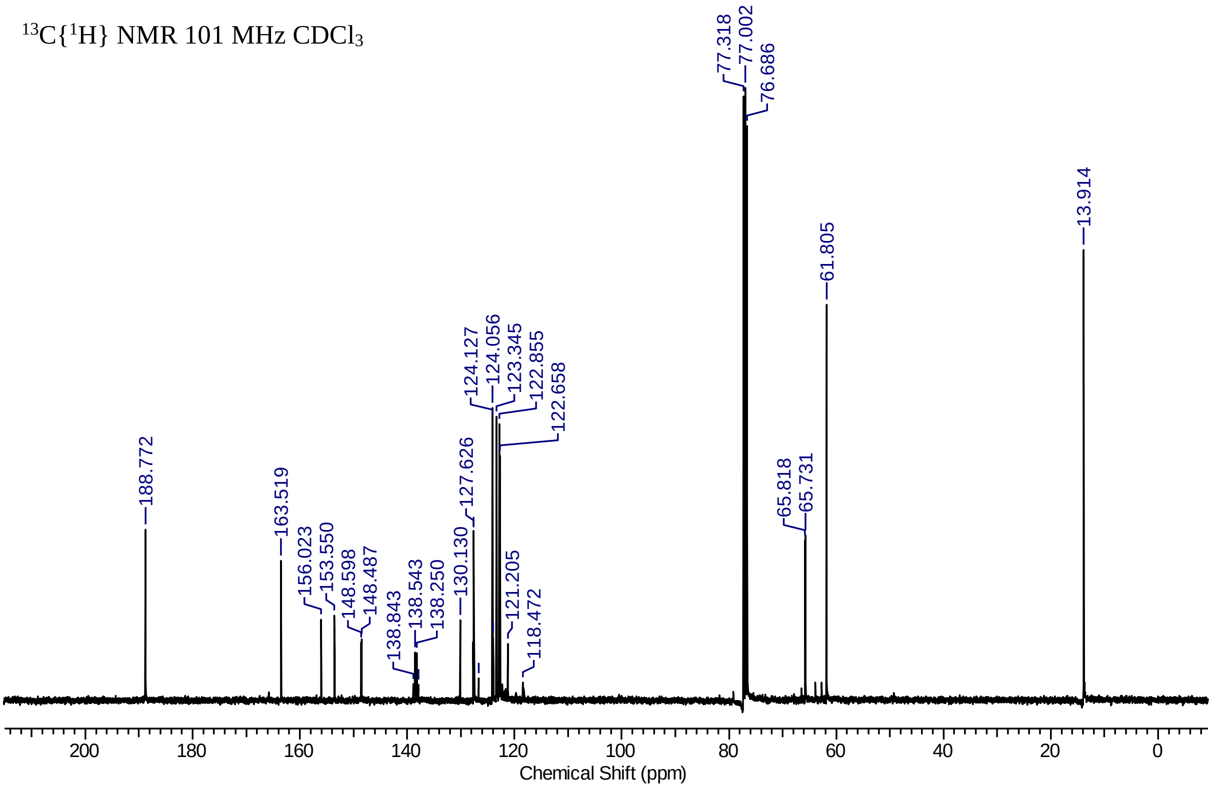

Figure S26. ^{1}H and ^{13}C NMR spectra of compound **1m**.

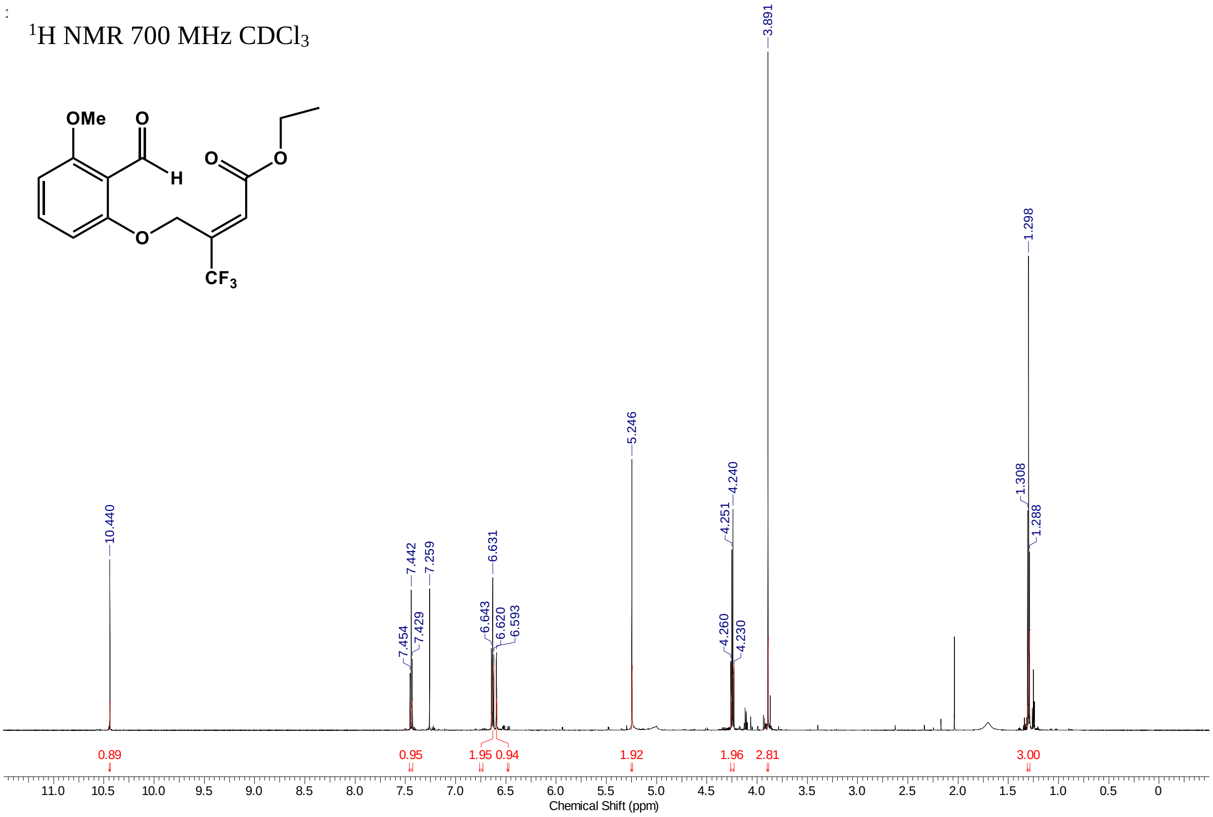

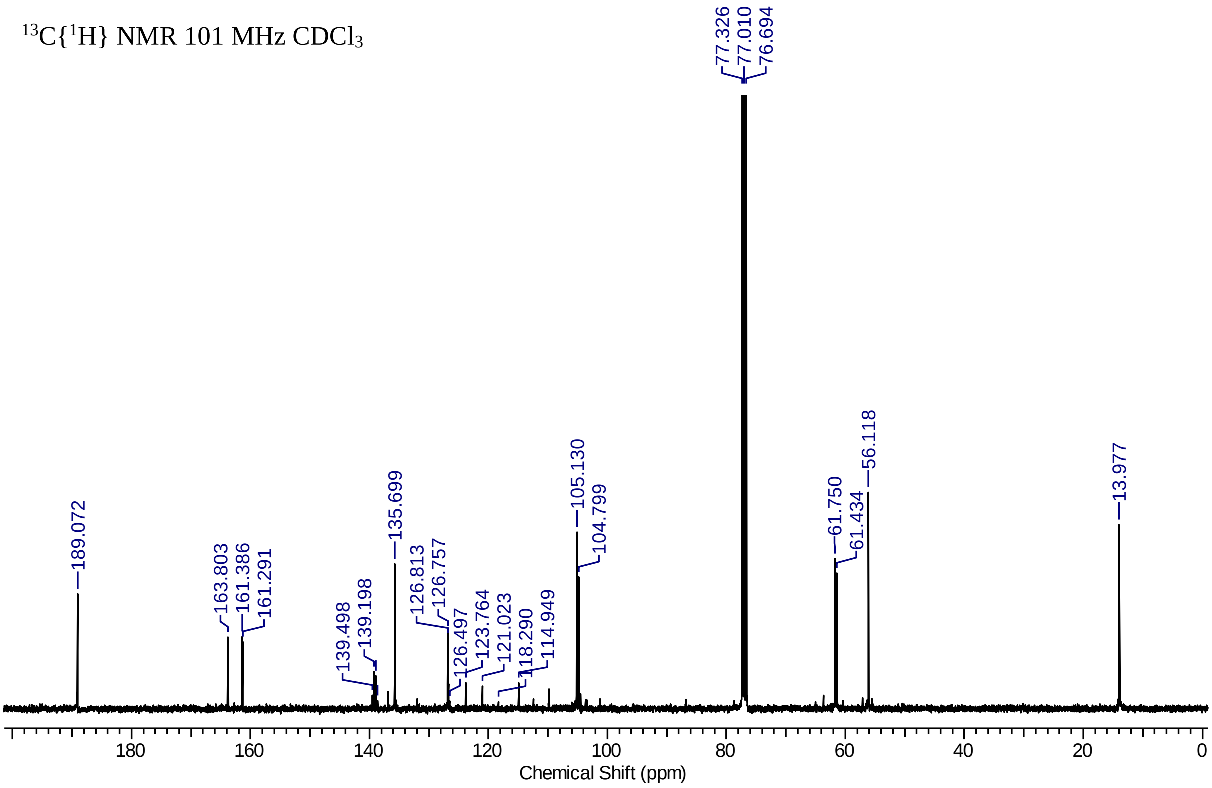

Figure S27. ^{1}H and ^{13}C NMR spectra of compound **1n**.

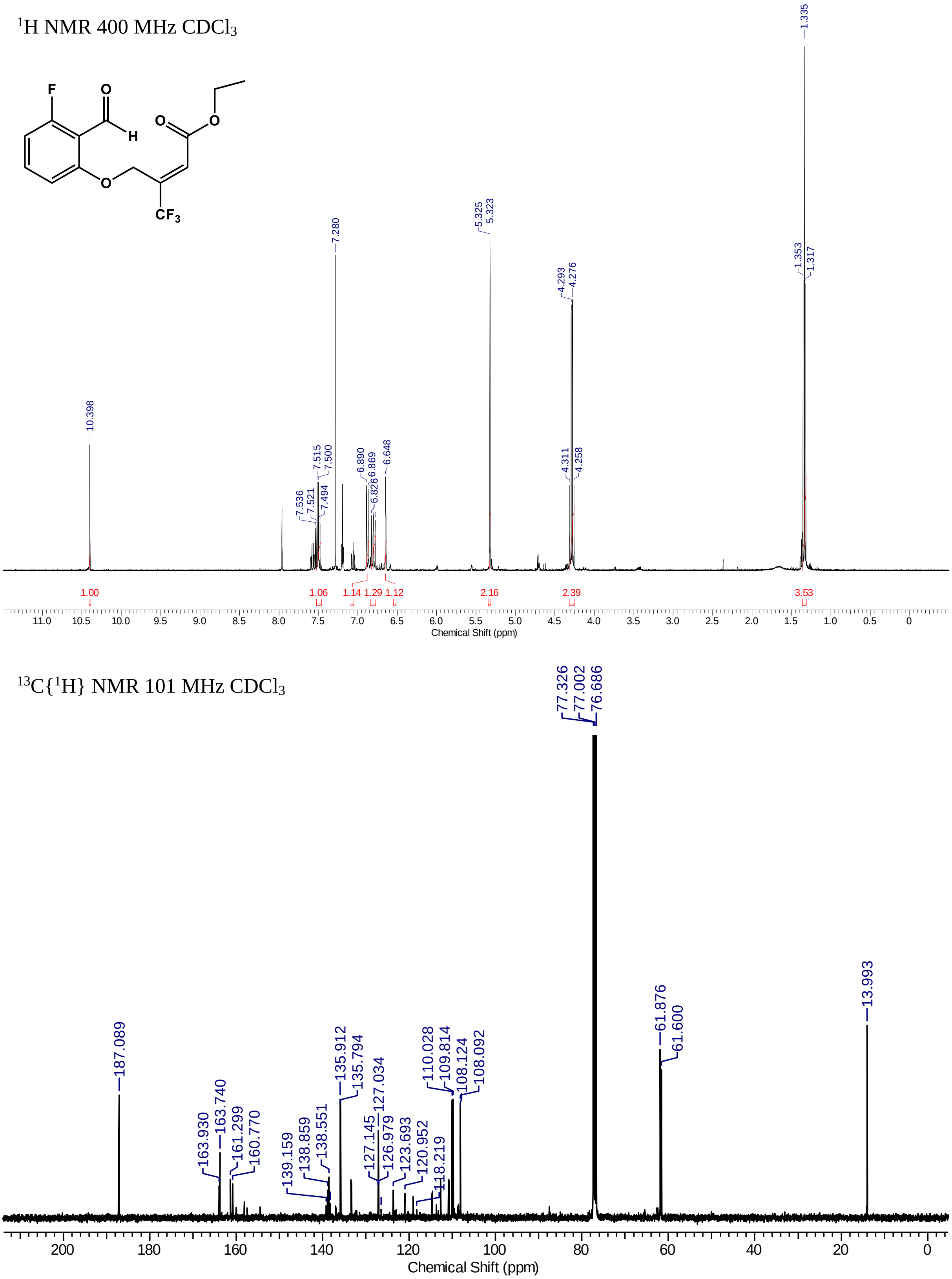

Figure S28. ^{1}H and ^{13}C NMR spectra of compound **1o**.

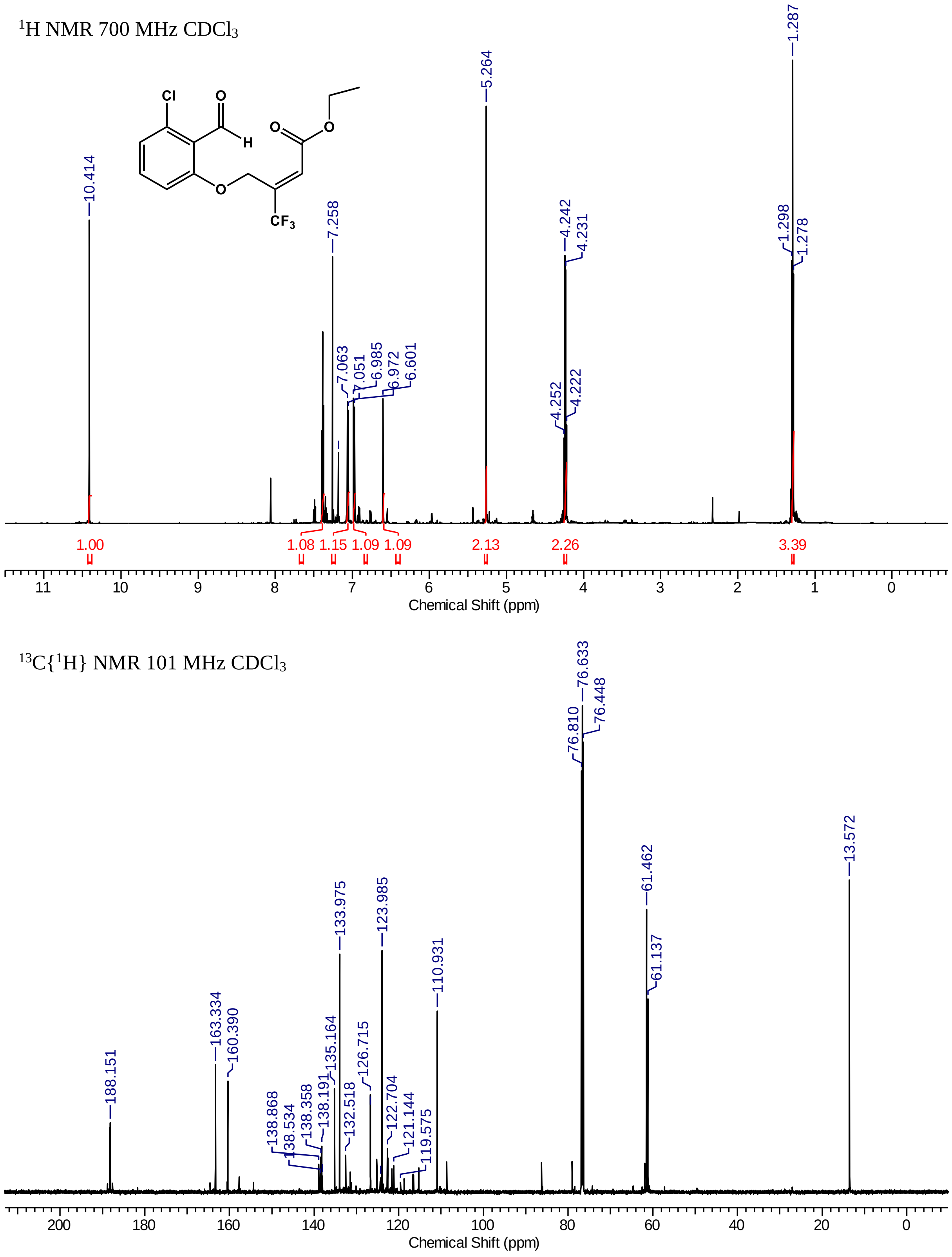

Figure S29. ^{1}H and ^{13}C NMR spectra of compound **1p**.

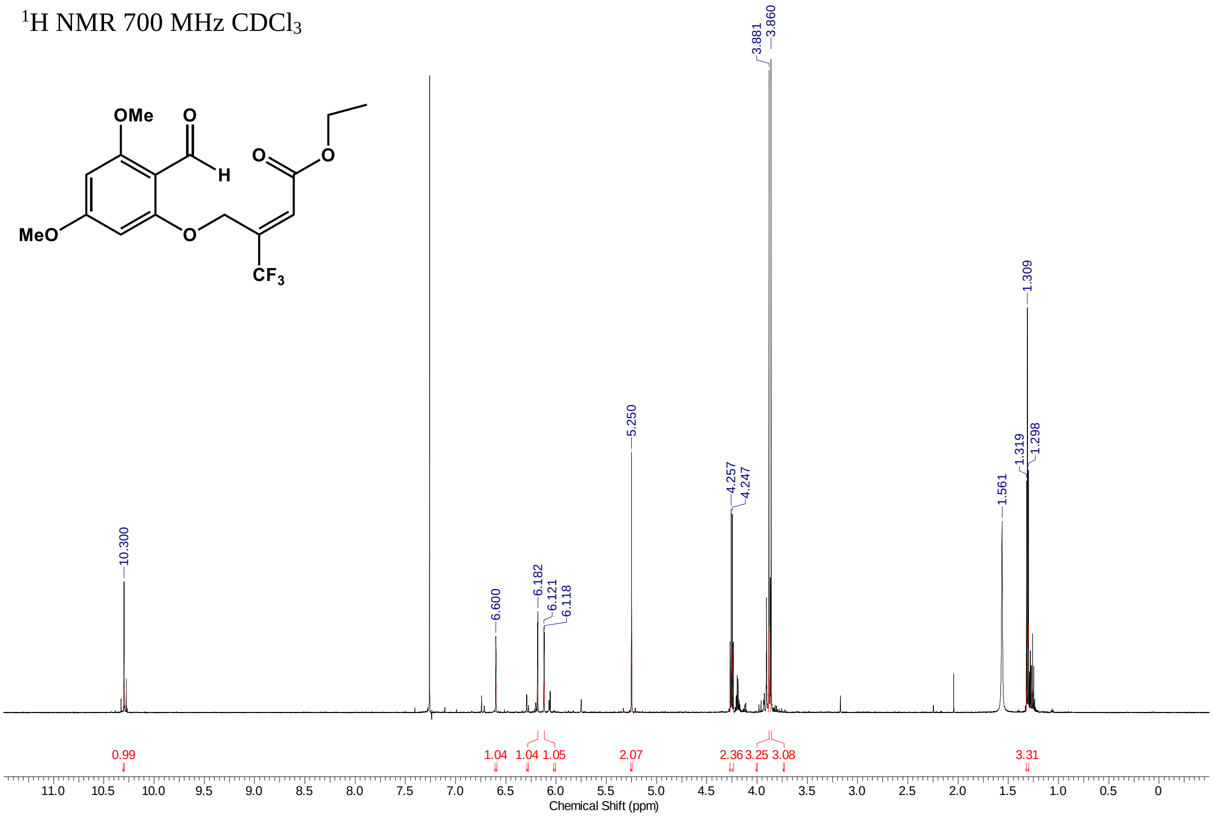

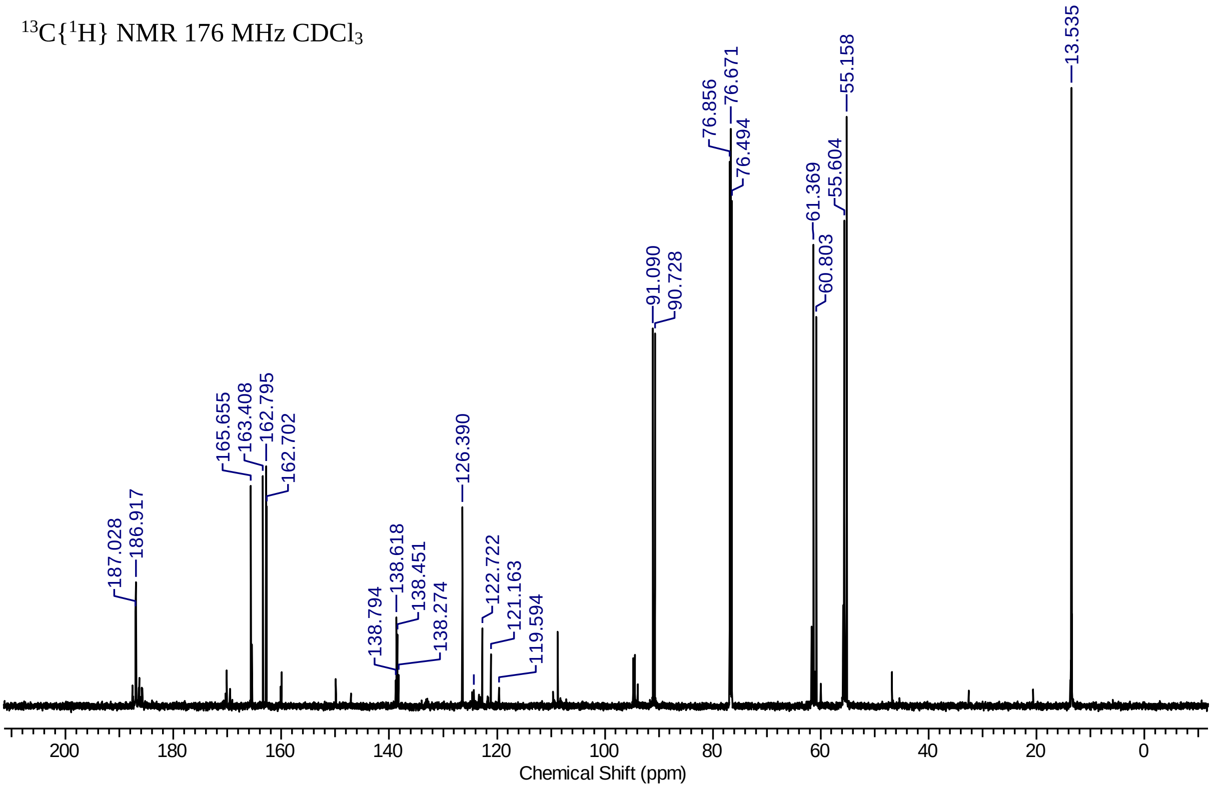

Figure S30. ^{1}H and ^{13}C NMR spectra of compound **1q**.

^{1}H NMR 700 MHz $CDCl_3$

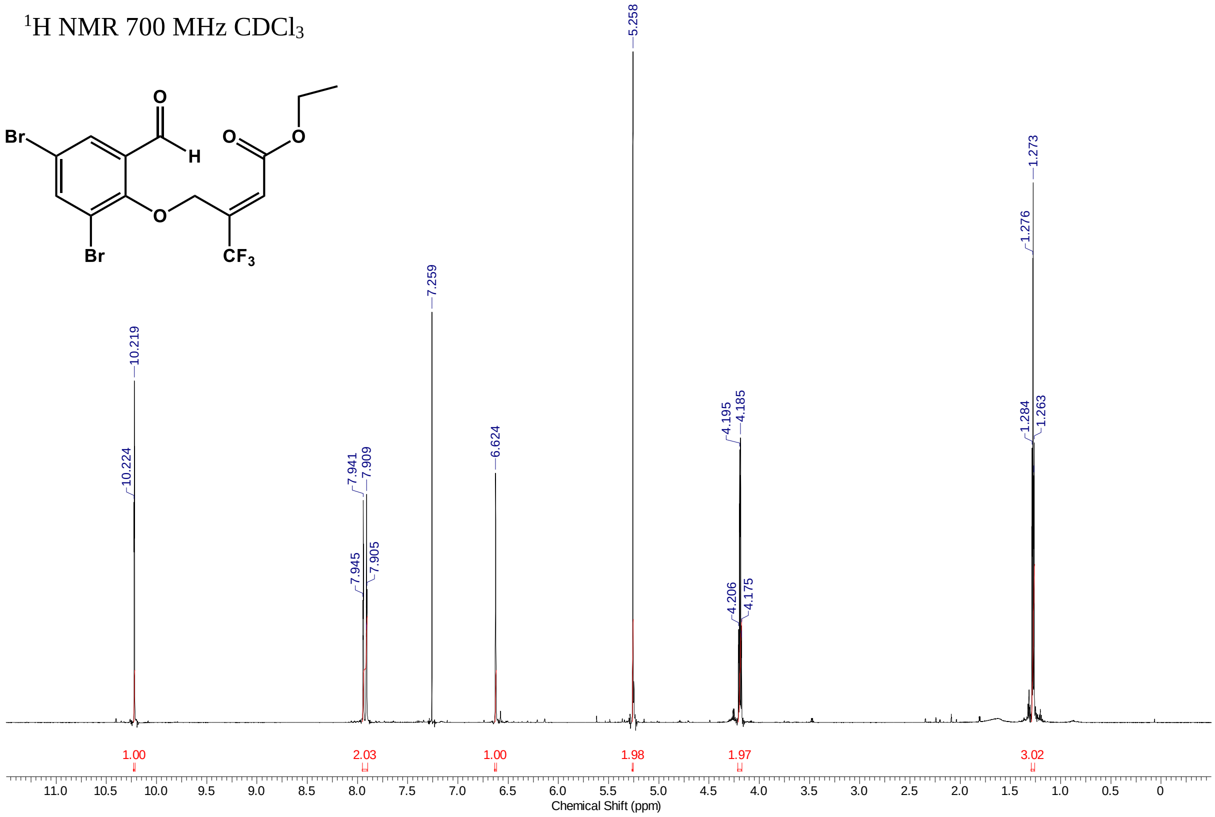

$^{13}C\{^{1}H\}$ NMR 101 MHz $CDCl_3$

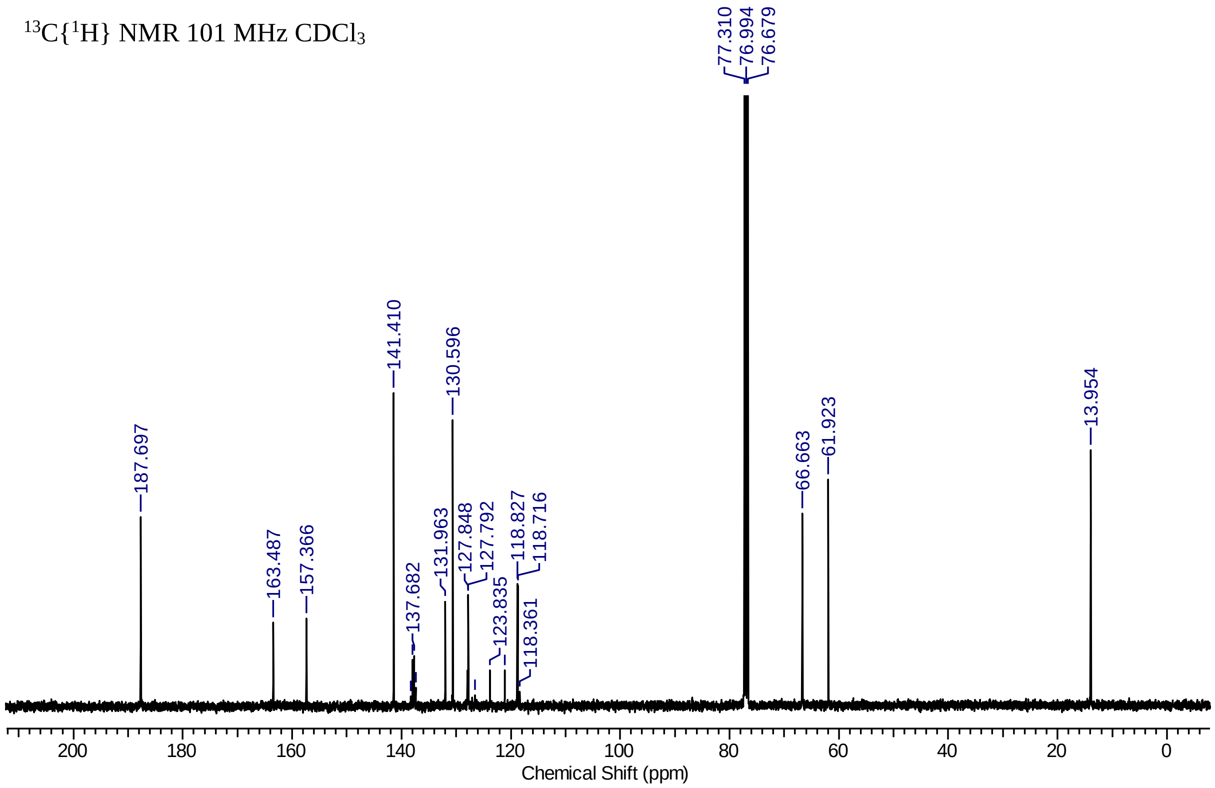

Figure S31. ^{1}H and ^{13}C NMR spectra of compound **1r**.

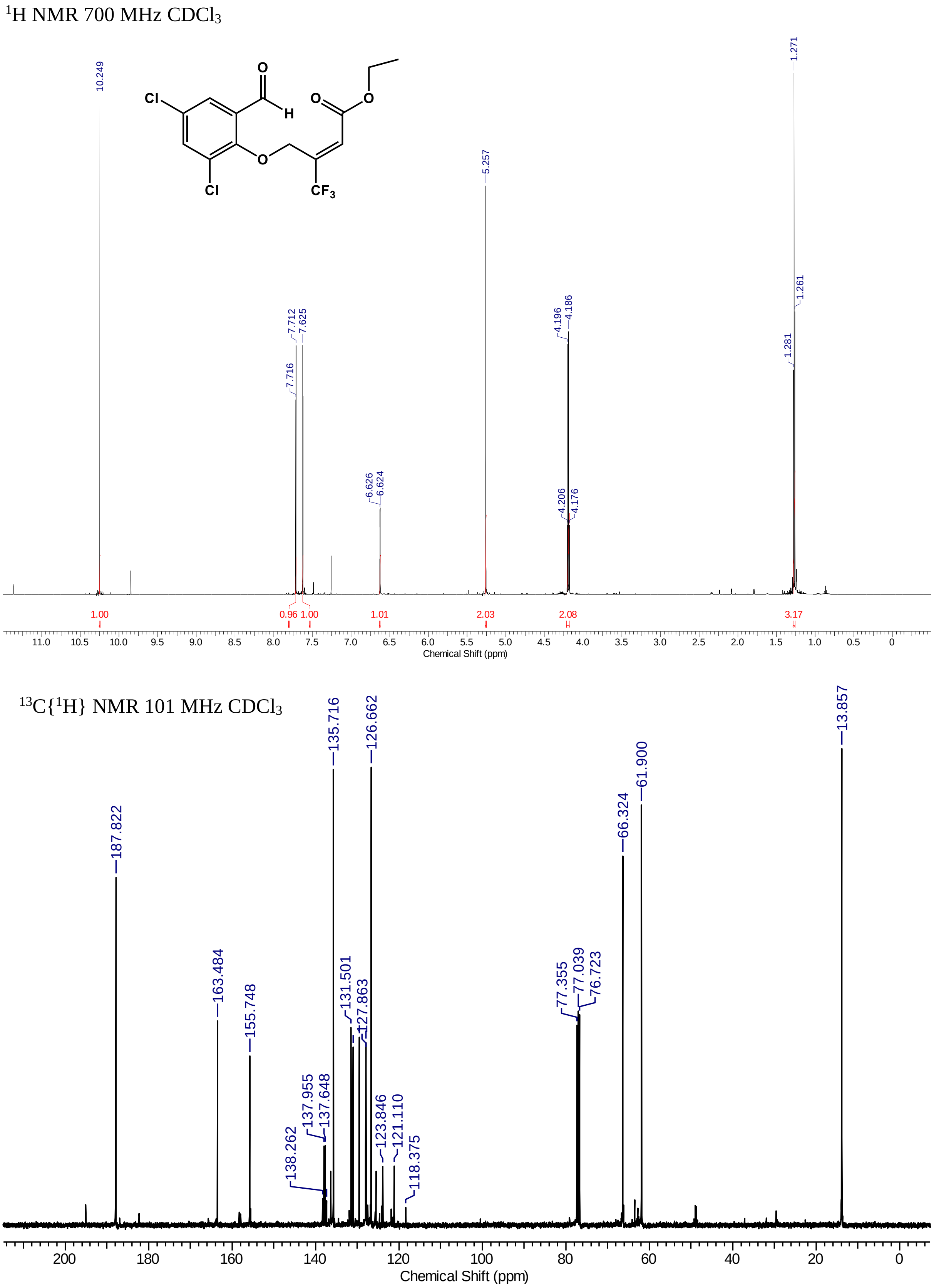

Figure S32. ^{1}H and ^{13}C NMR spectra of compound **1s**.

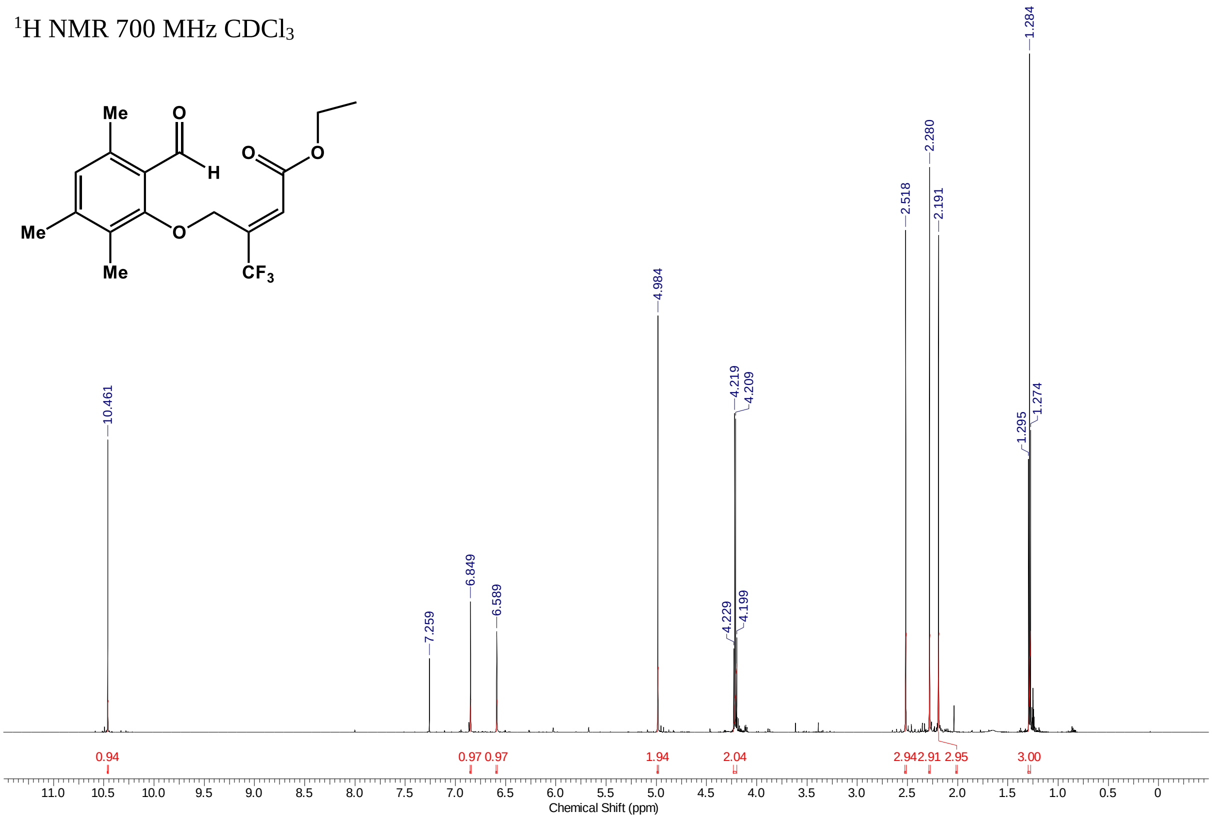

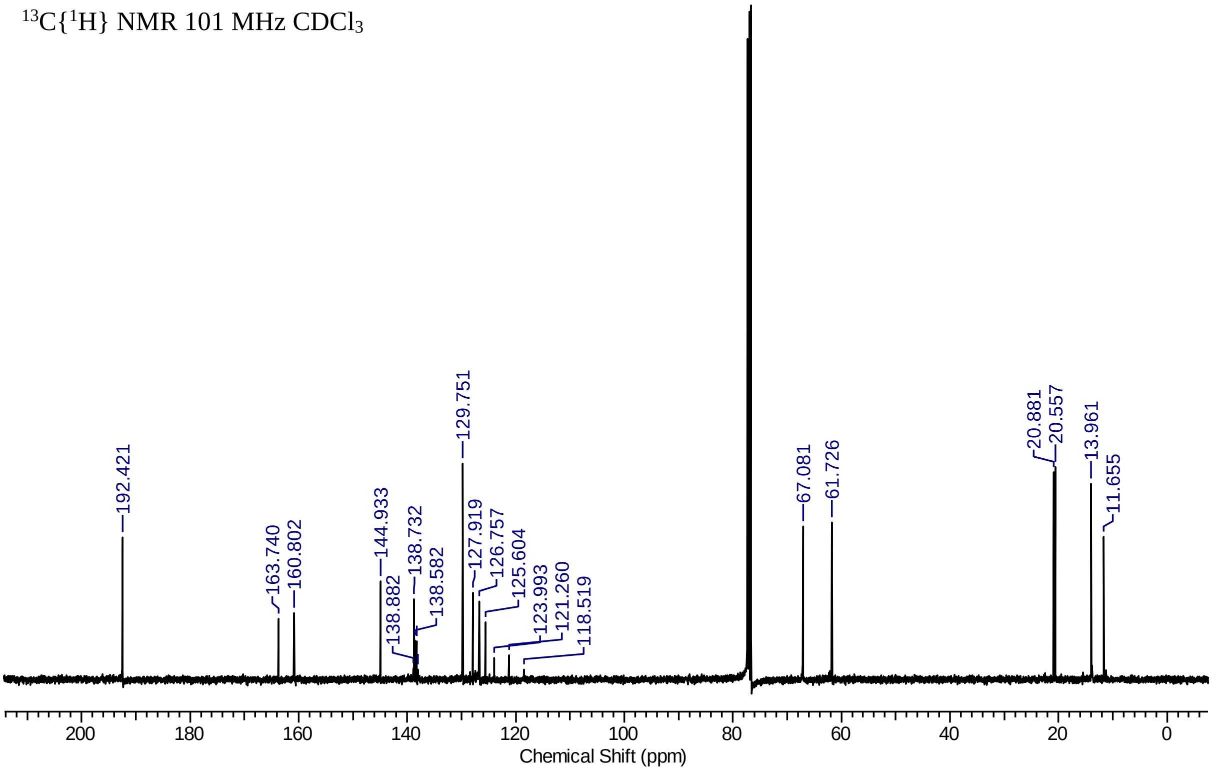

Figure S33. ^{1}H and ^{13}C NMR spectra of compound **1t**.

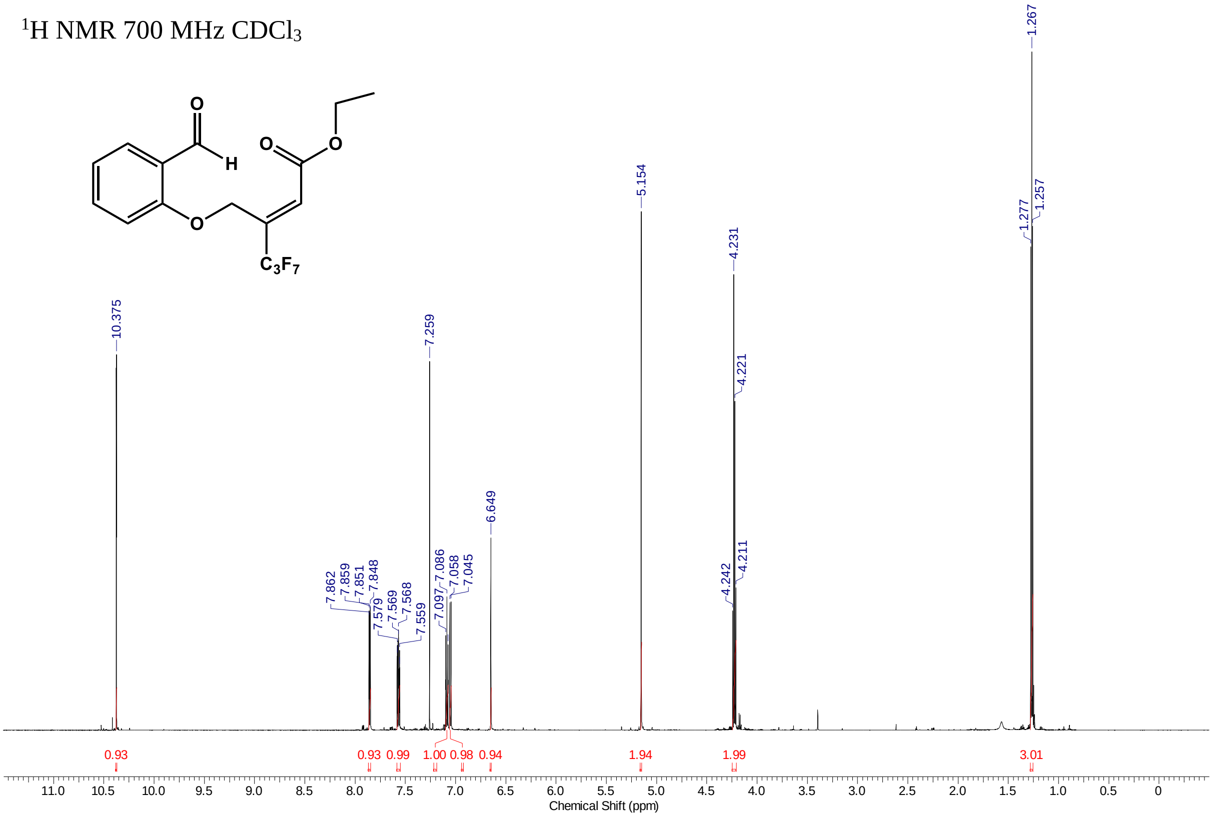

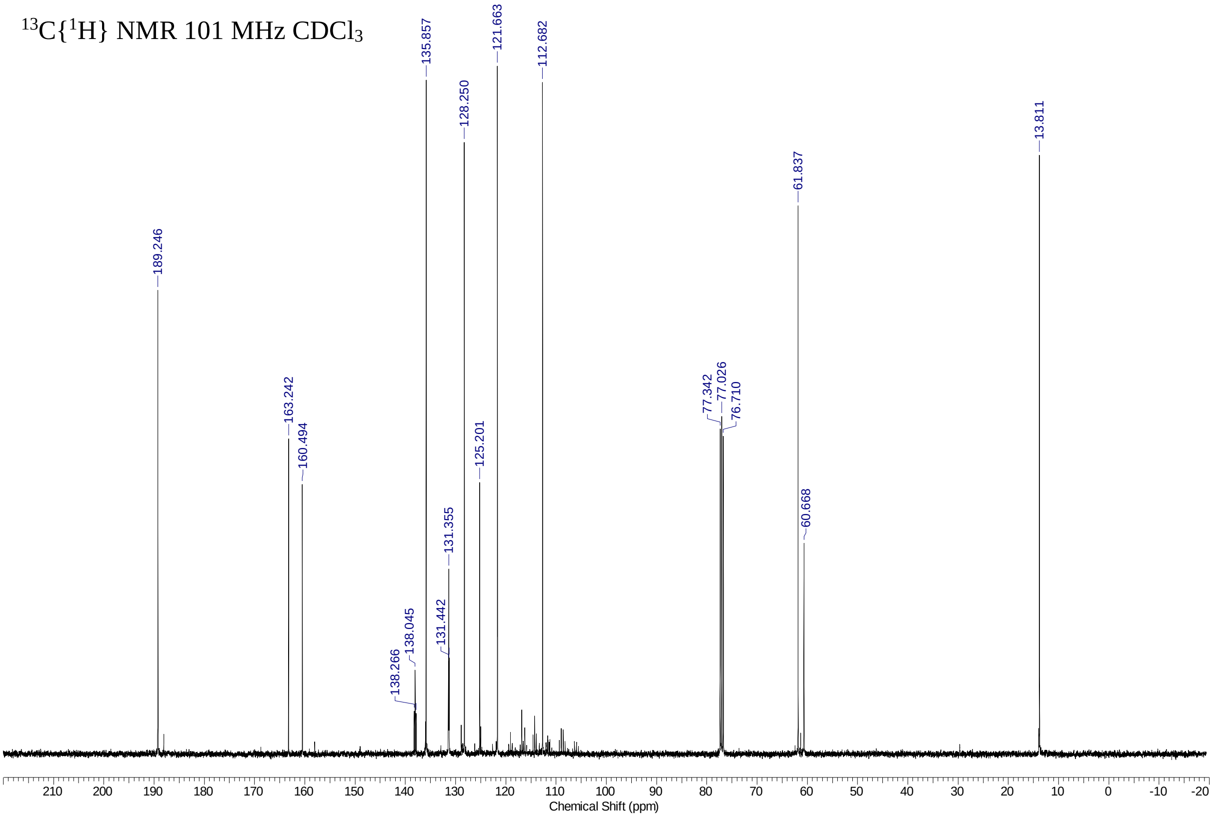

Figure S34. ^{1}H and ^{13}C NMR spectra of compound **1u.**

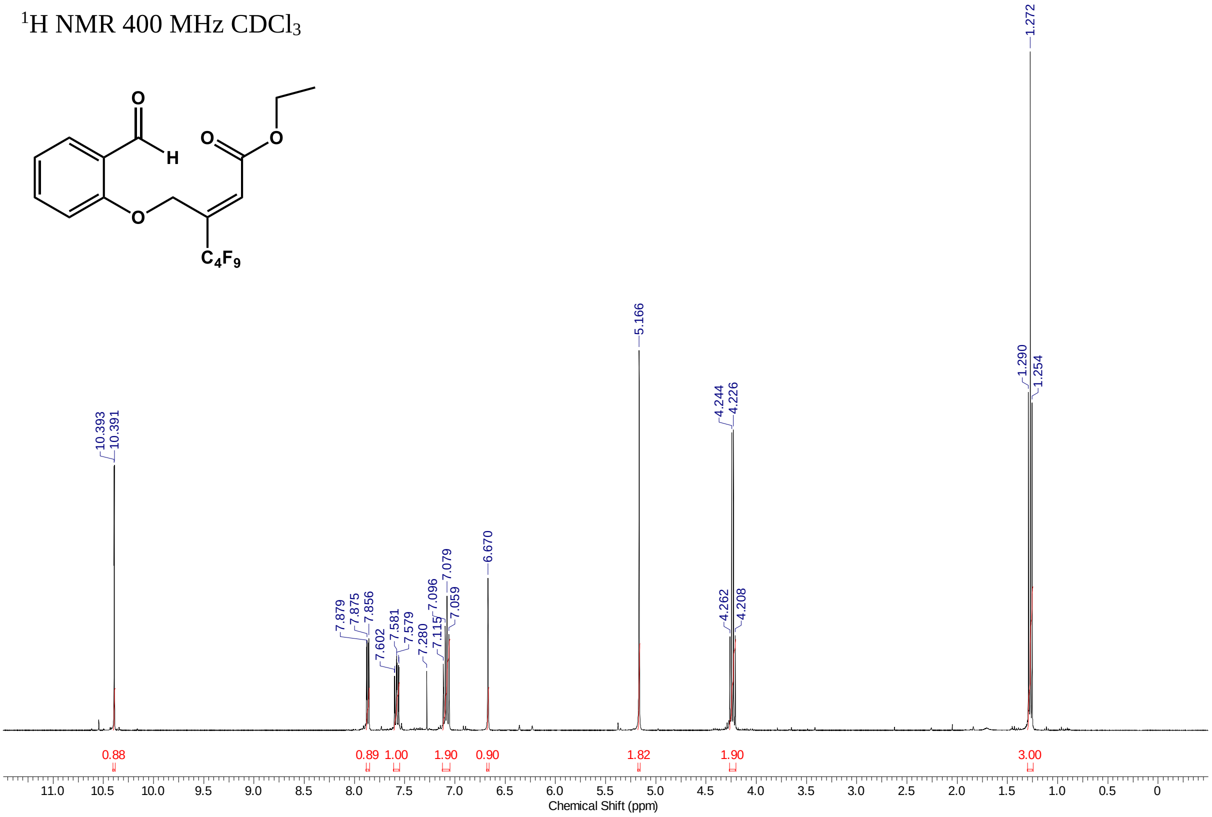

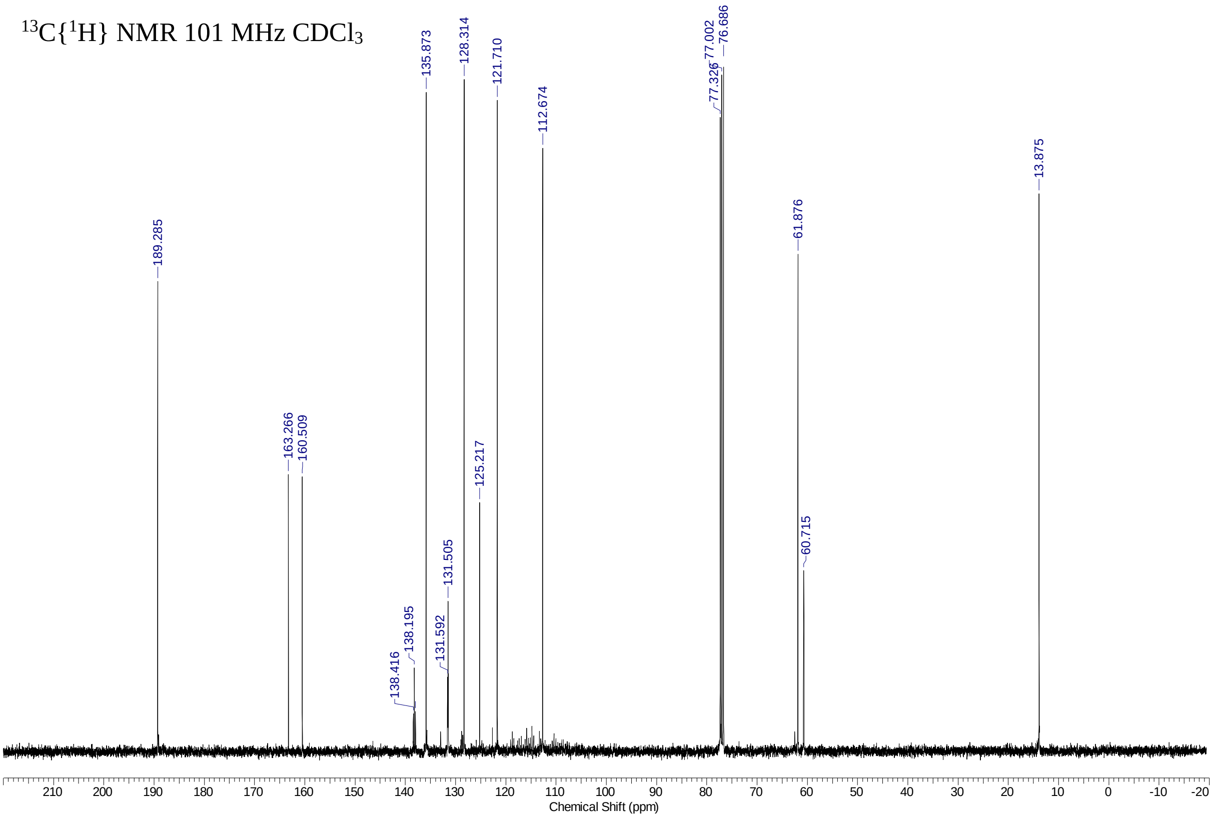

Figure S35. ^{1}H and ^{13}C NMR spectra of compound **1v.**

^{1}H NMR 700 MHz $CDCl_3$

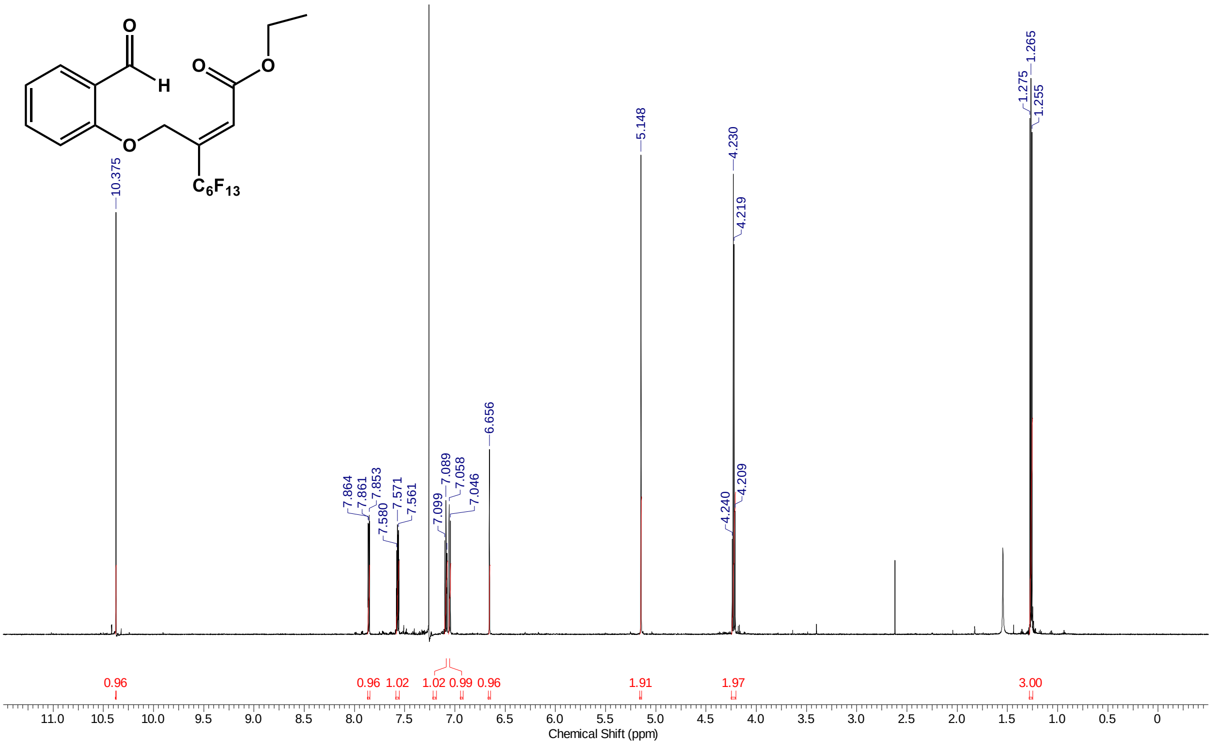

$^{13}C\{^{1}H\}$ NMR 101 MHz $CDCl_3$

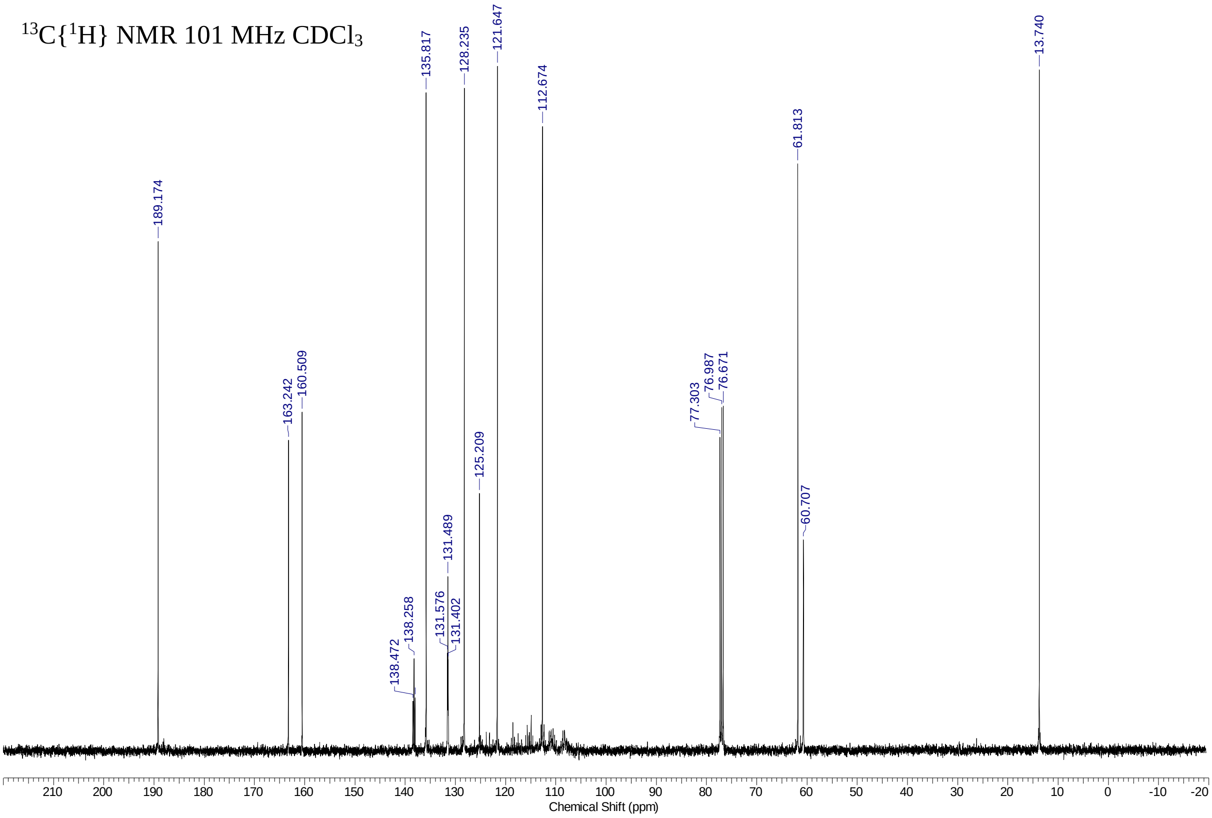

Figure S36. ^{1}H and ^{13}C NMR spectra of compound **1w.**

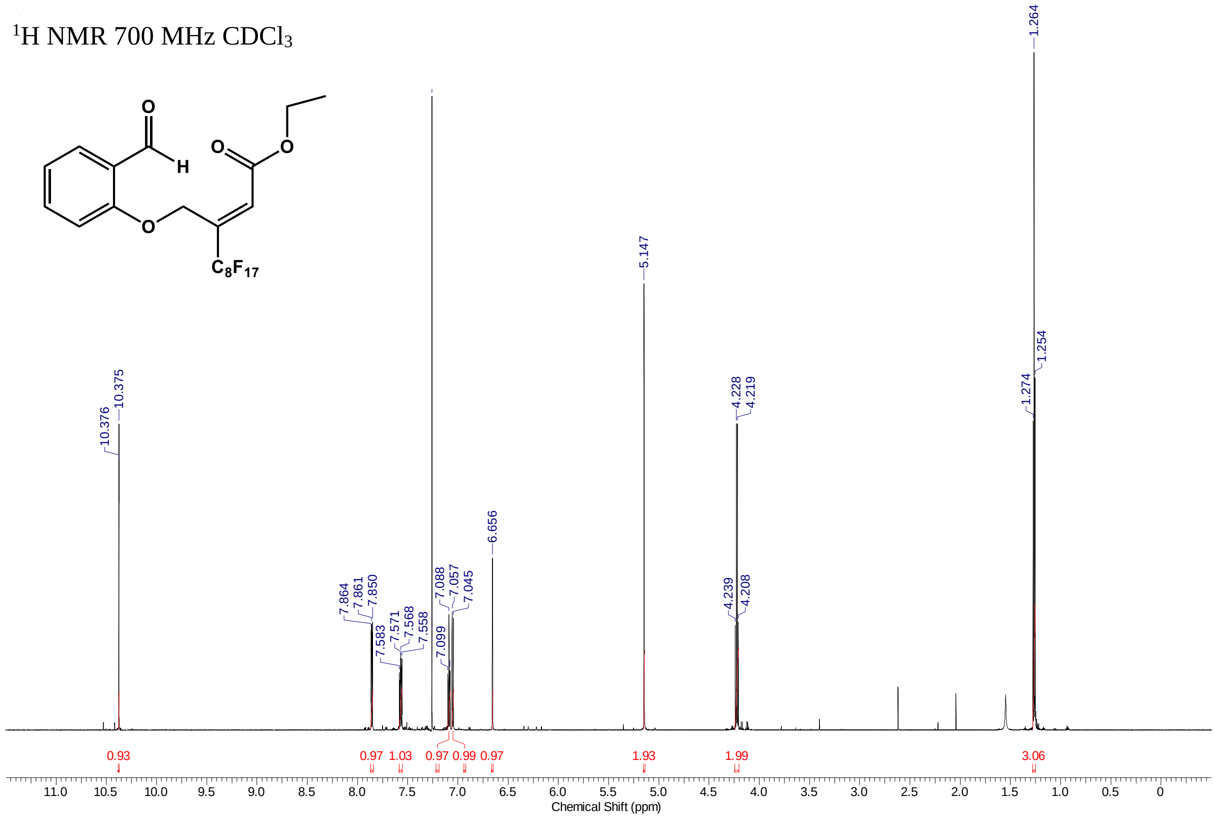

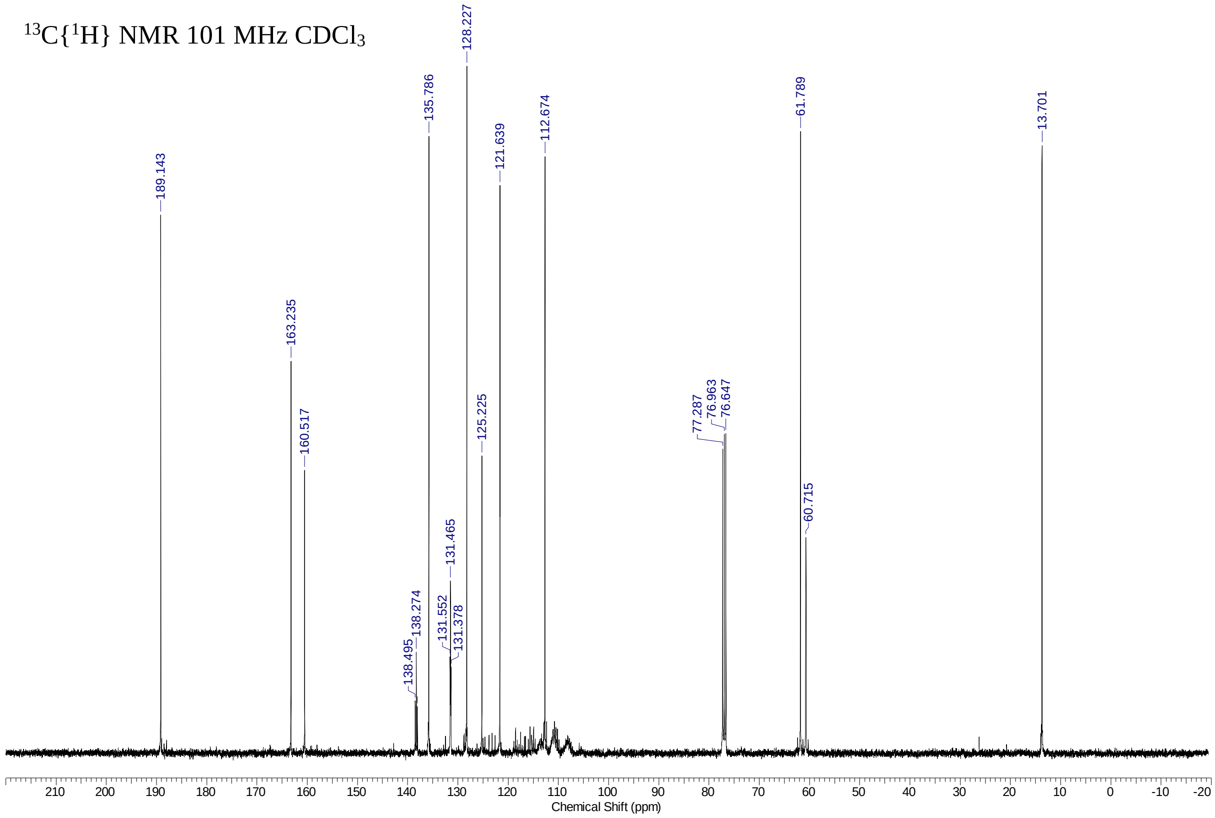

Figure S37. ^{1}H and ^{13}C NMR spectra of compound **1x.**

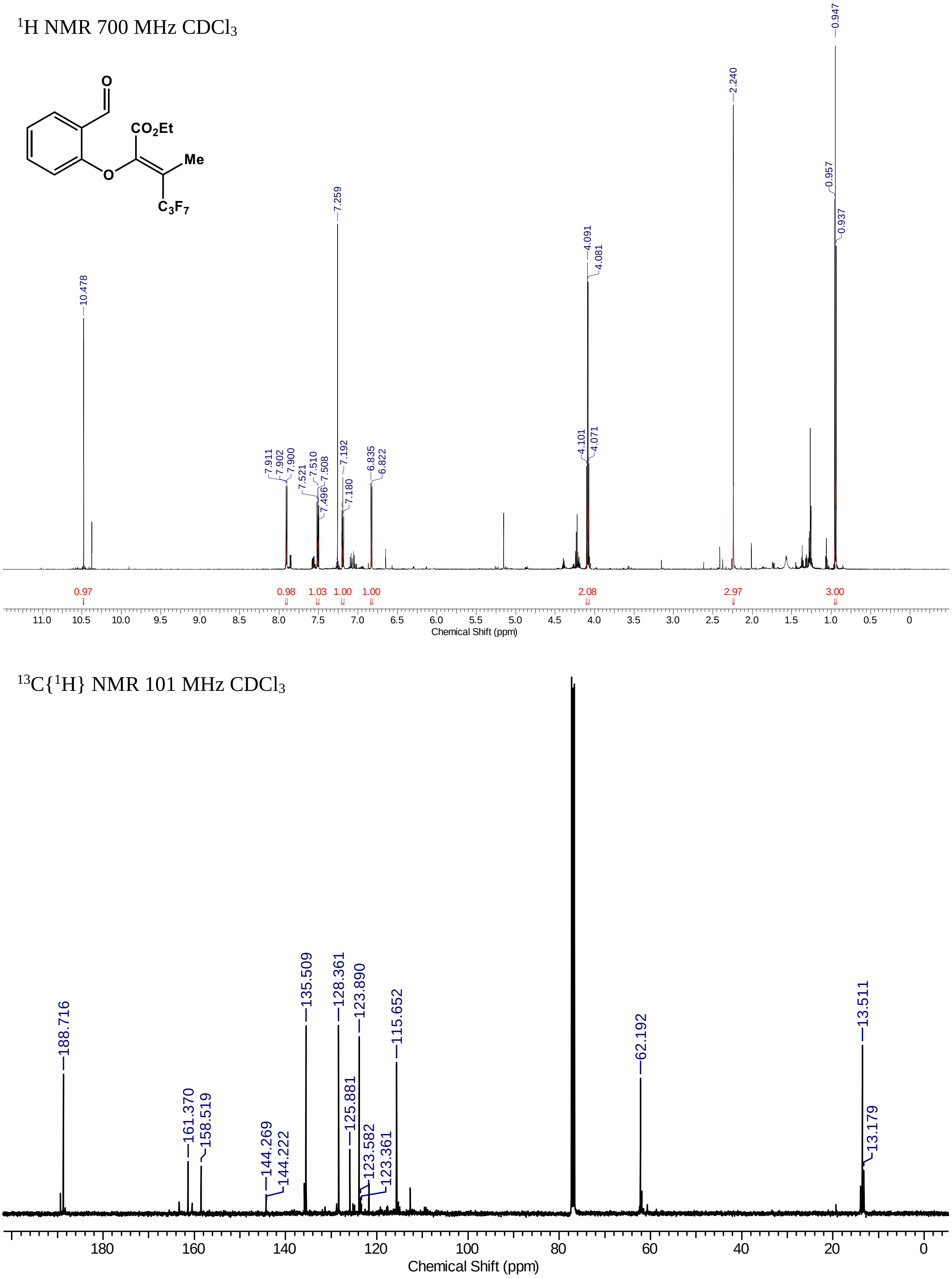

Figure S38. ^{1}H and ^{13}C NMR spectra of compound **3a.**

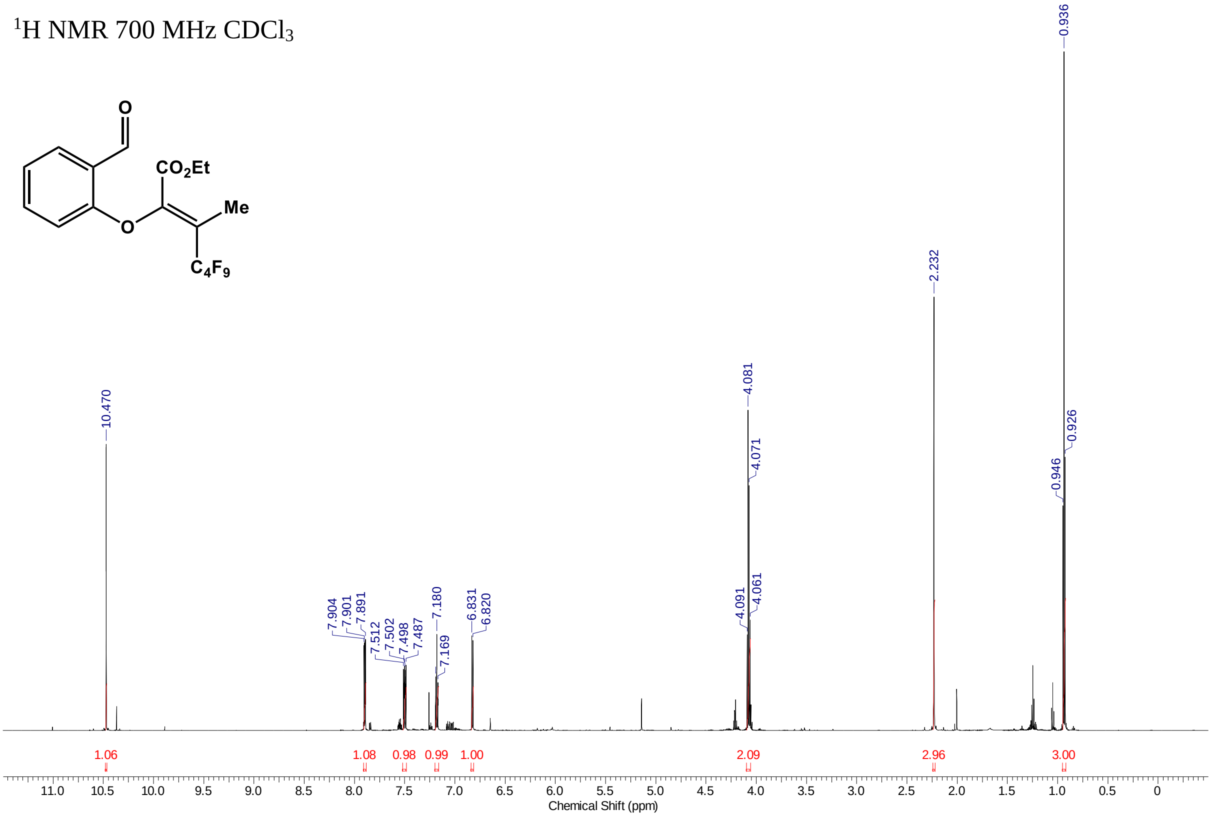

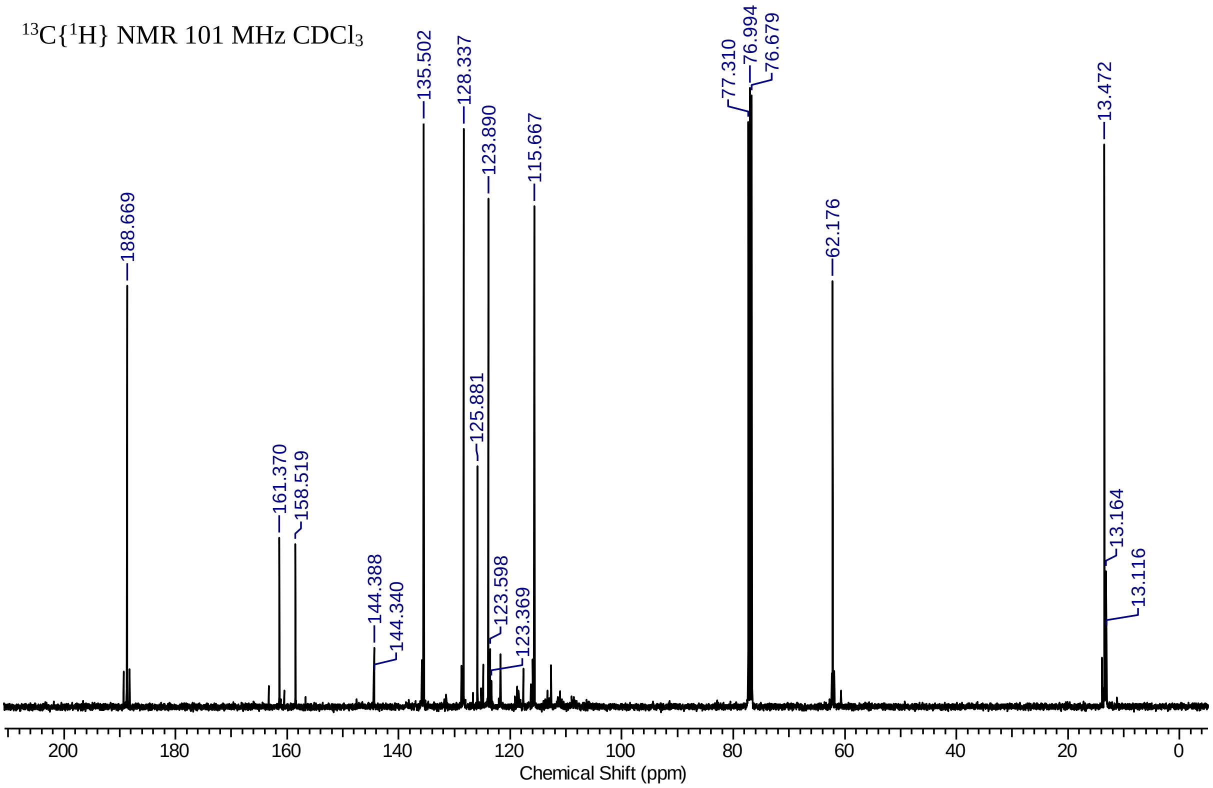

Figure S39. ^{1}H and ^{13}C NMR spectra of compound **3b.**

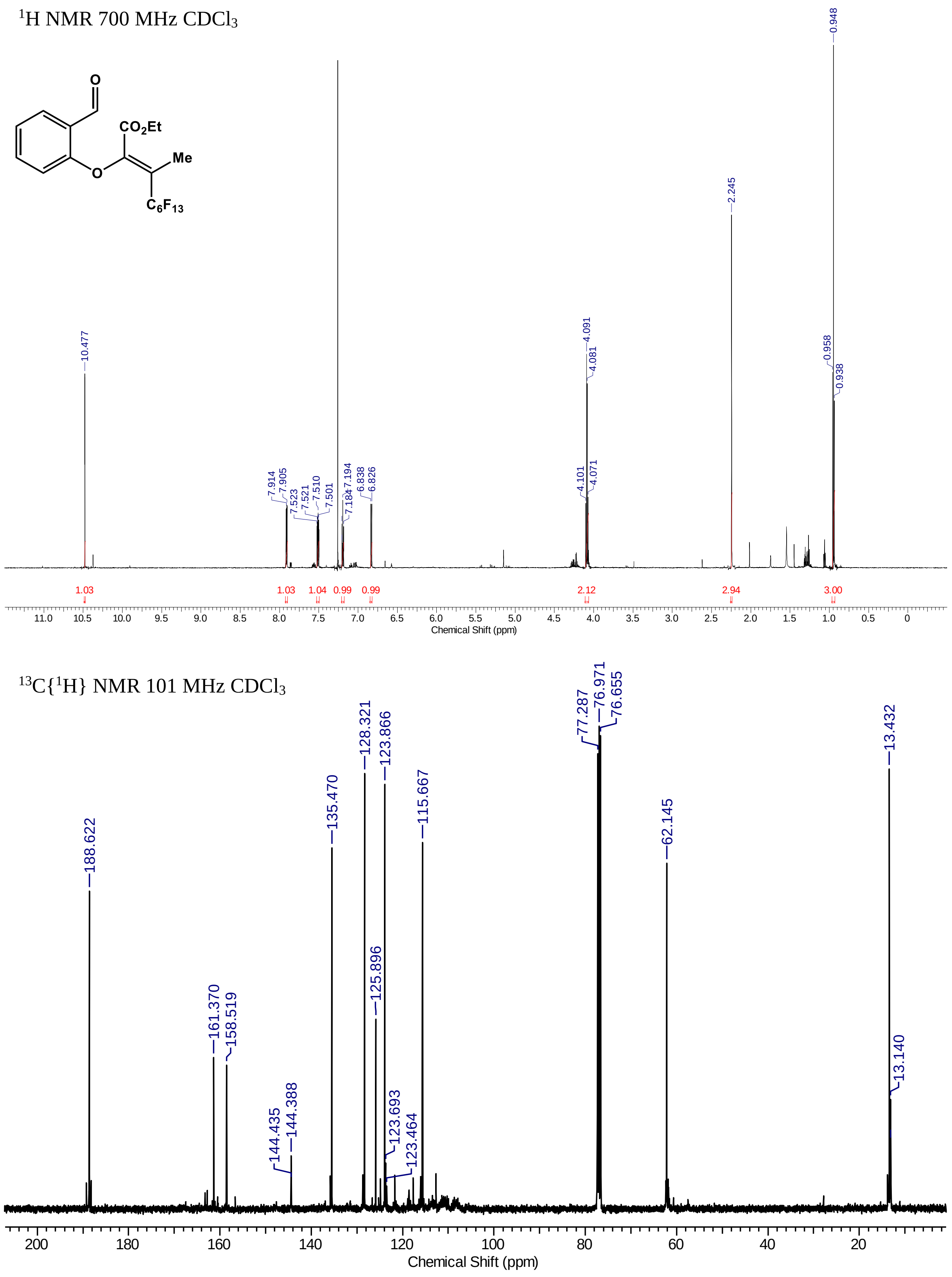

Figure S40. ^1H and ^{13}C NMR spectra of compound **3c**.

^{1}H NMR 700 MHz $CDCl_3$

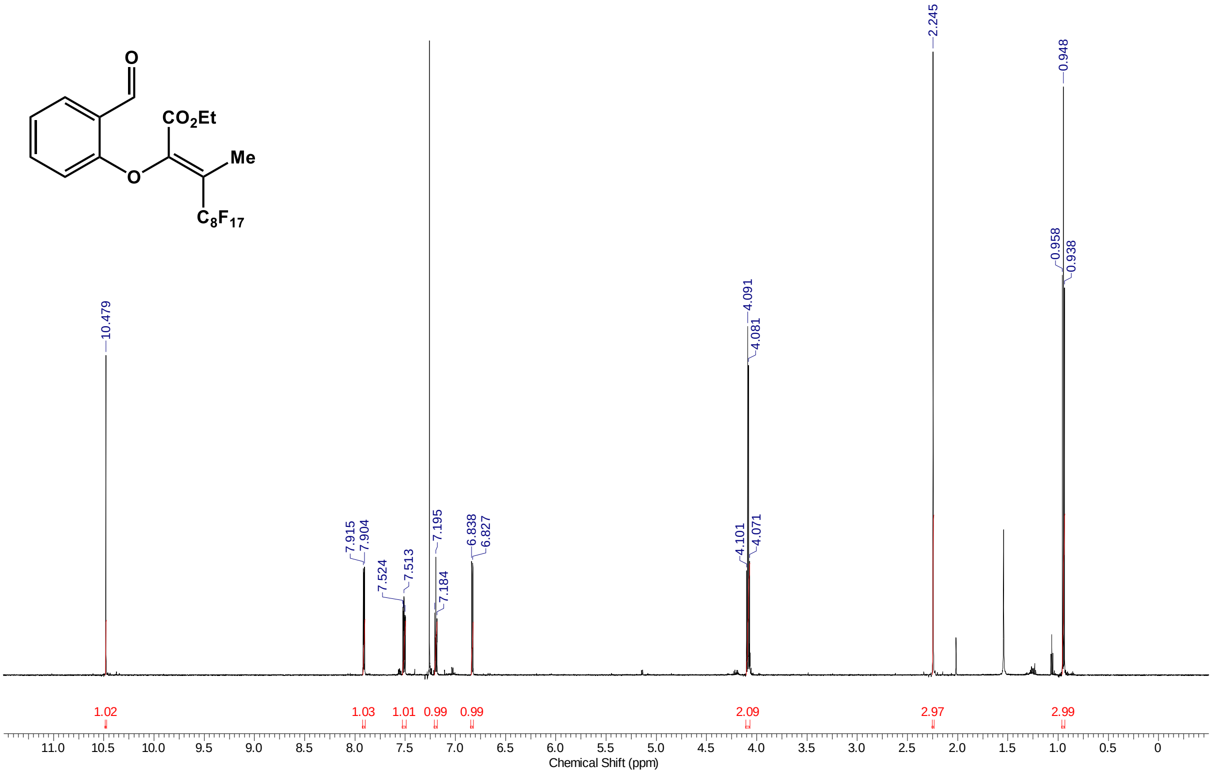

$^{13}C\{^{1}H\}$ NMR 101 MHz $CDCl_3$

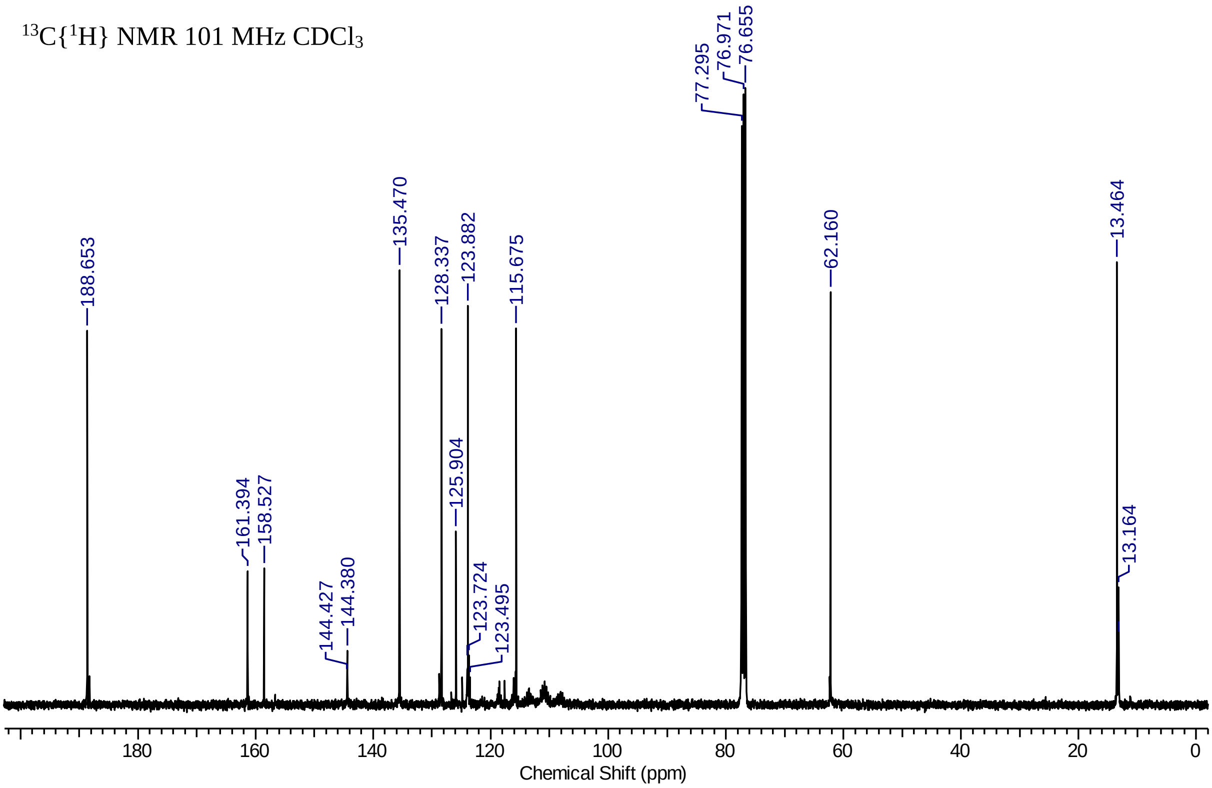

Figure S41. ^{1}H and ^{13}C NMR spectra of compound **3d.**

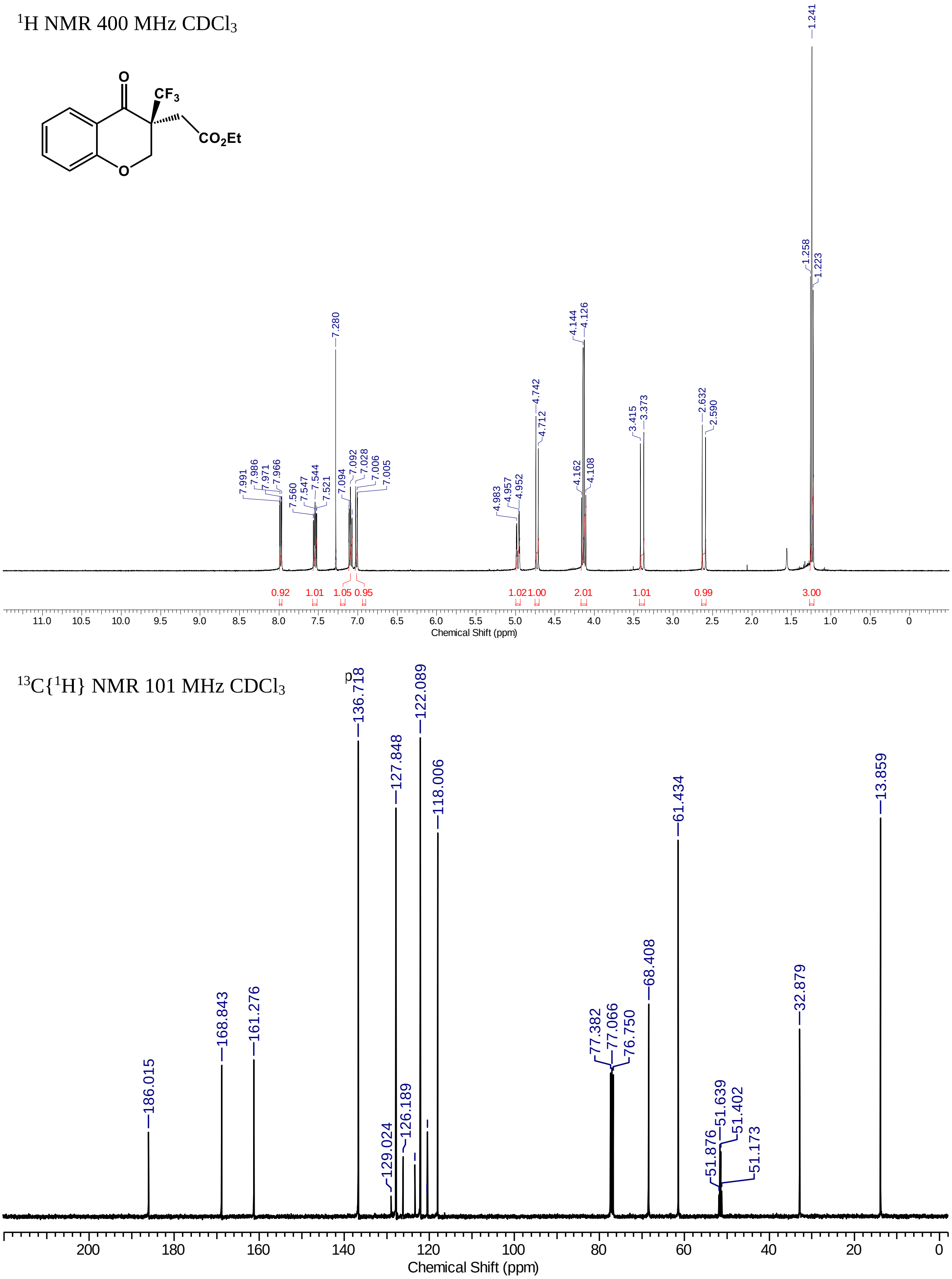

Figure S42. ^{1}H and ^{13}C NMR spectra of compound **2a.**

^{1}H NMR 700 MHz $CDCl_3$

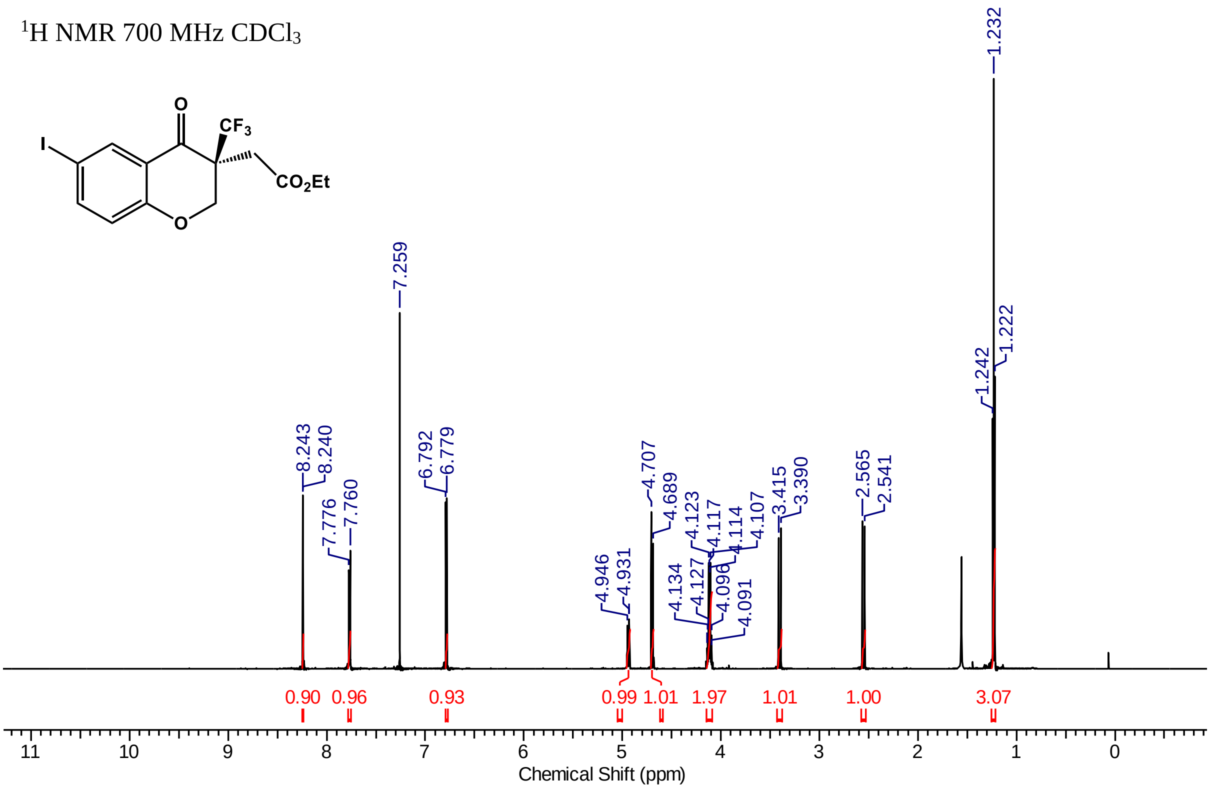

^{13}C{^{1}H} NMR 176 MHz $CDCl_3$

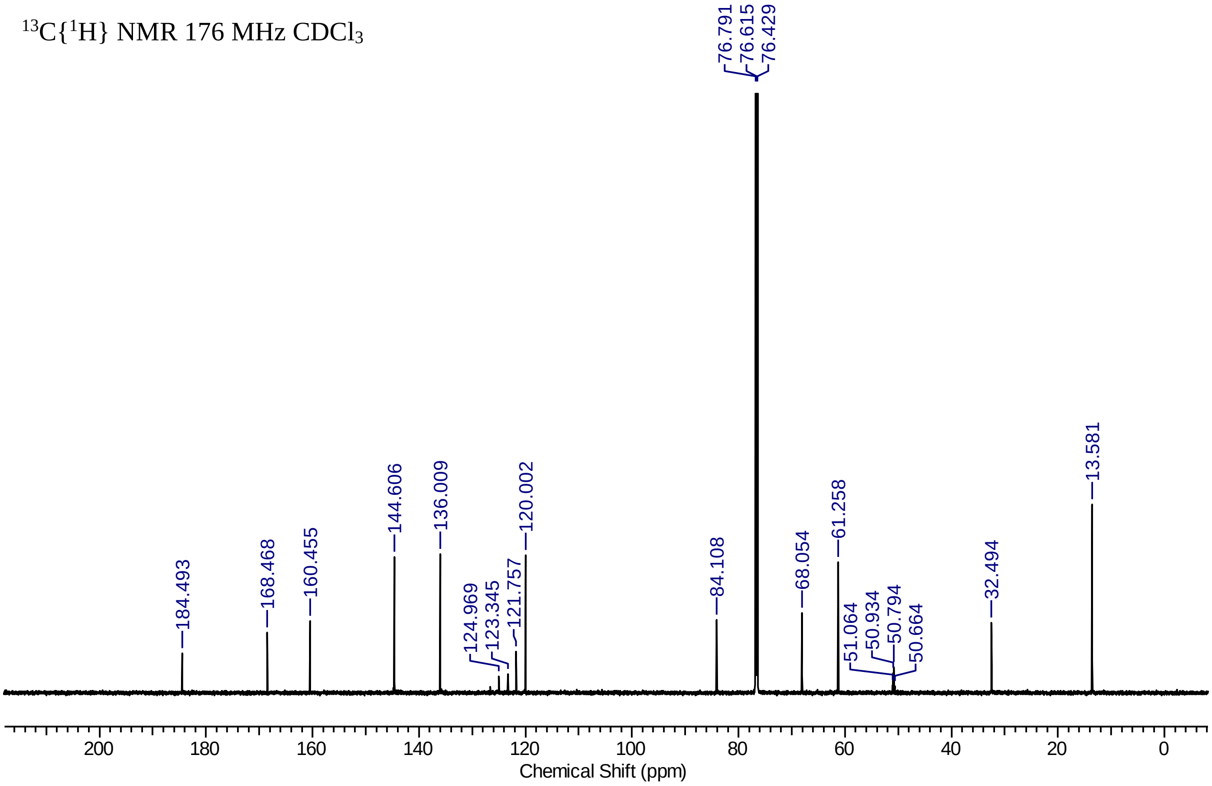

Figure S43. ^{1}H and ^{13}C NMR spectra of compound **2b.**

^{1}H NMR 400 MHz $CDCl_3$

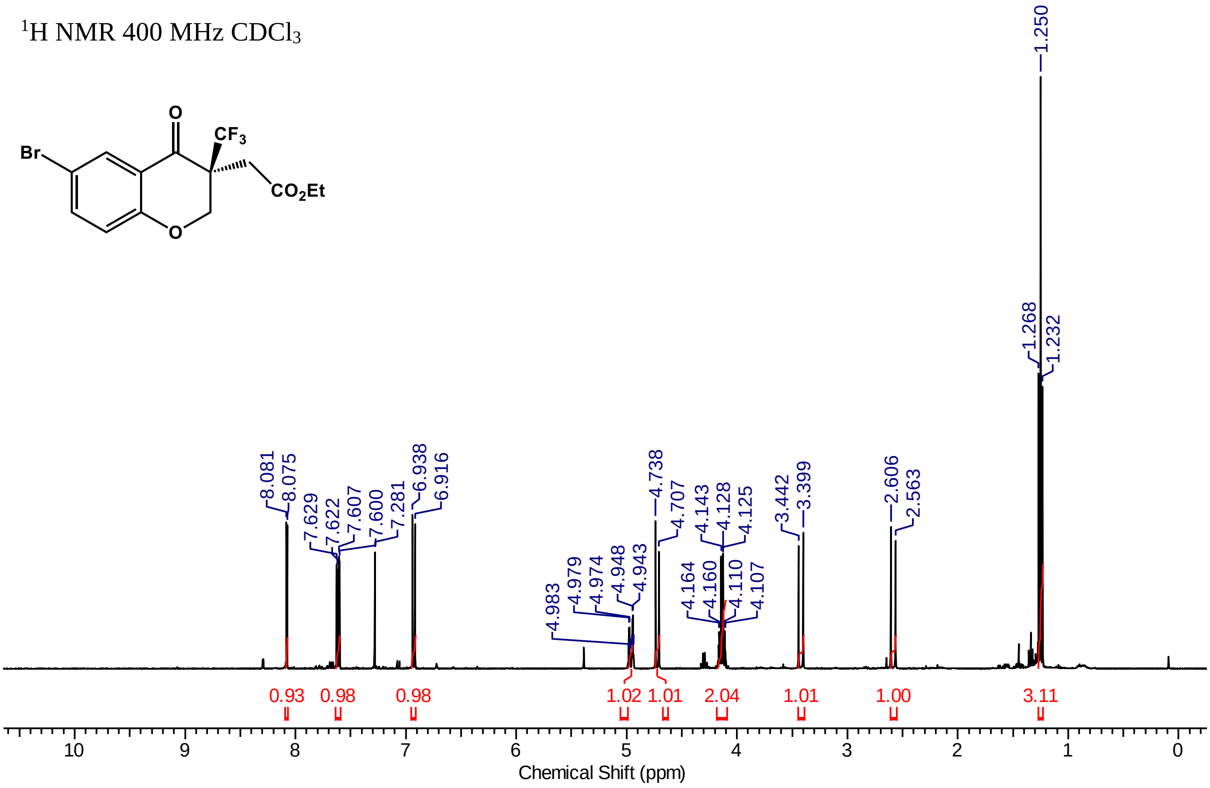

$^{13}C\{^{1}H\}$ NMR 101 MHz $CDCl_3$

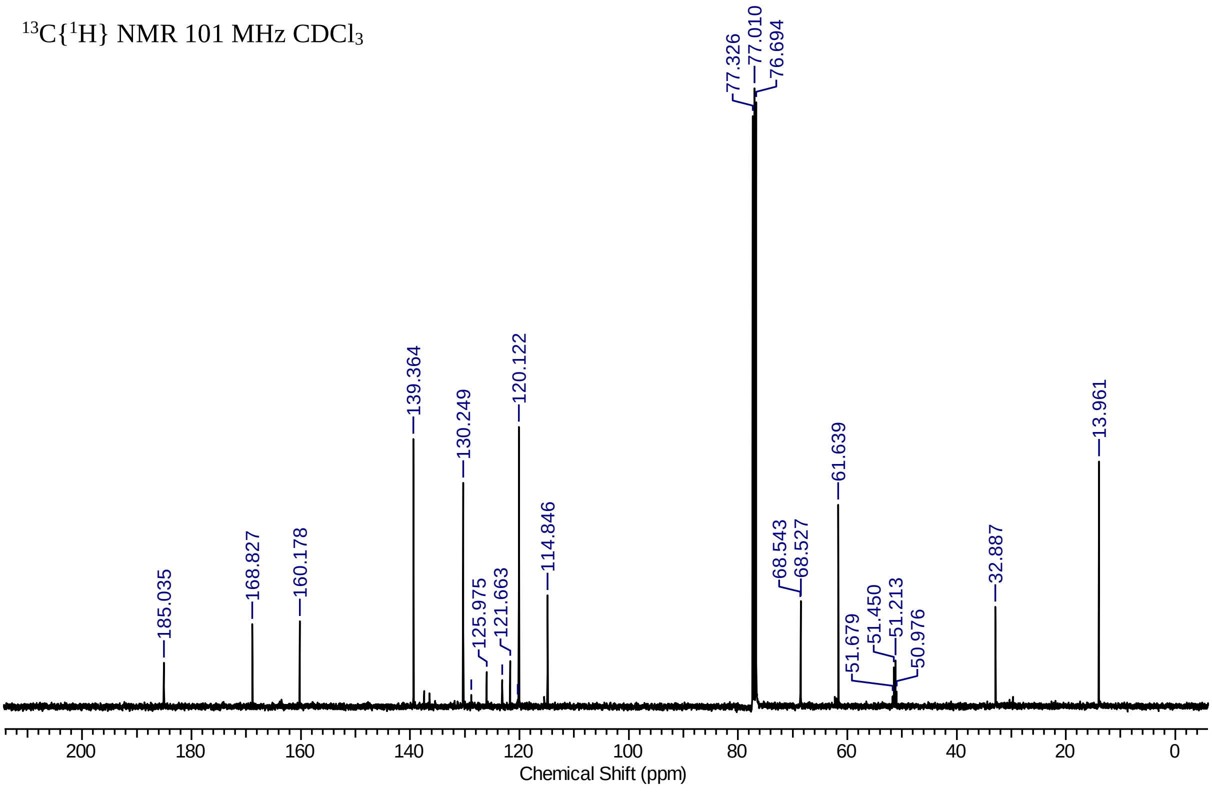

Figure S44. ^{1}H and ^{13}C NMR spectra of compound **2c.**

^{1}H NMR 700 MHz $CDCl_3$

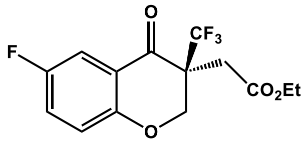

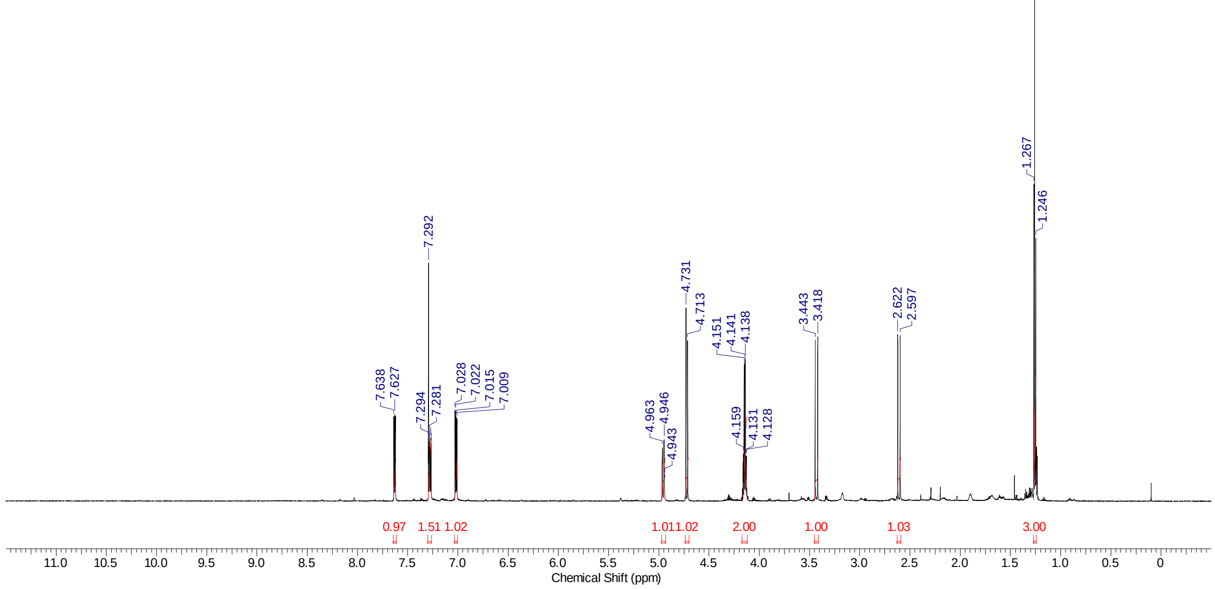

$^{13}C\{^{1}H\}$ NMR 101 MHz $CDCl_3$

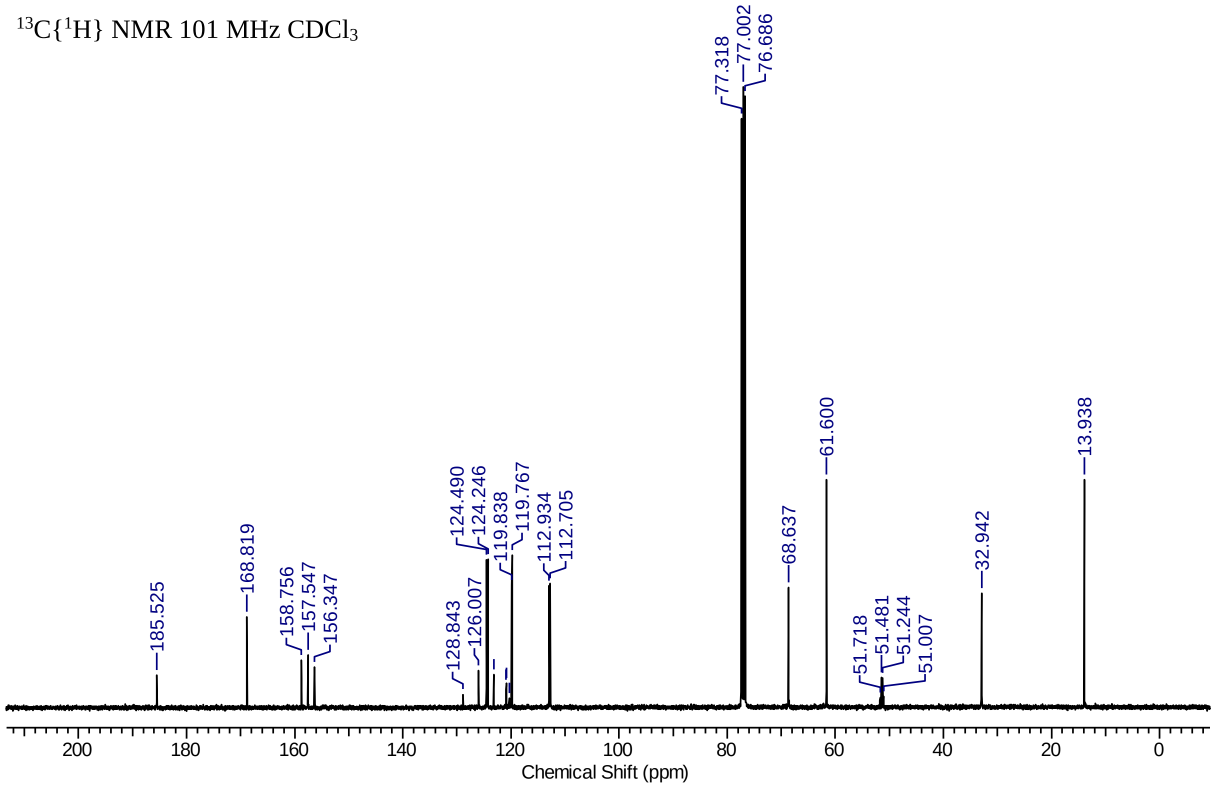

Figure S45. ^{1}H and ^{13}C NMR spectra of compound **2d.**

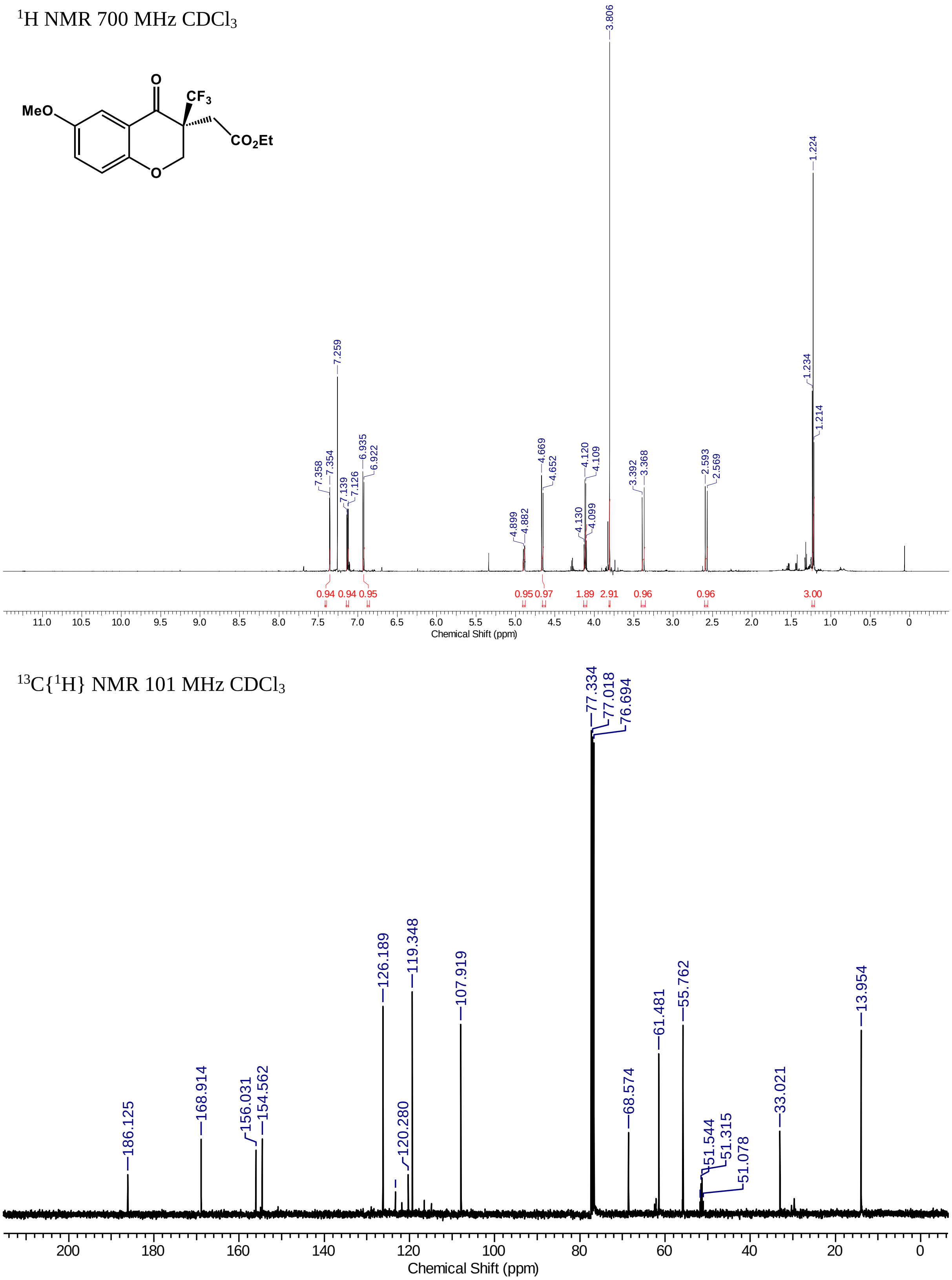

Figure S46. ^{1}H and ^{13}C NMR spectra of compound **2e.**

^{1}H NMR 700 MHz $CDCl_3$

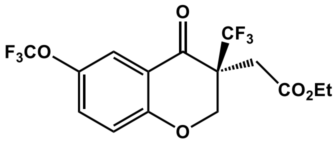

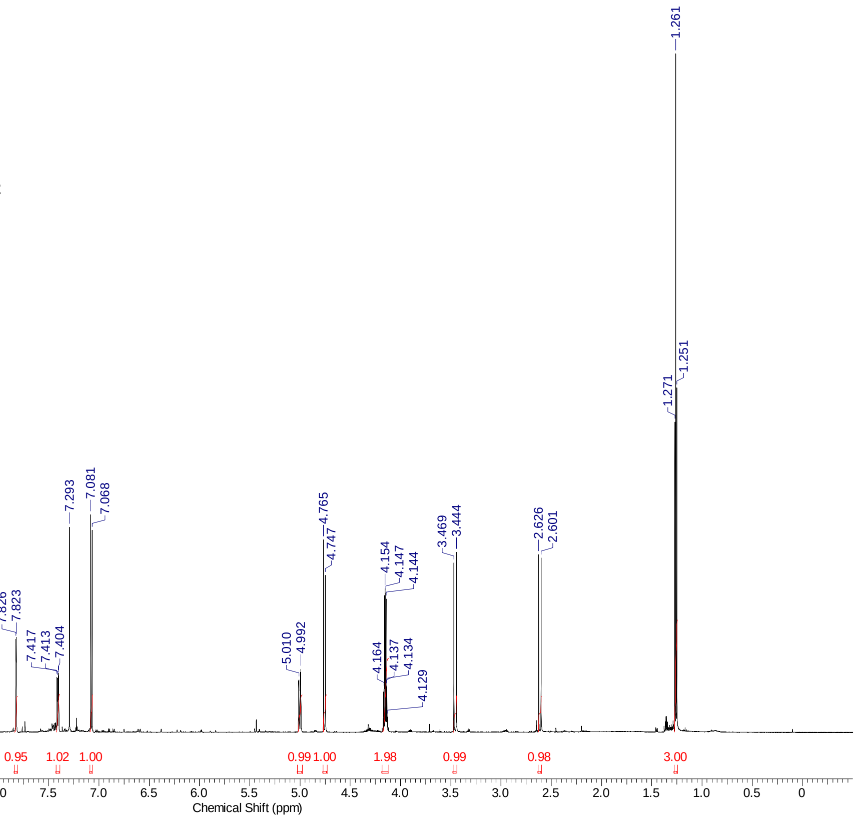

^{13}C{^{1}H} NMR 176 MHz $CDCl_3$

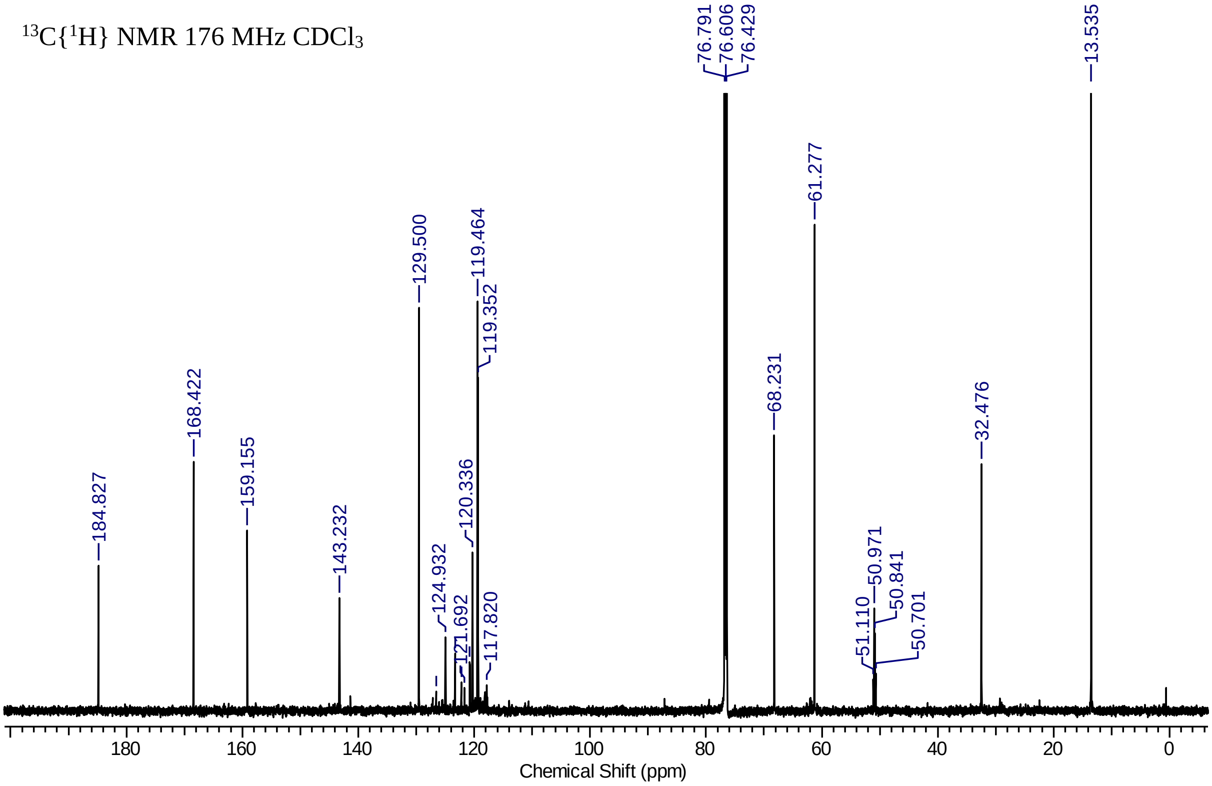

Figure S47. ^{1}H and ^{13}C NMR spectra of compound **2f.**

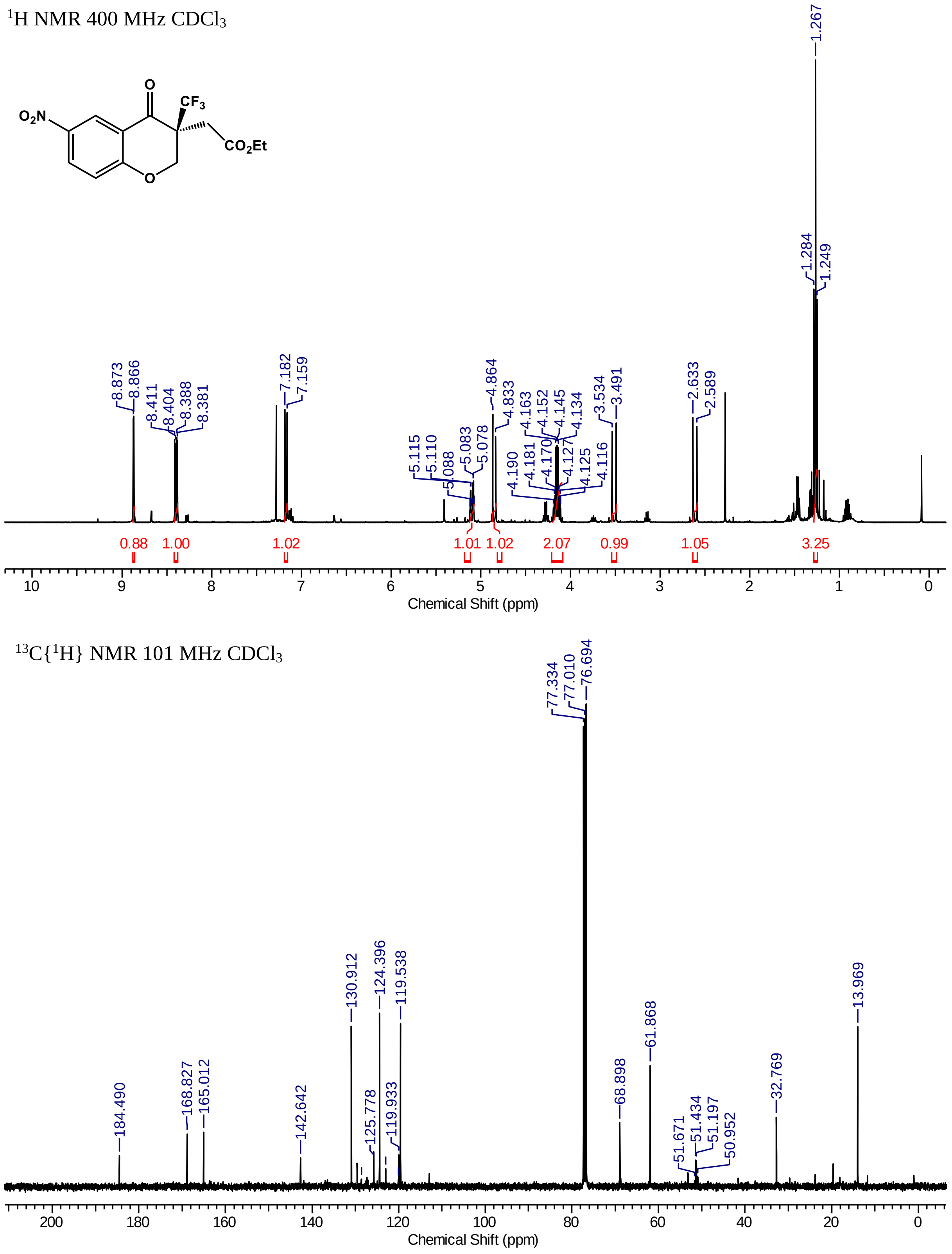

Figure S48. ^{1}H and ^{13}C NMR spectra of compound **2g.**

^{1}H NMR 700 MHz $CDCl_3$

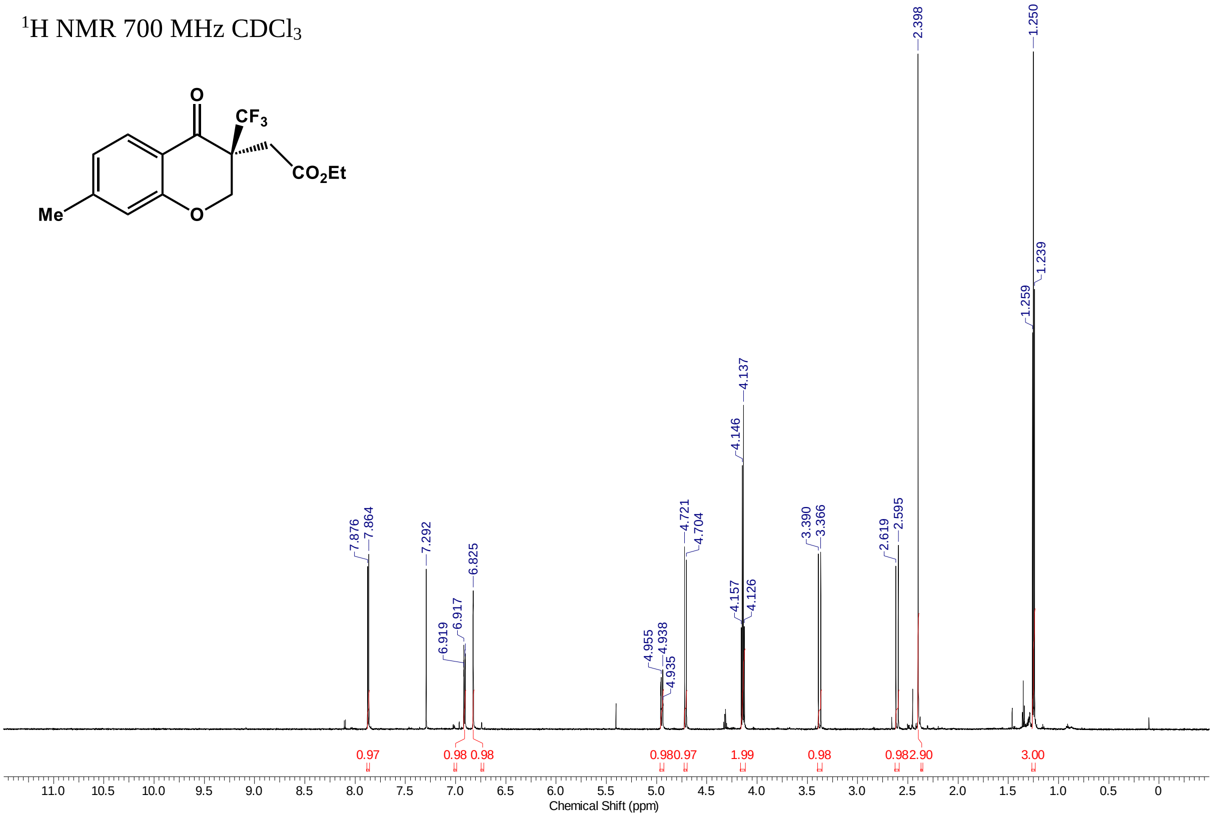

$^{13}C\{^1H\}$ NMR 101 MHz $CDCl_3$

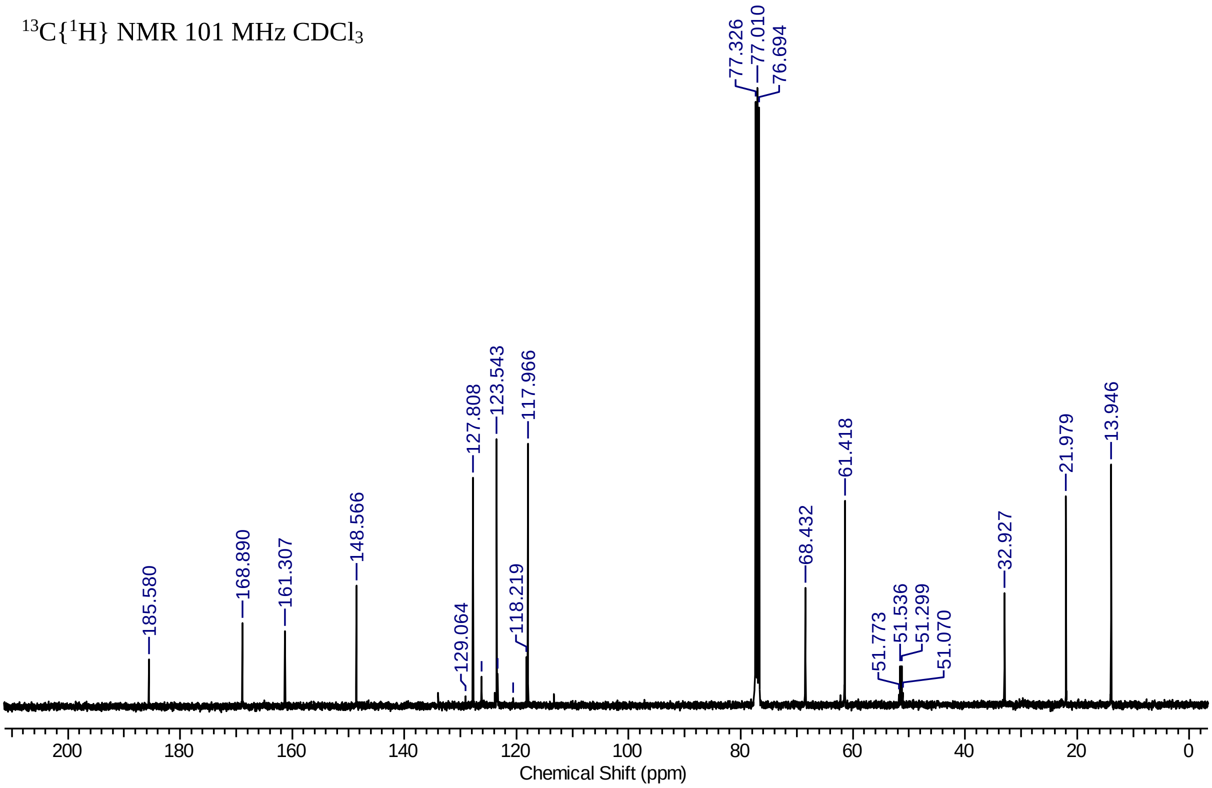

Figure S49. ^{1}H and ^{13}C NMR spectra of compound **2h.**

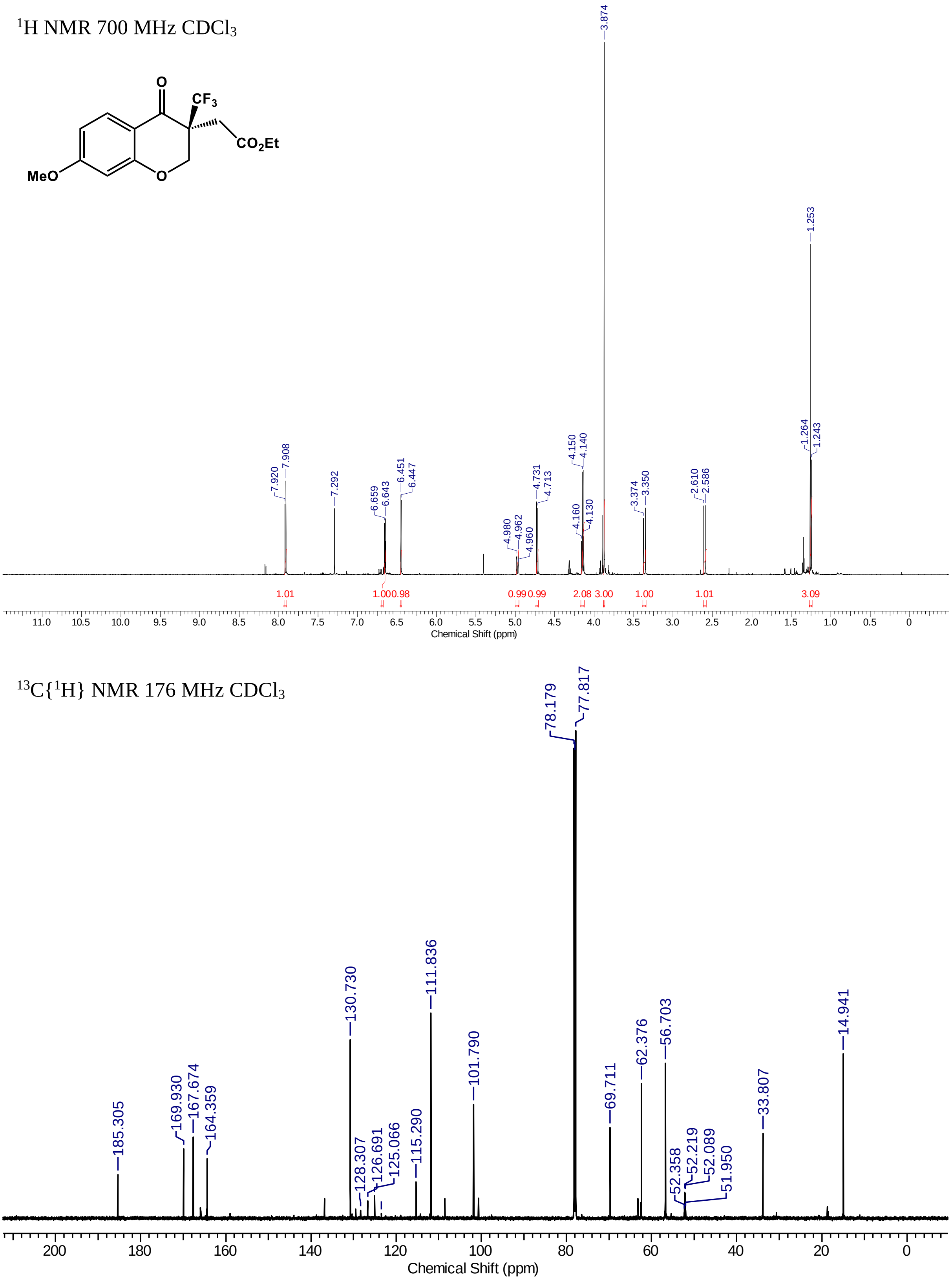

Figure S50. ^{1}H and ^{13}C NMR spectra of compound **2i.**

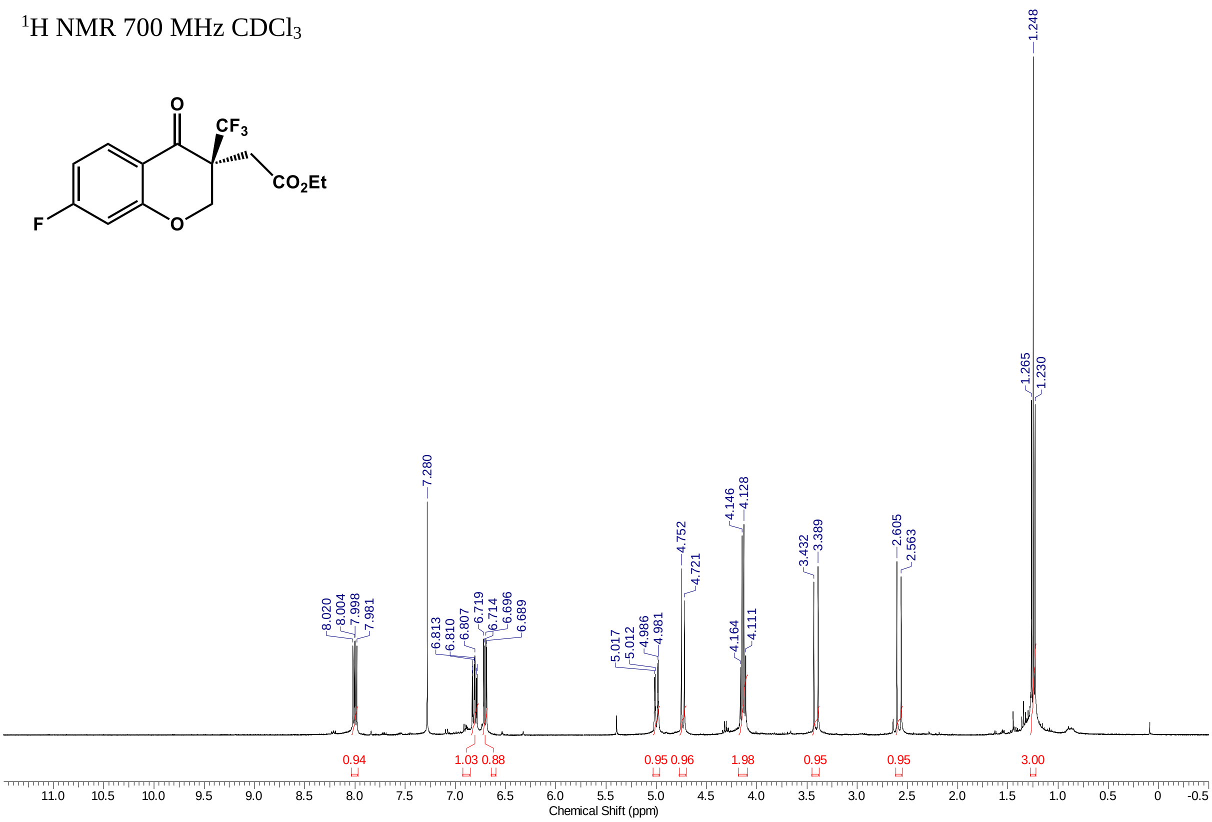

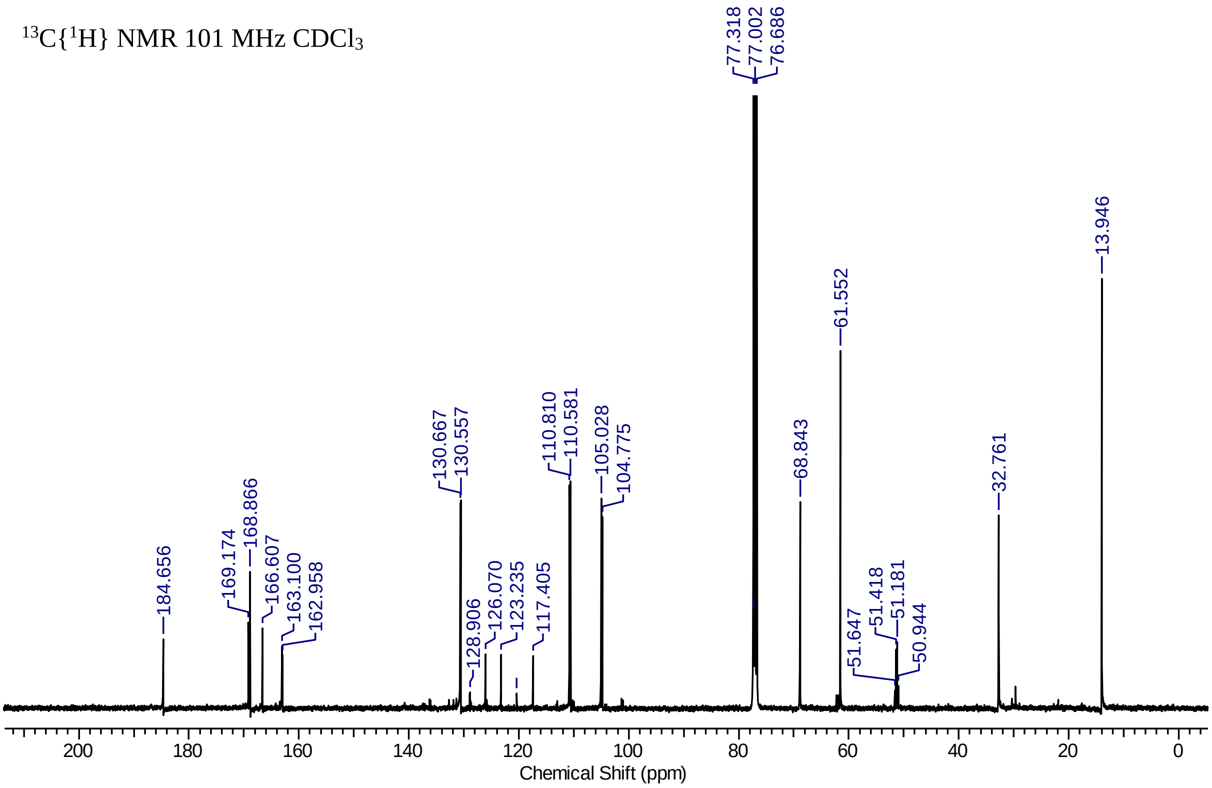

Figure S51. ^{1}H and ^{13}C NMR spectra of compound **2j.**

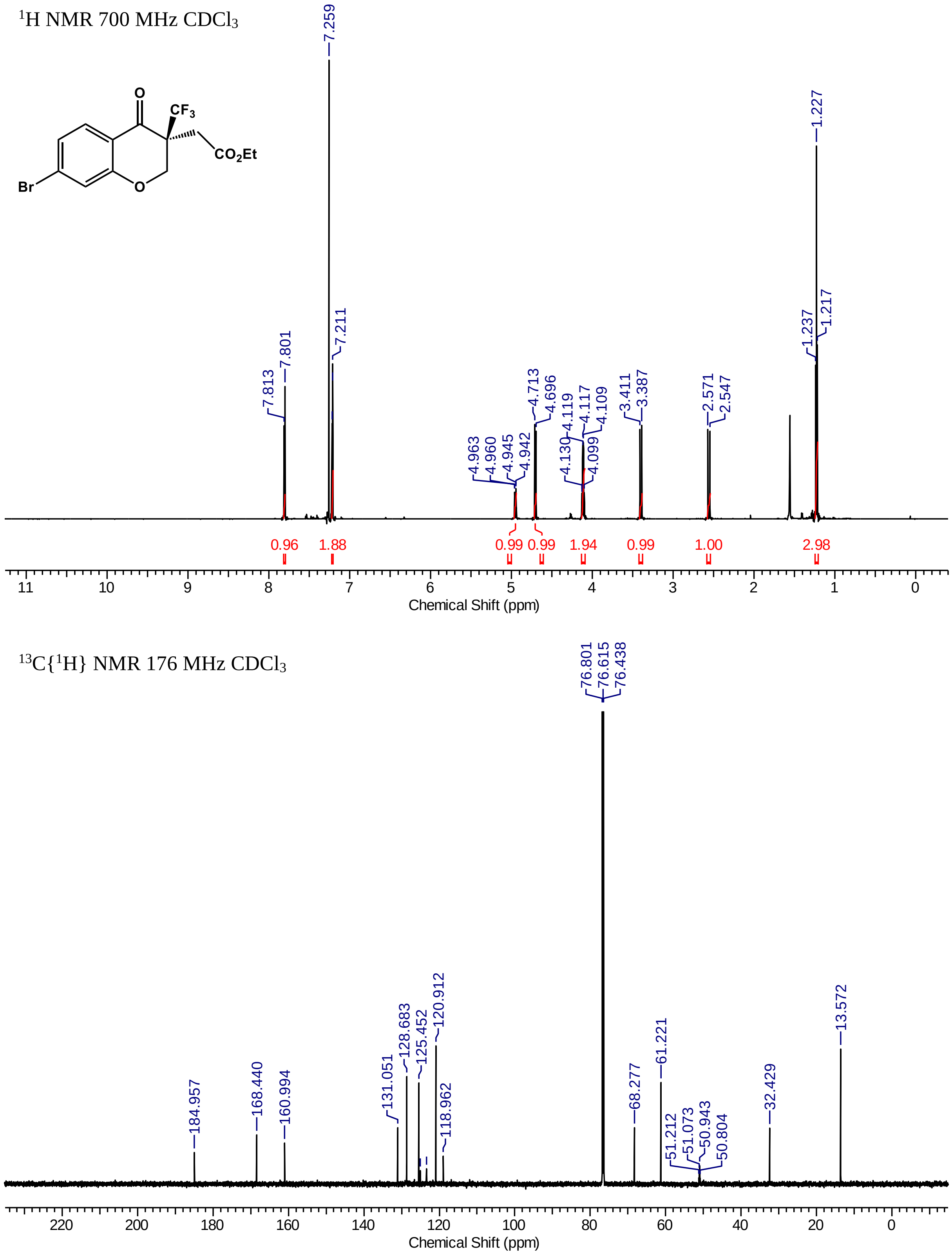

Figure S52. ^{1}H and ^{13}C NMR spectra of compound **2k.**

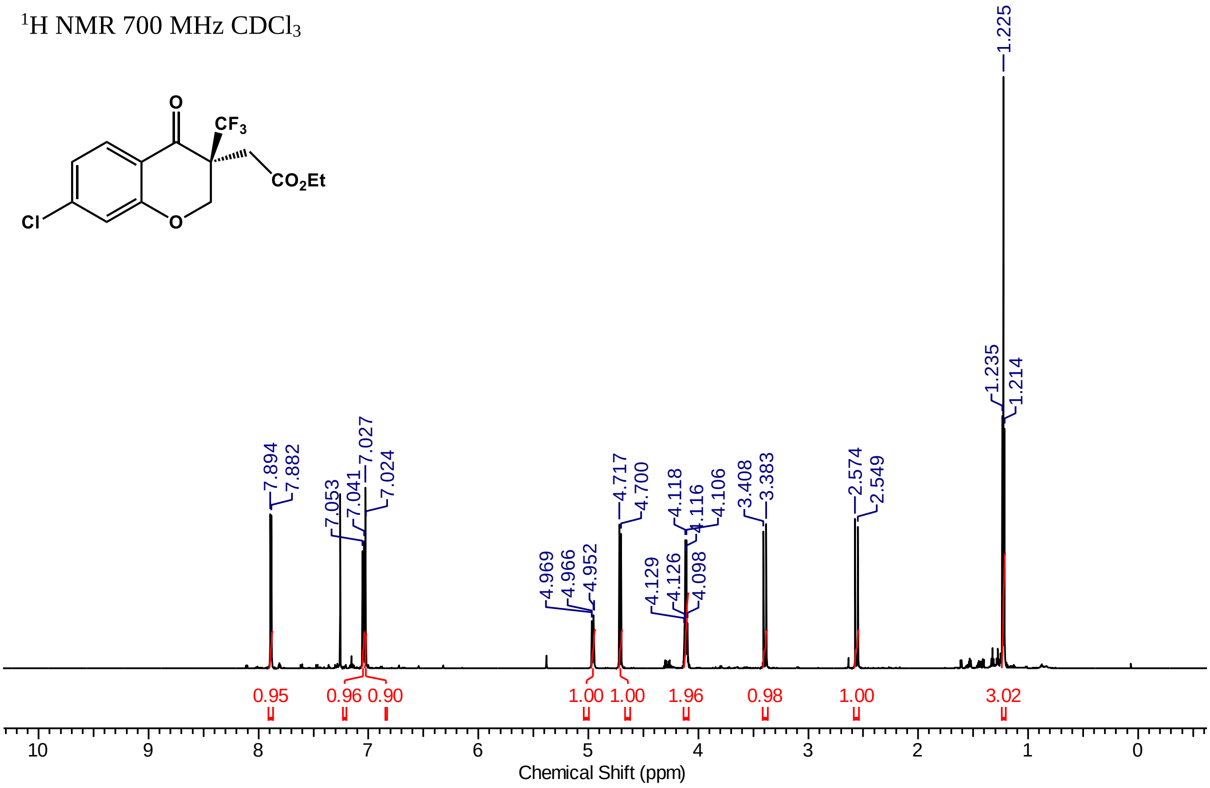

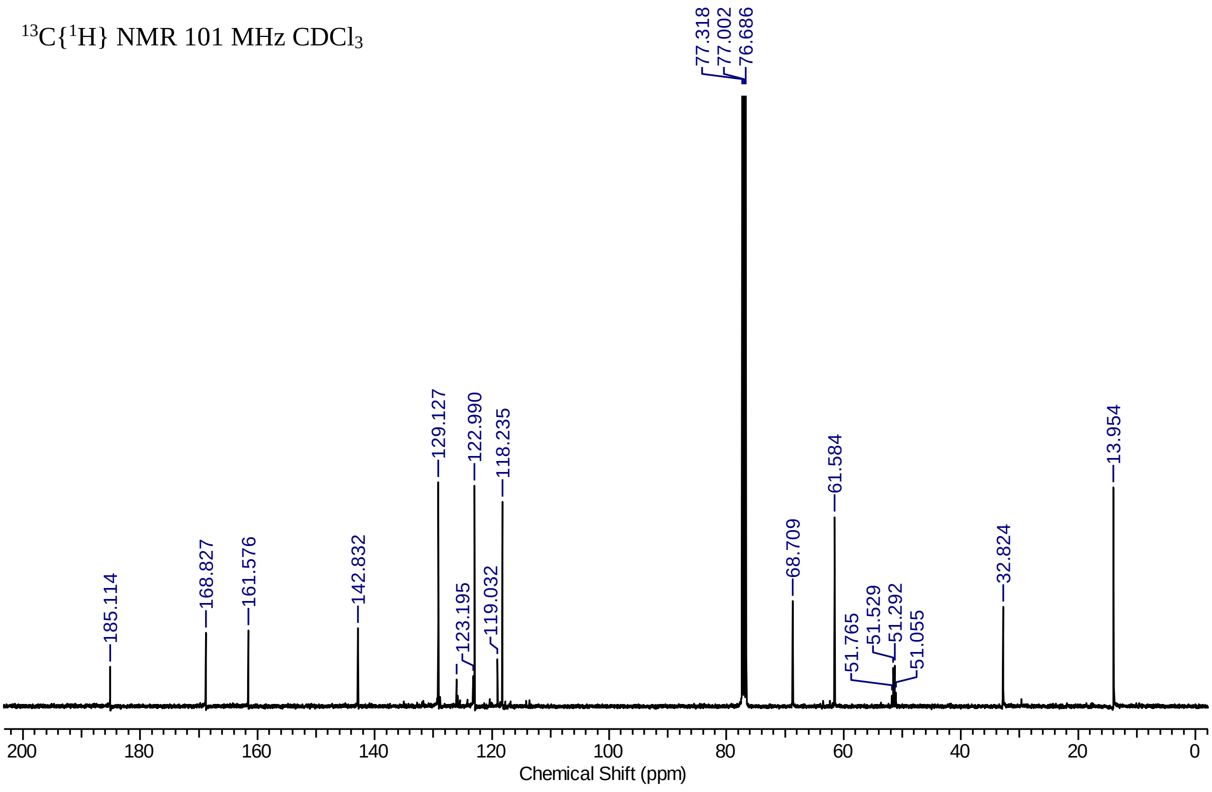

Figure S53. ^1H and ^{13}C NMR spectra of compound **2l.**

^{1}H NMR 700 MHz $CDCl_3$

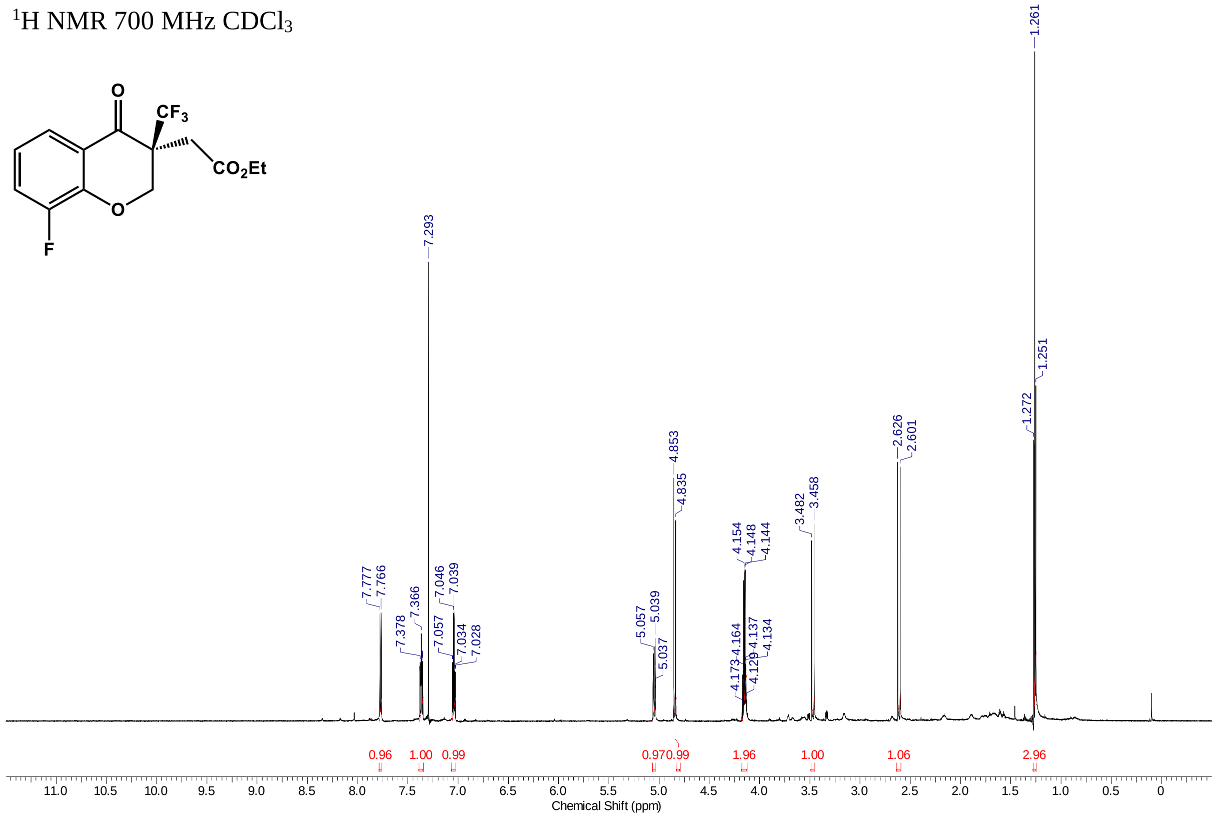

$^{13}C\{^{1}H\}$ NMR 101 MHz $CDCl_3$

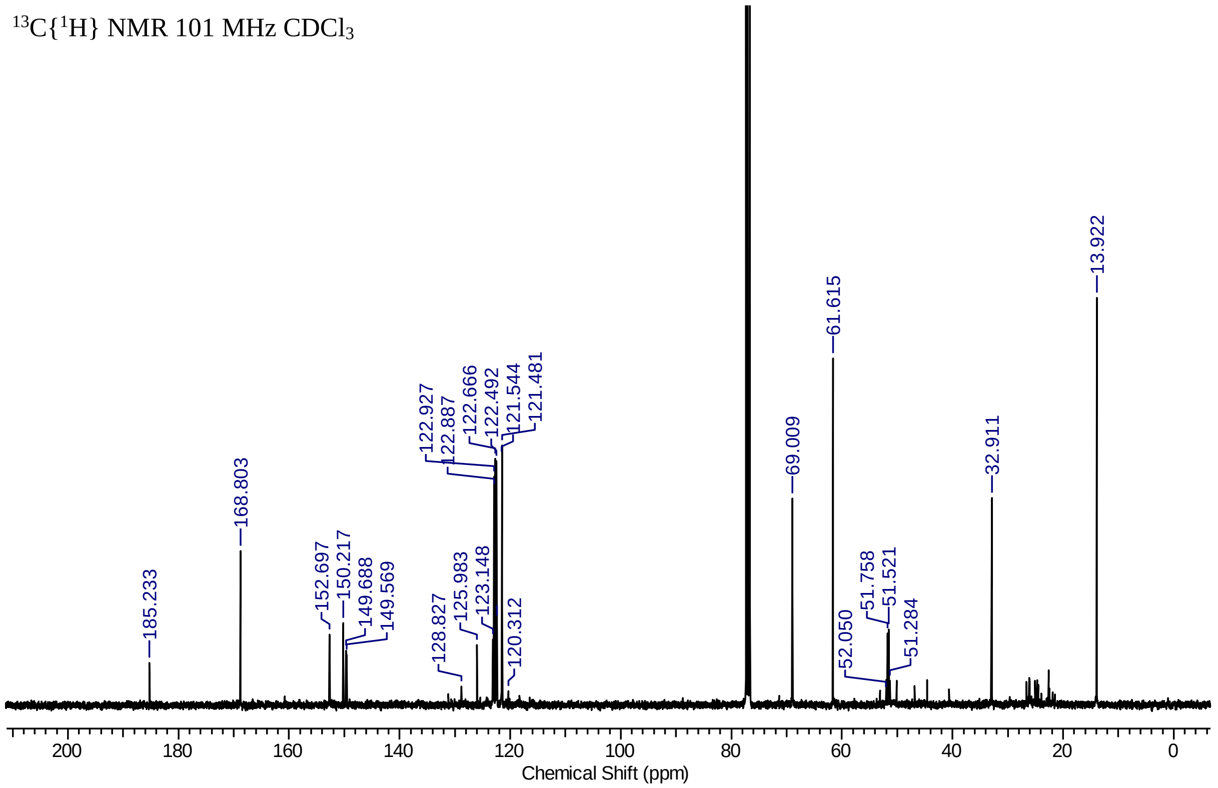

Figure S54. ^{1}H and ^{13}C NMR spectra of compound **2m.**

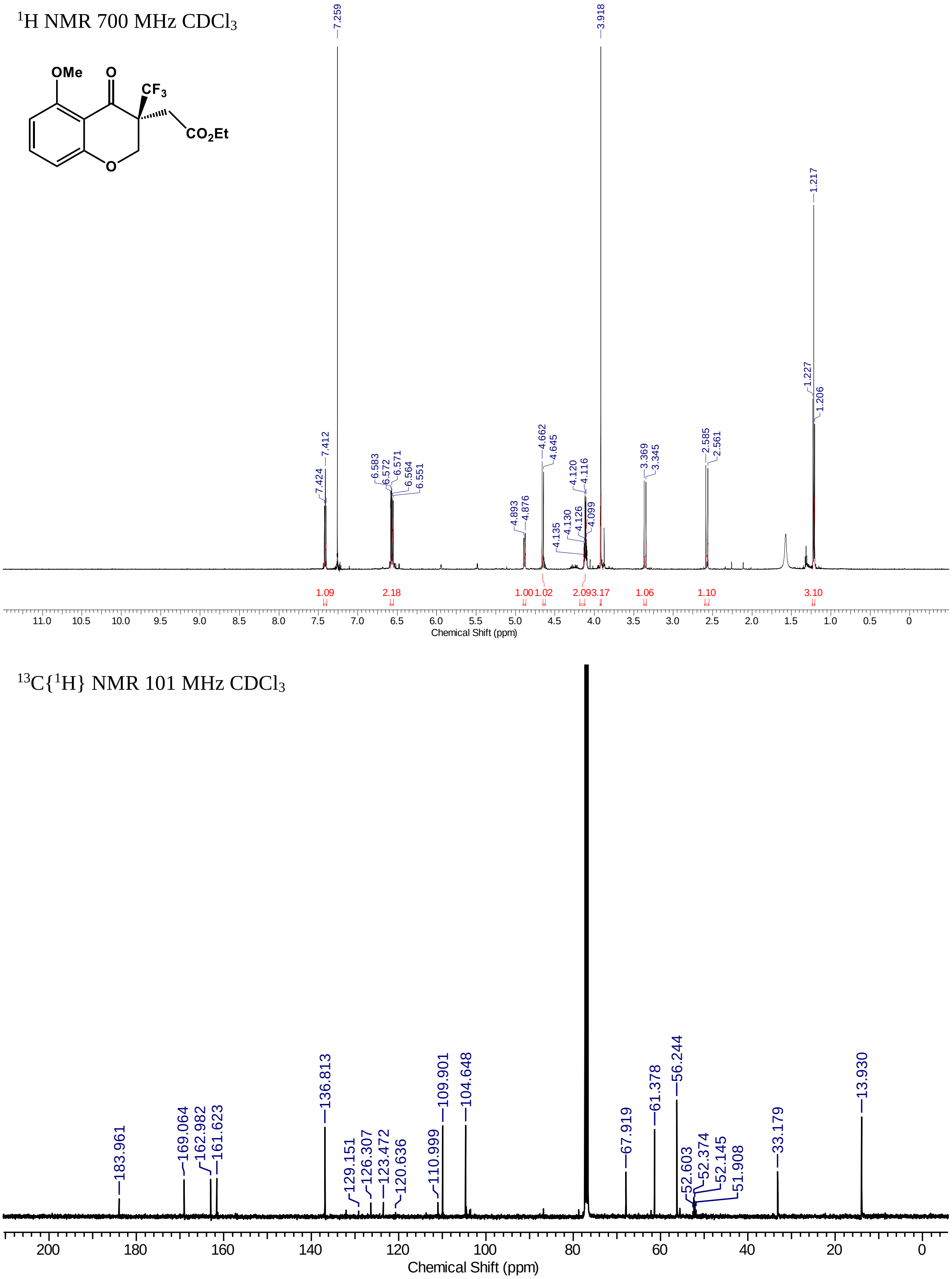

Figure S55. ^{1}H and ^{13}C NMR spectra of compound **2n.**

^{1}H NMR 400 MHz $CDCl_3$

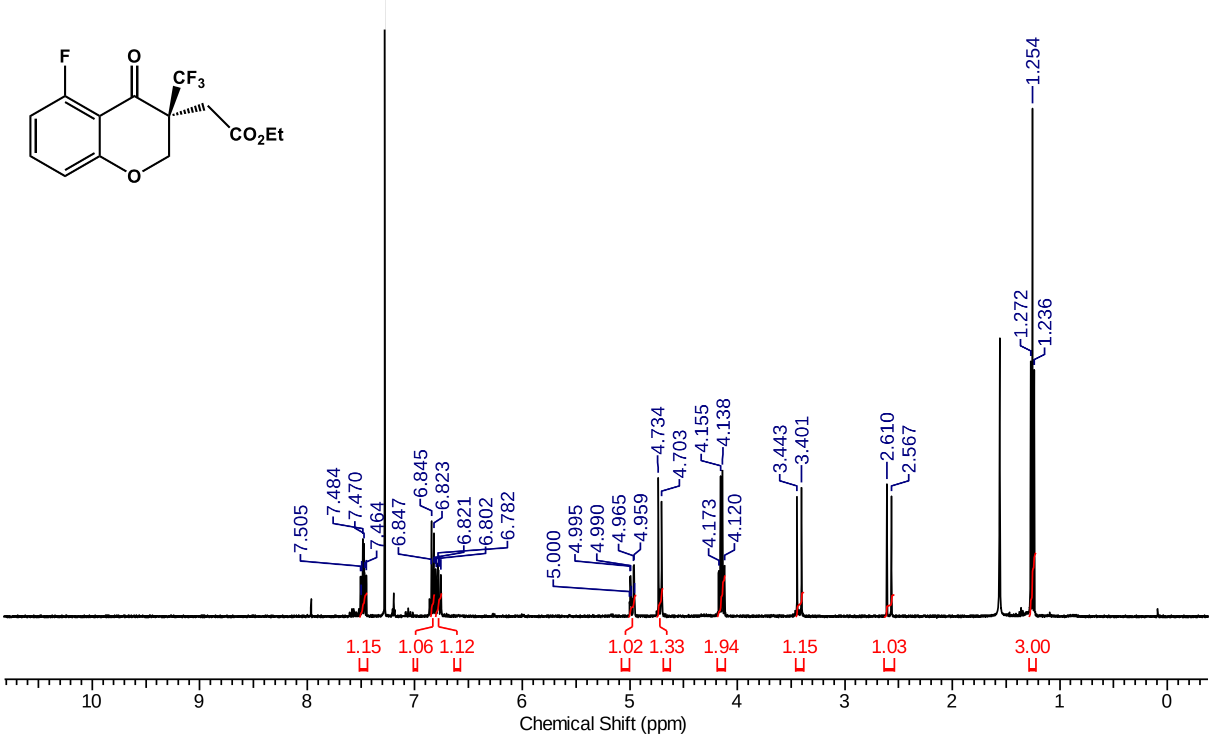

$^{13}C\{^{1}H\}$ NMR 101 MHz $CDCl_3$

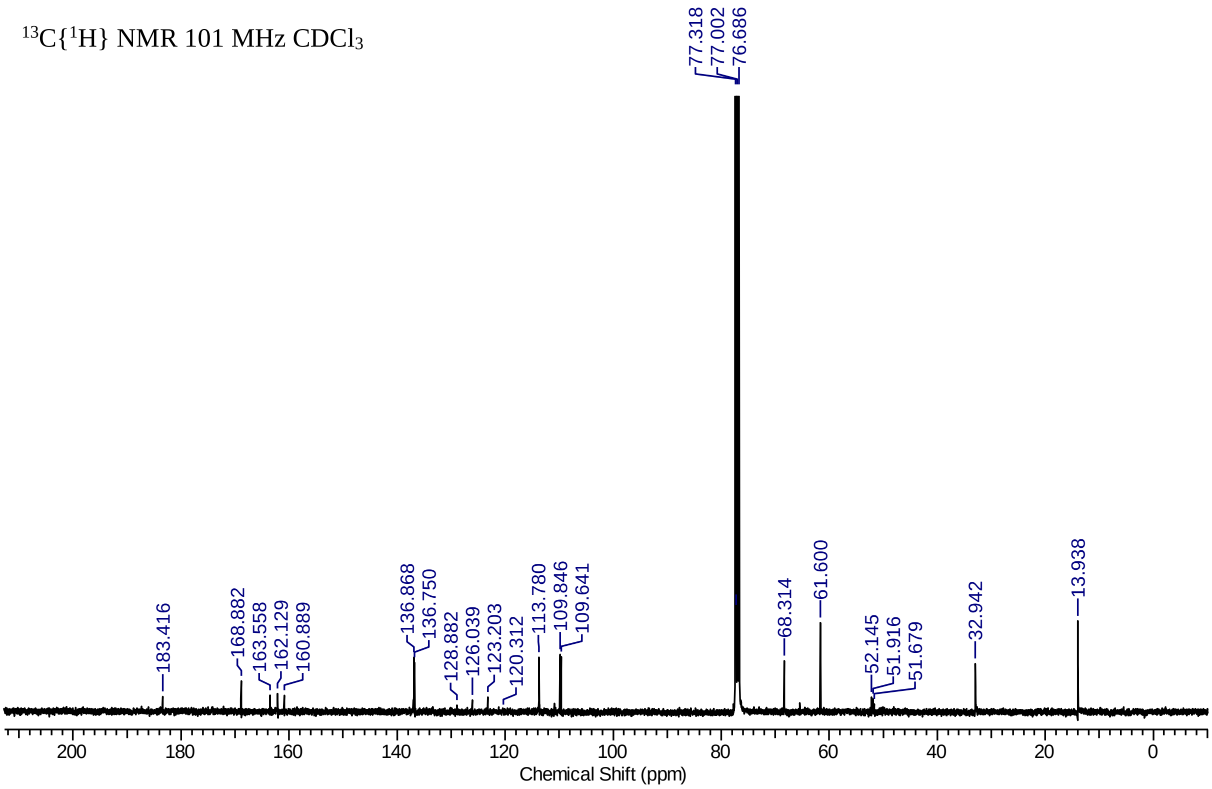

Figure S56. ^{1}H and ^{13}C NMR spectra of compound **2o.**

^{1}H NMR 700 MHz $CDCl_3$

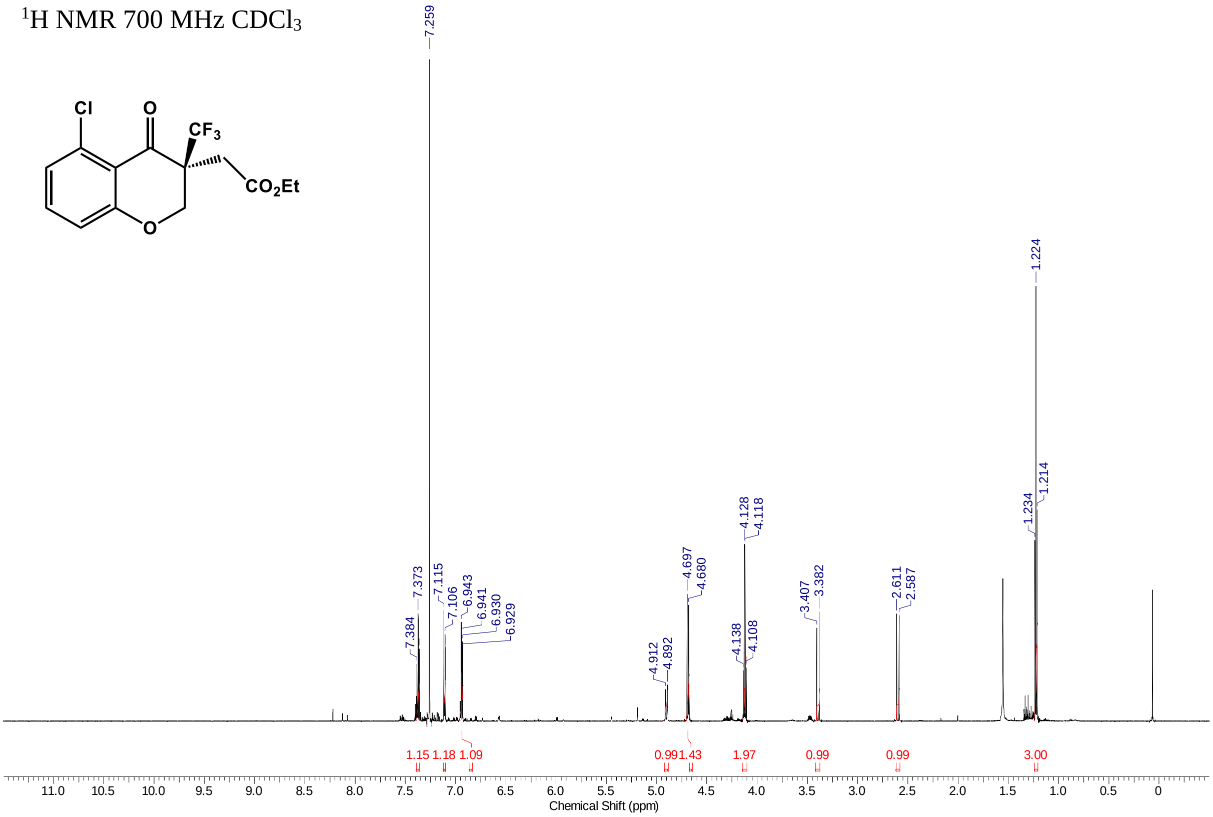

$^{13}C\{^{1}H\}$ NMR 101 MHz $CDCl_3$

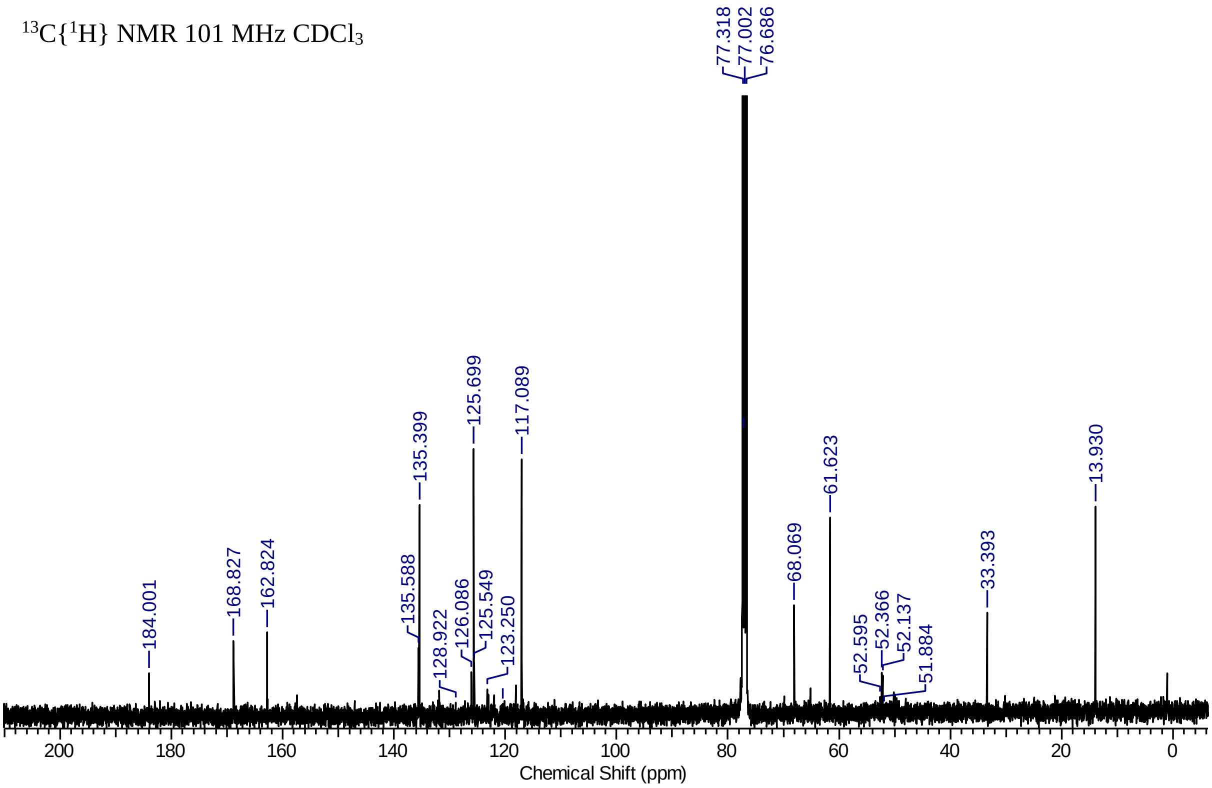

Figure S57. ^{1}H and ^{13}C NMR spectra of compound **2p.**

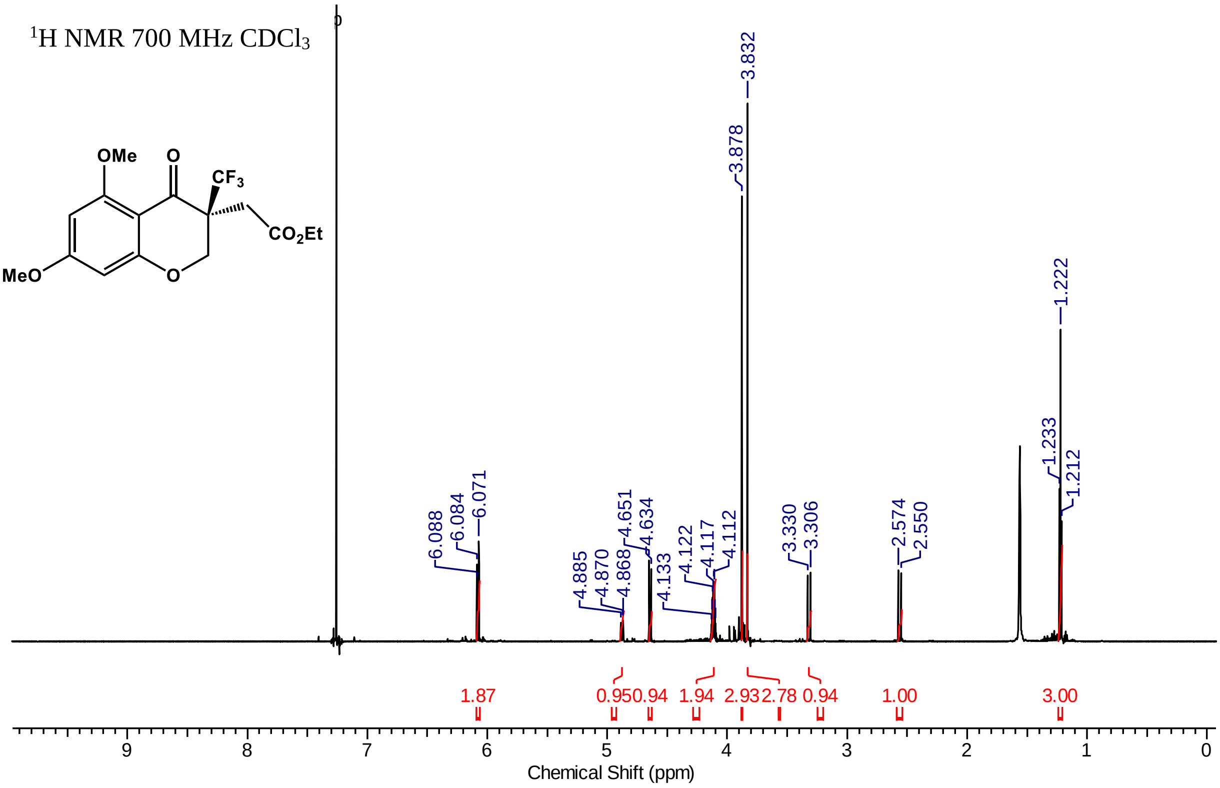

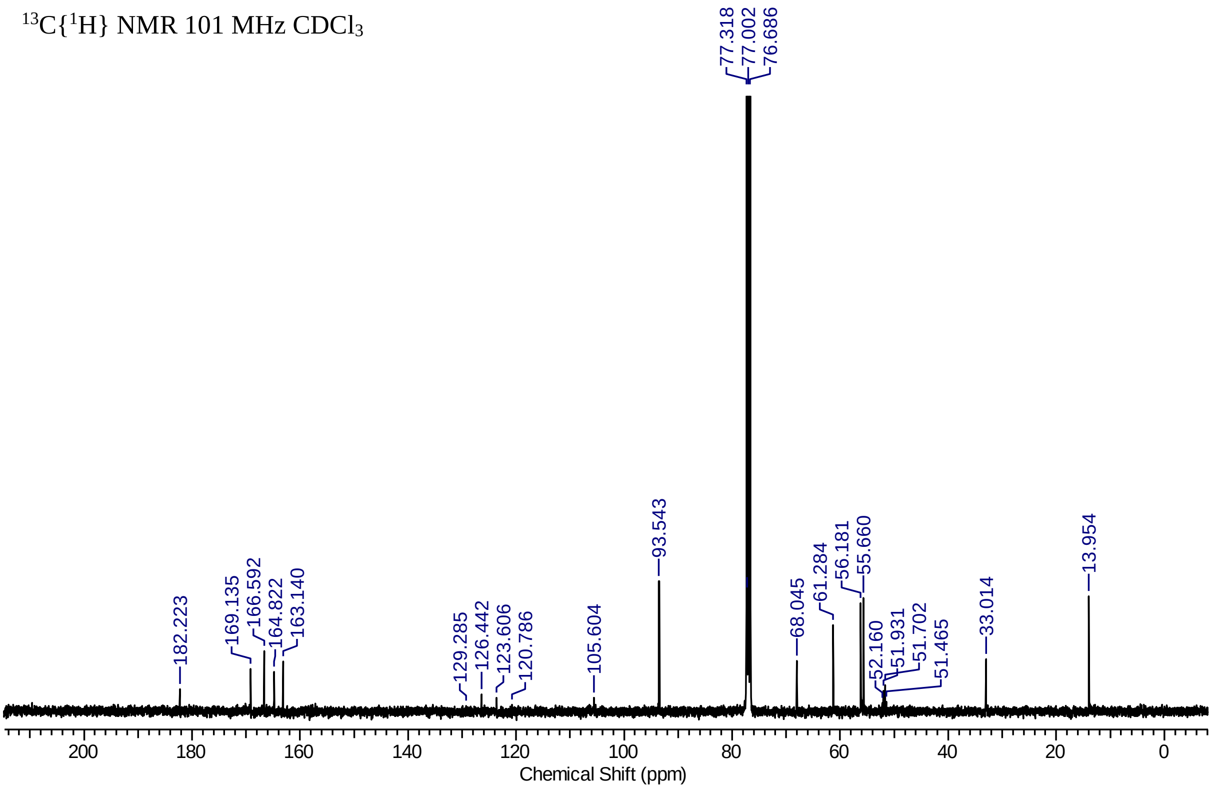

Figure S58. ^{1}H and ^{13}C NMR spectra of compound **2q.**

^1H NMR 700 MHz $CDCl_3$

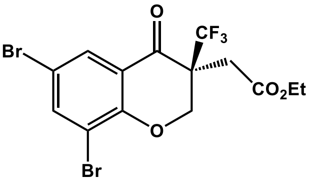

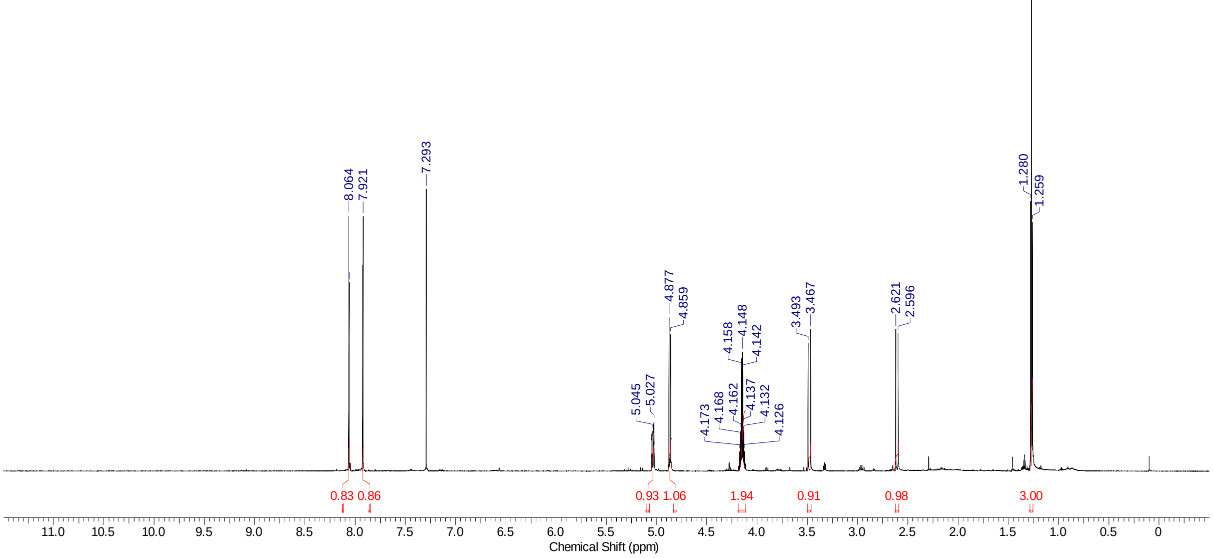

$^{13}C\{^1H\}$ NMR 176 MHz $CDCl_3$

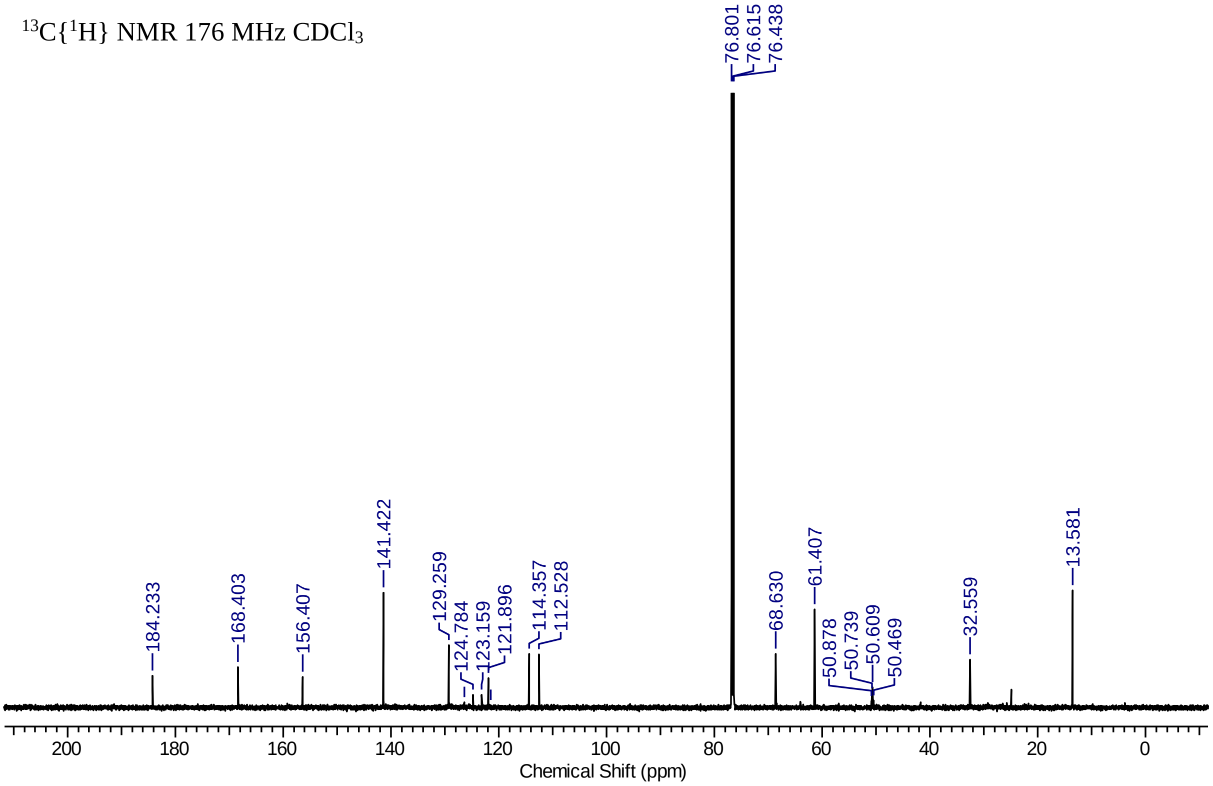

Figure S59. ^1H and ^{13}C NMR spectra of compound **2r.**

^{1}H NMR 700 MHz $CDCl_3$

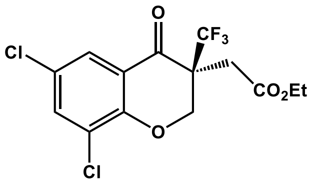

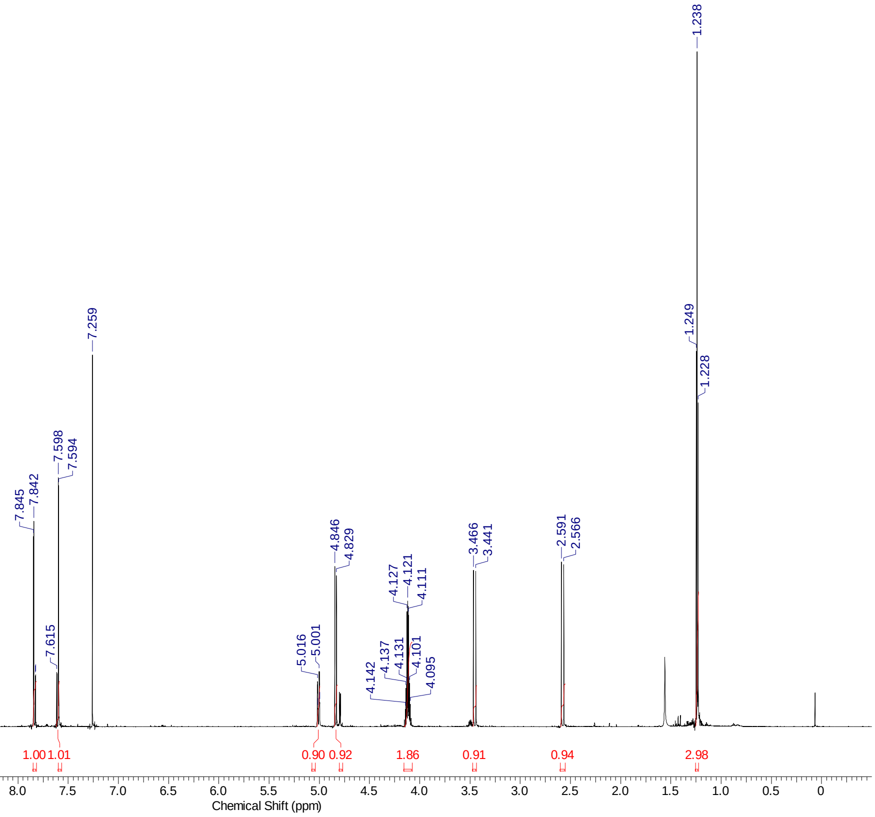

^{13}C{^{1}H} NMR 101 MHz $CDCl_3$

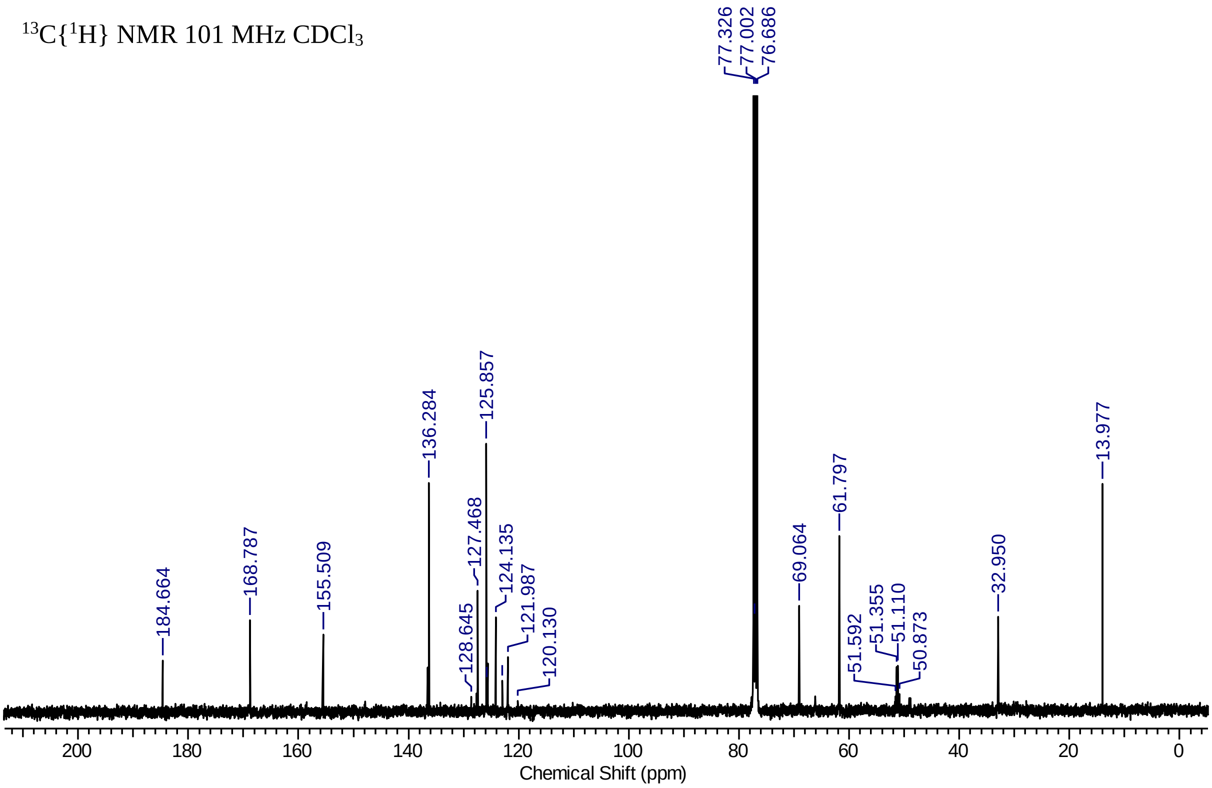

Figure S60. ^{1}H and ^{13}C NMR spectra of compound **2s.**

^{1}H NMR 700 MHz $CDCl_3$

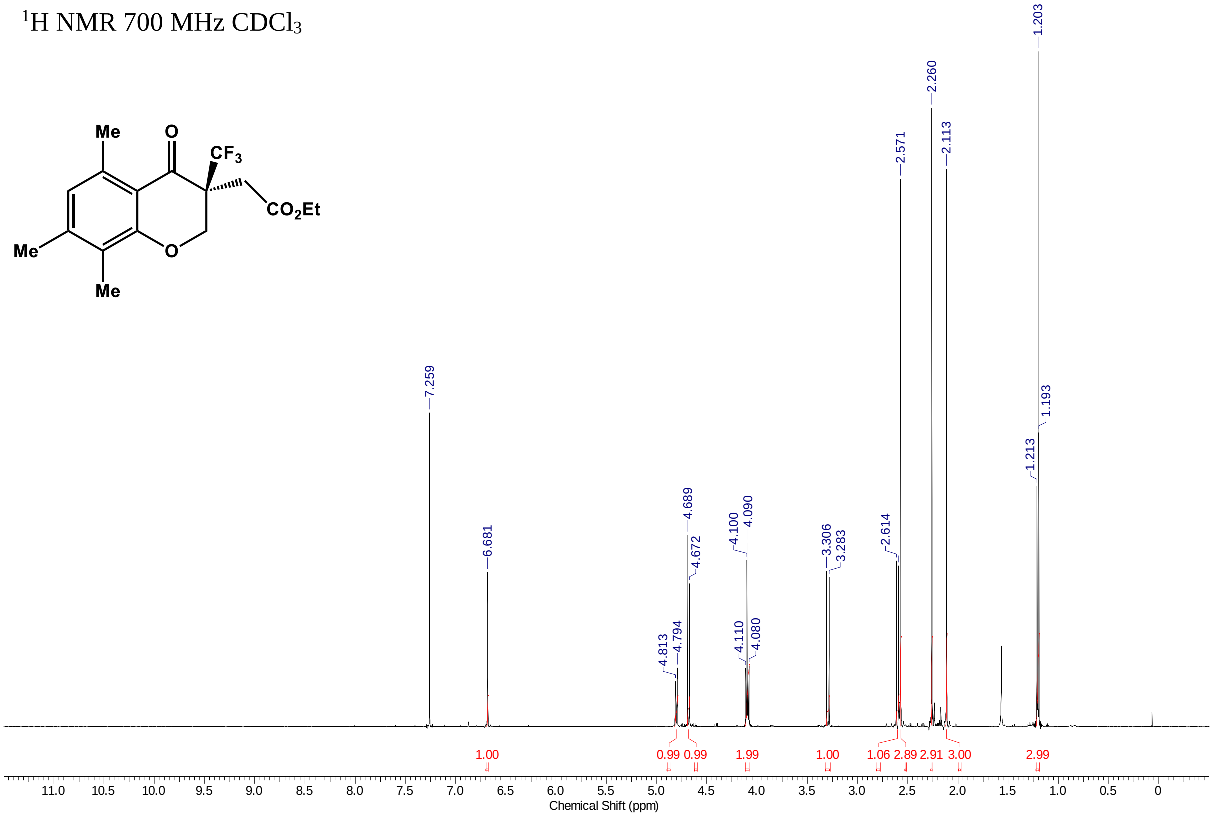

^{13}C{^{1}H} NMR 101 MHz $CDCl_3$

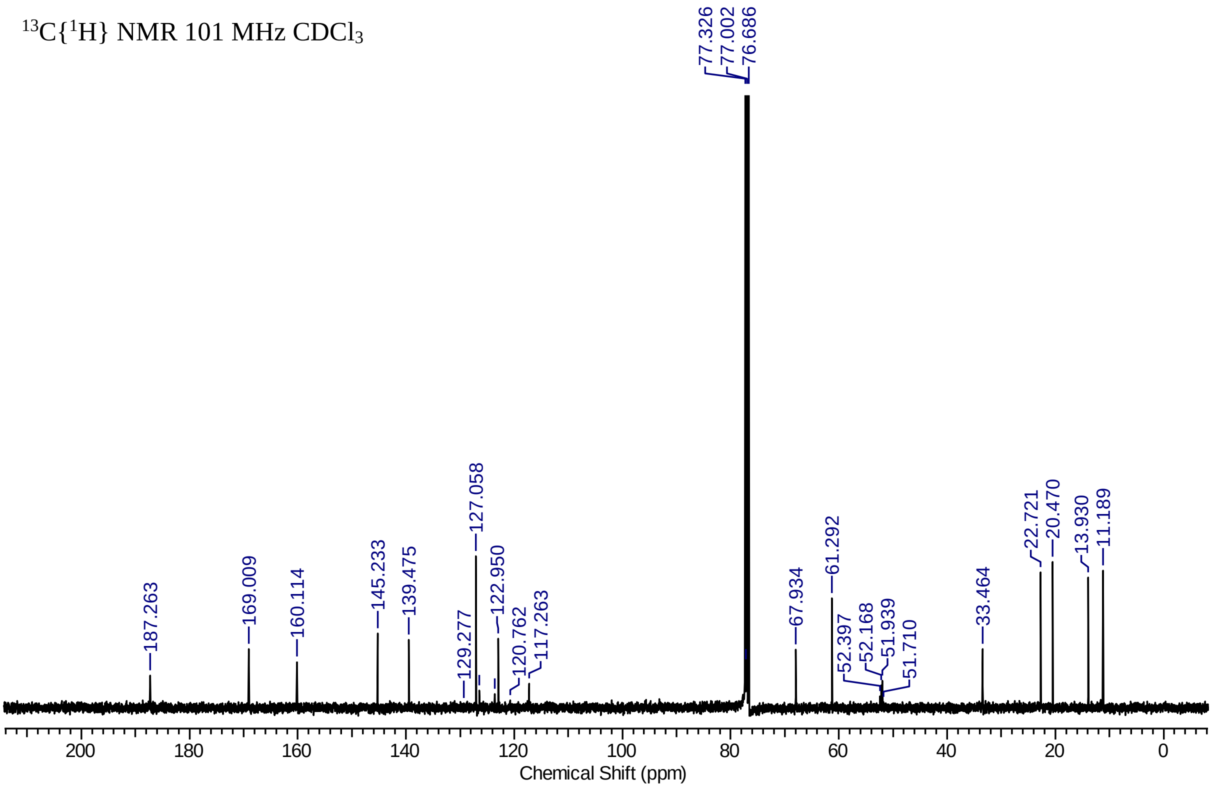

Figure S61. ^{1}H and ^{13}C NMR spectra of compound **2t.**

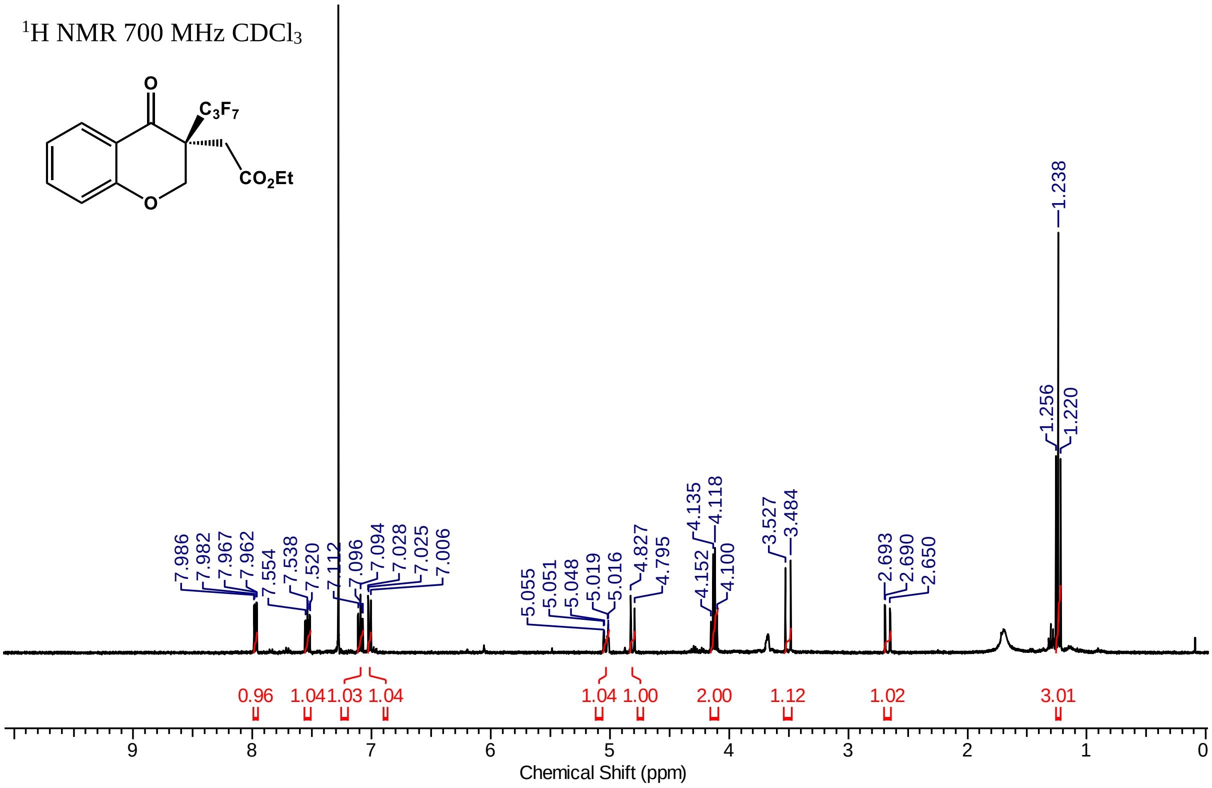

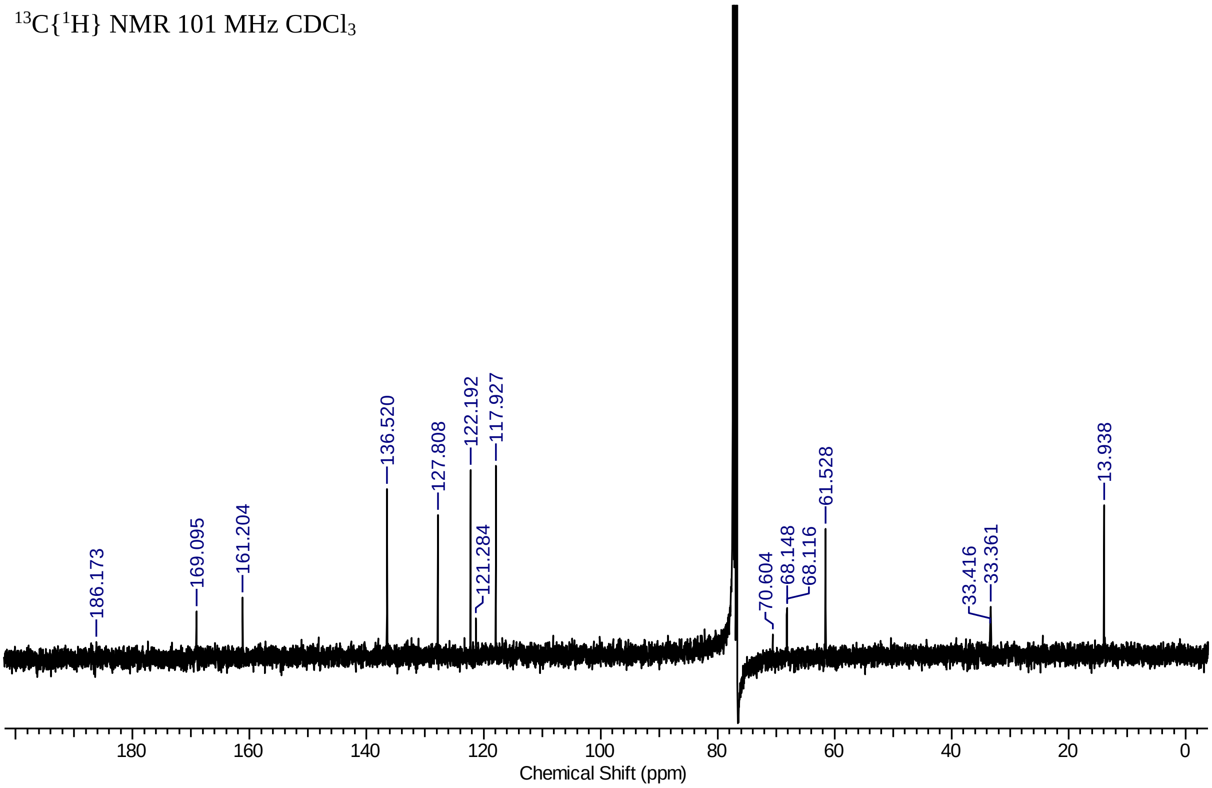

Figure S62. ^{1}H and ^{13}C NMR spectra of compound **2u.**

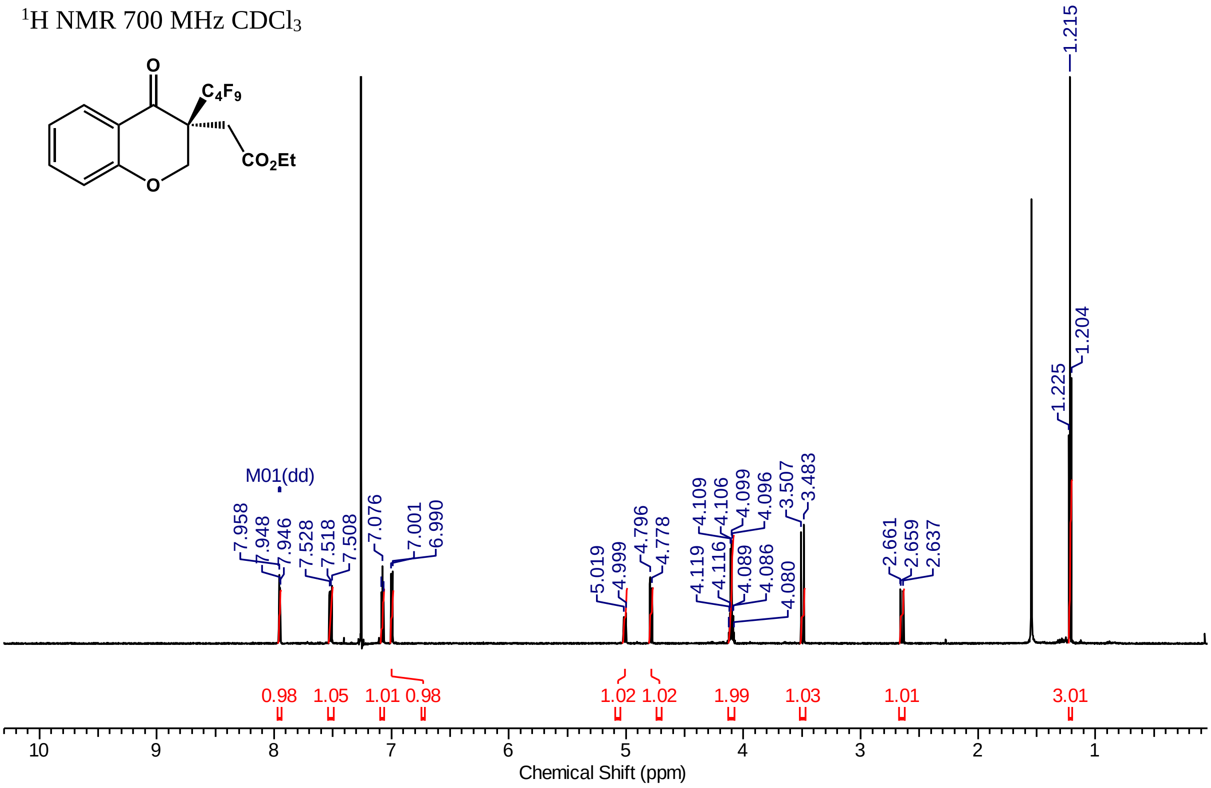

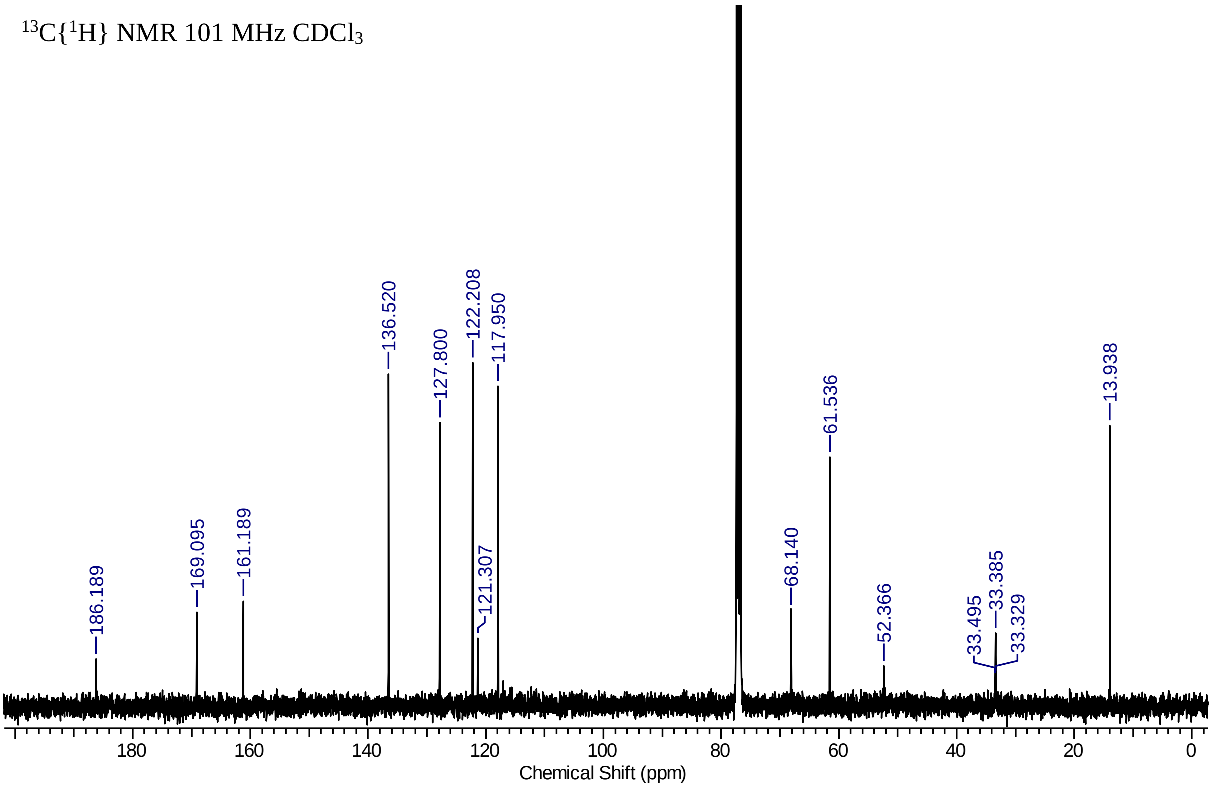

Figure S63. ^{1}H and ^{13}C NMR spectra of compound **2v.**

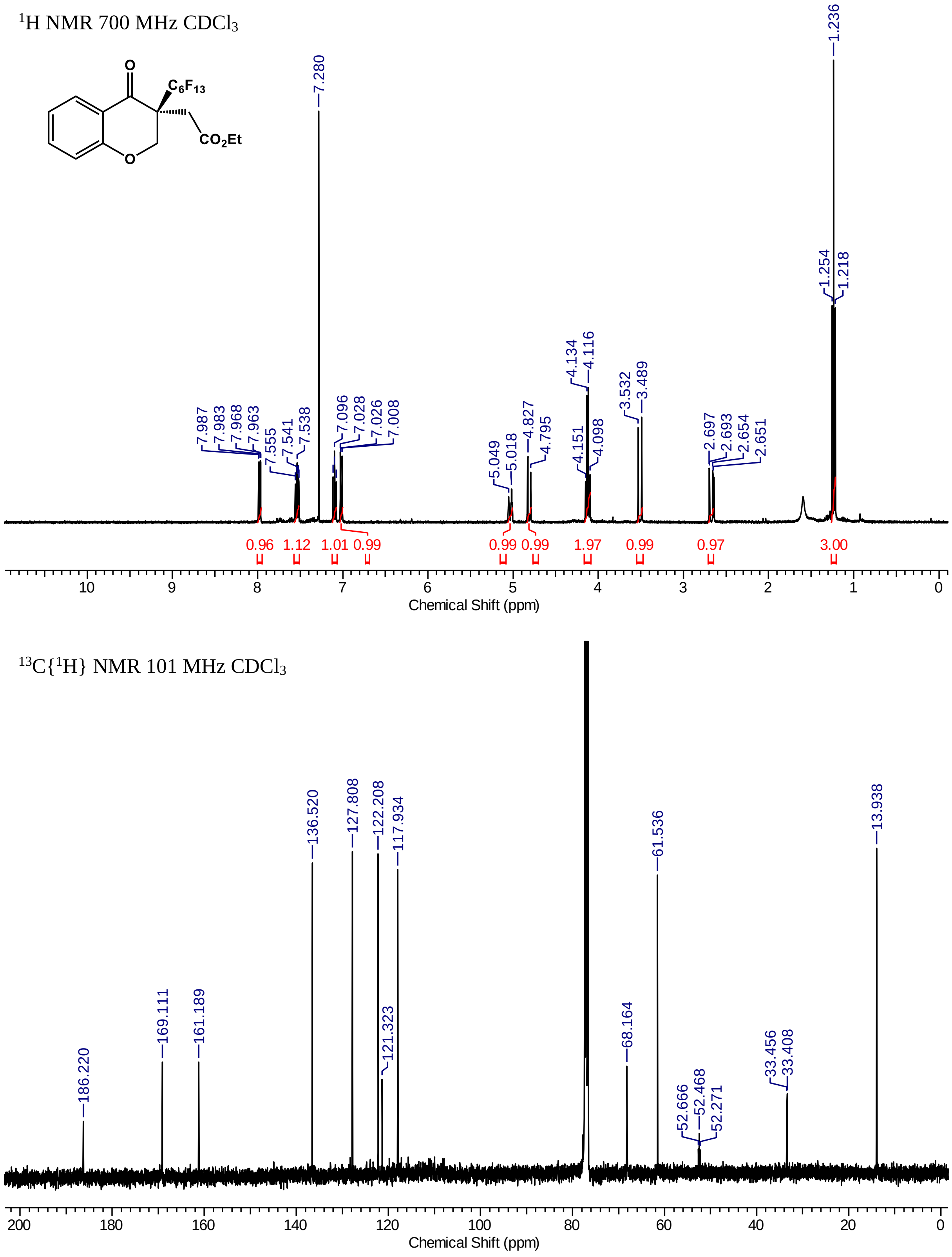

Figure S64. ^{1}H and ^{13}C NMR spectra of compound **2w.**

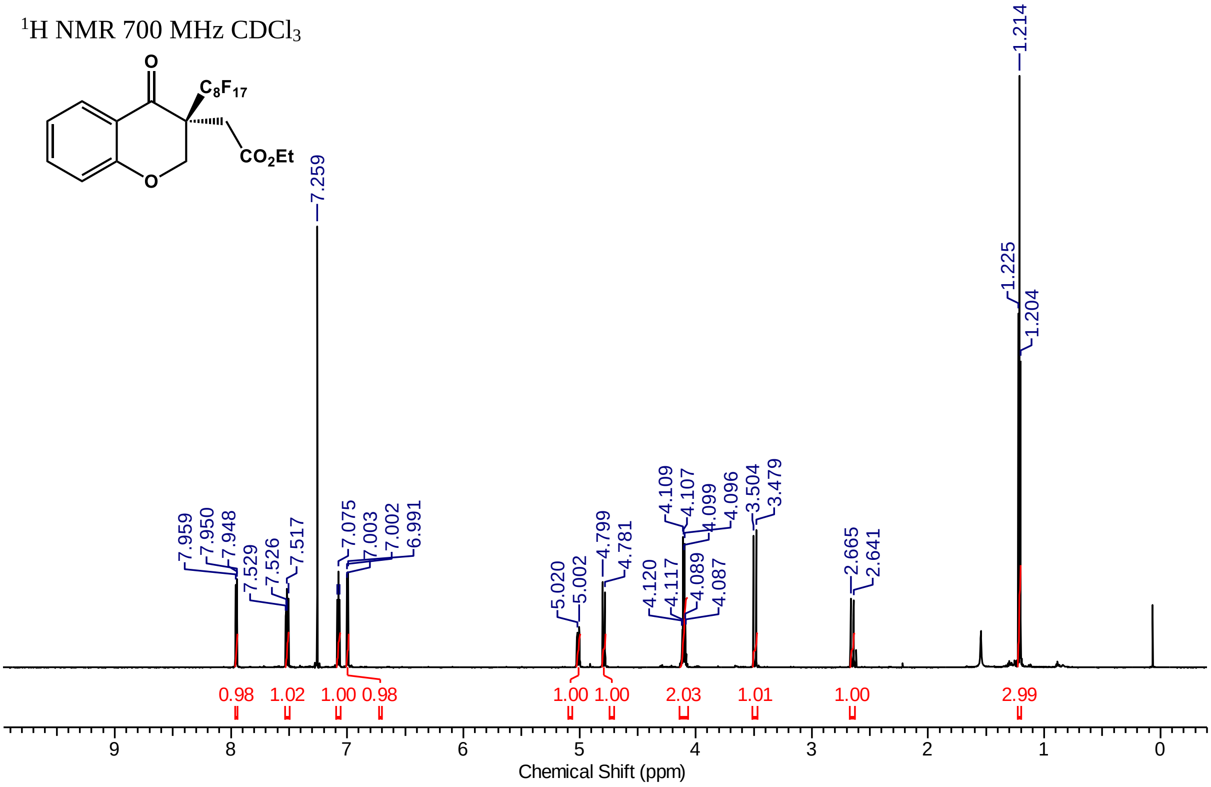

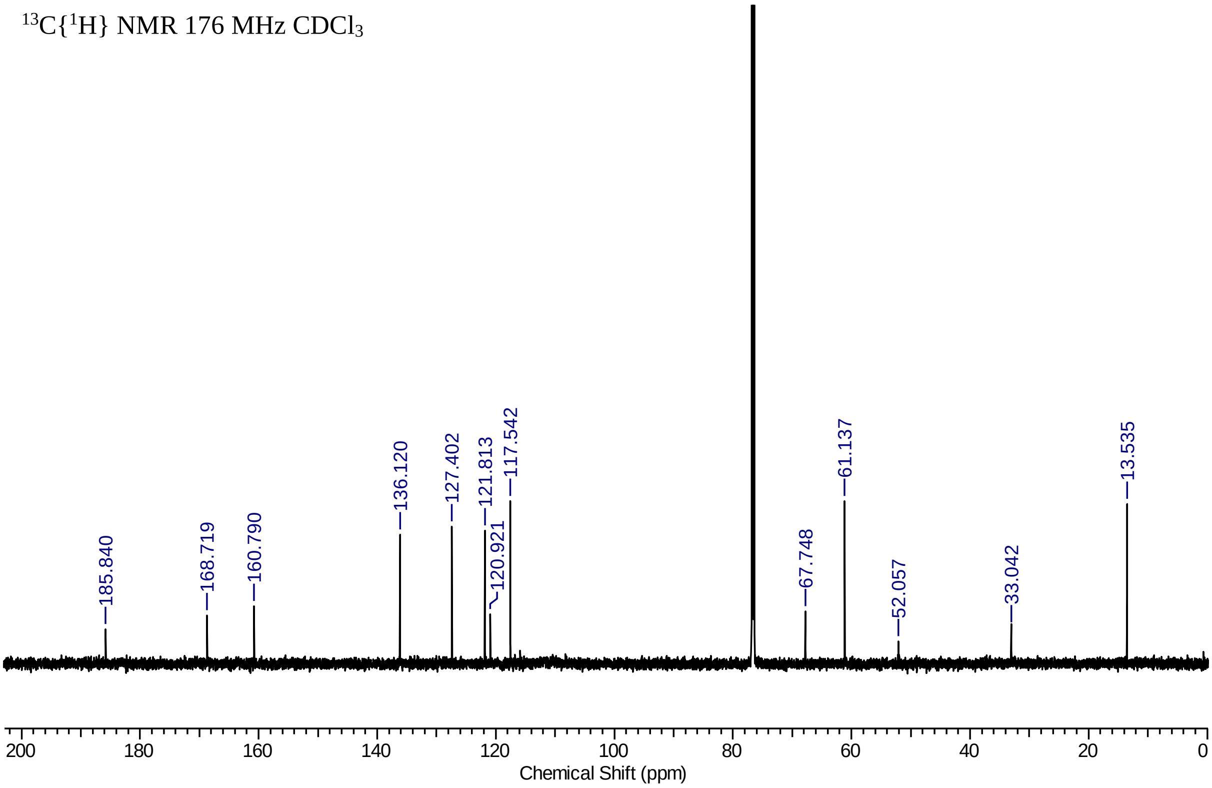

Figure S65. ¹H and ¹³C NMR spectra of compound **2x.**

¹H NMR 400 MHz $CDCl_3$

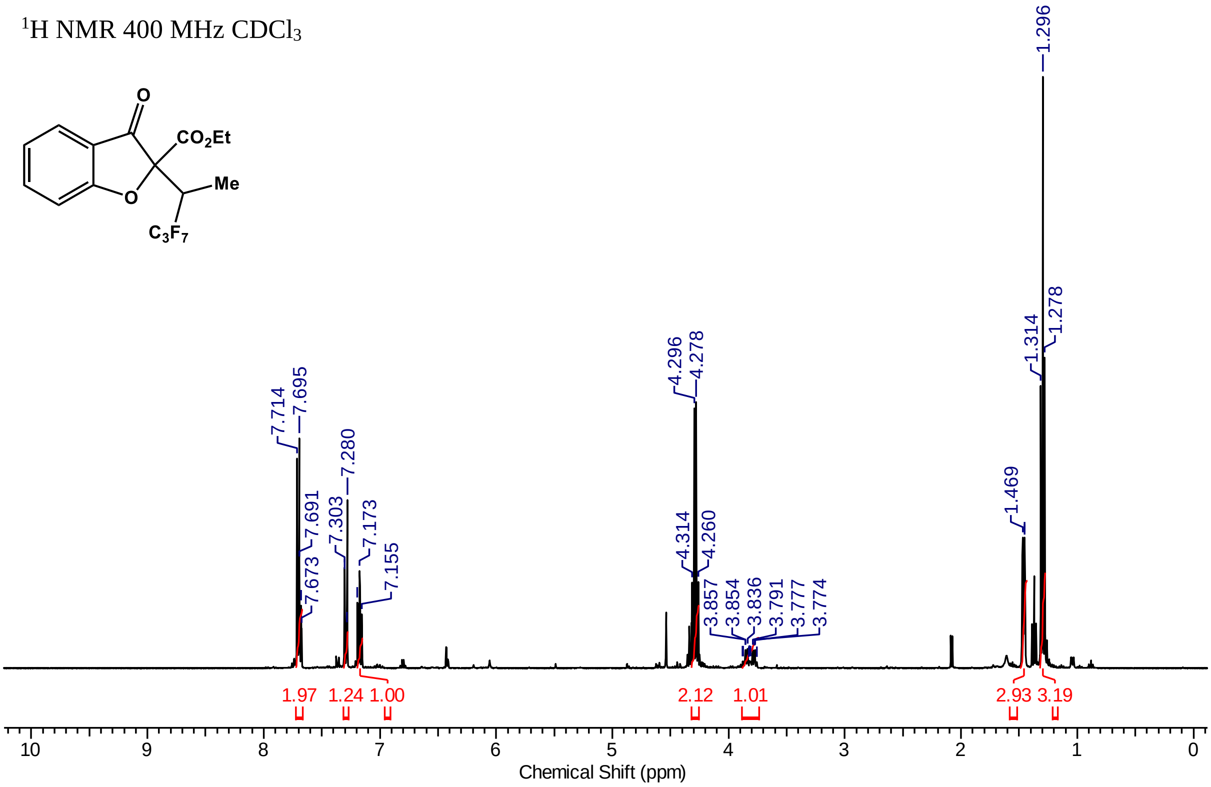

$^{13}C\{^1H\}$ NMR 101 MHz $CDCl_3$

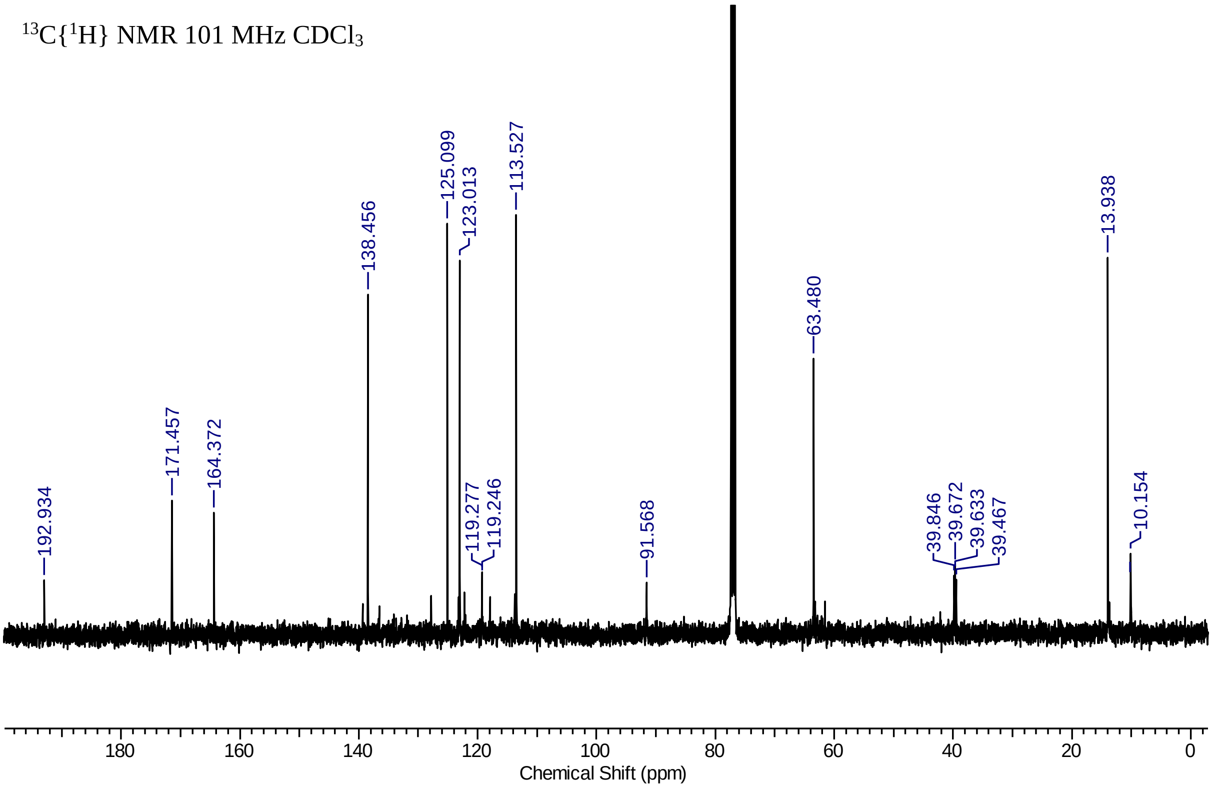

Figure S66. ¹H and ¹³C NMR spectra of compound **4a.**

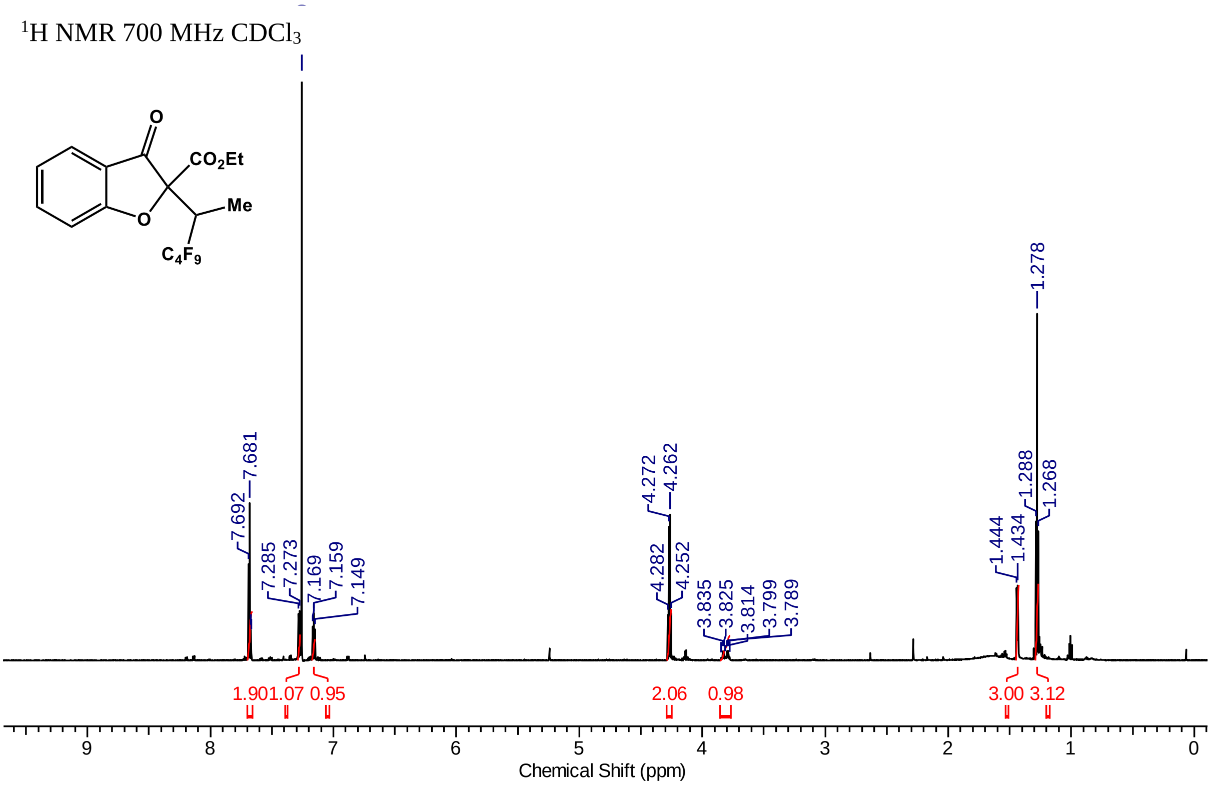

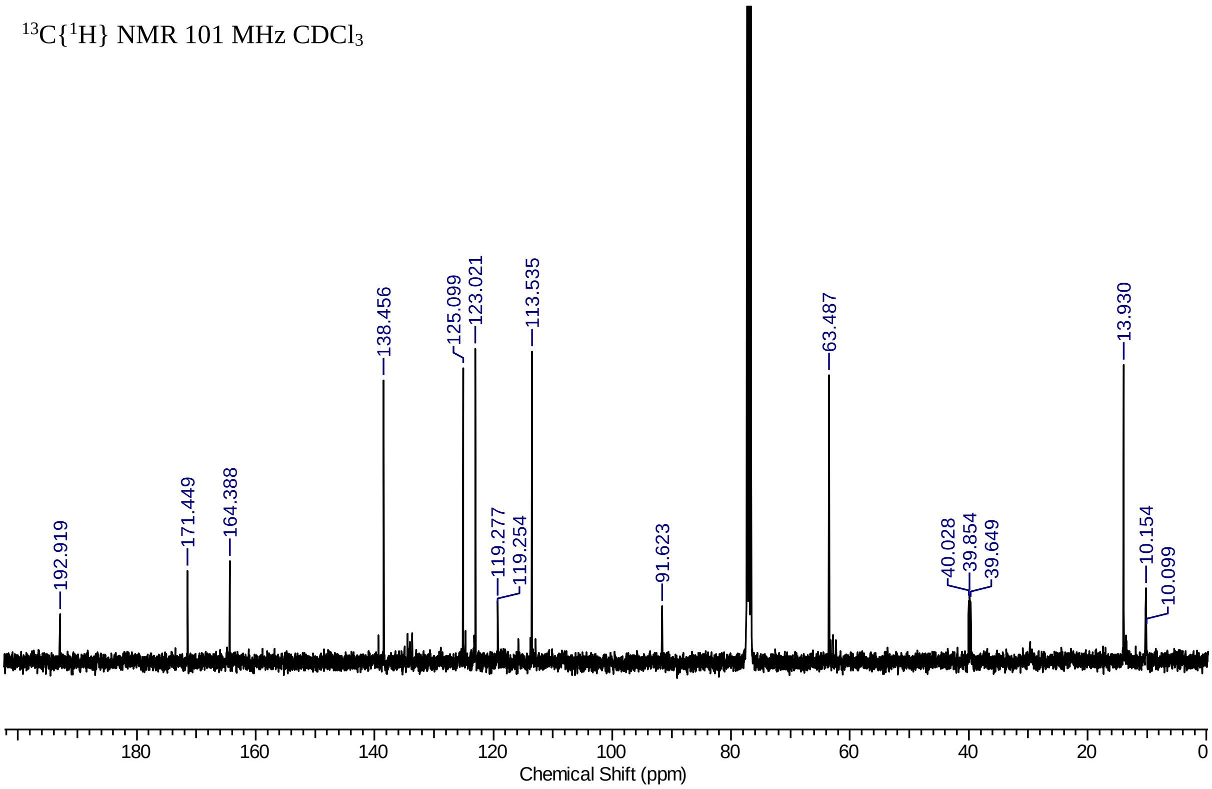

Figure S67. ^{1}H and ^{13}C NMR spectra of compound **4b.**

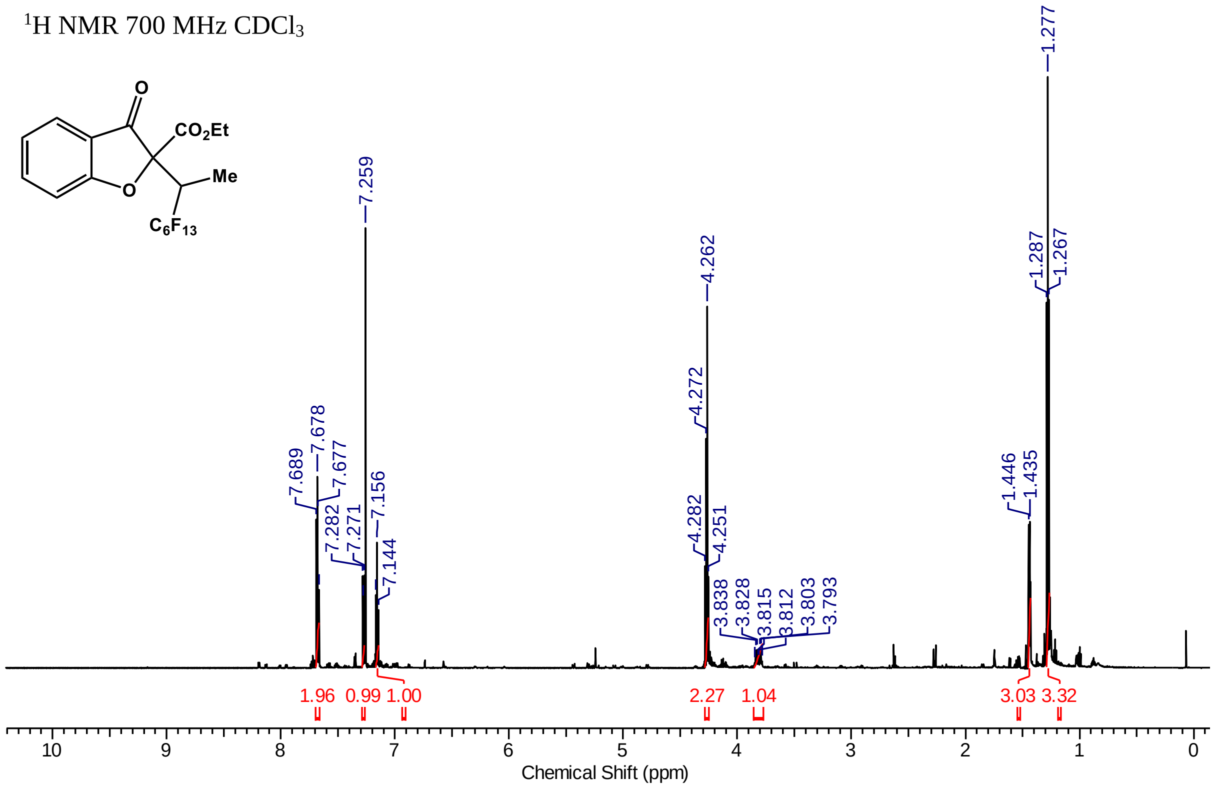

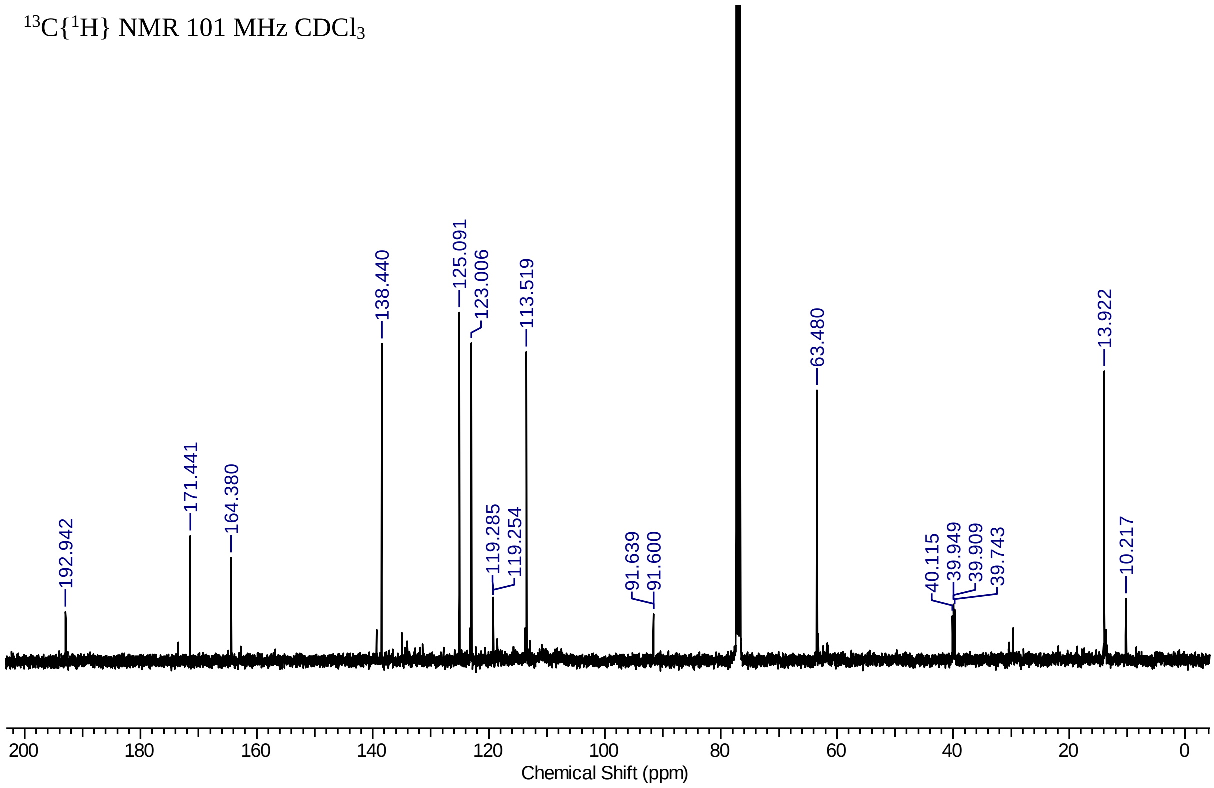

Figure S68. ^{1}H and ^{13}C NMR spectra of compound **4c.**

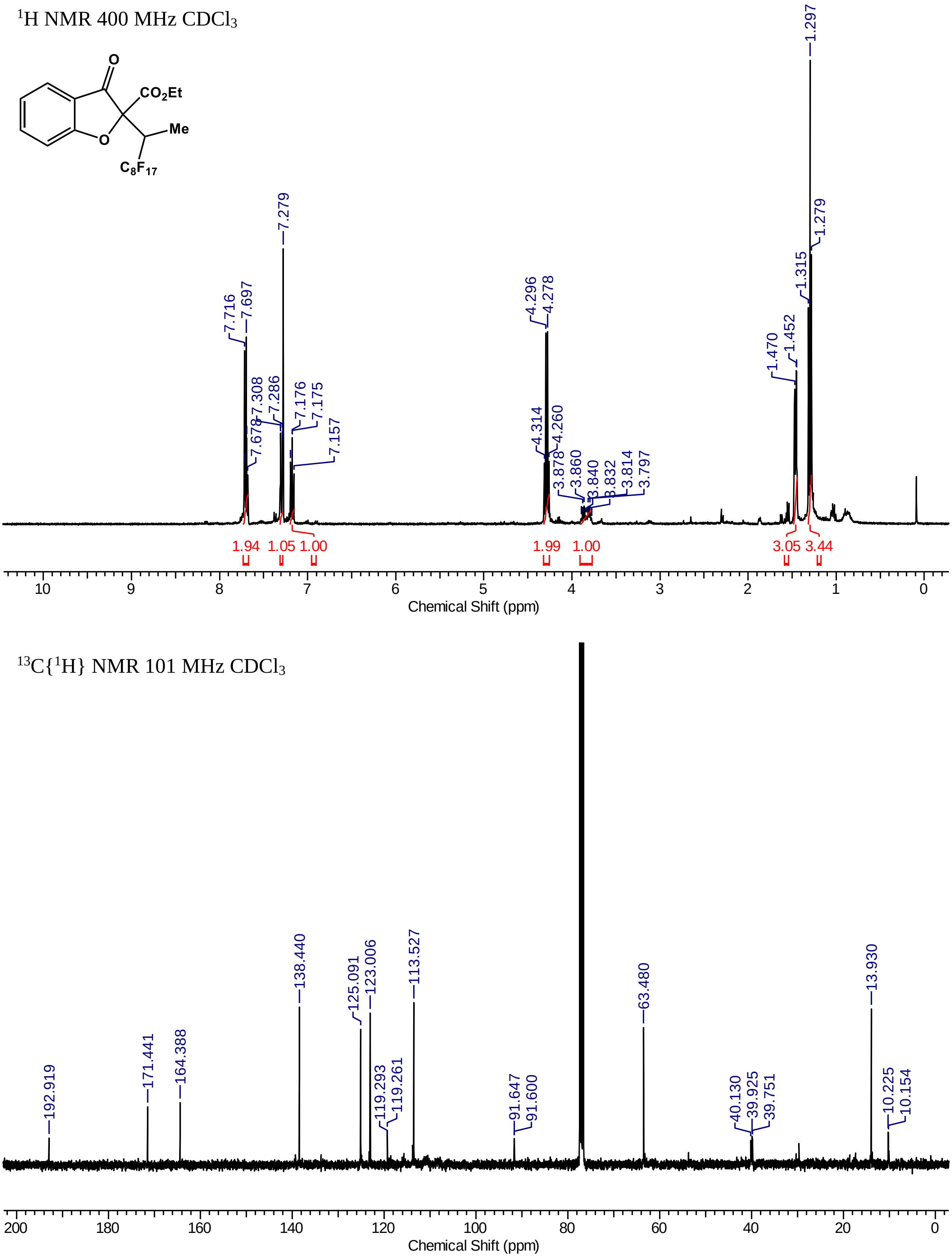

Figure S69. ^{1}H and ^{13}C NMR spectra of compound **4d.**

^{1}H NMR 400 MHz $CDCl_3$

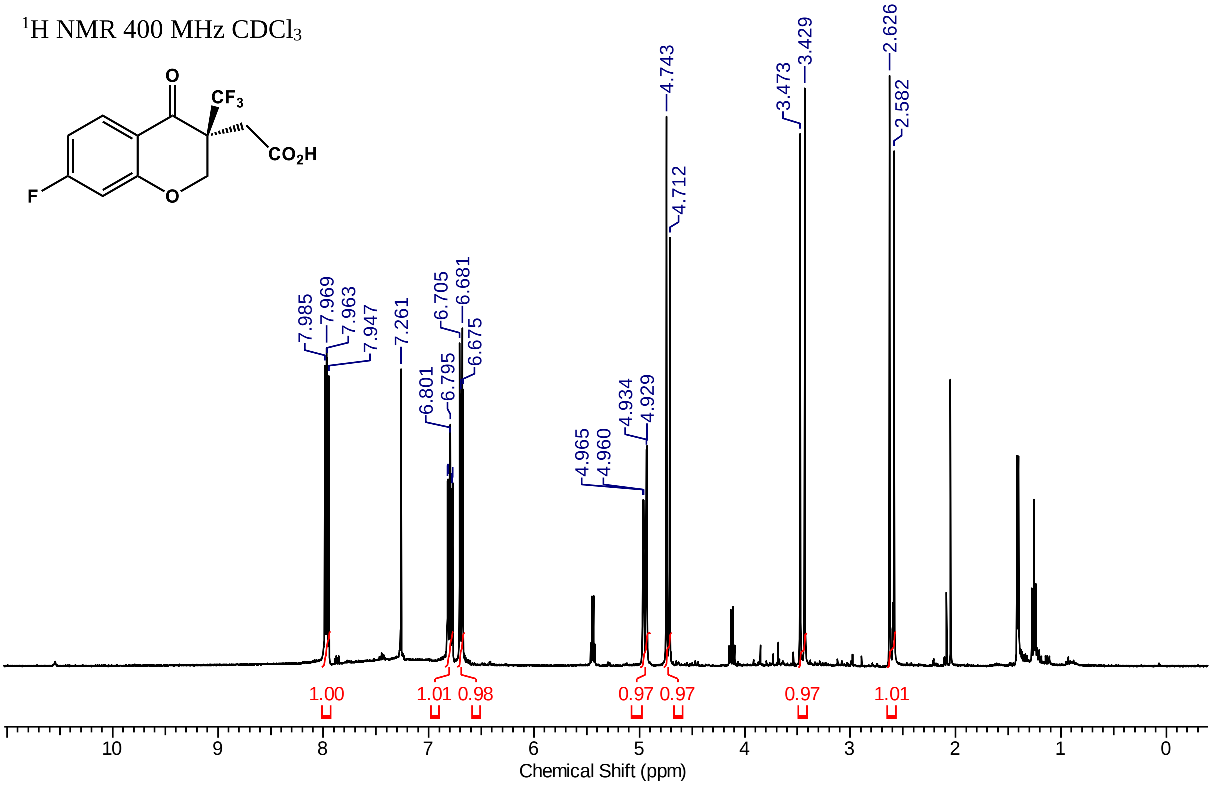

^{13}C{^{1}H} NMR 101 MHz $CDCl_3$

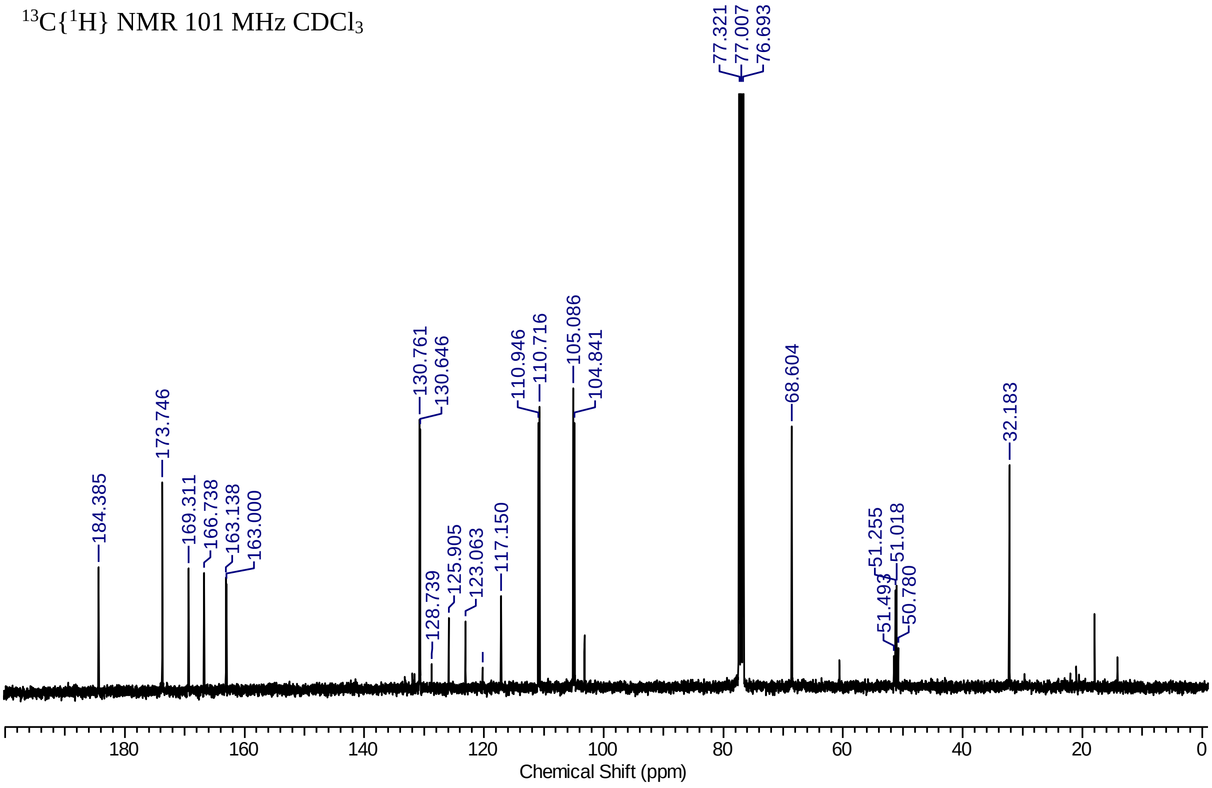

Figure S70. ^{1}H and ^{13}C NMR spectra of compound **7.**

5. HPLC Chromatograms

Additional Info : Peak(s) manually integrated

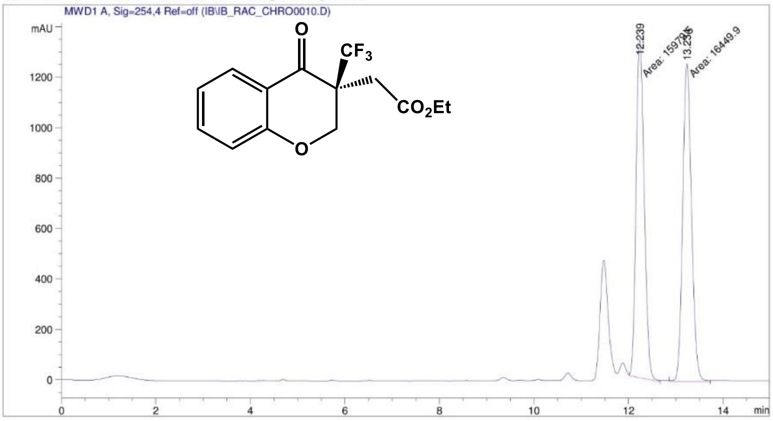


```
==================================================================
                        Area Percent Report
==================================================================

Sorted By             :      Signal
Multiplier:                   :      1.0000
Dilution:                     :      1.0000
Use Multiplier & Dilution Factor with ISTDs


Signal 1: MWD1 A, Sig=254,4 Ref=off

Peak RetTime Type  Width     Area      Height     Area
  #   [min]        [min]   [mAU*s]     [mAU]        %
----|-------|----|-------|----------|----------|--------|
   1  12.239 MM    0.1988 1.59795e4  1339.95837  49.2747
   2  13.234 MM    0.2173 1.64499e4  1261.53186  50.7253
```

Additional Info : Peak(s) manually integrated

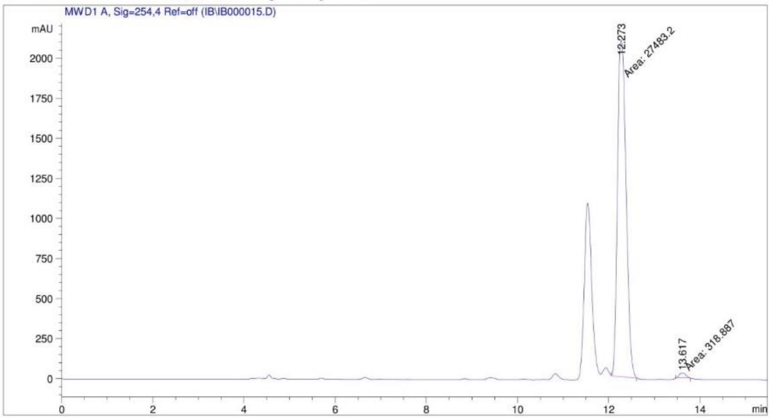


```
==================================================================
                        Area Percent Report
==================================================================

Sorted By             :      Signal
Multiplier:                   :      1.0000
Dilution:                     :      1.0000
Use Multiplier & Dilution Factor with ISTDs


Signal 1: MWD1 A, Sig=254,4 Ref=off

Peak RetTime Type  Width     Area      Height     Area
  #   [min]        [min]   [mAU*s]     [mAU]        %
----|-------|----|-------|----------|----------|--------|
   1  12.273 MM    0.2183 2.74832e4  2098.51782  98.8530
   2  13.617 MM    0.1661  318.88715   32.00179   1.1470
```

Figure S71. HPLC chromatograms of **2a** (racemic – top, chiral – bottom).

Additional Info : Peak(s) manually integrated

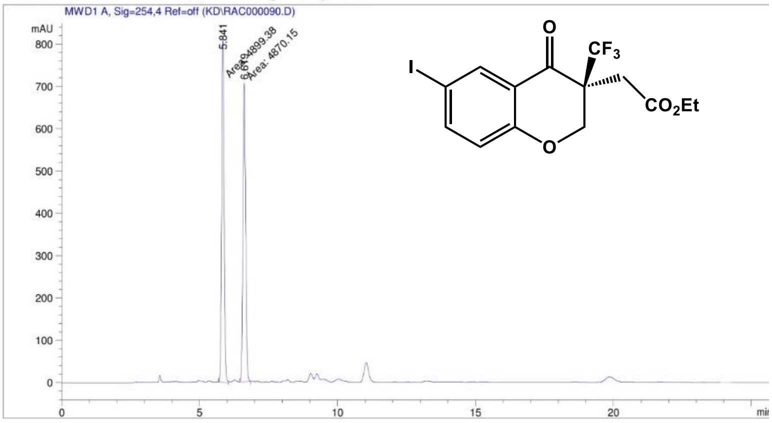

===

Area Percent Report

===

Sorted By : Signal
Multiplier: : 1.0000
Dilution: : 1.0000
Use Multiplier & Dilution Factor with ISTDs

Signal 1: MWD1 A, Sig=254,4 Ref=off

Peak #	RetTime [min]	Type	Width [min]	Area [mAU*s]	Height [mAU]	Area %
1	5.841	MM	0.1009	4899.37500	809.26038	50.1496
2	6.619	MM	0.1148	4870.15283	707.16504	49.8504

Additional Info : Peak(s) manually integrated

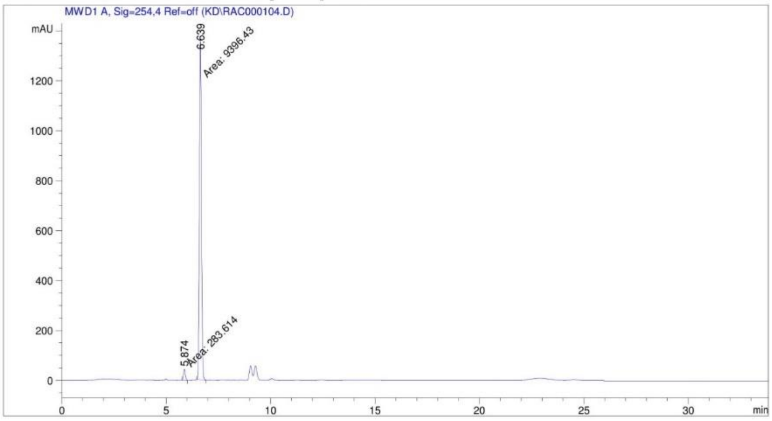

===

Area Percent Report

===

Sorted By : Signal
Multiplier: : 1.0000
Dilution: : 1.0000
Use Multiplier & Dilution Factor with ISTDs

Signal 1: MWD1 A, Sig=254,4 Ref=off

Peak #	RetTime [min]	Type	Width [min]	Area [mAU*s]	Height [mAU]	Area %
1	5.874	MM	0.1039	283.61383	45.50917	2.9299
2	6.639	MM	0.1151	9396.43262	1360.44067	97.0701

Figure S72. HPLC chromatograms of **2b** (racemic – top, chiral – bottom).

[illegible]

Additional Info : Peak(s) manually integrated

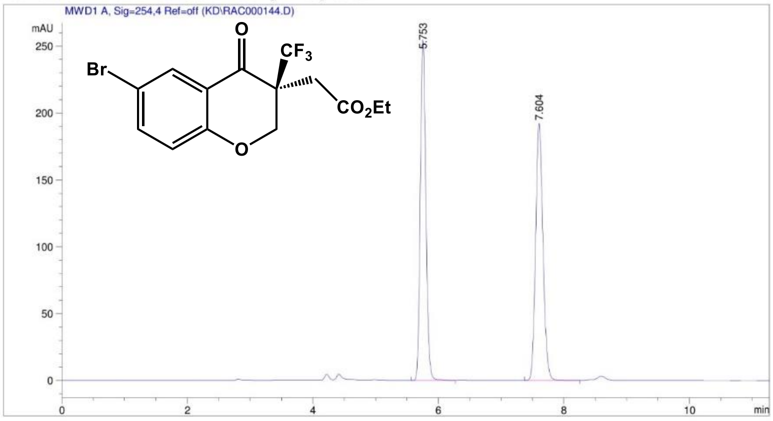

Area Percent Report

Sorted By : Signal
Multiplier: : 1.0000
Dilution: : 1.0000
Use Multiplier & Dilution Factor with ISTDs

Signal 1: MWD1 A, Sig=254,4 Ref=off

Peak #	RetTime [min]	Type	Width [min]	Area [mAU*s]	Height [mAU]	Area %
1	5.753	BV	0.0936	1561.74146	254.57571	49.9688
2	7.604	BB	0.1269	1563.68872	192.27576	50.0312

[illegible]

Additional Info : Peak(s) manually integrated

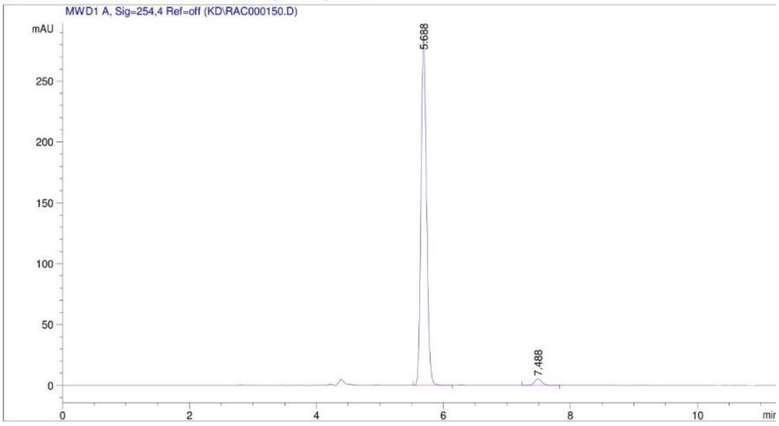

Area Percent Report

Sorted By : Signal
Multiplier: : 1.0000
Dilution: : 1.0000
Use Multiplier & Dilution Factor with ISTDs

Signal 1: MWD1 A, Sig=254,4 Ref=off

Peak #	RetTime [min]	Type	Width [min]	Area [mAU*s]	Height [mAU]	Area %
1	5.688	BB	0.0930	1725.69470	283.56064	97.5833
2	7.488	BB	0.1251	42.73806	5.24254	2.4167

Figure S73. HPLC chromatograms of **2c** (racemic – top, chiral – bottom).

Additional Info : Peak(s) manually integrated

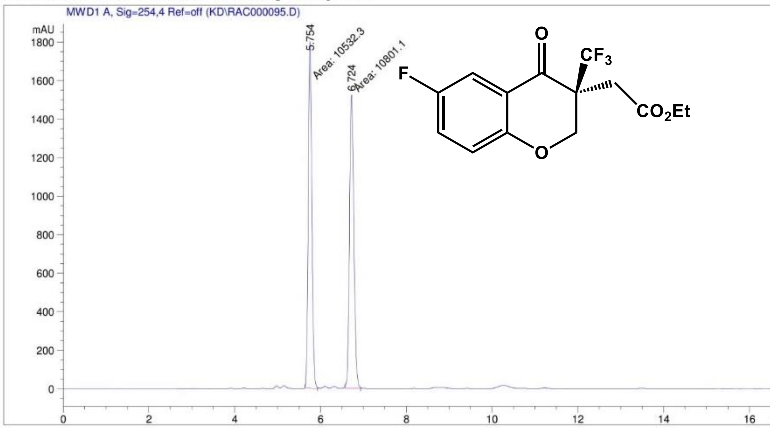

===
Area Percent Report
===

Sorted By : Signal
Multiplier: : 1.0000
Dilution: : 1.0000
Use Multiplier & Dilution Factor with ISTDs

Signal 1: MWD1 A, Sig=254,4 Ref=off

Peak #	RetTime [min]	Type	Width [min]	Area [mAU*s]	Height [mAU]	Area %
1	5.754	MM	0.0974	1.05323e4	1802.87878	49.3700
2	6.724	MM	0.1179	1.08011e4	1527.12366	50.6300

Additional Info : Peak(s) manually integrated

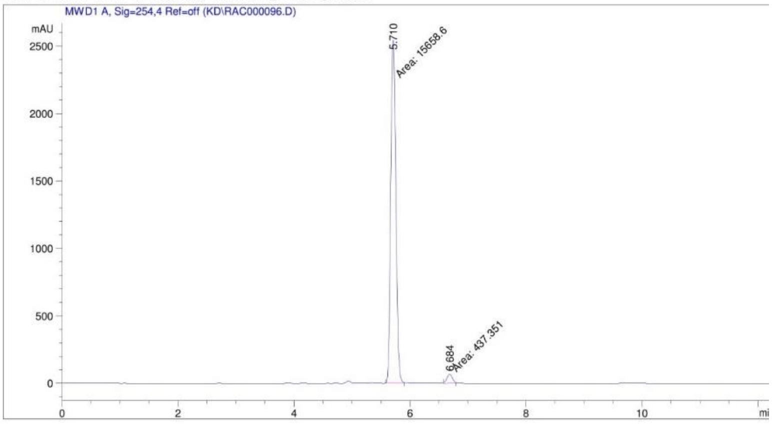

===
Area Percent Report
===

Sorted By : Signal
Multiplier: : 1.0000
Dilution: : 1.0000
Use Multiplier & Dilution Factor with ISTDs

Signal 1: MWD1 A, Sig=254,4 Ref=off

Peak #	RetTime [min]	Type	Width [min]	Area [mAU*s]	Height [mAU]	Area %
1	5.710	MM	0.1025	1.56586e4	2547.28711	97.2829
2	6.684	MM	0.1094	437.35129	66.63599	2.7171

Figure S74. HPLC chromatograms of **2d** (racemic – top, chiral – bottom).

Additional Info : Peak(s) manually integrated

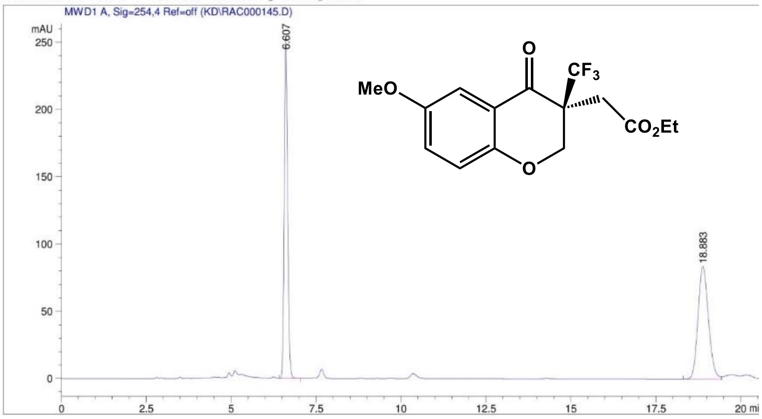

===

Area Percent Report

===

Sorted By : Signal
Multiplier: : 1.0000
Dilution: : 1.0000
Use Multiplier & Dilution Factor with ISTDs

Signal 1: MWD1 A, Sig=254,4 Ref=off

Peak #	RetTime [min]	Type	Width [min]	Area [mAU*s]	Height [mAU]	Area %
1	6.607	BB	0.1080	1766.10876	251.17668	49.6738
2	18.883	BV	0.3334	1789.30652	83.68530	50.3262

Additional Info : Peak(s) manually integrated

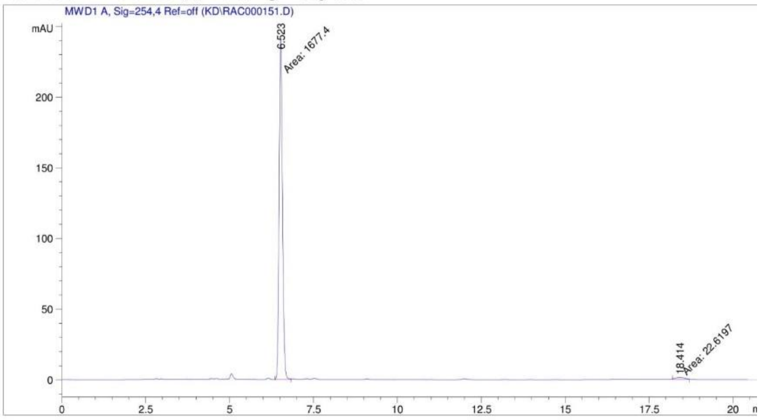

===

Area Percent Report

===

Sorted By : Signal
Multiplier: : 1.0000
Dilution: : 1.0000
Use Multiplier & Dilution Factor with ISTDs

Signal 1: MWD1 A, Sig=254,4 Ref=off

Peak #	RetTime [min]	Type	Width [min]	Area [mAU*s]	Height [mAU]	Area %
1	6.523	MM	0.1161	1677.40161	240.83060	98.6694
2	18.414	MM	0.2886	22.61974	1.30612	1.3306

Figure S75. HPLC chromatograms of **2e** (racemic – top, chiral – bottom).

Additional Info : Peak(s) manually integrated

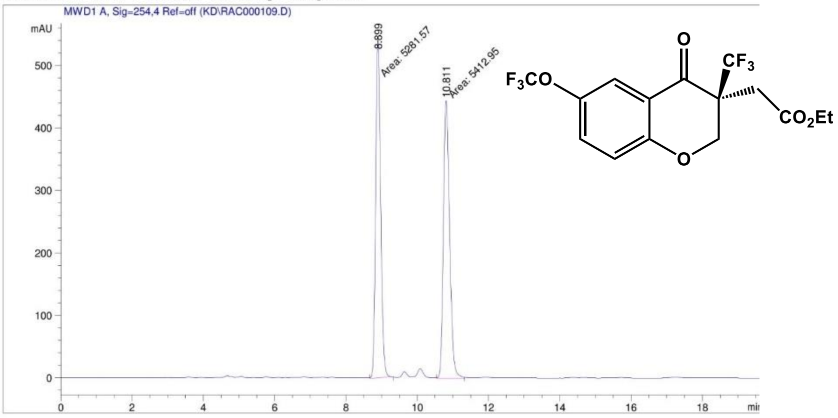

===
Area Percent Report
===

Sorted By : Signal
Multiplier: : 1.0000
Dilution: : 1.0000
Use Multiplier & Dilution Factor with ISTDs

Signal 1: MWD1 A, Sig=254,4 Ref=off

Peak #	RetTime [min]	Type	Width [min]	Area [mAU*s]	Height [mAU]	Area %
1	8.899	MM	0.1627	5281.56885	541.00964	49.3858
2	10.811	MM	0.2027	5412.94678	445.10895	50.6142

Additional Info : Peak(s) manually integrated

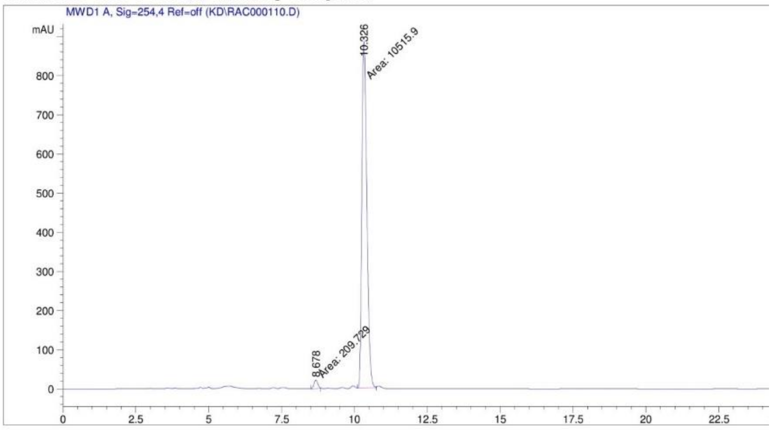

===
Area Percent Report
===

Sorted By : Signal
Multiplier: : 1.0000
Dilution: : 1.0000
Use Multiplier & Dilution Factor with ISTDs

Signal 1: MWD1 A, Sig=254,4 Ref=off

Peak #	RetTime [min]	Type	Width [min]	Area [mAU*s]	Height [mAU]	Area %
1	8.678	MM	0.1553	209.72903	22.50186	1.9554
2	10.326	MM	0.1979	1.05159e4	885.42780	98.0446

Figure S76. HPLC chromatograms of **2f** (racemic – top, chiral – bottom).

Additional Info : Peak(s) manually integrated

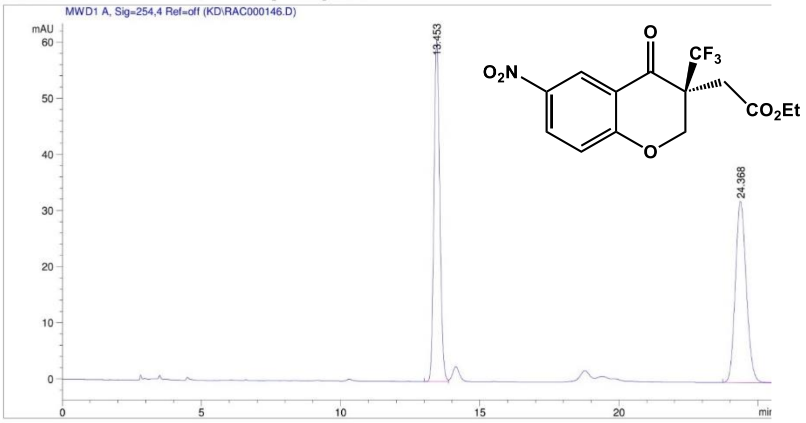

	Area Percent Report

Sorted By : Signal
Multiplier: : 1.0000
Dilution: : 1.0000
Use Multiplier & Dilution Factor with ISTDs

Signal 1: MWD1 A, Sig=254,4 Ref=off

Peak #	RetTime [min]	Type	Width [min]	Area [mAU*s]	Height [mAU]	Area %
1	13.453	BV	0.2230	880.48047	60.80042	50.0148
2	24.368	BB	0.4258	879.95844	32.30354	49.9852

chiralny

Additional Info : Peak(s) manually integrated

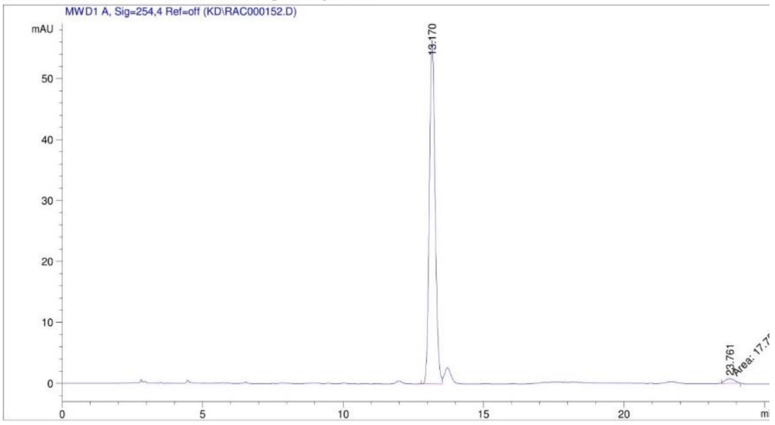

Area Percent Report

Sorted By : Signal
Multiplier: : 1.0000
Dilution: : 1.0000
Use Multiplier & Dilution Factor with ISTDs

Signal 1: MWD1 A, Sig=254,4 Ref=off

Peak #	RetTime [min]	Type	Width [min]	Area [mAU*s]	Height [mAU]	Area %
1	13.170	BV	0.2207	805.61493	56.38139	97.8439
2	23.761	MM	0.3818	17.75259	7.74966e-1	2.1561

Figure S77. HPLC chromatograms of **2g** (racemic – top, chiral – bottom).

Additional Info : Peak(s) manually integrated

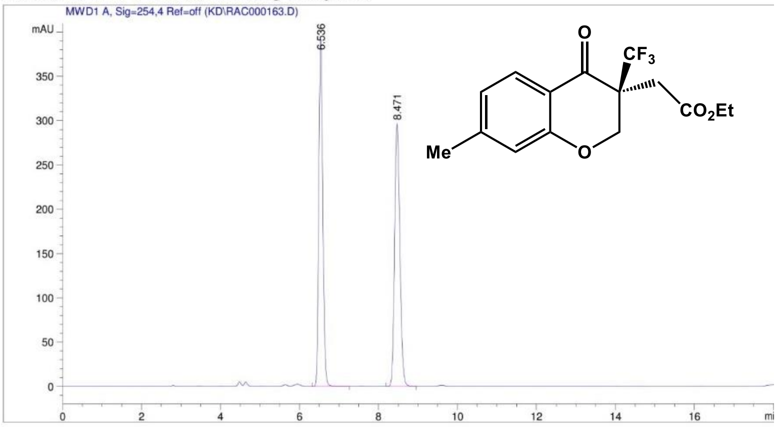

Area Percent Report

Sorted By : Signal
Multiplier: : 1.0000
Dilution: : 1.0000
Use Multiplier & Dilution Factor with ISTDs

Signal 1: MWD1 A, Sig=254,4 Ref=off

Peak #	RetTime [min]	Type	Width [min]	Area [mAU*s]	Height [mAU]	Area %
1	6.536	BB	0.1099	2743.41235	390.64825	50.0514
2	8.471	BB	0.1436	2737.77563	296.77371	49.9486

Additional Info : Peak(s) manually integrated

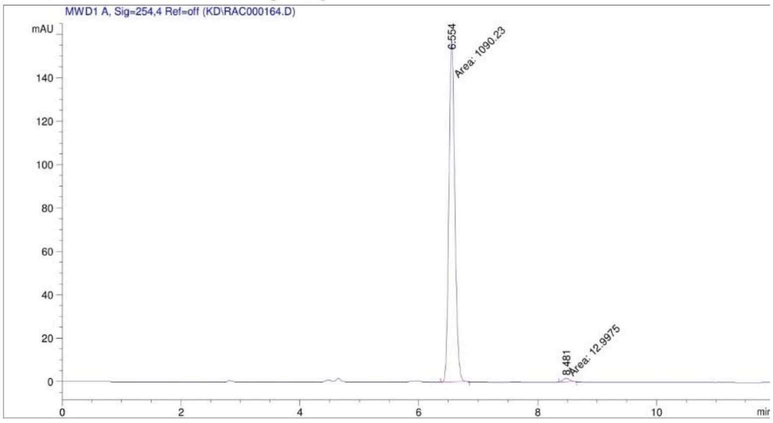

Area Percent Report

Sorted By : Signal
Multiplier: : 1.0000
Dilution: : 1.0000
Use Multiplier & Dilution Factor with ISTDs

Signal 1: MWD1 A, Sig=254,4 Ref=off

Peak #	RetTime [min]	Type	Width [min]	Area [mAU*s]	Height [mAU]	Area %
1	6.554	MM	0.1153	1090.23328	157.58357	98.8219
2	8.481	MM	0.1343	12.99750	1.61353	1.1781

Figure S78. HPLC chromatograms of **2h** (racemic – top, chiral – bottom).

Additional Info : Peak(s) manually integrated

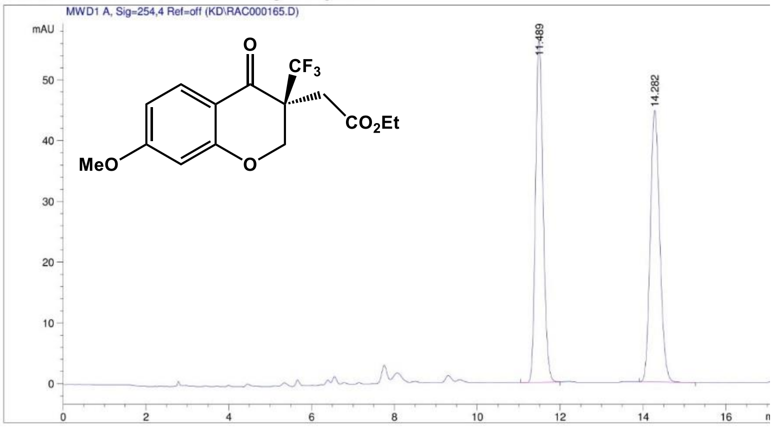

===
Area Percent Report
===

Sorted By : Signal
Multiplier: : 1.0000
Dilution: : 1.0000
Use Multiplier & Dilution Factor with ISTDs

Signal 1: MWD1 A, Sig=254,4 Ref=off

Peak #	RetTime [min]	Type	Width [min]	Area [mAU*s]	Height [mAU]	Area %
1	11.489	BB	0.1958	712.27753	56.23264	50.0898
2	14.282	BB	0.2452	709.72498	44.71370	49.9102

Additional Info : Peak(s) manually integrated

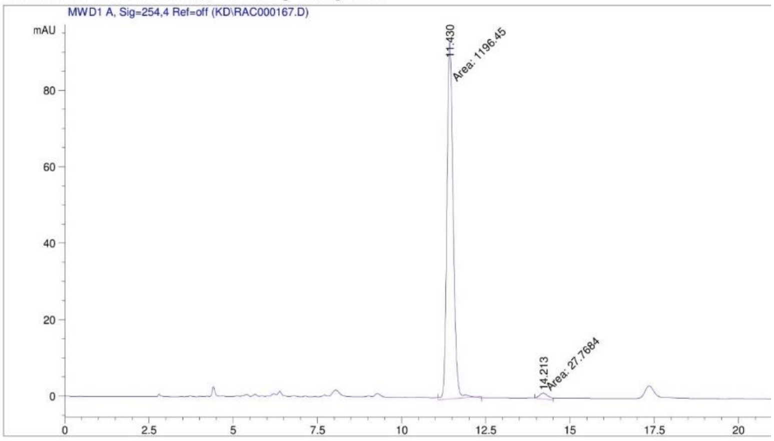

===
Area Percent Report
===

Sorted By : Signal
Multiplier: : 1.0000
Dilution: : 1.0000
Use Multiplier & Dilution Factor with ISTDs

Signal 1: MWD1 A, Sig=254,4 Ref=off

Peak #	RetTime [min]	Type	Width [min]	Area [mAU*s]	Height [mAU]	Area %
1	11.430	MM	0.2135	1196.45215	93.39294	97.7317
2	14.213	MM	0.3103	27.76842	1.49147	2.2683

Figure S79. HPLC chromatograms of **2i** (racemic – top, chiral – bottom).

Additional Info : Peak(s) manually integrated

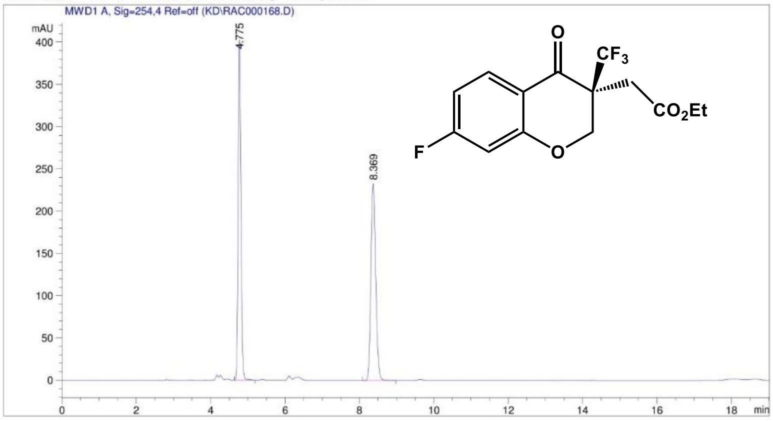

==

Area Percent Report

==

Sorted By : Signal
Multiplier: : 1.0000
Dilution: : 1.0000
Use Multiplier & Dilution Factor with ISTDs

Signal 1: MWD1 A, Sig=254,4 Ref=off

Peak #	RetTime [min]	Type	Width [min]	Area [mAU*s]	Height [mAU]	Area %
1	4.775	BB	0.0802	2078.00562	402.87070	49.9210
2	8.369	BB	0.1382	2084.58594	233.37201	50.0790

IB-chromanon-7-F-Chiralny

Additional Info : Peak(s) manually integrated

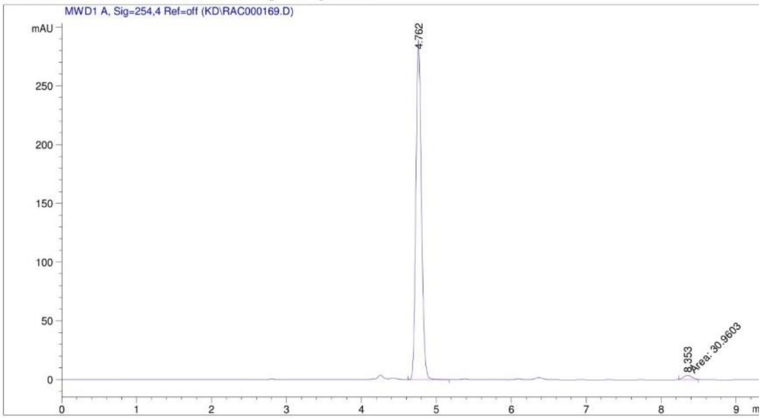

==

Area Percent Report

==

Sorted By : Signal
Multiplier: : 1.0000
Dilution: : 1.0000
Use Multiplier & Dilution Factor with ISTDs

Signal 1: MWD1 A, Sig=254,4 Ref=off

Peak #	RetTime [min]	Type	Width [min]	Area [mAU*s]	Height [mAU]	Area %
1	4.762	VV	0.0799	1487.90442	289.79050	97.9616
2	8.353	MM	0.1422	30.96031	3.62770	2.0384

Figure S80. HPLC chromatograms of **2j** (racemic – top, chiral – bottom).

Additional Info : Peak(s) manually integrated

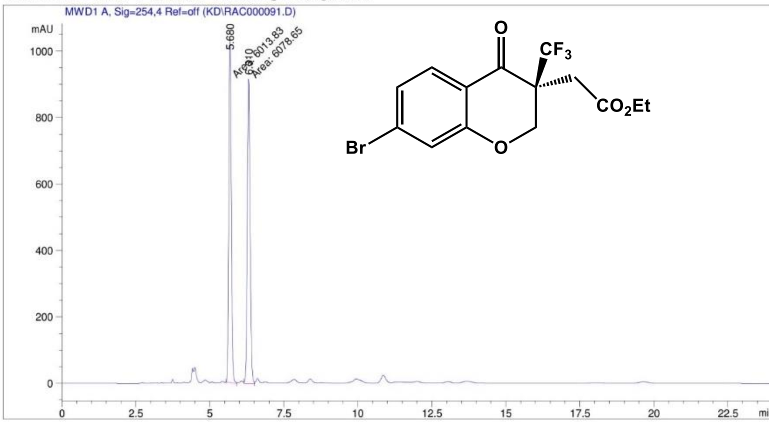

Area Percent Report

Sorted By : Signal
Multiplier: : 1.0000
Dilution: : 1.0000
Use Multiplier & Dilution Factor with ISTDs

Signal 1: MWD1 A, Sig=254,4 Ref=off

Peak #	RetTime [min]	Type	Width [min]	Area [mAU*s]	Height [mAU]	Area %
1	5.680	MM	0.0972	6013.83057	1030.98682	49.7320
2	6.310	MM	0.1100	6078.64746	920.80743	50.2680

Additional Info : Peak(s) manually integrated

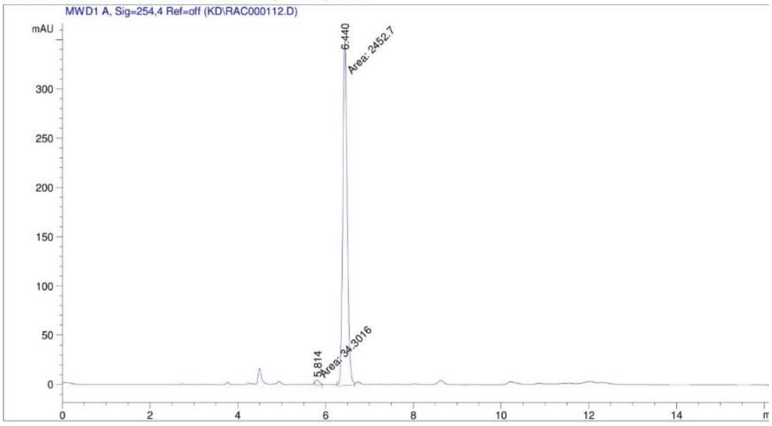

Area Percent Report

Sorted By : Signal
Multiplier: : 1.0000
Dilution: : 1.0000
Use Multiplier & Dilution Factor with ISTDs

Signal 1: MWD1 A, Sig=254,4 Ref=off

Peak #	RetTime [min]	Type	Width [min]	Area [mAU*s]	Height [mAU]	Area %
1	5.814	MM	0.1113	34.30164	5.13580	1.3792
2	6.440	MM	0.1165	2452.70190	350.76987	98.6208

Figure S81. HPLC chromatograms of **2k** (racemic – top, chiral – bottom).

Additional Info : Peak(s) manually integrated

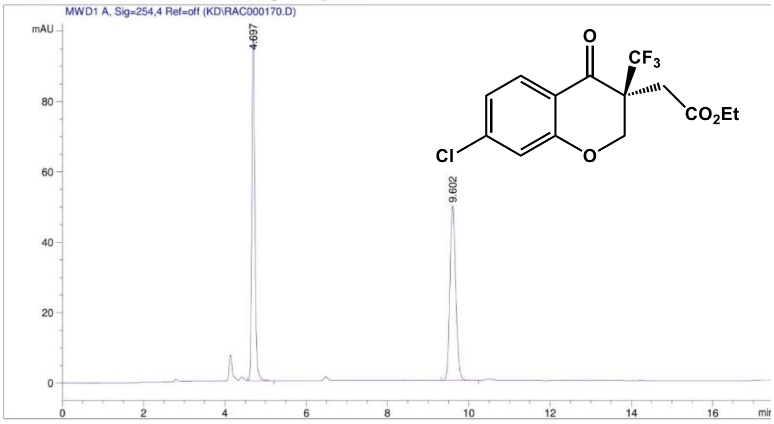

Area Percent Report

Sorted By : Signal
Multiplier: : 1.0000
Dilution: : 1.0000
Use Multiplier & Dilution Factor with ISTDs

Signal 1: MWD1 A, Sig=254,4 Ref=off

Peak #	RetTime [min]	Type	Width [min]	Area [mAU*s]	Height [mAU]	Area %
1	4.697	VB	0.0810	507.81110	97.05500	50.5703
2	9.602	BB	0.1545	496.35736	49.69268	49.4297

Additional Info : Peak(s) manually integrated

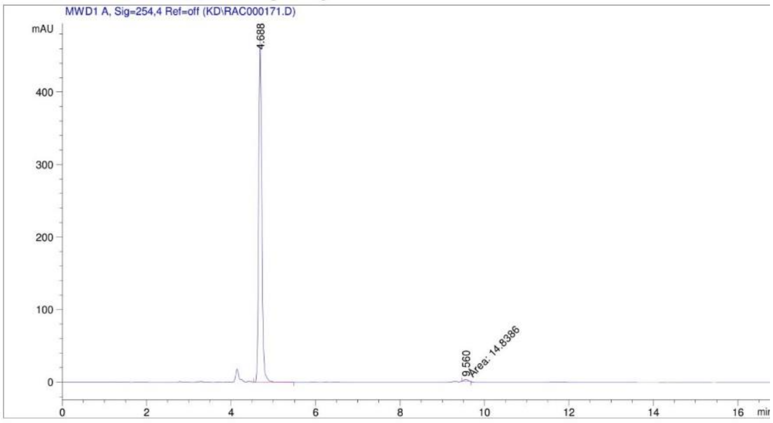

Area Percent Report

Sorted By : Signal
Multiplier: : 1.0000
Dilution: : 1.0000
Use Multiplier & Dilution Factor with ISTDs

Signal 1: MWD1 A, Sig=254,4 Ref=off

Peak #	RetTime [min]	Type	Width [min]	Area [mAU*s]	Height [mAU]	Area %
1	4.688	BB	0.0803	2439.91943	471.89246	99.3955
2	9.560	MM	0.1078	14.83860	2.29469	0.6045

Figure S82. HPLC chromatograms of **2l** (racemic – top, chiral – bottom).

Additional Info : Peak(s) manually integrated

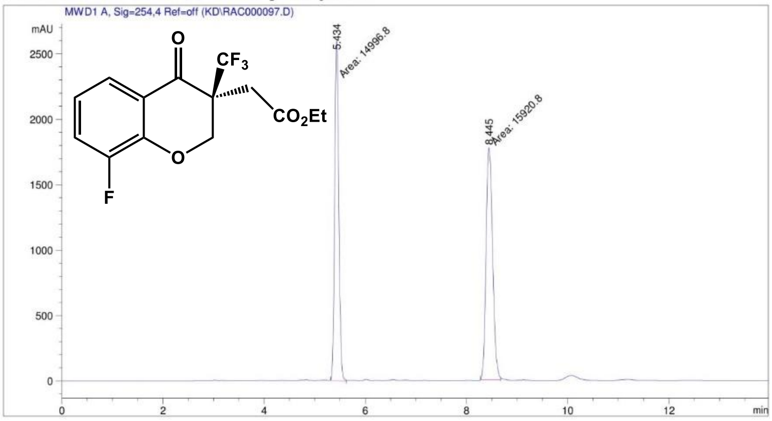


```
=====================================================================
                        Area Percent Report
=====================================================================

Sorted By             :      Signal
Multiplier:                   :      1.0000
Dilution:                     :      1.0000
Use Multiplier & Dilution Factor with ISTDs


Signal 1: MWD1 A, Sig=254,4 Ref=off
```

Peak #	RetTime [min]	Type	Width [min]	Area [mAU*s]	Height [mAU]	Area %
1	5.434	MM	0.0960	1.49968e4	2604.58667	48.5057
2	8.445	MM	0.1496	1.59208e4	1773.58411	51.4943

[illegible]

Additional Info : Peak(s) manually integrated

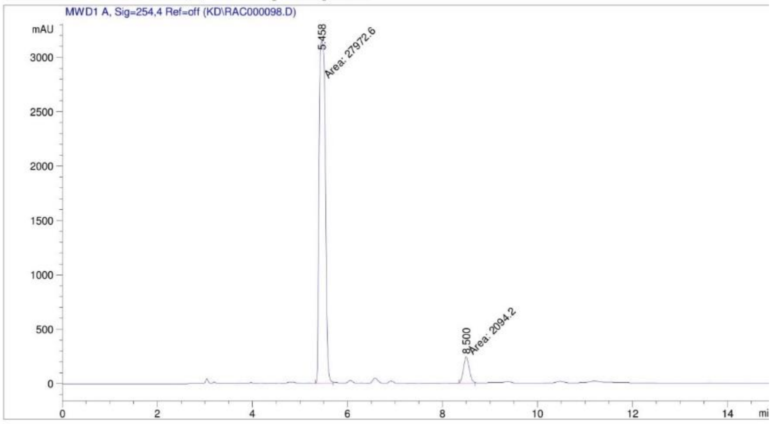


```
=====================================================================
                        Area Percent Report
=====================================================================

Sorted By             :      Signal
Multiplier:                   :      1.0000
Dilution:                     :      1.0000
Use Multiplier & Dilution Factor with ISTDs


Signal 1: MWD1 A, Sig=254,4 Ref=off
```

Peak #	RetTime [min]	Type	Width [min]	Area [mAU*s]	Height [mAU]	Area %
1	5.458	MM	0.1481	2.79726e4	3147.81030	93.0349
2	8.500	MM	0.1442	2094.19849	242.02745	6.9651

Figure S83. HPLC chromatograms of **2m** (racemic – top, chiral – bottom).

Additional Info : Peak(s) manually integrated

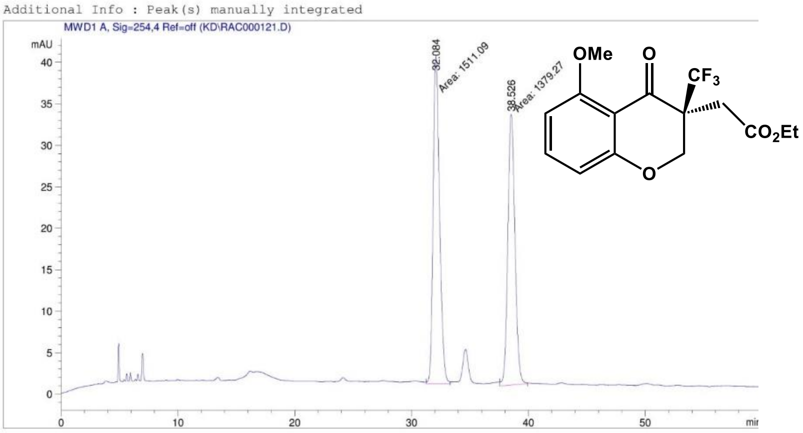


```
=====================================================================
                         Area Percent Report
=====================================================================

Sorted By             :      Signal
Multiplier:                   :      1.0000
Dilution:                     :      1.0000
Use Multiplier & Dilution Factor with ISTDs


Signal 1: MWD1 A, Sig=254,4 Ref=off
```

Peak #	RetTime [min]	Type	Width [min]	Area [mAU*s]	Height [mAU]	Area %
1	32.084	MM	0.6367	1511.09448	39.55570	52.2804
2	38.526	MM	0.7047	1379.27039	32.61897	47.7196

Additional Info : Peak(s) manually integrated

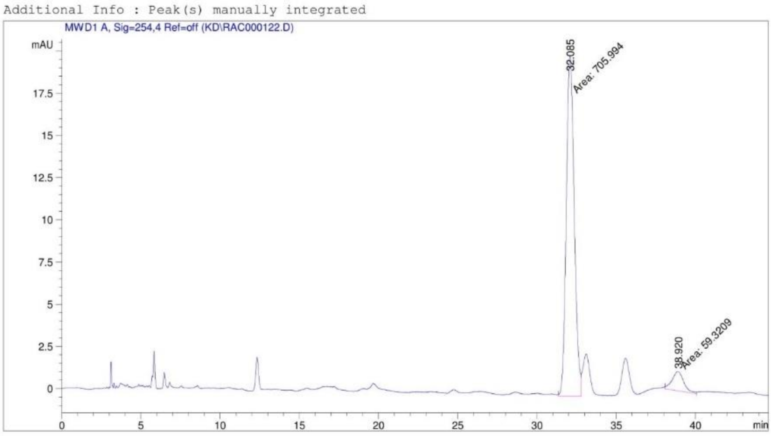


```
=====================================================================
                         Area Percent Report
=====================================================================

Sorted By             :      Signal
Multiplier:                   :      1.0000
Dilution:                     :      1.0000
Use Multiplier & Dilution Factor with ISTDs


Signal 1: MWD1 A, Sig=254,4 Ref=off
```

Peak #	RetTime [min]	Type	Width [min]	Area [mAU*s]	Height [mAU]	Area %
1	32.085	MF	0.5839	705.99414	20.15011	92.2488
2	38.920	MM	0.8558	59.32093	1.15531	7.7512

Figure S84. HPLC chromatograms of **2n** (racemic – top, chiral – bottom).

Additional Info : Peak(s) manually integrated

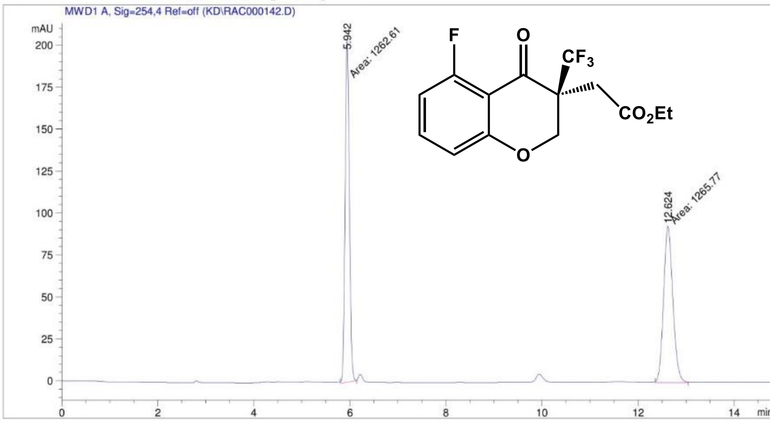

Area Percent Report

Sorted By : Signal
Multiplier: : 1.0000
Dilution: : 1.0000
Use Multiplier & Dilution Factor with ISTDs

Signal 1: MWD1 A, Sig=254,4 Ref=off

Peak #	RetTime [min]	Type	Width [min]	Area [mAU*s]	Height [mAU]	Area %
1	5.942	MM	0.1030	1262.61096	204.23894	49.9376
2	12.624	MM	0.2266	1265.76733	93.10606	50.0624

Additional Info : Peak(s) manually integrated

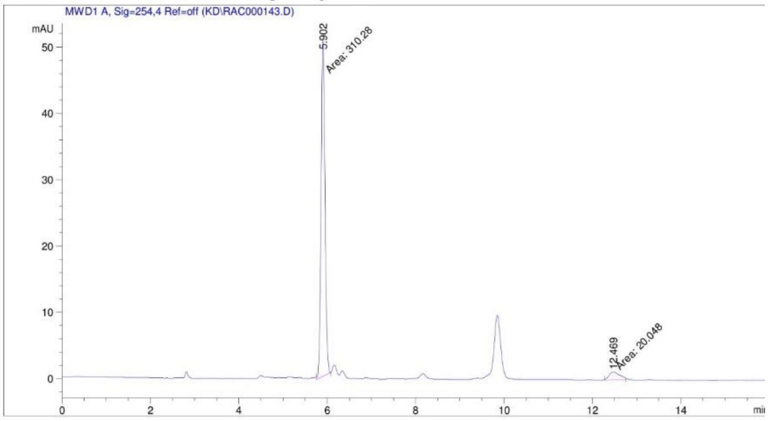

Area Percent Report

Sorted By : Signal
Multiplier: : 1.0000
Dilution: : 1.0000
Use Multiplier & Dilution Factor with ISTDs

Signal 1: MWD1 A, Sig=254,4 Ref=off

Peak #	RetTime [min]	Type	Width [min]	Area [mAU*s]	Height [mAU]	Area %
1	5.902	MM	0.1018	310.27972	50.81746	93.9309
2	12.469	MM	0.2787	20.04798	1.19876	6.0691

Figure S85. HPLC chromatograms of **2o** (racemic – top, chiral – bottom).

Additional Info : Peak(s) manually integrated

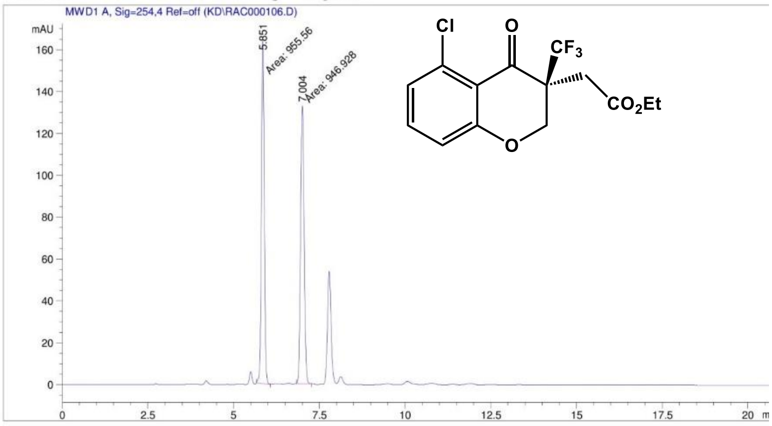

Area Percent Report

Sorted By : Signal
Multiplier: : 1.0000
Dilution: : 1.0000
Use Multiplier & Dilution Factor with ISTDs

Signal 1: MWD1 A, Sig=254,4 Ref=off

Peak #	RetTime [min]	Type	Width [min]	Area [mAU*s]	Height [mAU]	Area %
1	5.851	MM	0.0969	955.55994	164.38503	50.2269
2	7.004	MM	0.1183	946.92792	133.43764	49.7731

Additional Info : Peak(s) manually integrated

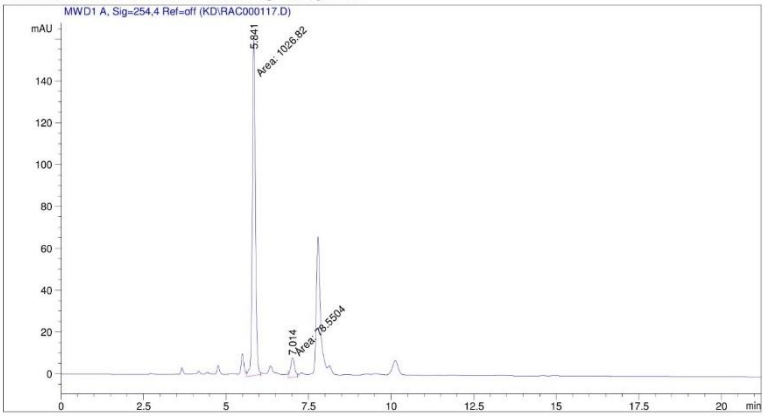

Area Percent Report

Sorted By : Signal
Multiplier: : 1.0000
Dilution: : 1.0000
Use Multiplier & Dilution Factor with ISTDs

Signal 1: MWD1 A, Sig=254,4 Ref=off

Peak #	RetTime [min]	Type	Width [min]	Area [mAU*s]	Height [mAU]	Area %
1	5.841	MM	0.1066	1026.81860	160.61009	92.8937
2	7.014	MM	0.1432	78.55035	9.14384	7.1063

Figure S86. HPLC chromatograms of **2p** (racemic – top, chiral – bottom).

Additional Info : Peak(s) manually integrated

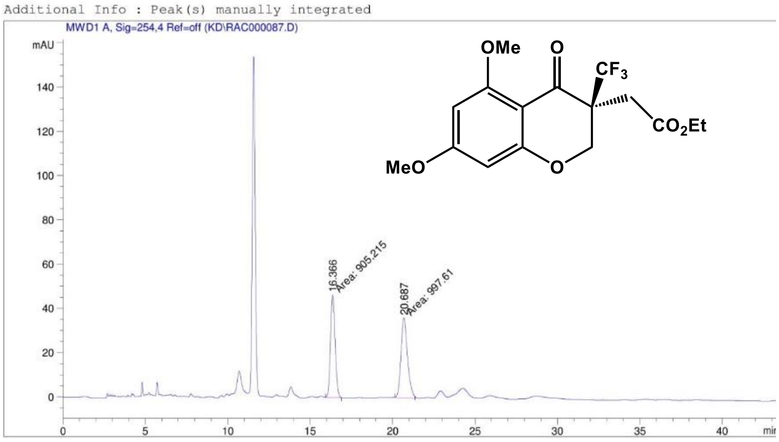

Area Percent Report

Sorted By : Signal
Multiplier: : 1.0000
Dilution: : 1.0000
Use Multiplier & Dilution Factor with ISTDs

Signal 1: MWD1 A, Sig=254,4 Ref=off

Peak #	RetTime [min]	Type	Width [min]	Area [mAU*s]	Height [mAU]	Area %
1	16.366	MM	0.3253	905.21484	46.37756	47.5722
2	20.687	MM	0.4608	997.60962	36.08604	52.4278

[illegible]

Additional Info : Peak(s) manually integrated

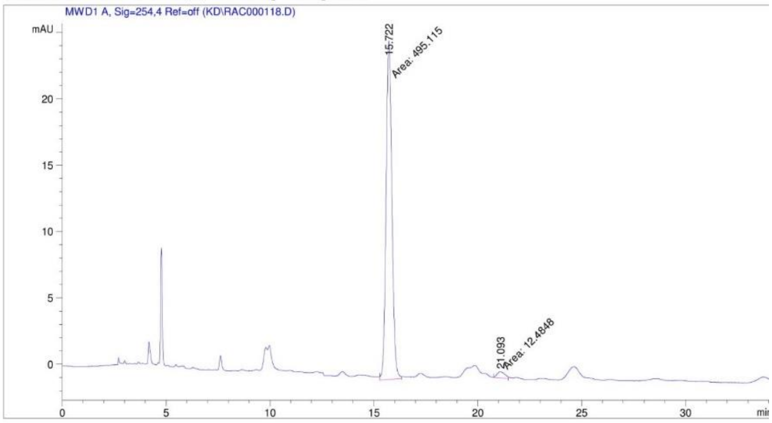

Area Percent Report

Sorted By : Signal
Multiplier: : 1.0000
Dilution: : 1.0000
Use Multiplier & Dilution Factor with ISTDs

Signal 1: MWD1 A, Sig=254,4 Ref=off

Peak #	RetTime [min]	Type	Width [min]	Area [mAU*s]	Height [mAU]	Area %
1	15.722	MM	0.3230	495.11469	25.54409	97.5404
2	21.093	MM	0.4391	12.48480	4.73880e-1	2.4596

Figure S87. HPLC chromatograms of **2q** (racemic – top, chiral – bottom).

Additional Info : Peak(s) manually integrated

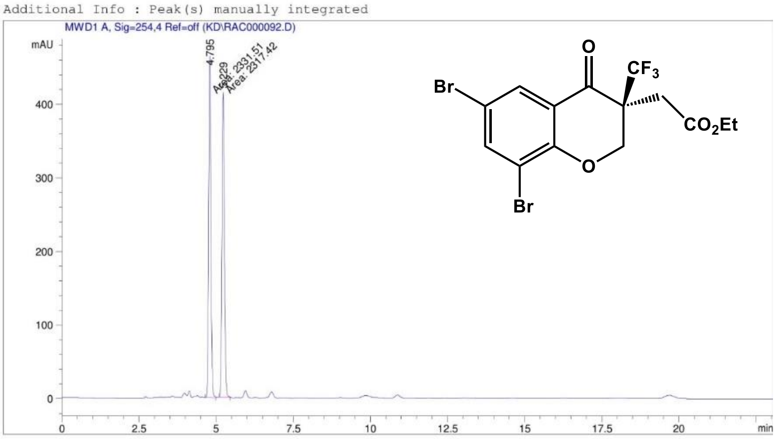

===

Area Percent Report

===

Sorted By : Signal
Multiplier: : 1.0000
Dilution: : 1.0000
Use Multiplier & Dilution Factor with ISTDs

Signal 1: MWD1 A, Sig=254,4 Ref=off

Peak #	RetTime [min]	Type	Width [min]	Area [mAU*s]	Height [mAU]	Area %
1	4.795	MM	0.0835	2331.51367	465.19107	50.1516
2	5.229	MM	0.0929	2317.42017	415.53836	49.8484

Additional Info : Peak(s) manually integrated

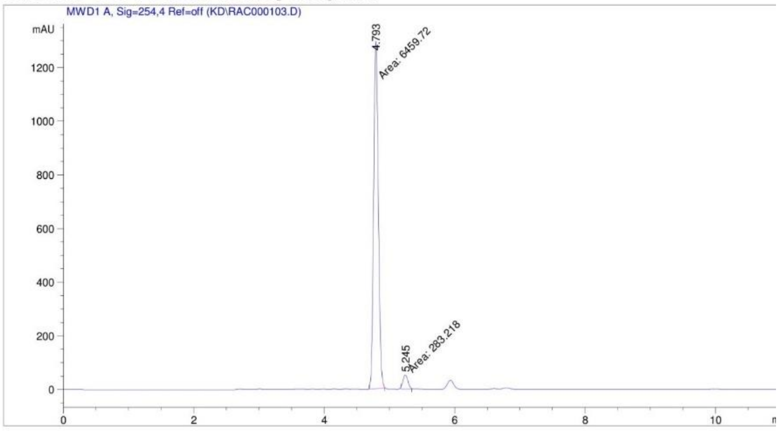

===

Area Percent Report

===

Sorted By : Signal
Multiplier: : 1.0000
Dilution: : 1.0000
Use Multiplier & Dilution Factor with ISTDs

Signal 1: MWD1 A, Sig=254,4 Ref=off

Peak #	RetTime [min]	Type	Width [min]	Area [mAU*s]	Height [mAU]	Area %
1	4.793	MM	0.0830	6459.71826	1297.89722	95.7998
2	5.245	MM	0.0884	283.21793	53.41651	4.2002

Figure S88. HPLC chromatograms of **2r** (racemic – top, chiral – bottom).

Additional Info : Peak(s) manually integrated

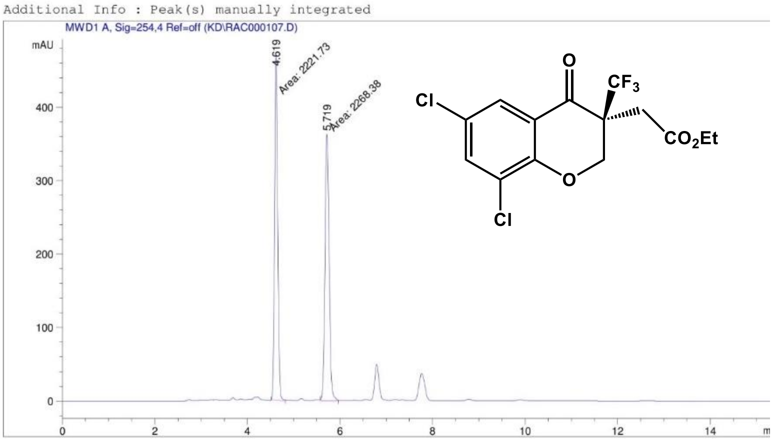

===

Area Percent Report

===

Sorted By : Signal
Multiplier: : 1.0000
Dilution: : 1.0000
Use Multiplier & Dilution Factor with ISTDs

Signal 1: MWD1 A, Sig=254,4 Ref=off

Peak #	RetTime [min]	Type	Width [min]	Area [mAU*s]	Height [mAU]	Area %
1	4.619	MM	0.0789	2221.72803	469.09247	49.4805
2	5.719	MM	0.1039	2268.38452	363.79507	50.5195

Additional Info : Peak(s) manually integrated

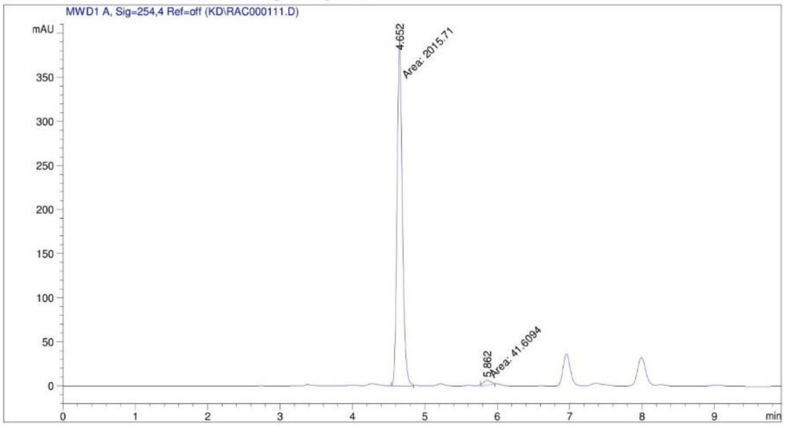

===

Area Percent Report

===

Sorted By : Signal
Multiplier: : 1.0000
Dilution: : 1.0000
Use Multiplier & Dilution Factor with ISTDs

Signal 1: MWD1 A, Sig=254,4 Ref=off

Peak #	RetTime [min]	Type	Width [min]	Area [mAU*s]	Height [mAU]	Area %
1	4.652	MM	0.0855	2015.71204	392.87918	97.9775
2	5.862	MM	0.1200	41.60942	5.77833	2.0225

Figure S89. HPLC chromatograms of **2s** (racemic – top, chiral – bottom).

[illegible]

Additional Info : Peak(s) manually integrated

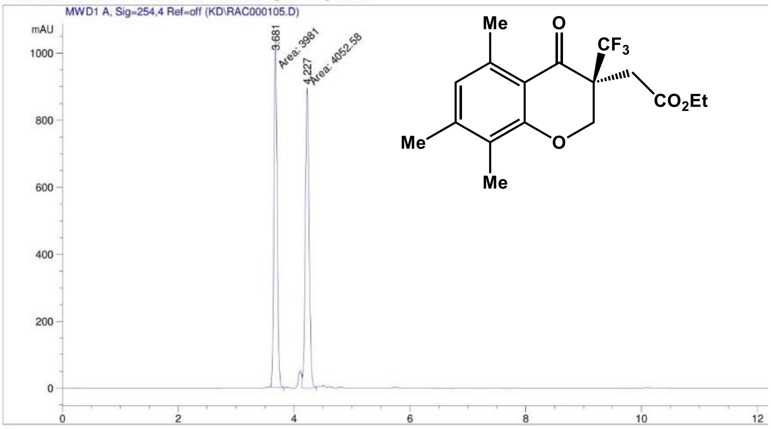

==

Area Percent Report

==

Sorted By : Signal
Multiplier: : 1.0000
Dilution: : 1.0000
Use Multiplier & Dilution Factor with ISTDs

Signal 1: MWD1 A, Sig=254,4 Ref=off

Peak #	RetTime [min]	Type	Width [min]	Area [mAU*s]	Height [mAU]	Area %
1	3.681	MM	0.0640	3981.00415	1036.35242	49.5545
2	4.227	FM	0.0752	4052.57983	897.63593	50.4455

[illegible]

Additional Info : Peak(s) manually integrated

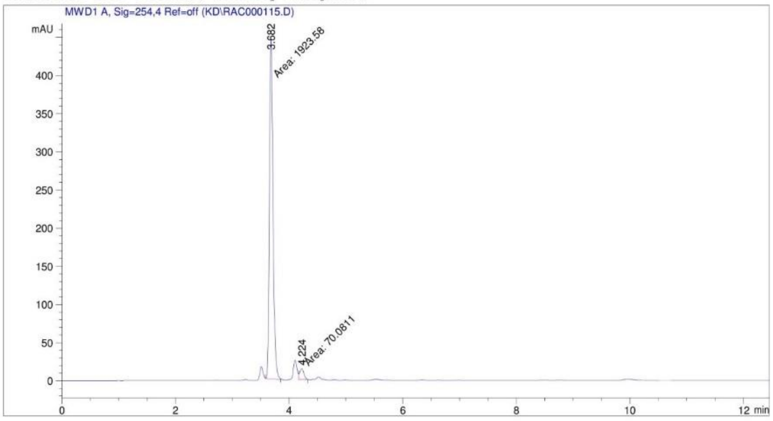

==

Area Percent Report

==

Sorted By : Signal
Multiplier: : 1.0000
Dilution: : 1.0000
Use Multiplier & Dilution Factor with ISTDs

Signal 1: MWD1 A, Sig=254,4 Ref=off

Peak #	RetTime [min]	Type	Width [min]	Area [mAU*s]	Height [mAU]	Area %
1	3.682	MM	0.0719	1923.58142	446.11127	96.4848
2	4.224	FM	0.0803	70.08110	14.54288	3.5152

Figure S90. HPLC chromatograms of **2t** (racemic – top, chiral – bottom).

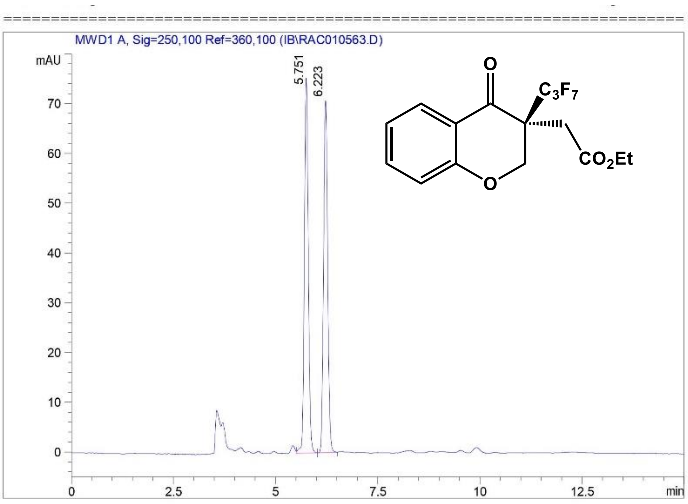

#	Meas. R	Area	Area %
1	5.751	501.738	50.420
2	6.223	493.377	49.580

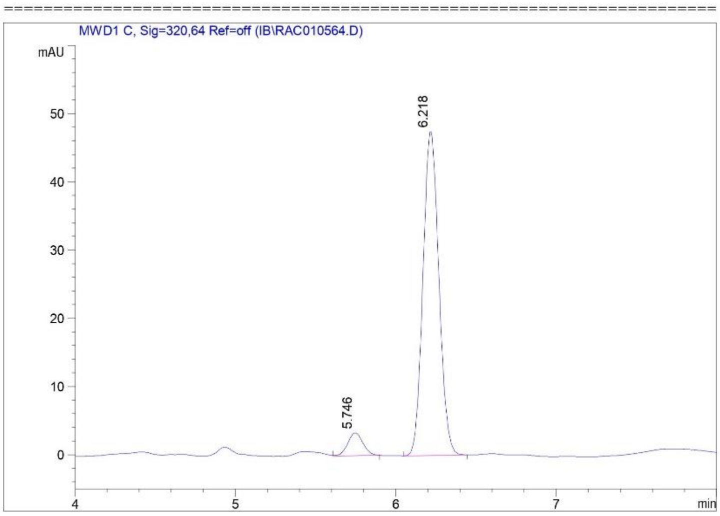

#	Meas. R	Area	Area %
1	5.746	21.917	6.301
2	6.218	325.911	93.699

Figure S91. HPLC chromatograms of **2u** (racemic – top, chiral – bottom).

Additional Info : Peak(s) manually integrated

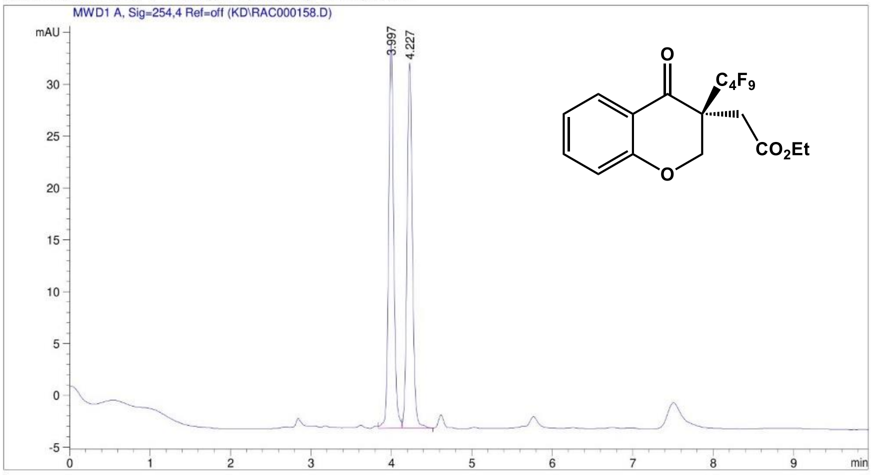

Area Percent Report

Sorted By : Signal
Multiplier: : 1.0000
Dilution: : 1.0000
Use Multiplier & Dilution Factor with ISTDs

Signal 1: MWD1 A, Sig=254,4 Ref=off

Peak #	RetTime [min]	Type	Width [min]	Area [mAU*s]	Height [mAU]	Area %
1	3.997	VV	0.0676	164.49899	37.17786	50.3064
2	4.227	VB	0.0718	162.49532	35.21177	49.6936

Last Changed : [illegible]

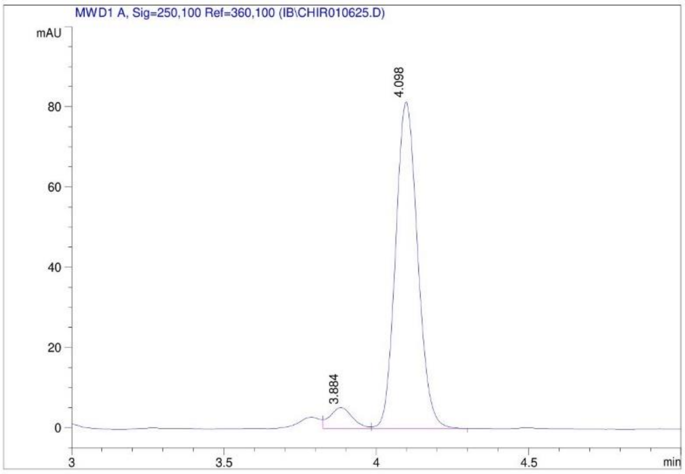

#	Meas. R	Area	Area %
1	3.884	27.030	6.185
2	4.098	410.022	93.815

Figure S92. HPLC chromatograms of **2v** (racemic – top, chiral – bottom).

Additional Info : Peak(s) manually integrated

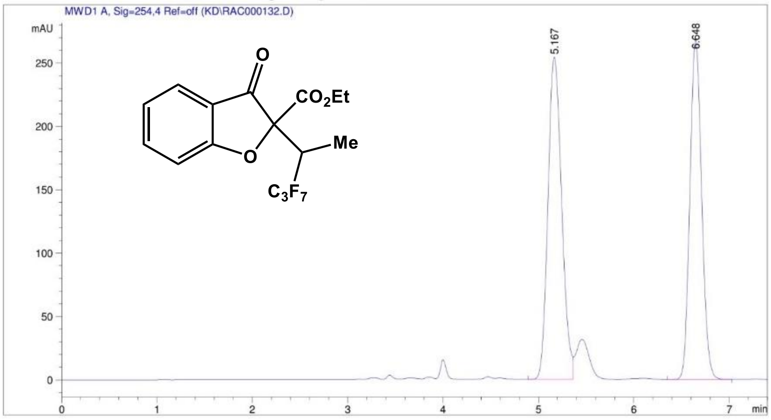

===
Area Percent Report
===

Sorted By : Signal
Multiplier: : 1.0000
Dilution: : 1.0000
Use Multiplier & Dilution Factor with ISTDs

Signal 1: MWD1 A, Sig=254,4 Ref=off

Peak #	RetTime [min]	Type	Width [min]	Area [mAU*s]	Height [mAU]	Area %
1	5.167	BV	0.1592	2553.17676	254.19301	52.7691
2	6.648	BV	0.1334	2285.21411	268.13779	47.2309

Additional Info : Peak(s) manually integrated

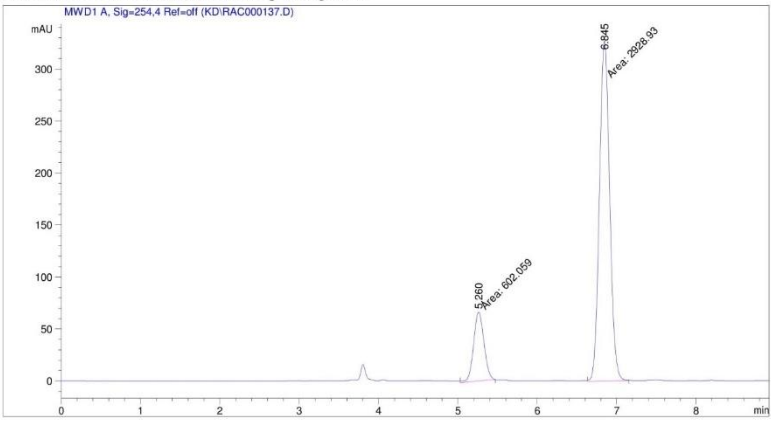

===
Area Percent Report
===

Sorted By : Signal
Multiplier: : 1.0000
Dilution: : 1.0000
Use Multiplier & Dilution Factor with ISTDs

Signal 1: MWD1 A, Sig=254,4 Ref=off

Peak #	RetTime [min]	Type	Width [min]	Area [mAU*s]	Height [mAU]	Area %
1	5.260	MM	0.1513	602.05945	66.31876	17.0507
2	6.845	MM	0.1489	2928.93262	327.83795	82.9493

Figure S93. HPLC chromatograms of **4a** (racemic – top, chiral – bottom).

Last changed : 2019-04-> 11:45:22

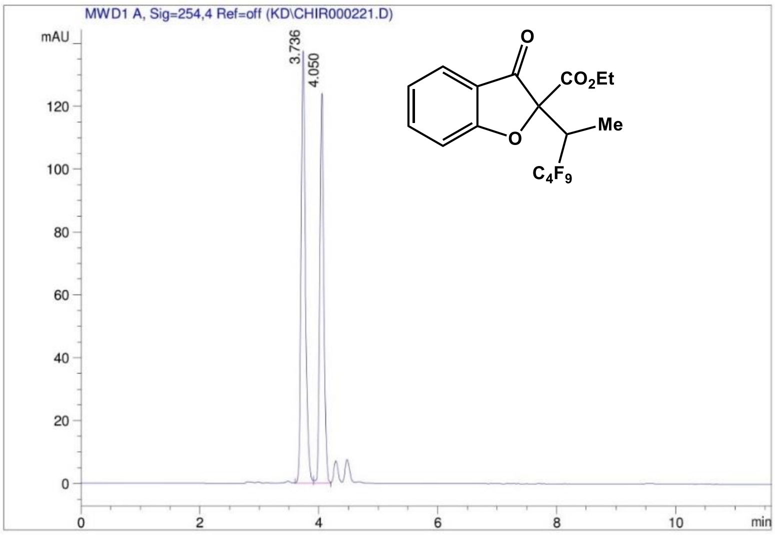

#	Meas. R	Area	Area %
1	3.736	687.222	54.014
2	4.050	585.085	45.986

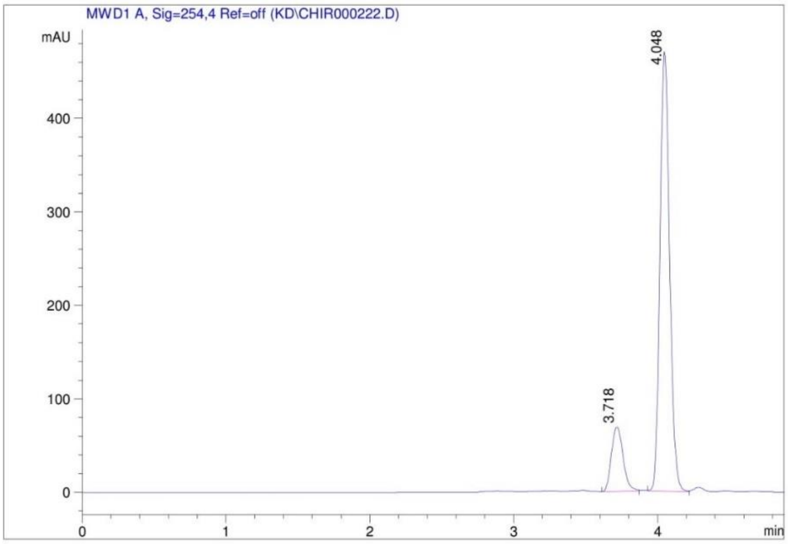

#	Meas. R	Area	Area %
1	3.718	388.786	15.100
2	4.048	2.186e3	84.900

Figure S94. HPLC chromatograms of **4b** (racemic – top, chiral – bottom).

Additional Info : Peak(s) manually integrated

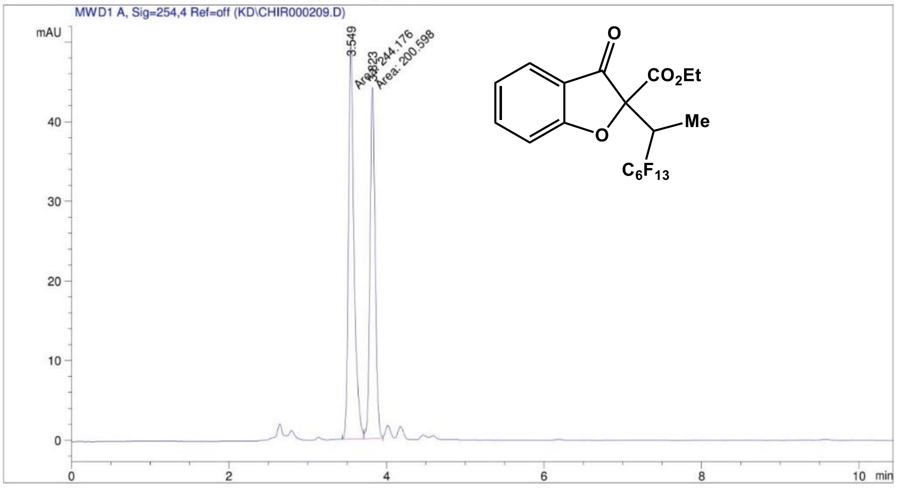

Peak #	RetTime [min]	Type	Width [min]	Area [mAU*s]	Height [mAU]	Area %
1	3.549	MF	0.0820	244.17551	49.60939	54.8989
2	3.823	FM	0.0754	200.59781	44.36678	45.1011
Totals :				444.77332	93.97617	

Additional Info : Peak(s) manually integrated

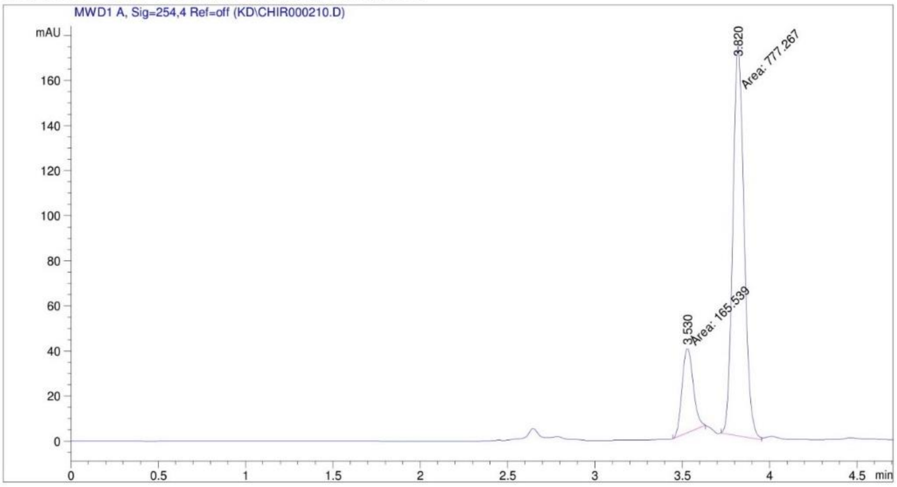

Peak #	RetTime [min]	Type	Width [min]	Area [mAU*s]	Height [mAU]	Area %
1	3.530	MM	0.0737	165.53903	37.45336	17.5581
2	3.820	MM	0.0747	777.26709	173.39758	82.4419
Totals :				942.80612	210.85094	

Figure S95. HPLC chromatograms of **4c** (racemic – top, chiral – bottom).

Last changed : 2019-04-> 14:02:21

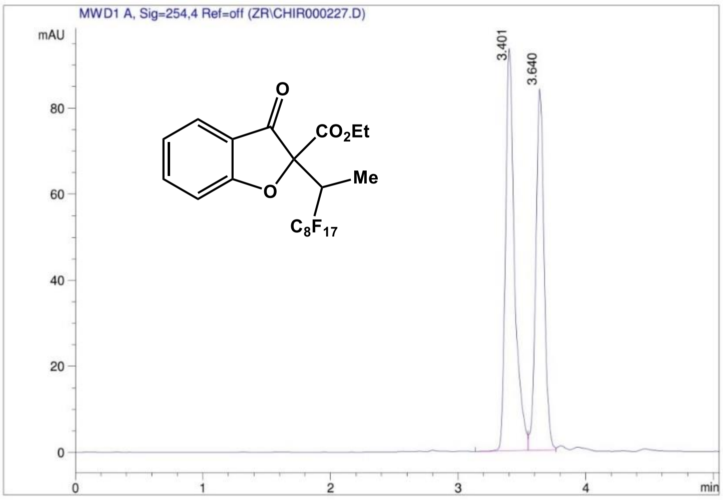

#	Meas. R	Area	Area %
1	3.401	462.126	54.885
2	3.640	379.863	45.115

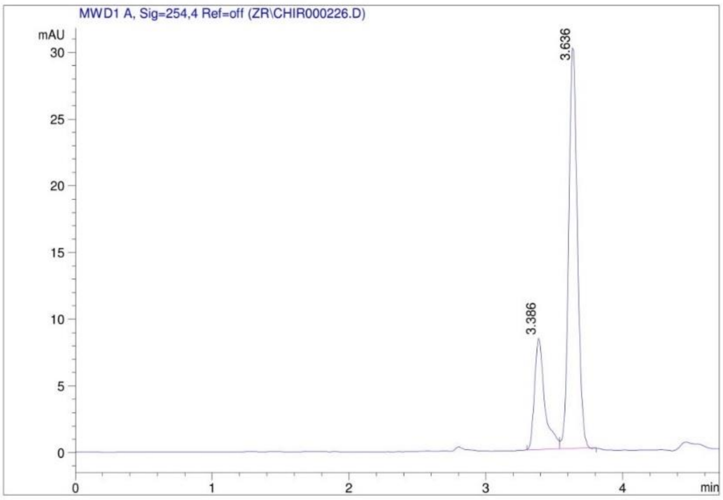

#	Meas. R	Area	Area %
1	3.386	43.130	24.464
2	3.636	133.173	75.536

Figure S96. HPLC chromatograms of **4d** (racemic – top, chiral – bottom).

6. Literature

(1) Abele, H.; Haas, A.; Lieb, M.; Zwingenber, *J. Fluor. Chem.* **1993**, 62, 1, 25-29.

(2) Yamazaki, T.; Mano, N.; Hikage, R.; Kaneko, T.; Kawasaki-Takasuka, T.; Yamada, S. *Tetrahedron* **2015**, 71, 8059-8066.

(3) Mannisto, J. K.; Sahari, A.; Lagerblom, K.; Niemi, T.; Nieger, M.; Sztanó, G.; Repo, T. *Chem. Eur. J.* **2019**, 25, 44, 10284-10289.
